# Neotenic phenomenon in gene expression in the skin of Foxn1- deficient (nude) mice - a projection for regenerative skin wound healing

**DOI:** 10.1186/s12864-016-3401-z

**Published:** 2017-01-09

**Authors:** Anna Kur-Piotrowska, Marta Kopcewicz, Leslie P. Kozak, Pawel Sachadyn, Anna Grabowska, Barbara Gawronska-Kozak

**Affiliations:** 1 Institute of Animal Reproduction and Food Research, Polish Academy of Sciences, ul. Tuwima 10, 10-748 Olsztyn, Poland; 2 Department of Molecular Biotechnology and Microbiology, Gdansk University of Technology, ul. G. Narutowicza 11/12, 80-233 Gdansk, Poland

**Keywords:** Transcriptome, Next-generation sequencing, Regeneration, Mmp-9

## Abstract

**Background:**

Mouse fetuses up to 16 day of embryonic development and nude (Foxn1- deficient) mice are examples of animals that undergo regenerative (scar-free) skin healing. The expression of transcription factor Foxn1 in the epidermis of mouse fetuses begins at embryonic day 16.5 which coincides with the transition point from scar-free to scar-forming skin wound healing. In the present study, we tested the hypothesis that Foxn1 expression in the skin is an essential condition to establish the adult skin phenotype and that Foxn1 inactivity in nude mice keeps skin in the immature stage resembling the phenomena of neoteny.

**Results:**

Uninjured skin of adult C57BL/6J (B6) mice, mouse fetuses at days 14 (E14) and 18 (E18) of embryonic development and B6.Cg-Foxn1 nu (nude) mice were characterized for their gene expression profiles by RNA sequencing that was validated through qRT-PCR, Western Blot and immunohistochemistry. Differentially regulated genes indicated that nude mice were more similar to E14 (model of regenerative healing) and B6 were more similar to E18 (model of reparative healing). The up-regulated genes in nude and E14 mice were associated with tissue remodeling, cytoskeletal rearrangement, wound healing and immune response, whereas the down-regulated genes were associated with differentiation. E14 and nude mice exhibit prominent up-regulation of keratin (Krt23, -73, -82, -16, -17), involucrin (Ivl) and filaggrin (Flg2) genes. The transcription factors associated with the Hox genes known to specify cell fate during embryonic development and promote embryonic stem cells differentiation were down-regulated in both nude and E14. Among the genes enriched in the nude skin but not shared with E14 fetuses were members of the Wnt and matrix metalloproteinases (Mmps) families whereas Bmp and Notch related genes were down-regulated.

**Conclusions:**

In summary, Foxn1 appears to be a pivotal control element of the developmental program and skin maturation. Nude mice may be considered as a model of neoteny among mammals. The resemblance of gene expression profiles in the skin of both nude and E14 mice are direct or indirect consequences of the Foxn1 deficiency. Foxn1 appears to regulate the balance between cell proliferation and differentiation and its inactivity creates a pro-regenerative environment.

**Electronic supplementary material:**

The online version of this article (doi:10.1186/s12864-016-3401-z) contains supplementary material, which is available to authorized users.

## Background

The maintenance of skin as a barrier that protects the host from the external environment requires a vigilant state in order to respond immediately to external, often unexpected, insults. Following injury, skin can heal by regenerative (scar-free) or reparative (scar-forming) processes. Regenerative skin healing, so common among lower vertebrates [1–3], is almost absent in adult mammals. In 1979, Rowlatt reported in his seminal paper that human fetuses are capable of regenerative (scar-free) response to skin wounds [4]. Follow-up studies showed that mammalian fetuses heal skin injuries in a scar-free way, if the injury occurs during first two trimesters of gestation. Thereafter, skin lesions heal with scar formation (in the reparative type of healing). The transition from scarless, fetal-type repair to adult-type repair with scarring occurs between days 16.5 and 18.5 of gestation for mice and rats [5] and 24 weeks for humans [6]. Comprehensive studies on regeneration in fetal skin have uncovered important differences between fetal and adult skin and identified factors that are prerequisites for scar-free healing; however, key pathways responsible for regenerative healing remain to be determined [7].

Recently, our laboratory introduced a genetic mouse model to determine the underlying mechanisms of scar-free healing by showing that adult nude (Foxn1-deficient) mice, similar to mammalian fetuses, are able to heal their skin injuries in a scar-free way [8–10]. To explore the possible mechanisms of skin regeneration, mouse models with variable immune-competency were examined to assess whether the immunodeficiency of nude mice involving the lack of thymus or T-lymphocytes could explain scar-free skin healing [8]. Although the results of the studies do not challenge the contribution of inflammatory response in the outcome of skin wound healing, they showed that the lack of thymus and/or T-cells was not a condition for scarless healing [8].

The nude phenotype is the consequence of a spontaneous point mutation in the forkhead box N1 (*Foxn1*) gene which encodes a transcriptional factor expressed in epithelial cells of the thymus, a distinct population of keratinocytes in the epidermis, and hair follicles. The pleiotropic effects of the *Foxn1* mutation are manifested by a lack of thymus, primary T-cell deficiency and hairless skin in mice, rats and humans. Studies on the role of Foxn1 in skin and hair development, although not as extensive as on the thymus, indicate that Foxn1 participates in hair follicle development, promotes keratinocyte differentiation and stimulates signal transduction between melanocytes and keratinocytes participating in the pigmentation process. Developmental studies by Brissette et al. showed that in the skin, Foxn1 expression begins around the mouth and nose area at day 14, then spreads over the entire skin by days 16–17 of embryonic development in mice [11]. This pattern of expression coincides with the transition from scar-free to scar-forming skin healing in mouse fetuses at day 16 of gestation. It is conceivable that a loss-of-function mutation in *Foxn1* in nude mice and the lack of normal Foxn1 expression during the scar-free healing phase in mammalian fetuses provide conditions favorable to regeneration. Moreover, our recent data revealed that Foxn1 can act as a regulator of the skin wound healing process. The experiments performed on Foxn1::Egfp transgenic mice showed that Foxn1-bearing cells participate in the wound healing process through engagement in re-epithelialization and possible involvement in scar formation due to Foxn1 activity during the epithelial-to-mesenchymal transition (EMT) [12]. In the present study using next-generation high-throughput DNA sequencing techniques, we aimed to test the hypothesis that Foxn1 expression in the skin is an essential condition for establishing the adult skin phenotype and that a lack of Foxn1 maintains skin in a neoteny stage (immature stage of development). We predict that Foxn1- null mice (nude mice) are neotenic in the transcriptomic signature of their skin.

## Results and discussion

In the present study, we have used two unique models of true regeneration among mammals, mouse fetuses at the 14^th^ day of embryonic development (E14) and nude mice, and genome-wide analysis of gene expression to uncover the transcriptional signature of scar-free skin healing. We hypothesize that uninjured skin of E14 fetuses and nude mice share a transcriptomic signature that predisposes them to regenerative skin healing when wounding occurs. In order to establish gene expression profiles in nude and E14 mice, we performed RNA-seq analysis of uninjured skin and epidermis from 6-week old C57BL/6J (B6) and B6.Cg-Foxn1 nu (nude), and skin from B6 fetuses at E14 and E18 day of embryonic development. To exclude differences that can arise from different genetic backgrounds, we used mice on C57BL/6J genetic background. Previous microarray studies, including a comparison of gene expression between cultured dermal fibroblasts isolated from E16.5 and E18.5 fetuses and cultured keratinocytes transfected with *Foxn1*, provided important insight into regenerative response genes [13, 14]. However, the obtained results are highly influenced by culture conditions and the manipulation of *Foxn1* transfection [13, 14]. In contrast to previous studies, we performed the analysis on intact skin to avoid in vitro culture artifacts. We are aware that our analyses are complicated due to the mixture of cellular skin components, such as keratinocytes, melanocytes, and Langerhans cells in the epidermis and dermal fibroblasts; endothelial cells; preadipocytes/adipocytes; and inflammatory cells in the dermis, among others. Nevertheless, wound healing is undoubtedly a process that involves multiple cell types interacting.

### Differential gene expression in skin and epidermis among: nude, B6, E14 and E18 mice

**Fig. 1:**
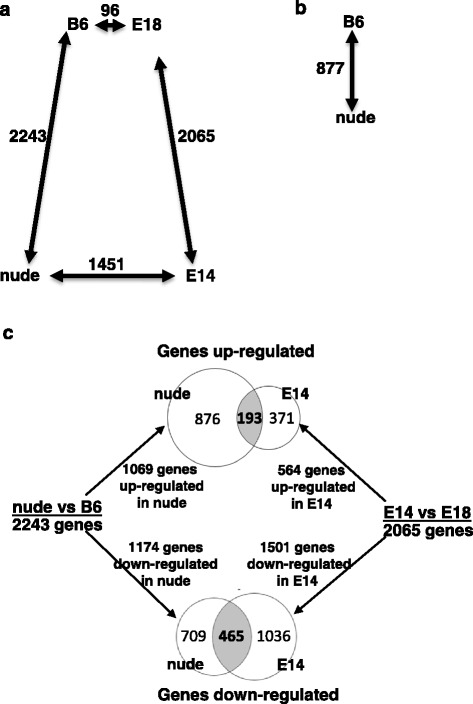
Comparison of the numbers of genes differentially regulated in skin (**a**) and epidermis (**b**). Genes that are up- and down-regulated in common for nude and E14 (**c**). B6 (C57BL/6J; 6 weeks old), nude (B6.Cg-Foxn1 nu; 6 weeks old), E14 (C57BL/6J; 14 day of embryonic development) and E18 (C57BL/6J; 18 day of embryonic development). Numbers next to arrows specify the number of genes that are differentially expressed (using a cutoff threshold of 2-fold difference). Length of arrows indicates the relative abundance of differently regulated genes between groups (**a**) and (**b**). The analysis was performed as an overlay of gene lists from two tested sets of genes: nude vs B6 and E14 vs E18 (**c**)

In the first step of analysis, we compared the transcriptomes from the skin of nude mice (regenerative) to transcriptomes from both adult B6 (reparative) and E14 (regenerative) mice. Subsequently, the transcriptomes from E18 (reparative) skin samples were compared with transcriptomes from both E14 (regenerative) and adult B6 (reparative) skin (Fig. 1a).

In this manner, we generated lists of genes showing statistically significant differences in expression (http://www.ncbi.nlm.nih.gov/geo/query/acc.cgi?acc=GSE71619).

For further analysis we used genes considered as a differentially expressed when changes in the expression were greater than or equal to two-fold. The comparison of skin gene expression between nude (model of skin regeneration) and B6 (model of skin reparation) showed 2243 genes that were variably expressed (up and down) (Fig. 1a). Similarly, 2065 genes were differentially regulated (up and down) in the comparison between the skin from E14 (regenerative) and E18 (reparative) fetuses (Fig. 1a). These numbers clearly demonstrate huge differences in gene expression between regenerative (nude and E14) vs reparative (B6 and E18) models of healing. In contrast to these data, the diversity in the number of differentially expressed genes between mice displaying similar characteristics of healing is smaller. The expression profile of E18 fetuses shows much higher similarity to the profile of adult 6 week-old B6 mice; as only 96 genes were found to be differentially expressed (Fig. 1a). Interestingly, the number of differentially expressed genes in adult nude vs E14 fetuses (1451) is considerably lower than the number of genes that differed between adult nude (regenerative) vs adult B6 (reparative) (2243) (Fig. 1a).

The nude phenotype results from the inactivity of transcription factor Foxn1 whose expression in the skin is restricted to epidermis. Therefore, we performed further analysis to identify the differentially regulated genes in the epidermis isolated from nude and B6 skin (Fig. 1b). The comparison revealed that 306 genes were down-regulated and 571 genes were up-regulated 2-fold and higher in nude epidermis. Such a substantial number of differentially regulated genes that are potent direct and indirect targets of Foxn1 suggests that this transcription factor broadly influences epidermis at the molecular and consequently physiological levels.

Since a number of differentially regulated genes in the first analysis (for example, nude vs E14, see Fig. 1a) may be partially due to the induction and repression of genes associated with developmental processes for E14, in the next step we sought to identify genes that are similarly regulated for nude and E14 mice (Fig. 1c). For this purpose we compared data from two analyses: nude vs B6 (2243 differently regulated genes) to E14 vs E18 (2065 differently regulated genes). We found that among the up-regulated genes in nude vs B6 (1069 genes) and E14 vs E18 (564 genes), 193 are common for nude and E14 (Fig. 1c; Additional file 1: Table S1.). The analysis showed that among 1174 genes down-regulated for nude vs B6 and 1501 for E14 vs E18, 465 genes were common for nude and E14 (Fig. 1c; Additional file 2: Table S2). The greater number of down-regulated vs up-regulated genes in regenerative skin tissues supports the concept proposed by Mercer et al. that the large number of down-regulated genes suggests that the loss of their activity plays significant roles in induction and maintenance of regeneration [15].

### Functional annotation terms grouped according to their biological meaning enriched in nude and E14 skin and nude epidermis

In the next step of the analysis, we focused on the list of differentially expressed genes shared between nude and E14 mice (Fig. 1c; Additional file 1: Table S1 and Additional file 2: Table S2). Genes associated with tissue remodeling, cytoskeletal rearrangements, wound healing and the immune response showed up-regulation in nude and E14 skin tissues, while those related to differentiation were down-regulated (Table 1). Similar gene categories associated with matched molecular and cellular functions have been suggested to influence the outcome of injury in other models of regeneration: lower vertebrates [16] and the MRL mouse [17].

**Table 1:** Selected genes enriched in differentially expressed transcripts nude and E14 skin and relevant functional categories. For epidermis the analysis relays on the comparison of genes differently regulated between nude and B6

Functional categories and representative genes	Skin				Epidermis	
	E14		nude		nude	
	up (*p*-value)	down (*p*-value)	up (*p*-value)	down (*p*-value)	up (*p*-value)	down (*p*-value)
Tissue remodeling						
Zymogen	× (1.2 × 10^−8^)		× (4.5 × 10^−9^)			
Endopeptidase activity	× (7.1 × 10^−2^)		× (9.3 × 10^−2^)			
Metallopeptidase	× (2.3 × 10^−2^)		× (2.4 × 10^−3^)			
Protease	× (5.0 × 10^−5^)		× (1.5 × 10^−6^)			
*Klk10*, kallikrein related-peptidase 10	10.66		63.36		2.61	
*Klk7,* kallikrein related-peptidase 7	11.14		10.95		2.15	
*Klk8*, kallikrein related-peptidase 8	3.22		4.26			
*Prss27*, protease serine 27	8.68		29.83		5.19	
Cytoskeletal rearrangments						
Keratinization	× (3.8 × 10^−2^)		× (8.9 × 10^−4^)		× (4.9 × 10^−2^)	
Keratin			× (3.2 × 10^−8^)			
Cornified envelope	× (4.6 × 10^−5^)		× (3.9 × 10^−7^)		× (1.6 × 10^−8^)	
Keratinocyte differentiation	× (1.5 × 10^−2^)		× (1.8 × 10^−4^)		× (1.9 × 10^−4^)	
*Krt23*, keratin 23	12.06		7.86			
*Krt73*, keratin 73	8.35		17.89		3.85	
*Krt82*, keratin 82	2.8		3.25		2.2	
*Krt17*, keratin 17	9.14		27.58		2.61	
*Krt16, k*eratin 16	13.3		33.43		3.69	
*Sprr2d, s*mall proline-rich protein	15.66		12.88		4.71	
*Flg2, f*ilaggrin family member 2	21.31					
*Rptn, r*epetin	4.65		34.82		3.85	
*Ivl*, involucrin	14.62		6.9		4.45	
Wound healing and immune response						
Inflammatory response	× (1.8 × 10^−7^)		× (8.3 × 10^−13^)		× (2.8 × 10^−2^)	
Chemokine activity	× (1.7 × 10^−5^)		× (6.2 × 10^−6^)		× (8.6 × 10^−2^)	
Positive regulation of T cell differentiation	× (5.2 × 10^−3^)		× (1.9 × 10^−2^)			
*Fn1*, fibronectin 1	2.49		2.77			
*Il-18, i*nterleukin 18	7.47		5.07			
*Il-1A, i*nterleukin 1 alpha	6.32		7.75			
*Il-1 F6, i*nterleukin-1 epsilon (Il1e)	13.67		12.27		4.34	
*Il-1 F8, i*nterleukin 1 family, member 8	9.67		10.45		4.15	
*Il-5*, interleukin 5	2.5		3.14			
Differentiation						
Differentiation		× (2.3 × 10^−4^)		× (1.6 × 10^−4^)		× (1.5 × 10^−3^)
Developmental protein		× (5.8 × 10^−11^)		× (3.0 × 10^−17^)		× (2.8 × 10^−2^)
*Foxd1, f*orkhead box D1		4.47		4.32		
*Gbx2*, gastrulation brain homeobox 2		18.2		7.65		
*Hoxb3*, homeobox B3. transcript variant 2		4.08		2.41		
*Hoxb5*, homeobox B5		5.83		3.7		
*Hoxc6*, homeobox C6		3.66		9.66		
*Hoxd13*, homeobox D13		6.33		5.39		
*Pax1*, paired box gene 1		8.94		9.47		
*Phox2b*, paired-like homeobox 2b		7.11		9.39		
*Pitx1*, paired-like homeodomain transcription factor 1		6.57		7.34		
*Six1*, sine oculis-related homeobox1 homolog (Drosophila)		4.07		8.29		
*Six2*, six2. complete sequence		15.18		10.96		

Among the most commonly up-regulated genes that were involved in tissue remodeling and extracellular matrix composition in common for nude and E14 are kallikreins and kallikrein-related peptidases including *Klk7*, *Klk8* and *Klk10* and protease serine 27 (*Prss27*) (Table 1). It has been suggested that Klk8 stimulates keratinocyte proliferation or differentiation depending on the stage of wound healing and its up-regulation in nude mouse skin was previously detected [18, 19]. Li et al. observed that Prss27, which is also up-regulated in nude and E14 according to our analysis (Table 1), was found in skin only during epidermal hyperproliferation associated with wound closure [20]. They suggested that Prss27 function is associated with re-epithelialization and terminal differentiation of keratinocytes. It is reported that Prss27 and Foxn1 are localized to the suprabasal layer of the epidermis [20]. Considering that Foxn1 acts as a transcriptional activator and it is responsible for the initial stages of differentiation in keratinocytes, the upregulation of Prss27 in the nude skin seems to be an indirect consequence of Foxn1 deficiency [20].

A number of extracellular factors and stimuli (like injury) alter cell behaviors that are mirrored by changes in keratinocyte cytoskeleton and the architecture of the epidermis. E14 and nude mice exhibit prominent up-regulation of keratin (Krt) genes (*Krt23*, -*73*, -*82*, -*16*, -*17*) in the skin and *Krt6a* and -*6b* in nude epidermis (Table 1, Additional file 3: Table S3). *Krt6*, -*16* and -*17* are biomarkers of keratinocytes in an activated (hyperproliferative) state [21]. Interestingly, elevated levels of *Krt6a*, - *6b* and *-16* were also detected in uninjured digital tips of the MRL mouse, the strain which displays features of enhanced regenerative abilities [22]. Moreover, mouse embryos and post-injured skin of Acomys (regenerative) mice exhibit elevated levels of keratins transcripts (*Krt6, -16, -17*) [23, 24].

As keratinocytes switch from proliferation to differentiation, they begin to synthesize cornified envelope protein precursors [25, 26]. Involucrin (Ivl), filaggrin (Flg2), small proline-rich protein 2d (Sprr2d) and repetin (Rptn) belong to this class of peptides and all are significantly up-regulated in E14 (skin) and nude (skin and epidermis) tissues (Table 1). It was showed that over-expression of Foxn1 results in increased expression of early differentiation markers (Krt1) and reduced expression of late differentiation markers, such as Ivl and Flg2 [27, 28]. It is not surprising that lack of Foxn1 in the nude mouse skin caused higher levels of Ivl and Flg2 expression [14, 29]. High levels of *Ivl* and *Flg2* expression in the nude and E14 skin detected in our study can indicate premature differentiation upon the loss of Foxn1 function. A similar observation of premature differentiation of interfollicular epidermal stem cells was made upon loss of function due to Wnt mutation [30]. These deductions are supported by the data showing that Wnt regulates *Foxn1* expression in thymus and in the skin [31, 32].

Surprisingly, in the uninjured skin (nude and E14) and epidermis (nude), transcriptome analysis revealed up-regulation of genes associated with wound healing and its essential inflammation phase (Table 1). Fibronectin 1 (*Fn1*), which is a component of the provisional matrix of embryos enabling migration and action of cells at the place of injury, is also up-regulated in uninjured nude and E14 skin (Table 1) [33]. Moreover, the skin of nude and E14 samples exhibited a significant elevation in levels of interleukin 1α (*Il-1A*) mRNA, a pro-inflammatory interleukin that triggers keratinocyte activation upon injury. Similarly, *Il-1 F6* and *Il-1 F8*, novel players in the Il-1 family signaling system are characteristic for activated (hyperproliferative) keratinocytes and are up-regulated in uninjured skin of nude and E14 mice (Table 1). It should be noted that these two cytokines were shown to influence tissue remodeling (e.g., via regulation of matrix metalloproteinases (Mmps) and fibronectin expression). Additionally, proinflamatory cytokines *Il-18* and *Il-5* [34, 35], which are normally rapidly translated in response to injury, are already up-regulated in un-injured skin of nude and E14 (Table 1). Slightly up-regulated levels of pro-inflammatory cytokines can be beneficial for regenerative resolving of wound healing process, as cytokines attract and stimulate cells (i.e., dermal fibroblasts) at the place of injury. Moreover, the MRL mouse possess increased populations of inflammatory cells during regenerative ear wound closure [36]. On the contrary, the robust inflammatory response in the adult-type repair is consider to be responsible for scar-forming healing usually associated with fibrosis and enhanced deposition of ECM [37]. Therefore, we suggest that fine-tuned inflammation, together with a proper balance between cell migration, proliferation, differentiation, and ECM deposition define whether the final outcome of injury is a chronic/non-healing ulcer, regenerated tissue, or a usual or hypertrophic scar. On the other hand, nude mice are severely immune-compromised, because non-active Foxn1 leads to thymus agenesis and a deficiency of T-cells [38]. Therefore, up-regulated transcripts of pro-inflammatory molecules and enhanced kallikreins activity suggest that the skin of nude mice, particularly the epidermal layer (Foxn1 host cells) can act as an immune, compensatory organ [39, 40].

It is remarkable that nude and E14 mice displayed elevated expression of genes that have been reported to be induced upon injury in scar healing models. This conclusion compromises ECM (tenascin (*Tnc*); Additional file 3: Table S3), *Fn1* (Table 1), serine proteases (kallikreins, *Prss27*) cytoskeletal molecules (keratins and keratins-associated proteins), and proinflammatory cytokines (Table 1). This predisposition to respond immediately, which is common between regenerative nude and E14 mice, is also partially true for the MRL mouse [22]. Therefore, we assume that E14 and nude mice share a transcriptomic milieu that predisposes them to regenerative skin healing when wounding occurs.

Functionally, Foxn1 is considered to be a transcription factor that activates and stimulates the first stages of keratinocyte differentiation [11]. The analysis of genes in common for nude and E14 revealed down-regulation of a set of genes connected with differentiation and development processes (Table 1). Since nude and E14 do not have an active *Foxn1* gene the list of down-regulated genes may contain potential targets of Foxn1. Among these possible targets are the genes coding transcription factors associated with the homeodomain: Hox genes (*Hoxb3*, *Hoxb5*, *Hoxc6*, *Hoxd13*), extended Hox genes (*Gbx2*), Paired domain (*Pax1*), Paired-like domain (*Phox2b*, *Pitx1*), Six/sine homeobox (*Six1*, *Six2*) and atypical Hox genes (*Pknox1*) (Table 1). These homeodomain-containing transcriptional regulators specify cell fate during embryonic development and promote embryonic stem cells differentiation [41] and they are known to undergo dynamic repression by Polycomb group (PcG) proteins during development [41].

There is a concept that “Cutaneous wound repair recapitulates embryonic skin development in numerous aspects, in an attempt to restore the integrity of the injured tissue” [33]. Our data supports this, suggesting that the transcriptomic profile of uninjured nude skin resembles that of regenerative uninjured fetuses’ skin, that is, a neotenic relationship. Similar retained features of embryonic metabolism were described in adult MRL mice [42]. In our regenerative models, we detected down-regulation of some transcription factors that promote differentiation and block proliferation of epidermal progeny cells (genes repressed by PcG; Table 1). This can lead to an increase in the number of undifferentiated, presumably epidermal stem cells (ESC) [43]. Previously, it was shown that a local population of ESC exerts a positive impact on skin regenerative capacity [44]. Correspondingly, our previous data showed that large populations of nude dermal fibroblasts display stem cell biomarkers compared to wild type B6 dermal fibroblasts [45]. Therefore, we assume that the lack of Foxn1 activity prevents the final stages of differentiation [14] and keeps cells in an immature progenitor-like state [43, 46].

### Genes enriched in nude skin but not shared with E14 fetuses

From the total of 1566 genes that are differently regulated between nude vs B6 and not expressed in the E14 skin (Additional file 3: Table S3 and Additional file 4: Table S4), we selected genes that are possibly related to Foxn1 deficiency and provide a transcriptomic signature for regeneration (Table 2). Among these genes, members of the Wnt, Bmp and Notch families were significantly altered (Table 2). It has been shown that genes belonging to these families are responsible for the maintenance of skin homeostasis by playing a role in regulating cell proliferation and differentiation in the skin wound healing process [30, 47–50]. Moreover, it is thought that they take part in regeneration and scarless skin wound healing [33, 51].

**Table 2:** Selected group of genes characteristic for nude mice

Gene	Gene name	Epidermis		Skin	
		nude vs B6		nude vs B6	
		up	down	up	down
*Wisp2*	WNT1 inducible signaling pathway protein 2			13.46	
*Wnt10a*	Wingless related MMTV integration site 10a			4.64	
*Wnt11*	Wingless-related MMTV integration site 11	3.38		2.48	
*Wnt7a*	Wingless-related MMTV integration site 7A				5.63
*Fzd1*	Frizzled homolog 1 (Drosophila)			2.18	
*Fzd6*	Frizzled homolog 6 (Drosophila)			2.02	
*Frzb*	Frizzled-related protein				5.08
*Sfrp2*	Secreted frizzled-related protein 2				2.38
*Ctnnb1*	Catenin (cadherin associated protein), beta 1	4.9			
*Ctnnbl1*	Catenin, beta like 1				2.26
*Dkk1*	Dickkopf homolog 1 (Xenopus laevis)				8.22
*Dlk1*	Delta-like 1 homolog (Drosophila)				9.01
*Dll4*	Dll-4 mRNA for Delta-4				3.18
*Dll1* ^a^	Delta-like 1 (Drosophila)		2.7		2.33
*Notch2*	Notch2		2.36		
*Notch4*	Notch gene homolog 4 (Drosophila)				2.48
*Hes5*	Hairy and enhancer of split 5 (Drosophila)				10.26
*Hes6* ^a^	Hairy and enhancer of split 6 (Drosophila)				8.41
*Hey1*	Brain cDNA, clone MNCb-2686, similar to Mus musculus hairy/enhancer-of-split related with YRPW motif1				3.39
*Hey2*	HES-related repressor protein 1 HERP1				2.39
*Bmp2*	Bone morphogenetic protein 2		2.4		3.55
*Bmp4*	Bone morphogenetic protein 4		3.23		
*Bmpr1b*	Bone morphogenetic protein receptor, type 1B				2.75
*Smad1*	MAD homolog 1 (Drosophila)				2.56
*Mmp-12*	Matrix metallopeptidase 12	3.89		64.36	
*Mmp-3*	Matrix metallopeptidase 3			21.83	
*Mmp-13*	Matrix metallopeptidase 13			5.46	
*Mmp-8*	Matrix metallopeptidase 8			4.71	
*Timp1*	Tissue inhibitor of metalloproteinase 1			3.58	
*Mmp-9*	Matrix metallopeptidase 9		2.22	3.28	
*Mmp-2*	Matrix metallopeptidase 2	2.21		3.02	

^a^Genes that are down regulated in E14 skin (see Table S2)

The analysis of differentially regulated genes revealed that Wnt family genes are generally up-regulated in nude skin. Elevated gene expression in nude skin was observed for Wnt ligands *Wisp2*, *Wnt10a* and *Wnt11*; Wnt mediator β-catenin (*Ctnnb1*) and Wnt receptors frizzled 1 (*Fzd1*) and frizzled 6 (*Fzd6*). Concurrently, Wnt pathway inhibitors, dickkopf 1 (*Dkk1*), frizzled-related protein (*Frzb*) and secreted frizzled-related protein 2 (*Sfrp2*) were down-regulated. Among the Wnt family genes altered in the nude mouse, the expression of *Ctnnb1* mRNA was up-regulated in the epidermis but not in the skin (Table 2). In contrast, *Wnt7a* and catenin β-like1 (*Ctnnbl1*) were down-regulated in the skin of nude mice as was *Mmp-7*, a component of the Wnt signaling pathway and the target gene for β-catenin in both nude and E14 skin (Additional file 2: Table S2).

In contrast to the genes of the Wnt family, the genes related to Notch and Bmp are down-regulated in the nude skin (Table 2). The Notch group of genes includes the Notch receptors (*Notch2* and *4*), Notch ligands (*Dll1*, *4,* and Dlk*1*) and its transcription factor targets, *Hes* (*5* and *6*) and *Hey* (*1* and *2*), are down-regulated. Similar to Notch, several genes in the Bmp family are down-regulated in the nude skin, including Bmp ligands (*Bmp2* and *Bmp4*), their receptor (*Bmpr1b*), and an intracellular component of the Bmp pathway, *Smad1* (Table 2).

The next group of genes enriched in nude skin but not shared with E14 is matrix metalloproteinases (*Mmps*) together with metalloproteinases tissue inhibitors (*Timps*) (Table 2). The products of these genes are indispensable for skin wound healing process resolution particularly during the final remodeling phase of healing [52] and they are required for the regeneration process [53]. The present analysis confirmed our previous study showing high levels of Mmps in the uninjured and post-injured skin of nude mice [8–10, 45]. It has been proposed that high levels of Mmps (i.e., Mmp-9) during the regeneration process can prevent scar formation [53]. Our analysis revealed that *Mmp-12, -3, -13, -8, -9,* and *-2* together with *Timp1* are up-regulated in the skin tissues of nude mice (Table 2). The analysis of entire skin tissues as well as enzymatically separated epidermal part of the skin allowed us to detect an unusual pattern of *Mmp-9* expression. Whereas *Mmp-9* expression was up-regulated in the skin (similar to other *Mmps*), it was down-regulated in the epidermis (Table 2).

### Changes in genes and protein expression– validation of sequencing data

**Fig. 2:**
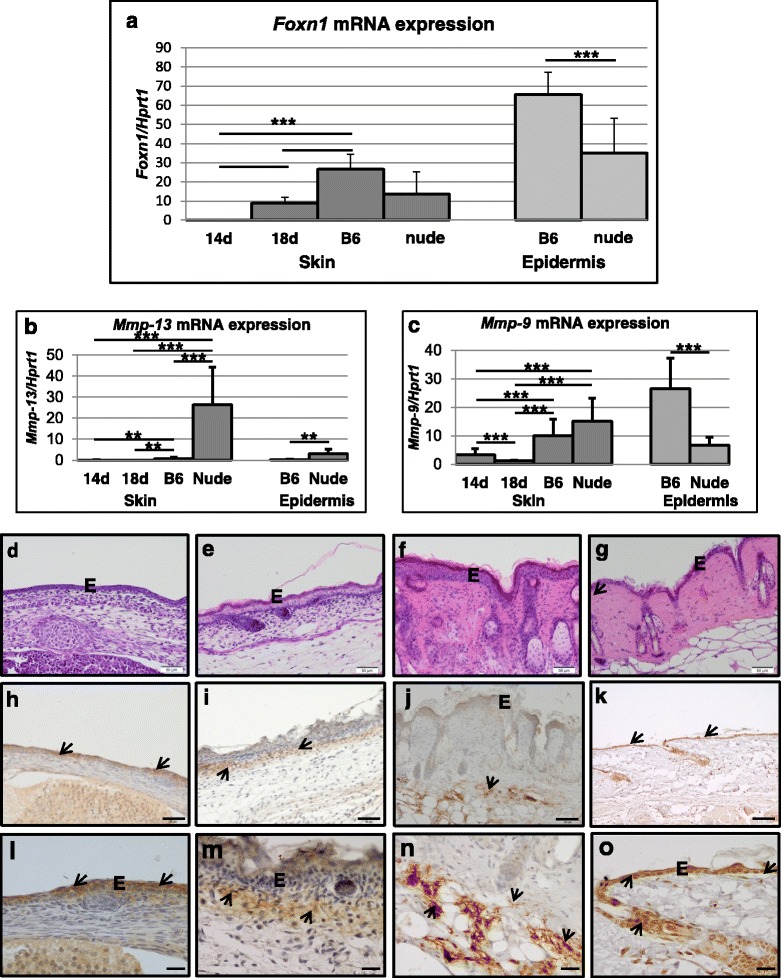
Validation of sequencing data by qRT-PCR for *Foxn1* (**a**), *Mmp-13 *(**b**) and *Mmp-9* (**c**). Histological (**d**-**g**), immunohistochemical (**h**-**o**) analyses of skin tissues from E14 (**d**, **h**, **l**), E18 (**e**, **i**, **m**), nude (**f**, **j**, **n**) and B6 (**g**, **k**, **o**) skin. Hematoxylin and eosin stained sections (**d**-**g**), and immunohistochemical detection of Mmp-9 protein expression (**h**-**o**). Figure l-o are the higher magnifications of figures h-k. Arrows indicate positive reaction for Mmp-9 in epidermis: E14 (**h**, **l**) and B6 (**k**, **o**); dermis: E18 (**i**, **m**) and subcutenous tissues in nude (**j**, **n**). Immunohistochemical sections (**h**-**o**) were counterstained with hematoxylin. E - epidermis. Scale bar = 50 μm (**d**-**j**), 100 μm (**k**), 20 μm (**l**-**o**)

To confirm and validate sequencing data we performed qRT-PCR, Western Blot and immunohistochemical detection experiments using independent biological samples from nude, B6, E14 and E18 skin. First, in order to establish and verify the levels of *Foxn1* mRNA expression in the examined skin tissues, we performed qRT-PCR analysis (Fig. 2a). We detected no *Foxn1* transcripts in E14 skin samples, whereas its expression was detected in E18 and adult skin (Fig. 2a). *Foxn1* mRNA expression was observed not only in B6 but also in Foxn1 deficient nude mice although at lower levels. The highest levels of *Foxn1* transcripts were detected in enzymatically separated epidermis, the host tissue for the Foxn1 expression (Fig. 2a). *Foxn1* mRNA was detected in the nude skin tissue because a single base pair (G) deletion in the exon 3 introduces a frameshift and a premature stop codon causing the encoded protein to terminate upstream of the DNA-binding domain (http://jaxmice.jax.org/strain/000819.html).

**Fig. 3:**
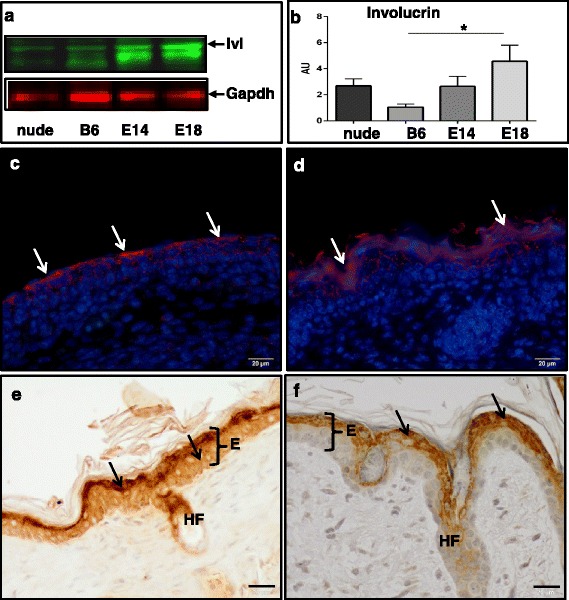
Involucrin protein expression in nude, B6, E14 and E18 skin samples. Representative Western Blots (**a**), densitometric analysis (**b**) and immunodetection (**c**-**f**) of involucrin protein expression in E14 (**c**), E18 (**d**), nude (**e**), B6 (**f**) skin samples. Arrows indicate positive reaction for involucrin (**c**-**f**). Immunohistochemical sections (**e**-**f**) were counterstained with hematoxylin. E – epidermis, HF – hair follicles. Scale bar = 20 μm

One of the characteristic features of nude skin is a robust up-regulation of *Mmp-12*, *-3*, *-13*, *-8*, *-9*, and *-2* (Table 2). qRT-PCR validation (Fig. 2b and c) confirmed that *Mmp-13* and *Mmp-9* are up-regulated in the skin of nude mice as shown by RNA-seq analysis (Table 2). However, RNA-seq results showed a discrepancy in *Mmp-9* expression between the whole skin samples and the enzymatically separated epidermis. In contrast to the skin, the epidermis from the nude skin showed significant down-regulation of *Mmp-9* expression (Table 2). qRT-PCR examination of an independent set of samples from nude and B6 confirmed sequencing RNA-seq results indicating differences between *Mmp-9* expression in epidermis (Fig. 2c; *p* < 0.001). Since the expression and regulation of Mmp-9 seems to be of particular importance in skin we performed immunohistochemical detection of Mmp-9 protein expression (Fig. 2h-o). Whereas the Mmp-9 protein in the nude skin tissue was localized to subcutaneous tissues and dermis with no expression in the epidermis (Fig. 2j and n - arrows), B6 skin showed strong Mmp-9 expression in the epidermis (Fig. 2k and o - arrows). Skin samples from E14 (Fig. 2h and l) and E18 (Fig. 2i and m) show Mmp-9 protein expression in the epidermis (E14) and dermis (E18). The high levels of Mmp-9 expression in nude dermis (Fig. 2c, j and n) could be partially explained by the differences in dermal fibroblasts phenotype between nude and wild type mice [10]. Our previous studies showed the ability of nude dermal fibroblasts to synthesize higher than wild type mRNA and protein levels of Mmp-9 [10]. The transcriptomic data for *Ivl* expression showed an increase in *Ivl* transcript levels in E14 skin and in the nude skin and epidermis (Table 1). Western Blot (Fig. 3a-b) and immunohistology (3c-f) analyses showed higher levels of Ivl protein in E18 than E14 (Fig. 3a-b; compare Fig. 3c and d). Moreover, we observed strong Ivl immunoreactivity in the entire epidermis of nude (Fig. 3e) but only at outer layer of B6 (Fig. 3f) epidermis.

### Foxn1 deficiency in epidermis affects dermal part of the skin

**Fig. 4:**
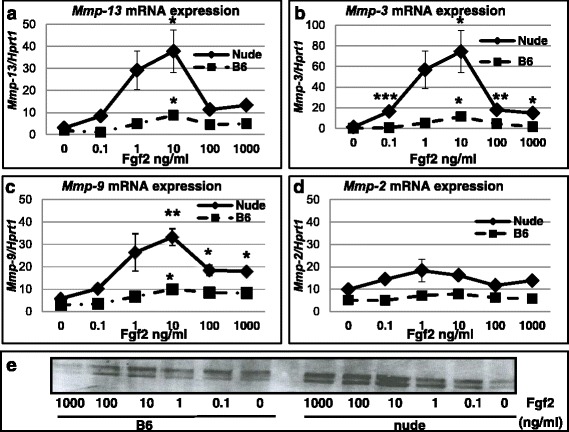
In vitro analysis of Fgf2 effects on Mmp-13 (**a**), Mmp-3 (**b**), Mmp-9 (**c**, **e**) and Mmp-2 (**d**) expression in dermal fibroblasts from nude and B6 mice. Quantitative RT-PCR for *Mmp-13* (**a**), *Mmp-3* (**b**), *Mmp-9* (**c**) and *Mmp-2* (**d**) mRNA expression and Western blot for Mmp-9 (**e**) protein are presented

Although the mechanism of Foxn1 action is largely unknown, recent data point to Fgf2 as a mediator of Foxn1 function in the epidermis [54]. The present analysis showing the differences in gene expression between B6 and nude mice in epidermal and dermal skin parts (Tables 1 and 2, Additional file 1: Table S1, Additional file 2: Table S2, Additional file 3: Table S3, Additional file 4: Table S4) may indicate that Foxn1 elicits its action not only within the epidermis but it participates in dermal cell functionality. Moreover, our previous data clearly showed phenotypic differences between the nude and B6 dermal fibroblasts, indicating a population of immature (stem cell-like) dermal fibroblasts in the nude dermis [45]. Furthermore, the nude skin, as well as the nude dermal fibroblasts, expressed higher levels of Mmps than in B6 mice [10, 45]. Consequently, we reasoned that Foxn1 expression in the epidermis may affect dermal fibroblasts via Fgf2. To evaluate this concept, we measured the effect of Fgf2 on *Mmps* expression in the dermal fibroblasts from nude and B6 mice. Isolated and cultured cells were treated for 24 h with increasing doses of Fgf2 (0.1, 1, 10, 100, 1000 ng/ml; Fig. 4). The analysis of *Mmps* mRNA expression showed that Fgf2 increased *Mmp-3*, *-9* and *-13* expression in both nude and B6 dermal fibroblasts but had no effect on *Mmp-2* expression. Interestingly, the stimulatory effect of Fgf2 was profound for the nude dermal fibroblasts. The increase in *Mmp-13*, *-3* and *-9* expression in nude dermal fibroblasts was already observed at a lower dose (0.1 ng/ml) of Fgf2 to achieve the highest impact on *Mmp* expression at 10 ng/ml (Fig. 4a, b and c). The profound effect of Fgf2 on Mmp-9 expression in the dermal fibroblasts from nude mice was confirmed by Western Blot analysis of protein isolates from nude and B6 dermal fibroblasts (Fig. 4e). Collectively, we propose that B6 dermal fibroblasts achieve the adult/mature state through the first wave of Foxn1 expression during embryonic development, whereas nude dermal fibroblasts, which respond much more strongly to Fgf2 stimulation, are sustained in a fetal (neoteny) stage. These data, together with our previous reports, support the concept that Foxn1 activity is an essential condition to establish the adult skin phenotype. Further on, Foxn1 as a key component of skin maturation, could be responsible for scar-forming healing and its deficiency sustains skin in neoteny, which can lead to scar-free skin healing as it is observed in E14 and nude mice.

## Conclusions

This is the first report showing that two models of skin regeneration, nude mice and E14 fetuses, share similarities that comprise expression patterns associated with genes having specific biological and cellular functions (terms). The genes associated with these functional terms may be considered as a signature for regeneration. Moreover, based on the down-regulation of transcription factors, which promote differentiation instead of proliferation (e.g., during embryo development), we provide a theory that the retarded development of the skin of nude mice leads to the retention of a large population of immature, progenitor-like stem cells. Foxn1 appears to be a pivotal control element of the developmental program and skin maturation. Therefore, nude mice may be considered as a unique example of neoteny among mammals. In addition, nude and E14 mice can be viewed as genetically programmed to immediately respond to injury, since elevated levels of critical transcripts are already in place. The consequences of Foxn1 deficiency suggest an interplay of Foxn1 with Wnt, Bmp and Notch signaling. Moreover, Foxn1 appears to be a regulator of Mmp-9 expression in the skin.

## Methods

### Animals and sample collection

The experimental animal procedures performed in these studies have been approved by the Institutional Animal Care and Use Committee at the Pennington Biomedical Research Center and Institutional Animal Care and Use Committee at the University of Warmia and Mazury.

C57BL/6J (B6) and B6.Cg-Foxn1 nu (nude) mice were purchased from The Jackson Laboratory, ME. Twelve-week-old female and male B6 mice were caged together and the presence of a vaginal plug indicated possible pregnancy and the embryonic age of fetus was designated as gestational day 0.5 (E1). Embryos were collected at embryonic days E14 and E18. Pregnant mice were anesthetized by intraperitoneal injections of ketamine (45 mg/kg) and xylazine (3 mg/kg). An abdominal midline incision was made to expose the uterus. The fetuses were removed from the uterine cavity. Under the dissecting microscopy (10 × magnification, Zeiss, Germany), individual fetuses were immediately killed by decapitation and the full-thickness dorsal skin of fetus was excised with micro-scissors. The skin samples were immediately snap-frozen in liquid nitrogen for subsequent RNA and /or protein isolation or fixed in: 4% paraformaldehyde (PFA) (Sigma-Aldrich, St. Louis, MO, USA) or 10% formalin for histological analyses. The pregnant mice were euthanized after the surgery was completed.

Six-week-old female C57BL/6J (B6, *n* = 10) and B6.Cg-Foxn1 nu (nude, *n* = 10) mice coming directly from The Jackson Laboratory (Bar Harbor, ME, USA) were used to isolate skin tissues from adult animals. Wild type mice were shaved the day before tissue collections. Animals (B6: *n* = 6, nude: *n* = 6) were sacrificed and full-thickness back skin samples were dissected and immediately transferred into liquid nitrogen for subsequent RNA extraction or fixed in: 4% PFA (Sigma-Aldrich) or 10% formalin for histological analysis. Four animals per group (B6 *n* = 4, nude *n* = 4) were used for epidermis isolation. Dissected skin was submerged in HBSS (Sigma-Aldrich) with primocin (InvivoGen, San Diego, CA, USA) followed by overnight incubation in dispase (Life Technologies) solution at 4 °C on a rocking platform. The next day, the epidermis was collected, washed in PBS and transferred to Trizol reagent (Invitrogen, Carlsbad, CA, USA) for RNA isolation.

### Library generation & sequencing

Skin samples of E14, E18, nude and B6 consisted of *n* = 6 per group. Epidermal samples for nude and B6 were equal *n* = 4 per group. Total RNA from each sample was isolated using Trizol reagent as recommended by the supplier. RNA was further purified by using RNeasy and RNase-Free DNase kits (Qiagen, Valencia, CA, USA). RNA integrity was checked by 1% (m/v) agarose gel electrophoresis. The quality of total RNA was analyzed using an Agilent Bioanalyzer and all samples reached a quality score (RIN) above 7. Total RNA samples were prepared for sequencing using the SOLiD™ SAGE Kit with Barcoding Adaptor Module Kit (Life Technologies) using 500 ng input according to the manufacturer’s protocol. Emulsion PCR and SOLiD sequencing, SAGE-sequencing of 35 base pair tags, 30 base pairs in a single direction, were performed according to the manufacturer’s instructions for the SOLiD™ 3 System (Life Technologies). SAGE-seq data does not require the transcript abundance calculation based upon transcript length or identification of isoforms since each transcript is mapped by a single 3′ tag. It is important to emphasize, that SAGE sequencing was performed on individual but not pooled RNA samples (total *n* = 32; including: E14 *n* = 6, E18 *n* = 6, nude *n* = 6, B6 *n* = 6 per skin and nude *n* = 4, B6 = 4 per epidermis).

The number of reads mapping to a unique transcript obtained from the sequencing the DNA fragments were as follow: epidermis B6 18,520,100; epidermis nude 12,689,199; skin E14 26,735,763; skin E18 21,730,454; skin nude 22,873,269 and skin B6 26,406,841.

Erroneous sequence tags were eliminated using quality values before alignment where the quality value ‘q’ corresponds to the failing probability of *p* = 10^(-q/10). Tag counts were included if the quality value of each base call in the tag was greater than or equal to 10.

### Data analysis-SAGE-seq dataset

Sequenced fragments were aligned to the mouse NCBI37/mm9 SAGE reference sequence using SOLiD™ SAGE™v1.10 software (Life Technologies). Raw counts were used as the input for differential expression analysis using GeneSifter, Geospiza, Inc. Libraries were normalized using the geometric mean. Reads with up to 1 mismatch which mapped uniquely to a single transcript were counted. Fold changes of expression at the gene level were calculated. For the preliminary selection, genes met a fold-change in expression of > or =1.5 between experimental groups and a *p*-value or Benjamini-Hochberg adjusted *p*-value of 0.05. The obtained gene data set were deposited in the Gene Expression Omnibus (GEO) repository: GSE71619.

For further analysis we used genes considered as a differentially expressed based on: 1) A greater than or equal to two fold change in expression; 2) The *p* value was less than or equal to 0.05. Functional gene annotation was performed with DAVID bioinformatics platform 6.8 provided by the National Institute of Allergy and Infectious Diseases (https://david.ncifcrf.gov). [55, 56]. The statistical significance of gene set enrichment was determined using the modified Fisher’s exact test. Benjamini-Hochberg correction procedure for multiple comparisons using a *p*-value threshold of <0.05 was carried out for the final selection of ontological terms.

### Real-time PCR and western blot analyses

Sample preparations of total RNA for qRT-PCR and protein isolation for Western Blot analyses were performed as described previously [10] To measure the levels of *Foxn1*, *Mmp-9*, *Mmp-13*, *Hprt1* mRNA expression, single tube TaqMan® Gene Expression Assays (Life Technologies) were used. Each run included a standard curve based on aliquots of RNA pooled from different skin tissue samples. All samples were analyzed in duplicates. mRNA expression levels were normalized to the reference gene - *Hprt1* (hypoxanthine phosphoribosyl transferase 1) and multiplied by 10.

Primary antibodies used in Western Blot analysis were rabbit anti-involucrin (Covance; Princeton, NJ, USA), rabbit anti-Mmp-9 (Millipore, Billerica, MA, USA) and mouse anti-Gapdh (AbCam, Cambridge, UK). As secondary antibodies IRDye800TM and Cy5.5 (Rockland, Limerick, PA, USA) were used. Bands were visualized using the Odyssey imaging system (LI-COR Bioscience) according to the manufacturer’s protocol. Densitometric protein analysis was performed as previously described [12].

### Histology and immunohistochemistry

Formalin fixed, paraffin embedded and sectioned (5 μm) skin tissues were used for histological analysis (H&E staining) and immunohistological detection of Mmp-9 and involucrin expression. For immunohistochemistry skin sections were deparaffinized in xylene (Sigma-Aldrich), treated with 0.75% glycine (Sigma-Aldrich) for 30 min to block free aldehyde groups and with 3% H_2_O_2_ (Sigma-Aldrich) for 5 min to block endogenous peroxidases. Slides were blocked in 10% normal horse serum (Vector Laboratories, Burlingame, CA, USA) for 1 h at RT. Primary antibodies: rabbit anti-involucrin (1:1500, Covance) or rabbit anti-Mmp-9 (1:100, Millipore) were applied (o/n; 4 °C). Antibody binding was detected with the ABC complex (Vectastain ABC kit from Vector Laboratories). Peroxidase activity was revealed using 3,3′-diaminobenzidine tetrahydrochloride (Sigma- Aldrich) as a substrate. Two types of controls were performed: (a) the primary antibody was omitted during the procedure; (b) the primary antibody was substituted with nonspecific immunoglobulin G (IgG) during the immunostaining procedure. Tissue sections were mounted undercoverslips with DPX (Merck; Kenilworth, NJ, USA).

### Immunofluorescence

The immunofluorescence procedure was based on a published protocols [12, 57] with modifications. Skin tissues for immunofluorescent detection of involucrin were fixed for 2 h in 4% paraformaldehyde (Sigma-Aldrich) in 0.1 M phosphate buffer (PB). Fixed tissues were stored in 18% sucrose (Sigma-Aldrich) in PB with 0.01% sodium azide (Sigma-Aldrich). Cryostat sections (8 μm) were blocked in 10% normal horse serum (Vector Laboratories) in 0,1% BSA in PB for 1 h at RT and incubated with rabbit anti-involucrin (1:1500; Covance) antibodies o/n at 4 °C. Tissue sections were subsequently incubated with Alexa Fluor 594 (Life Technologies) for 1 h at RT. Slides were mounted under coverslips using ProLong® Gold Antifade Mountant with DAPI (Life Technologies).

Sections were visualized and photographed with an Olympus microscope (BX43) equipped with an Olympus digital camera (XC50) and analyzed with CellSens Dimension 1 Software (Olympus Soft Imaging Solutions GmbH).

## Additional files

Additional file 1: Table S1. Genes in common for E14 and nude skin that are up-regulated for nude vs B6 and up-regulated for E14 vs E18. (DOCX 34 kb)
Additional file 2: Table S2. Genes in common for E14 and nude skin that are down-regulated for nude vs B6 and down-regulated for E14 vs E18. (DOCX 78 kb)
Additional file 3: Table S3. Genes that are up-regulated in nude (vs B6) skin and epidermis, and not in common with E14 (vs E18). (DOCX 103 kb)
Additional file 4: Table S4. Genes that are down-regulated in nude (vs B6) skin and epidermis, and not in common with E14 (vs E18). (DOCX 101 kb)

## Abbreviations

B6: C57BL/6J
Bmp: Bone morphogenetic proteins
Ctnnb1: β-catenin
Ctnnbl1: Catenin β-like1
Dkk1: Dickkopf 1
E14: Mouse fetuses at 14 day of embryonic development
E18: Mouse fetuses at 18 day of embryonic development
ESC: Epidermal stem cells
Fgf2: Fibroblasts growth factor 2 (basic fibroblasts growth factor)
Flg2: Filaggrin
Fn1: Fibronectin 1
Foxn1: Forkhead box N1
Frzb: Frizzled-related protein
Fzd: Frizzled 1
Il: Interleukin
Ivl: Involucrin
Klk: Kallikrein-related peptidases
Krt: Keratin
Mmp: Matrix metalloproteinases
nude: B6.Cg-Foxn1 nu
PcG: Polycomb group proteins
PFA: Paraformaldehyde
Prss27: Protease serine 27
qRT-PCR: Quantitative reverse transcriptase real-time PCR
Rptn: Repetin
Sfrp2: Secreted frizzled-related protein 2
Sprr2d: Small proline-rich protein
Timp: Tissue inhibitors of matrix metalloproteinases
Wnt: Wingless
