# Supplementary material for: Neotenic phenomenon in gene expression in the skin of Foxn1- deficient (nude) mice - a projection for regenerative skin wound healing
Source: BMC Genomics. 2017 Jan 9;18:56. doi: 10.1186/s12864-016-3401-z (PMC5223329; doi:10.1186/s12864-016-3401-z)
Supplement: Additional file 1: Table S1. — Genes in common for E14 and nude skin that are up-regulated for nude vs B6 and up-regulated for E14 vs E18. (DOCX 34 kb) [file 12864_2016_3401_MOESM1_ESM.docx]

Table S1.

Genes in common for E14 and nude skin that are up-regulated for nude vs B6 and up-regulated for E14 vs E18.

| No. | Gene | Gene name | Up in nude skin | Up in E14 skin |
| --- | --- | --- | --- | --- |
| 1 | *216984* | ecotropic viral integration site 2b | 2.96 | 2.03 |
| 2 | *631101* | PREDICTED: Mus musculus hypothetical LOC631101 (LOC631101), mRNA | 21.28 | 17.52 |
| 3 | *667301* |  | 8.18 | 3.93 |
| 4 | *668804* | tyrosine 3-monooxygenase/tryptophan 5-monooxygenase activation protein, theta polypeptide pseudogene | 2.65 | 2.37 |
| 5 | *100039526* | predicted gene 2290 | 3.48 | 11.31 |
| 6 | *100043996* | similar to Grhl1 protein | 6.37 | 7.35 |
| 7 | *1110018J18Rik* | RIKEN cDNA 1110018J18 gene (1110018J18Rik), mRNA | 2.48 | 2.1 |
| 8 | *1110067D22Rik* | RIKEN cDNA 1110067D22 gene, mRNA (cDNA clone MGC:29165 IMAGE:5036749) | 2.26 | 3.35 |
| 9 | *1300014I06Rik* | RIKEN cDNA 1300014I06 gene (1300014I06Rik), mRNA | 3.43 | 2.8 |
| 10 | *1810005K13Rik* | RIKEN cDNA 1810005K13 gene (1810005K13Rik), mRNA | 8.16 | 5.07 |
| 11 | *2200001I15Rik* | RIKEN cDNA 2200001I15 gene, mRNA (cDNA clone MGC:182265 IMAGE:9056159) | 6.96 | 16.09 |
| 12 | *2200002K05Rik* | RIKEN cDNA 2200002K05 gene (2200002K05Rik), mRNA | 2.21 | 2.25 |
| 13 | *2310007B03Rik* | RIKEN cDNA 2310007B03 gene, mRNA (cDNA clone MGC:107226 IMAGE:30315016) | 7.32 | 6.36 |
| 14 | *2310042E22Rik* | RIKEN cDNA 2310042E22 gene (2310042E22Rik), mRNA | 29.51 | 12.98 |
| 15 | *2610528A11Rik* | PREDICTED: Mus musculus RIKEN cDNA 2610528A11 gene (2610528A11Rik), mRNA | 6.76 | 7.92 |
| 16 | *6330406I15Rik* | RIKEN cDNA 6330406I15 gene, mRNA (cDNA clone IMAGE:4946298) | 4.62 | 4.56 |
| 17 | *Ace2* | Angiotensin I converting enzyme (peptidyl-dipeptidase A) 2, mRNA (cDNA clone MGC:25940 IMAGE:4236529) | 3.38 | 7.53 |
| 18 | *Ahr* | Strain A/J aryl-hydrocarbon receptor (Ahr) | 2.48 | 2.96 |
| 19 | *Aif1* | Allograft inflammatory factor 1, mRNA (cDNA clone MGC:35939 IMAGE:5346103) | 6.14 | 2.22 |
| 20 | *Ankrd13c* | Ankyrin repeat domain 13c, mRNA (cDNA clone MGC:170619 IMAGE:8862014) | 2.16 | 2.45 |
| 21 | *Aox4* | Aldehyde oxidase 4 (Aox4), mRNA | 2.14 | 2.47 |
| 22 | *Apol8* | Apolipoprotein L 8, mRNA (cDNA clone MGC:156854 IMAGE:40090463) | 2.25 | 2.61 |
| 23 | *Arpc1b* | Actin related protein 2/3 complex, subunit 1B, mRNA (cDNA clone MGC:8155 IMAGE:3589768) | 3.53 | 2.41 |
| 24 | *Aspn* | Asporin, mRNA (cDNA clone MGC:41375 IMAGE:1365428) | 8.66 | 12.24 |
| 25 | *Atg9b* | SONE major form (Nos3as) | 3.54 | 7 |
| 26 | *AU015228* | Expressed sequence AU015228, mRNA (cDNA clone MGC:183817 IMAGE:9087817) | 2.16 | 2.77 |
| 27 | *B230120H23Rik* | RIKEN cDNA B230120H23 gene, mRNA (cDNA clone MGC:35825 IMAGE:5346593) | 2.68 | 2.44 |
| 28 | *Bag1* | BCL2-associated athanogene 1, mRNA (cDNA clone MGC:5757 IMAGE:3495306) | 2.32 | 2.22 |
| 29 | *BC028528* | CDNA sequence BC028528 (BC028528), mRNA | 2.82 | 2.52 |
| 30 | *Bspry* | B-box and SPRY domain containing (Bspry), mRNA | 5.78 | 4.18 |
| 31 | *C130090K23Rik* | RIKEN cDNA C130090K23 gene, mRNA (cDNA clone MGC:27925 IMAGE:3584006) | 4.68 | 4.05 |
| 32 | *Calml3* | Calmodulin-like 3, mRNA (cDNA clone MGC:6356 IMAGE:3493620) | 4.86 | 12.46 |
| 33 | *Camk1d* | Calcium/calmodulin-dependent protein kinase ID, mRNA (cDNA clone IMAGE:5052777) | 2.14 | 3.39 |
| 34 | *Car12* | Carbonic anyhydrase 12, mRNA (cDNA clone IMAGE:4948680) | 3.47 | 3.91 |
| 35 | *Casp14* | Caspase 14 (Casp14), mRNA | 13.4 | 6.08 |
| 36 | *Cast* | Calpastatin, mRNA (cDNA clone MGC:12116 IMAGE:3710078) | 4.07 | 2.35 |
| 37 | *Cd44* | CD44 antigen, mRNA (cDNA clone IMAGE:3153835) | 2.87 | 4.3 |
| 38 | *Cd53* | CD53 antigen, mRNA (cDNA clone MGC:29192 IMAGE:5010267) | 6.46 | 3.21 |
| 39 | *Ceacam19* | Carcinoembryonic antigen-related cell adhesion molecule 19, mRNA (cDNA clone MGC:130130 IMAGE:40051723) | 5.92 | 6.32 |
| 40 | *Cgref1* | Cell growth regulator with EF hand domain 1, mRNA (cDNA clone MGC:28551 IMAGE:4206019) | 2.38 | 2.62 |
| 41 | *Chmp4c* | RIKEN cDNA 2310010I16 gene, mRNA (cDNA clone IMAGE:3603631) | 3.34 | 7.87 |
| 42 | *Clec11a* | C-type lectin domain family 11, member a, mRNA (cDNA clone MGC:5762 IMAGE:3484887) | 4.14 | 3.09 |
| 43 | *Cln8* | Ceroid-lipofuscinosis, neuronal 8, mRNA (cDNA clone MGC:13730 IMAGE:4188527) | 2.13 | 2.53 |
| 44 | *Col19a1* | Collagen, type XIX, alpha 1 (Col19a1), mRNA | 2.57 | 2.12 |
| 45 | *Col1a1* | Collagen, type I, alpha 1, mRNA (cDNA clone IMAGE:3586143) | 3.57 | 6.26 |
| 46 | *Cpa4* | Carboxypeptidase A4, mRNA (cDNA clone MGC:74350 IMAGE:30249166) | 4.98 | 7.84 |
| 47 | *Cpeb2* | Cytoplasmic polyadenylation element binding protein 2, mRNA (cDNA clone MGC:130319 IMAGE:40055458) | 4.58 | 4.98 |
| 48 | *Csgalnact1* | Chondroitin sulfate N-acetylgalactosaminyltransferase 1, mRNA (cDNA clone MGC:99961 IMAGE:30470205) | 3.56 | 2.72 |
| 49 | *Ctla2a* | Cytotoxic T lymphocyte-associated protein 2 alpha, mRNA (cDNA clone MGC:41186 IMAGE:1244311) | 4.34 | 5.67 |
| 50 | *Ctsc* | Cathepsin C (Ctsc), mRNA | 5.31 | 3.13 |
| 51 | *Dcn* | Decorin (Dcn), mRNA | 3.43 | 5.03 |
| 52 | *Dct* | Dopachrome tautomerase (Dct), mRNA | 7.14 | 7.15 |
| 53 | *Defb1* | Defensin beta 1, mRNA (cDNA clone MGC:36107 IMAGE:4975536) | 16.63 | 7.36 |
| 54 | *Dnase1l2* | Deoxyribonuclease 1-like 2 (Dnase1l2), mRNA | 5.51 | 7.09 |
| 55 | *Dpt* | Dermatopontin (Dpt), mRNA | 7.68 | 16.58 |
| 56 | *Dsc1* | Desmocollin 1 (Dsc1), mRNA | 3.53 | 4.85 |
| 57 | *Edn1* | Endothelin 1, mRNA (cDNA clone MGC:36081 IMAGE:5059522) | 5.15 | 2.1 |
| 58 | *EG432982* | Predicted gene, EG432982, mRNA (cDNA clone MGC:182505 IMAGE:9056399) | 3.2 | 2.17 |
| 59 | *Egln3* | EGL nine homolog 3 (C. elegans), mRNA (cDNA clone MGC:36685 IMAGE:5371854) | 4.18 | 2.39 |
| 60 | *Ehf* | Ets homologous factor, mRNA (cDNA clone IMAGE:3660677) | 9.96 | 2.94 |
| 61 | *Ell2* | Elongation factor RNA polymerase II 2 (Ell2), mRNA | 2.46 | 2.78 |
| 62 | *Elovl7* | ELOVL family member 7, elongation of long chain fatty acids (yeast) (Elovl7), mRNA | 7.94 | 10.23 |
| 63 | *ENSMUSG00000044330* | Predicted gene, ENSMUSG00000044330 (ENSMUSG00000044330), mRNA | 2.14 | 2.25 |
| 64 | *ENSMUSG00000052825* | PREDICTED: Mus musculus similar to Eif2s2 protein (LOC100040220), mRNA | 2.09 | 2.35 |
| 65 | *Ero1l* | ERO1-like (S. cerevisiae), mRNA (cDNA clone MGC:35586 IMAGE:2654098) | 6.88 | 2.49 |
| 66 | *F13a1* | Coagulation factor XIII, A1 subunit (F13a1), mRNA | 3.44 | 4.22 |
| 67 | *Fam162a* | Family with sequence similarity 162, member A (Fam162a), mRNA | 2.38 | 3.21 |
| 68 | *Fbln5* | Fibulin 5, mRNA (cDNA clone MGC:5656 IMAGE:3482574) | 3.22 | 2.08 |
| 69 | *Fcgr3* | Fc gamma receptor III (Fcgr3) mRNA, Fcgr3-b allele | 4.37 | 2.85 |
| 70 | *Fn1* | Fibronectin 1, mRNA (cDNA clone IMAGE:4985138) | 2.77 | 2.49 |
| 71 | *Fos* | FBJ osteosarcoma oncogene, mRNA (cDNA clone MGC:36690 IMAGE:2582234) | 5.68 | 4.71 |
| 72 | *Galnt3* | UDP-N-acetyl-alpha-D-galactosamine:polypeptide N-acetylgalactosaminyltransferase 3 (Galnt3), mRNA | 2.24 | 2.53 |
| 73 | *Galnt6* | N-acetylgalactosaminyltransferase T-6 | 3.66 | 5.86 |
| 74 | *Gbp6* | Guanylate binding protein 6 (Gbp6), mRNA | 2.38 | 2.59 |
| 75 | *Glrx* | Glutaredoxin (Glrx), mRNA | 4.44 | 3.5 |
| 76 | *Gm2a* | GM2 ganglioside activator protein, mRNA (cDNA clone MGC:5949 IMAGE:3482848) | 3.24 | 4.14 |
| 77 | *Gpr87* | G protein-coupled receptor 87 (Gpr87), mRNA | 5.61 | 9.84 |
| 78 | *Grhl1* | Grainyhead-like 1 (Drosophila), mRNA (cDNA clone MGC:36546 IMAGE:4951512) | 6.34 | 7.29 |
| 79 | *Gsdma* | Gasdermin A (Gsdma), mRNA | 6.88 | 5.58 |
| 80 | *Gsdmc* | Gasdermin C (Gsdmc), mRNA | 8.56 | 7.99 |
| 81 | *Gsdmd* | Gasdermin D (Gsdmd), mRNA | 2.78 | 2.3 |
| 82 | *Guca1a* | Guanylate cyclase activator 1a (retina), mRNA (cDNA clone MGC:25102 IMAGE:4501021) | 3.15 | 2.67 |
| 83 | *Ifi202b* | Interferon activated gene 202B, mRNA (cDNA clone MGC:18552 IMAGE:3990226) | 8.43 | 7.08 |
| 84 | *Il18* | Interleukin 18, mRNA (cDNA clone MGC:36130 IMAGE:4988988) | 5.07 | 7.47 |
| 85 | *Il1a* | Interleukin 1 alpha, mRNA (cDNA clone MGC:5780 IMAGE:3599550) | 7.75 | 6.32 |
| 86 | *Il1f6* | Interleukin-1 epsilon (Il1e) | 12.27 | 13.67 |
| 87 | *Il1f8* | IL-1F8 (IL1F8) | 10.45 | 9.67 |
| 88 | *Il5* | Interleukin 5 (Il5), mRNA | 3.14 | 2.5 |
| 89 | *Insig2* | INSIG-2 membrane protein | 3.87 | 3.27 |
| 90 | *Ivl* | Involucrin (Ivl), mRNA | 6.9 | 14.62 |
| 91 | *Kctd11* | Potassium channel tetramerisation domain containing 11 (Kctd11), mRNA | 3.58 | 4.11 |
| 92 | *Kctd4* | Potassium channel tetramerisation domain containing 4, mRNA (cDNA clone MGC:41021 IMAGE:1332429) | 6.59 | 5 |
| 93 | *Klk10* | Kallikrein related-peptidase 10 (Klk10), mRNA | 63.36 | 10.66 |
| 94 | *Klk7* | Kallikrein related-peptidase 7 (chymotryptic, stratum corneum), mRNA (cDNA clone MGC:38194 IMAGE:5322691) | 10.95 | 11.14 |
| 95 | *Klk8* | NP mRNA for neuropsin | 4.26 | 3.22 |
| 96 | *Krt16* | Keratin 16 (Krt16), mRNA | 33.43 | 13.03 |
| 97 | *Krt17* | Keratin 17 (Krt17), mRNA | 27.58 | 9.14 |
| 98 | *Krt23* | Keratin 23, mRNA (cDNA clone MGC:28496 IMAGE:4187035) | 7.86 | 12.06 |
| 99 | *Krt73* | Keratin 73 (Krt73), mRNA | 17.89 | 8.35 |
| 100 | *Krt82* | Keratin 82 (Krt82), mRNA | 3.25 | 2.8 |
| 101 | *Krtdap* | Keratinocyte differentiation associated protein, mRNA (cDNA clone MGC:179003 IMAGE:9053995) | 9.55 | 10.4 |
| 102 | *Lce1c* | Late cornified envelope 1C (Lce1c), mRNA | 6.25 | 13.22 |
| 103 | *Lce1e* | Late cornified envelope 1E (Lce1e), mRNA | 20.41 | 18.77 |
| 104 | *Lce1f* | Late cornified envelope 1F (Lce1f), mRNA | 22.95 | 15.2 |
| 105 | *Lce1g* | Late cornified envelope 1G (Lce1g), mRNA | 19.27 | 19.77 |
| 106 | *Lce1h* | Late cornified envelope 1H (Lce1h), mRNA | 14.96 | 14.81 |
| 107 | *Lce1i* | Late cornified envelope 1I (Lce1i), mRNA | 9.95 | 14.37 |
| 108 | *Lce3c* | Late cornified envelope 3C (Lce3c), mRNA | 11.86 | 15.32 |
| 109 | *Lcp2* | Lymphocyte cytosolic protein 2, mRNA (cDNA clone MGC:11660 IMAGE:3603181) | 2.16 | 2.8 |
| 110 | *Lhfpl1* | Lipoma HMGIC fusion partner-like 1, mRNA (cDNA clone MGC:58969 IMAGE:6389932) | 5.8 | 2.92 |
| 111 | *Lilrb4* | Leukocyte immunoglobulin-like receptor, subfamily B, member 4, mRNA (cDNA clone MGC:6091 IMAGE:3585404) | 11.08 | 6.69 |
| 112 | *Lpxn* | Leupaxin | 2.63 | 2.47 |
| 113 | *Lrat* | Lecithin-retinol acyltransferase (phosphatidylcholine-retinol-O-acyltransferase) (Lrat), mRNA | 2.95 | 4.26 |
| 114 | *Lrrc15* | Leucine rich repeat containing 15 (Lrrc15), mRNA | 14.58 | 4.88 |
| 115 | *Lum* | Lumican (Lum), mRNA | 3.72 | 5.14 |
| 116 | *Ly96* | ESOP1 (Esop1) | 4.41 | 6.15 |
| 117 | *Lypd6b* | LY6/PLAUR domain containing 6B (Lypd6b), mRNA | 2.47 | 2.46 |
| 118 | *Mafb* | V-maf musculoaponeurotic fibrosarcoma oncogene family, protein B (avian) (Mafb), mRNA | 2.33 | 3.77 |
| 119 | *Map3k8* | Mitogen-activated protein kinase kinase kinase 8 (Map3k8), mRNA | 4.3 | 3.93 |
| 120 | *Mapk13* | Mitogen-activated protein kinase 13 (Mapk13), mRNA | 2.74 | 4.2 |
| 121 | *Metrnl* | Meteorin, glial cell differentiation regulator-like (Metrnl), mRNA | 7.54 | 5.94 |
| 122 | *Mgst2* | CDNA clone IMAGE:9054092 | 4.73 | 4.44 |
| 123 | *Mrc1* | Mannose receptor, C type 1 (Mrc1), mRNA | 4.49 | 5.17 |
| 124 | *Ms4a6c* | MS4A6C protein | 4.64 | 2.93 |
| 125 | *Ms4a6d* | Membrane-spanning 4-domains, subfamily A, member 6D, mRNA (cDNA clone MGC:25778 IMAGE:4016611) | 9.41 | 3.58 |
| 126 | *Mtl5* | Tesmin mRNA, 5 UTR variant 1 | 2.68 | 2.34 |
| 127 | *Myh9* | Myosin, heavy polypeptide 9, non-muscle, mRNA (cDNA clone IMAGE:4913872) | 2.15 | 2.55 |
| 128 | *Mypn* | MKIAA4170 protein | 2.23 | 2.06 |
| 129 | *Nab1* | Ngfi-A binding protein 1, mRNA (cDNA clone MGC:18399 IMAGE:4236565) | 2.07 | 3.12 |
| 130 | *Nfe2l3* | Nuclear factor, erythroid derived 2, like 3, mRNA (cDNA clone MGC:5702 IMAGE:3485182) | 5.71 | 3.77 |
| 131 | *Ocln* | Occludin (Ocln), mRNA | 2.23 | 2 |
| 132 | *Otop2* | Hypothetical protein containing ten transmembrane-spanning domain | 5.82 | 4.14 |
| 133 | *OTTMUSG00000005148* | Predicted gene, OTTMUSG00000005148, mRNA (cDNA clone MGC:182396 IMAGE:9056290) | 5.67 | 7.37 |
| 134 | *Ovol1* | OVO homolog-like 1 (Drosophila), mRNA (cDNA clone MGC:29182 IMAGE:5006712) | 4.23 | 8.45 |
| 135 | *Pdgfrl* | Platelet-derived growth factor receptor-like (Pdgfrl), mRNA | 3.26 | 3.07 |
| 136 | *Pilrb2* | Paired immunoglobin-like type 2 receptor beta 2, mRNA (cDNA clone MGC:182573 IMAGE:9056467) | 4.29 | 7.06 |
| 137 | *Pla2g4e* | CDNA clone IMAGE:9053568 | 11.39 | 6.27 |
| 138 | *Plb1* | Phospholipase B1, mRNA (cDNA clone IMAGE:5373588) | 2.77 | 3.06 |
| 139 | *Plcxd2* | Phosphatidylinositol-specific phospholipase C, X domain containing 2 (Plcxd2), mRNA | 2.32 | 2.1 |
| 140 | *Pof1b* | Premature ovarian failure 1b protein (Pof1b) | 7.92 | 7.63 |
| 141 | *Polr2b* | Polymerase (RNA) II (DNA directed) polypeptide B (Polr2b), mRNA | 4.84 | 2.66 |
| 142 | *Pp11r* | Placental protein 11 related (Pp11r), mRNA | 6.86 | 15.66 |
| 143 | *Prdm1* | PR domain containing 1, with ZNF domain (Prdm1), mRNA | 2.69 | 5.23 |
| 144 | *Prss27* | Protease, serine 27, mRNA (cDNA clone MGC:151388 IMAGE:40126330) | 29.83 | 8.68 |
| 145 | *Psca* | Prostate stem cell antigen (Psca), mRNA | 7.4 | 12.43 |
| 146 | *Qpct* | Glutaminyl-peptide cyclotransferase (glutaminyl cyclase), mRNA (cDNA clone MGC:27858 IMAGE:3491756) | 2.68 | 3.05 |
| 147 | *Rab27b* | RAB27B (Yrcmrab27b) | 2.99 | 4.99 |
| 148 | *Rasgef1b* | Strain C3H GPI-gamma 4 | 2.78 | 2.37 |
| 149 | *Rassf10* | Ras association (RalGDS/AF-6) domain family (N-terminal) member 10, mRNA (cDNA clone IMAGE:4016409) | 2.83 | 2.96 |
| 150 | *Rassf9* | Ras association (RalGDS/AF-6) domain family (N-terminal) member 9 (Rassf9), mRNA | 3.61 | 4.34 |
| 151 | *Rbp2* | Retinol binding protein 2, cellular (Rbp2), mRNA | 20.51 | 12.71 |
| 152 | *Rdh12* | Retinol dehydrogenase 12, mRNA (cDNA clone MGC:27665 IMAGE:4527851) | 6.53 | 4.67 |
| 153 | *Rdh9* | Retinol dehydrogenase 9 (Rdh9), mRNA | 7.66 | 9.09 |
| 154 | *Rgs18* | RGS | 3.15 | 2.77 |
| 155 | *Rnf13* | Ring finger protein 13 (Rnf13), transcript variant 2, mRNA | 2.76 | 2.94 |
| 156 | *Rptn* | Repetin (Rptn), mRNA | 34.82 | 4.65 |
| 157 | *S100a14* | S100 calcium binding protein A14, mRNA (cDNA clone MGC:35763 IMAGE:5151924) | 6.61 | 10.36 |
| 158 | *Scpep1* | Serine carboxypeptidase 1 (Scpep1), mRNA | 3.39 | 3.89 |
| 159 | *Sdr16c6* | Short chain dehydrogenase/reductase family 16C, member 6 (Sdr16c6), mRNA | 10.36 | 7.14 |
| 160 | *Serpinb1a* | EIA (Serpinb1) | 2.46 | 4.94 |
| 161 | *Shb* | Src homology 2 domain-containing transforming protein B, mRNA (cDNA clone IMAGE:5135741) | 2.72 | 2.14 |
| 162 | *Skint3* | Selection and upkeep of intraepithelial T cells 3 (Skint3), transcript variant 1, mRNA | 6.81 | 6.77 |
| 163 | *Skint4* | Strain C57BL/6J skint 4 isoform a precursor (Skint4) | 4.12 | 6.28 |
| 164 | *Sla* | Src-like adaptor, mRNA (cDNA clone MGC:41214 IMAGE:1430090) | 3.63 | 2.1 |
| 165 | *Slc15a2* | Solute carrier family 15 (H+/peptide transporter), member 2, mRNA (cDNA clone IMAGE:3583898) | 3.54 | 4.02 |
| 166 | *Slc30a1* | Solute carrier family 30 (zinc transporter), member 1 (Slc30a1), mRNA | 2.15 | 2.27 |
| 167 | *Slc34a2* | Solute carrier family 34 (sodium phosphate), member 2 (Slc34a2), mRNA | 3.11 | 4.27 |
| 168 | *Slc46a2* | Solute carrier family 46, member 2 (Slc46a2), mRNA | 9.38 | 12.94 |
| 169 | *Slc5a1* | Solute carrier family 5 (sodium/glucose cotransporter), member 1, mRNA (cDNA clone MGC:6369 IMAGE:3497611) | 4.71 | 4.68 |
| 170 | *Slc7a8* | Solute carrier family 7 (cationic amino acid transporter, y+ system), member 8 (Slc7a8), mRNA | 4.12 | 2.2 |
| 171 | *Slurp1* | Secreted Ly6/Plaur domain containing 1 (Slurp1), mRNA | 32.76 | 10.54 |
| 172 | *Sostdc1* | Sclerostin domain containing 1, mRNA (cDNA clone MGC:29422 IMAGE:5064413) | 4.78 | 2.12 |
| 173 | *Sparc* | Secreted acidic cysteine rich glycoprotein, mRNA (cDNA clone MGC:6232 IMAGE:3586402) | 3.41 | 2.95 |
| 174 | *Spon2* | Spondin 2, extracellular matrix protein (Spon2), mRNA | 2.57 | 3.36 |
| 175 | *Sprr2d* | Small proline-rich protein 2D (Sprr2d), mRNA | 12.88 | 15.66 |
| 176 | *Srpx* | Sushi-repeat-containing protein, mRNA (cDNA clone MGC:31012 IMAGE:3993025) | 3.73 | 3.5 |
| 177 | *Tbc1d10c* | MFLJ00332 protein | 2.37 | 2.49 |
| 178 | *Tfpi2* | Tissue factor pathway inhibitor 2, mRNA (cDNA clone MGC:13885 IMAGE:4023742) | 4.68 | 5 |
| 179 | *Thbs2* | Thrombospondin 2, mRNA (cDNA clone IMAGE:3583417) | 2.65 | 3.07 |
| 180 | *Thy1* | Thymus cell antigen 1, theta (Thy1), mRNA | 6.5 | 7.24 |
| 181 | *Tm4sf1* | Transmembrane 4 superfamily member 1 (Tm4sf1), mRNA | 4.11 | 3.79 |
| 182 | *Tmem40* | Transmembrane protein 40 (Tmem40), mRNA | 6.29 | 5.48 |
| 183 | *Tmem62* | Transmembrane protein 62, mRNA (cDNA clone MGC:99427 IMAGE:5708443) | 3.34 | 2.19 |
| 184 | *Tmem95* | PREDICTED: Mus musculus similar to TMEM95 protein (LOC432576), mRNA | 3.65 | 4.29 |
| 185 | *Tmprss11f* | Transmembrane protease, serine 11f, mRNA (cDNA clone MGC:164260 IMAGE:40130906) | 4 | 3.21 |
| 186 | *Tmprss13* | Transmembrane protease, serine 13, mRNA (cDNA clone IMAGE:3490022) | 4.46 | 8.93 |
| 187 | *Tprg* | Transformation related protein 63 regulated (Tprg), mRNA | 5.45 | 13.07 |
| 188 | *Trappc6a* | Trafficking protein particle complex 6A, mRNA (cDNA clone MGC:46781 IMAGE:2811950) | 2.18 | 2.52 |
| 189 | *Trex2* | 3-5 exonuclease TREX2 | 5.32 | 8.1 |
| 190 | *Tyrp1* | Tyrosinase-related protein 1 (Tyrp1), mRNA | 13.51 | 3.84 |
| 191 | *Ubn2* | MKIAA2030 protein | 2.62 | 2.95 |
| 192 | *Yod1* | YOD1 OTU deubiquitinating enzyme 1 homologue (S. cerevisiae), mRNA (cDNA clone MGC:170661 IMAGE:8862056) | 2.28 | 2.43 |
| 193 | *Zfp750* | Zinc finger protein 750, mRNA (cDNA clone MGC:183816 IMAGE:9087816) | 4.74 | 9.54 |
