# Supplementary material for: Neotenic phenomenon in gene expression in the skin of Foxn1- deficient (nude) mice - a projection for regenerative skin wound healing
Source: BMC Genomics. 2017 Jan 9;18:56. doi: 10.1186/s12864-016-3401-z (PMC5223329; doi:10.1186/s12864-016-3401-z)
Supplement: Additional file 2: Table S2. — Genes in common for E14 and nude skin that are down-regulated for nude vs B6 and down-regulated for E14 vs E18. (DOCX 78 kb) [file 12864_2016_3401_MOESM2_ESM.docx]

Table S2.

Genes in common for E14 and nude skin that are down-regulated for nude vs B6 and down-regulated for E14 vs E18.

| No. | Gene | Gene name | Down in nude skin | Down in E14 skin |
| --- | --- | --- | --- | --- |
| 1 | *76976* | signal peptidase complex subunit 3 homolog pseudogene | 2.97 | 2.24 |
| 2 | *93704* | protocadherin gamma subfamily B, 7 | 3.95 | 4.46 |
| 3 | *216964* | transformation related protein 53 inducible protein 13 | 3.13 | 2.69 |
| 4 | *432950* | predicted gene 5471 | 2.57 | 2.61 |
| 5 | *625349* | predicted gene 6579 | 4.02 | 3.92 |
| 6 | *627681* | predicted gene 6779 | 4.02 | 5 |
| 7 | *667679* | predicted gene 8757 | 2.74 | 3.49 |
| 8 | *676691* | predicted gene 9686 | 4.38 | 2.88 |
| 9 | 100039459 | predicted gene 12691 | 2.8 | 3.5 |
| 10 | 100041204 | glyceraldehyde-3-phosphate dehydrogenase pseudogene | 4.58 | 3.33 |
| 11 | 100043080 | predicted gene 14859 | 2.73 | 3.49 |
| 12 | 100043183 |  | 2.84 | 2.59 |
| 13 | 100043384 |  | 7.51 | 7.61 |
| 14 | 100043736 | predicted gene 10762 | 3 | 4.35 |
| 15 | 100048858 | similar to Ribosomal protein S6 kinase, polypeptide 2 | 2.79 | 2.49 |
| 16 | *0610010K14Rik* | RIKEN cDNA 0610010K14 gene (0610010K14Rik), mRNA | 2.22 | 2.97 |
| 17 | *1110051M20Rik* | RIKEN cDNA 1110051M20 gene, mRNA (cDNA clone MGC:113786 IMAGE:6843427) | 2.72 | 2.39 |
| 18 | *1500001A10Rik* | RIKEN cDNA 1500001A10 gene, mRNA (cDNA clone IMAGE:5101000) | 2.96 | 3.53 |
| 19 | *1500010J02Rik* | RIKEN cDNA 1500010J02 gene, mRNA (cDNA clone IMAGE:4241696) | 3.52 | 3.14 |
| 20 | *1500011B03Rik* | PREDICTED: Mus musculus RIKEN cDNA 1500011B03 gene, transcript variant 2 (1500011B03Rik), mRNA | 3.47 | 3.64 |
| 21 | *1700020C11Rik* | RIKEN cDNA 1700020C11 gene (1700020C11Rik), mRNA | 2.34 | 2.18 |
| 22 | *2310001A20Rik* | RIKEN cDNA 2310001A20 gene (2310001A20Rik), mRNA | 2.57 | 2.57 |
| 23 | *2310003H01Rik* | RIKEN cDNA 2310003H01 gene, mRNA (cDNA clone MGC:37540 IMAGE:4986832) | 3.73 | 3.18 |
| 24 | *2510003E04Rik* | RIKEN cDNA 2510003E04 gene, mRNA (cDNA clone IMAGE:4481051) | 5.26 | 3.89 |
| 25 | *2610017I09Rik* | CDNA clone IMAGE:6404937 | 26.02 | 11.69 |
| 26 | *2610110G12Rik* | RIKEN cDNA 2610110G12 gene, mRNA (cDNA clone MGC:25587 IMAGE:4007862) | 8.19 | 8.79 |
| 27 | *2700081O15Rik* | RIKEN cDNA 2700081O15 gene, mRNA (cDNA clone MGC:106440 IMAGE:6825382) | 2.88 | 2.25 |
| 28 | *2810025M15Rik* | RIKEN cDNA 2810025M15 gene, mRNA (cDNA clone MGC:67868 IMAGE:3585495) | 3.41 | 2.33 |
| 29 | *2810046L04Rik* | RIKEN cDNA 2810046L04 gene, mRNA (cDNA clone IMAGE:5321620) | 2.43 | 2.63 |
| 30 | *3010026O09Rik* | RIKEN cDNA 3010026O09 gene, mRNA (cDNA clone MGC:41012 IMAGE:1315017) | 4.43 | 2.71 |
| 31 | *3110006E14Rik* | RIKEN cDNA 3110006E14 gene (3110006E14Rik), mRNA | 7.89 | 7.48 |
| 32 | *5730403B10Rik* | RIKEN cDNA 5730403B10 gene, mRNA (cDNA clone MGC:8188 IMAGE:3590511) | 2.04 | 2.31 |
| 33 | *5730453I16Rik* | RIKEN cDNA 5730453I16 gene, mRNA (cDNA clone MGC:46982 IMAGE:4506179) | 2.31 | 2.09 |
| 34 | *5930434B04Rik* | RIKEN cDNA 5930434B04 gene, mRNA (cDNA clone IMAGE:3484433) | 2.42 | 2.55 |
| 35 | *6330407J23Rik* | RIKEN cDNA 6330407J23 gene (6330407J23Rik), mRNA | 7.61 | 7.79 |
| 36 | *6530401D17Rik* | RIKEN cDNA 2900062L11 gene, mRNA (cDNA clone IMAGE:5365902) | 4.75 | 3.37 |
| 37 | *8430410A17Rik* | RIKEN cDNA 8430410A17 gene, mRNA (cDNA clone MGC:36239 IMAGE:5026919) | 2.48 | 2.75 |
| 38 | *8430423G03Rik* | PREDICTED: Mus musculus RIKEN cDNA 8430423G03 gene (8430423G03Rik), mRNA | 4.11 | 4.07 |
| 39 | *9130011E15Rik* | RIKEN cDNA 9130011E15 gene (9130011E15Rik), mRNA | 3.72 | 2.95 |
| 40 | *Abhd8* | Abhydrolase domain containing 8, mRNA (cDNA clone MGC:35837 IMAGE:5050705) | 3.31 | 3.23 |
| 41 | *Accn2* | Amiloride-sensitive cation channel 2, neuronal, mRNA (cDNA clone IMAGE:5363734) | 6.7 | 4.41 |
| 42 | *Accn4* | Amiloride-sensitive cation channel 4, pituitary (Accn4), mRNA | 6.67 | 6.91 |
| 43 | *Acin1* | Apoptotic chromatin condensation inducer 1 (Acin1), transcript variant 2, mRNA | 4.31 | 5.03 |
| 44 | *Acot11* | Brown fat inducible thioesterase 2 | 3.17 | 2.39 |
| 45 | *Actl6a* | Actin-like 6A, mRNA (cDNA clone MGC:5731 IMAGE:3491205) | 2.89 | 2.66 |
| 46 | *Acy1* | Aminoacylase 1, mRNA (cDNA clone MGC:11593 IMAGE:3964138) | 2.68 | 2.06 |
| 47 | *Adat1* | Adenosine deaminase, tRNA-specific 1, mRNA (cDNA clone MGC:28961 IMAGE:4458235) | 2.3 | 2.83 |
| 48 | *Agpat1* | 1-acylglycerol-3-phosphate O-acyltransferase 1 (lysophosphatidic acid acyltransferase, alpha), mRNA (cDNA clone MGC:5841 IMAG | 3.03 | 2.32 |
| 49 | *Agxt2l2* | Alanine-glyoxylate aminotransferase 2-like 2, mRNA (cDNA clone MGC:37320 IMAGE:4975449) | 2.69 | 2.56 |
| 50 | *AI132487* | Expressed sequence AI132487 (AI132487), mRNA | 2.22 | 2.36 |
| 51 | *AI314976* | Expressed sequence AI314976, mRNA (cDNA clone MGC:31023 IMAGE:3989373) | 2.11 | 2.49 |
| 52 | *AI854517* | PREDICTED: Mus musculus expressed sequence AI854517 (AI854517), mRNA | 11.98 | 9.62 |
| 53 | *Aifm2* | Apoptosis-inducing factor, mitochondrion-associated 2 (Aifm2), nuclear gene encoding mitochondrial protein, transcript varian | 2.8 | 4.99 |
| 54 | *Ak3l1* | Adenylate kinase 3-like 1 (Ak3l1), nuclear gene encoding mitochondrial protein, mRNA | 2.8 | 3.29 |
| 55 | *Akap1* | A kinase (PRKA) anchor protein 1 (Akap1), nuclear gene encoding mitochondrial protein, transcript variant 1, mRNA | 6.04 | 3.29 |
| 56 | *Akr1b3* | Aldo-keto reductase family 1, member B3 (aldose reductase), mRNA (cDNA clone MGC:14038 IMAGE:3708927) | 2.26 | 2.87 |
| 57 | *Akt2* | Thymoma viral proto-oncogene 2, mRNA (cDNA clone MGC:14031 IMAGE:4187425) | 2.57 | 2.47 |
| 58 | *Alg2* | Asparagine-linked glycosylation 2 homolog (yeast, alpha-1,3-mannosyltransferase) (Alg2), mRNA | 4.42 | 4.1 |
| 59 | *Alx1* | ALX homeobox 1, mRNA (cDNA clone MGC:59626 IMAGE:6513129) | 4.9 | 7.24 |
| 60 | *Anapc7* | Anaphase promoting complex subunit 7, mRNA (cDNA clone MGC:7204 IMAGE:3482261) | 2.38 | 2.03 |
| 61 | *Angpt1* | Angiopoietin 1 (Angpt1), mRNA | 2.61 | 2.58 |
| 62 | *Ankrd46* | Ankyrin repeat domain 46, mRNA (cDNA clone IMAGE:5401906) | 2.2 | 2.37 |
| 63 | *Ap2a2* | Adaptor protein complex AP-2, alpha 2 subunit, mRNA (cDNA clone IMAGE:3709070) | 2.42 | 2.09 |
| 64 | *Apbb1* | Amyloid beta (A4) precursor protein-binding, family B, member 1, mRNA (cDNA clone IMAGE:4503589) | 2.15 | 5.42 |
| 65 | *Apc2* | Adenomatosis polyposis coli 2 (Apc2), mRNA | 4.32 | 6.45 |
| 66 | *Apex1* | Apurinic/apyrimidinic endonuclease 1 (Apex1), mRNA | 2.65 | 3.47 |
| 67 | *Aph1a* | Anterior pharynx defective 1a homolog (C. elegans) (Aph1a), transcript variant 1, mRNA | 2.05 | 2.98 |
| 68 | *Aplp1* | Amyloid beta (A4) precursor-like protein 1, mRNA (cDNA clone MGC:25658 IMAGE:4481838) | 5.47 | 10.18 |
| 69 | *Apoa1bp* | Apolipoprotein A-I binding protein (Apoa1bp), mRNA | 2.29 | 2.14 |
| 70 | *Arf5* | ADP-ribosylation factor 5 (Arf5), mRNA | 2.5 | 3.21 |
| 71 | *Arhgef10l* | Rho guanine nucleotide exchange factor (GEF) 10-like, mRNA (cDNA clone IMAGE:4166618) | 2.8 | 3.28 |
| 72 | *Arl6ip6* | ADP-ribosylation factor-like 6 interacting protein 6 (Arl6ip6), mRNA | 2.53 | 2.34 |
| 73 | *Arnt2* | Aryl hydrocarbon receptor nuclear translocator 2 (Arnt2), mRNA | 4.68 | 4.91 |
| 74 | *Arpc1a* | Actin related protein 2/3 complex, subunit 1A, mRNA (cDNA clone MGC:5680 IMAGE:3485409) | 2.12 | 2.39 |
| 75 | *Asb4* | Ankyrin repeat and SOCS box-containing 4 (Asb4), mRNA | 7.42 | 4.75 |
| 76 | *Asb8* | Ankyrin repeat and SOCS box-containing 8 (Asb8), mRNA | 3.62 | 4.81 |
| 77 | *Atg12* | Apg12 | 2.2 | 2.5 |
| 78 | *Atl1* | Atlastin GTPase 1, mRNA (cDNA clone IMAGE:1362466) | 2.93 | 2.62 |
| 79 | *Atp6v0e2* | ATPase, H+ transporting, lysosomal V0 subunit E2 (Atp6v0e2), mRNA | 4.12 | 3.99 |
| 80 | *Atp9b* | ATPase, class II, type 9B, mRNA (cDNA clone IMAGE:3498651) | 3.04 | 2.77 |
| 81 | *Atxn1l* | DNA segment, Chr 8, ERATO Doi 587, expressed, mRNA (cDNA clone IMAGE:3326038) | 2.59 | 2.55 |
| 82 | *AU042671* | Expressed sequence AU042671, mRNA (cDNA clone IMAGE:6810236) | 2.97 | 2.61 |
| 83 | *B9d2* | B9 protein domain 2, mRNA (cDNA clone MGC:41256 IMAGE:1265537) | 2.01 | 2.66 |
| 84 | *Bap1* | Uch-x4 mRNA for ubiquitin C-terminal hydrolase X4 | 2.8 | 2.42 |
| 85 | *Bat1a* | HLA-B-associated transcript 1A, mRNA (cDNA clone MGC:38799 IMAGE:5359825) | 3.58 | 3.43 |
| 86 | *Bat2l* | HLA-B associated transcript 2-like, mRNA (cDNA clone MGC:59386 IMAGE:6329995) | 2.19 | 2.06 |
| 87 | *BC017158* | CDNA sequence BC017158 (BC017158), mRNA | 3.68 | 2.29 |
| 88 | *BC018507* | CDNA sequence BC018507, mRNA (cDNA clone IMAGE:4976833) | 2.63 | 2.41 |
| 89 | *Bcl7a* | B-cell CLL/lymphoma 7A, mRNA (cDNA clone MGC:47407 IMAGE:4501205) | 2.13 | 2.37 |
| 90 | *Bex2* | Brain expressed X-linked 2, mRNA (cDNA clone MGC:41182 IMAGE:5058536) | 11.04 | 7.45 |
| 91 | *Bivm* | Basic, immunoglobulin-like variable motif-containing protein (Bivm) | 3.42 | 2.72 |
| 92 | *Blcap* | Bladder cancer associated protein homolog (human), mRNA (cDNA clone MGC:25535 IMAGE:3597231) | 2.21 | 3.55 |
| 93 | *Bms1* | BMS1 homolog, ribosome assembly protein (yeast), mRNA (cDNA clone IMAGE:4503618) | 2.47 | 2.31 |
| 94 | *Bmyc* | Brain expressed myelocytomatosis oncogene (Bmyc), mRNA | 2.48 | 2.28 |
| 95 | *Brwd2* | Bromodomain and WD repeat domain containing 2, mRNA (cDNA clone MGC:47139 IMAGE:4948388) | 2.27 | 2.17 |
| 96 | *Bsdc1* | BSD domain containing 1, mRNA (cDNA clone IMAGE:5344303) | 2.07 | 2.19 |
| 97 | *Bsn* | Bassoon (Bsn), mRNA | 2.33 | 2.18 |
| 98 | *Btbd2* | BTB (POZ) domain containing 2 (Btbd2), mRNA | 2.13 | 2.29 |
| 99 | *Btbd6* | BTB (POZ) domain containing 6, mRNA (cDNA clone MGC:37682 IMAGE:5059510) | 4.09 | 5.57 |
| 100 | *C030046I01Rik* | RIKEN cDNA C030046I01 gene, mRNA (cDNA clone IMAGE:5029949) | 2.3 | 3.4 |
| 101 | *Cadm4* | Cell adhesion molecule 4 (Cadm4), mRNA | 3.9 | 3.53 |
| 102 | *Calm3* | Calmodulin III (Calm3) mRNA, 3 untranslated region | 2.52 | 2.88 |
| 103 | *Camk2n2* | Calcium/calmodulin-dependent protein kinase II inhibitor 2, mRNA (cDNA clone MGC:182800 IMAGE:9056694) | 3.1 | 3.71 |
| 104 | *Carhsp1* | Calcium regulated heat stable protein 1, mRNA (cDNA clone MGC:19027 IMAGE:4166353) | 2.89 | 3.56 |
| 105 | *Ccdc134* | Mus musculus, clone IMAGE:3500870, mRNA | 4.48 | 2.12 |
| 106 | *Ccdc51* | Coiled-coil domain containing 51 (Ccdc51), mRNA | 2.37 | 3.86 |
| 107 | *Cdc25a* | Cell division cycle 25 homolog A (S. pombe) (Cdc25a), mRNA | 2.47 | 2.84 |
| 108 | *Cdca7* | Cell division cycle associated 7 (Cdca7), mRNA | 4.85 | 4.26 |
| 109 | *Cdh2* | Cadherin 2, mRNA (cDNA clone MGC:36119 IMAGE:5100313) | 6.73 | 4.54 |
| 110 | *Chd4* | Chromodomain helicase DNA binding protein 4, mRNA (cDNA clone IMAGE:3492938) | 2.69 | 3.44 |
| 111 | *Chgb* | Chromogranin B, mRNA (cDNA clone MGC:25417 IMAGE:4511248) | 6.63 | 6.85 |
| 112 | *Chmp7* | CHMP family, member 7 (Chmp7), mRNA | 2.12 | 3.06 |
| 113 | *Chst14* | Carbohydrate (N-acetylgalactosamine 4-0) sulfotransferase 14 (Chst14), mRNA | 4.11 | 2.83 |
| 114 | *Chtf8* | CTF8, chromosome transmission fidelity factor 8 homolog (S. cerevisiae) (Chtf8), mRNA | 4.72 | 6.81 |
| 115 | *Clasp2* | CLIP associating protein 2, mRNA (cDNA clone IMAGE:5366873) | 2.27 | 2.41 |
| 116 | *Clip2* | CAP-GLY domain containing linker protein 2, mRNA (cDNA clone MGC:27892 IMAGE:3498067) | 2.02 | 2.55 |
| 117 | *Cnksr3* | Cnksr family member 3, mRNA (cDNA clone MGC:37681 IMAGE:5059479) | 2.61 | 2.16 |
| 118 | *Cnn2* | Calponin 2, mRNA (cDNA clone MGC:27949 IMAGE:3589158) | 2.12 | 2.63 |
| 119 | *Cntnap2* | Contactin associated protein-like 2, mRNA (cDNA clone IMAGE:4456740) | 4.34 | 7 |
| 120 | *Col2a1* | Collagen, type II, alpha 1, mRNA (cDNA clone IMAGE:4506267) | 8.13 | 10.63 |
| 121 | *Cox5b* | Cytochrome c oxidase, subunit Vb (Cox5b), mRNA | 8.43 | 4.71 |
| 122 | *Crmp1* | Collapsin response mediator protein 1, mRNA (cDNA clone MGC:25290 IMAGE:4505284) | 9.13 | 9.25 |
| 123 | *Cspg5* | Neuroglycan C | 3.54 | 3.44 |
| 124 | *Csrnp2* | Cysteine-serine-rich nuclear protein 2 (Csrnp2), mRNA | 4.29 | 2.93 |
| 125 | *Ctf1* | Cardiotrophin 1, mRNA (cDNA clone MGC:36108 IMAGE:4986446) | 4.02 | 3.54 |
| 126 | *Ctnnbl1* | Catenin, beta like 1, mRNA (cDNA clone IMAGE:3371695) | 2.26 | 3.17 |
| 127 | *Cul9* | Cullin 9, mRNA (cDNA clone IMAGE:4486757) | 2.08 | 2.86 |
| 128 | *Cxxc4* | PREDICTED: Mus musculus hypothetical protein LOC100039199 (LOC100039199), mRNA | 5.45 | 3.04 |
| 129 | *Cyb5r1* | Cytochrome b5 reductase 1 (Cyb5r1), mRNA | 3.93 | 2.15 |
| 130 | *D1Bwg0212e* | DNA segment, Chr 1, Brigham & Womens Genetics 0212 expressed, mRNA (cDNA clone MGC:58820 IMAGE:6773595) | 2.43 | 2.44 |
| 131 | *D430019H16Rik* | PREDICTED: Mus musculus RIKEN cDNA D430019H16 gene (D430019H16Rik), mRNA | 10.46 | 4.98 |
| 132 | *Dab2ip* | Disabled homolog 2 (Drosophila) interacting protein (Dab2ip), transcript variant 2, mRNA | 4.84 | 3.19 |
| 133 | *Dact3* | NAA-3 protein | 2.58 | 2.64 |
| 134 | *Dcc* | Deleted in colorectal carcinoma (Dcc), mRNA | 5.76 | 4.7 |
| 135 | *Dchs1* | CDNA clone IMAGE:40091711 | 6.7 | 2.88 |
| 136 | *Dcx* | Doublecortin (Dcx), transcript variant 4, mRNA | 5.79 | 4.35 |
| 137 | *Ddb1* | Damaged-DNA recognition protein 1 (Ddb1 gene) | 3.99 | 4.77 |
| 138 | *Ddef2* | Development and differentiation enhancing factor 2 (Ddef2), transcript variant 3, mRNA | 2.02 | 2.22 |
| 139 | *Dedd* | Death effector domain-containing, mRNA (cDNA clone MGC:29193 IMAGE:5011149) | 2.33 | 2.37 |
| 140 | *Dem1* | Defects in morphology 1 homolog (S. cerevisiae), mRNA (cDNA clone MGC:32508 IMAGE:5064775) | 3.45 | 3.81 |
| 141 | *Dgcr14* | DiGeorge syndrome critical region gene 14, mRNA (cDNA clone MGC:11442 IMAGE:3708646) | 2.53 | 3.94 |
| 142 | *Dgcr2* | DiGeorge syndrome critical region gene 2, mRNA (cDNA clone IMAGE:2609402) | 3.38 | 2.95 |
| 143 | *Dhx35* | DEAH (Asp-Glu-Ala-His) box polypeptide 35 (Dhx35), mRNA | 2.37 | 2.73 |
| 144 | *Dll1* | Delta-like 1 (Drosophila) (Dll1), mRNA | 2.33 | 2.48 |
| 145 | *Dmwd* | Dystrophia myotonica-containing WD repeat motif, mRNA (cDNA clone MGC:37679 IMAGE:5054380) | 4 | 2.85 |
| 146 | *Dnajb5* | Heat shock protein cognate 40 (Hsc40) | 4.26 | 3.11 |
| 147 | *Dnajc27* | DnaJ (Hsp40) homolog, subfamily C, member 27 (Dnajc27), mRNA | 5.46 | 3.79 |
| 148 | *Dnpep* | Aspartyl aminopeptidase (Dnpep), transcript variant 2, mRNA | 2.89 | 2.56 |
| 149 | *Drg2* | Developmentally regulated GTP binding protein 2 (Drg2), mRNA | 2.09 | 2.86 |
| 150 | *E130308A19Rik* | RIKEN cDNA E130308A19 gene (E130308A19Rik), transcript variant 1, mRNA | 2.45 | 3.24 |
| 151 | *E2f6* | E2F transcription factor 6, mRNA (cDNA clone MGC:46747 IMAGE:5358554) | 2.55 | 2.3 |
| 152 | *Ebf2* | Early B-cell factor 2 (Ebf2), mRNA | 6.87 | 2.81 |
| 153 | *Efr3b* | EFR3 homolog B (S. cerevisiae), mRNA (cDNA clone MGC:182739 IMAGE:9056633) | 6.35 | 3.15 |
| 154 | *Elp4* | Elongation protein 4 homolog (S. cerevisiae) (Elp4), mRNA | 2.32 | 2.8 |
| 155 | *Eml1* | Echinoderm microtubule associated protein like 1, mRNA (cDNA clone IMAGE:4456782) | 3.28 | 2.2 |
| 156 | *Emx2* | Empty spiracles-like protein 2 | 4.23 | 2.96 |
| 157 | *ENSMUSG00000072907* | PREDICTED: Mus musculus similar to Oog1 protein, transcript variant 2 (LOC100039315), mRNA | 4.85 | 5.7 |
| 158 | *Entpd4* | Ectonucleoside triphosphate diphosphohydrolase 4, mRNA (cDNA clone IMAGE:4221257) | 3.67 | 2.33 |
| 159 | *Epb4.2* | Erythrocyte protein band 4.2 (Epb4.2), mRNA | 4.6 | 5.32 |
| 160 | *Ephb4* | Eph receptor B4, mRNA (cDNA clone MGC:11526 IMAGE:3709926) | 3.83 | 2.95 |
| 161 | *Ercc8* | Excision repaiross-complementing rodent repair deficiency, complementation group 8, mRNA (cDNA clone MGC:30748 IMAGE:3985548) | 2.53 | 2.22 |
| 162 | *Etfb* | Electron transferring flavoprotein, beta polypeptide (Etfb), mRNA | 7.21 | 2.09 |
| 163 | *Ets1* | Ets-1 mRNA, partial cds, alternatively spliced | 2.52 | 2.01 |
| 164 | *Fads1* | Leptin-suppressed transcript LST1-like mRNA sequence | 2.16 | 2.47 |
| 165 | *Fam117a* | Family with sequence similarity 117, memberA, mRNA (cDNA clone MGC:66582 IMAGE:6415669) | 4.19 | 2.52 |
| 166 | *Fam125b* | MFLJ00022 protein | 4.25 | 2.98 |
| 167 | *Fam131b* | Family with sequence similarity 131, member B (Fam131b), transcript variant a, mRNA | 4.37 | 3.63 |
| 168 | *Fam178a* | Family with sequence similarity 178, member A, mRNA (cDNA clone MGC:183912 IMAGE:9087912) | 2.95 | 2.03 |
| 169 | *Fam19a5* | Family with sequence similarity 19, member A5 (Fam19a5), mRNA | 4 | 4.56 |
| 170 | *Fam98b* | Family with sequence similarity 98, member B (Fam98b), mRNA | 2.71 | 3.03 |
| 171 | *Fancg* | Fanconi anemia complementation group G (Fancg) | 2.54 | 2.18 |
| 172 | *Fbxo10* | F-box protein 10, mRNA (cDNA clone MGC:107720 IMAGE:6836944) | 2.43 | 2.43 |
| 173 | *Fbxo21* | F-box protein 21 (Fbxo21), mRNA | 4.41 | 3.07 |
| 174 | *Fbxw8* | F-box and WD-40 domain protein 8, mRNA (cDNA clone IMAGE:5102839) | 3.71 | 2.64 |
| 175 | *Fkbp4* | FK506 binding protein 4, mRNA (cDNA clone MGC:6528 IMAGE:2651490) | 3.05 | 3.55 |
| 176 | *Foxd1* | Forkhead box D1 (Foxd1), mRNA | 4.32 | 4.47 |
| 177 | *Foxp4* | Winged-helix repressor FOXP4 | 3.89 | 4.31 |
| 178 | *Foxred1* | FAD-dependent oxidoreductase domain containing 1, mRNA (cDNA clone MGC:38853 IMAGE:5361053) | 2.13 | 2.14 |
| 179 | *Fpgs* | Folylpolyglutamyl synthetase, mRNA (cDNA clone MGC:7296 IMAGE:3485401) | 4.23 | 3.61 |
| 180 | *Fxyd7* | FXYD domain-containing ion transport regulator 7 (Fxyd7), mRNA | 5.35 | 8.6 |
| 181 | *G3bp2* | RNA-binding protein isoform G3BP-2a (G3BP2) | 2.19 | 2.23 |
| 182 | *Gbx2* | Gastrulation brain homeobox 2 (Gbx2), mRNA | 7.65 | 18.2 |
| 183 | *Gdap1* | Ganglioside-induced differentiation-associated-protein 1 (Gdap1), mRNA | 3 | 3.32 |
| 184 | *Gm1943* | Gene model 1943, (NCBI), mRNA (cDNA clone IMAGE:5044781) | 3.43 | 4.01 |
| 185 | *Gm672* | MKIAA0427 protein | 2.7 | 2.49 |
| 186 | *Gmpr* | Guanosine monophosphate reductase (Gmpr), mRNA | 7.89 | 2.06 |
| 187 | *Gnao1* | Guanine nucleotide binding protein, alpha O (Gnao1), transcript variant A, mRNA | 3.86 | 3.78 |
| 188 | *Gng3* | Guanine nucleotide binding protein (G protein), gamma 3, mRNA (cDNA clone MGC:36069 IMAGE:5365311) | 8.4 | 14.95 |
| 189 | *Gnl3l* | Guanine nucleotide binding protein-like 3 (nucleolar)-like, mRNA (cDNA clone MGC:90757 IMAGE:6843880) | 2.72 | 3.63 |
| 190 | *Gpr50* | G-protein-coupled receptor 50 (Gpr50), mRNA | 4.5 | 4.25 |
| 191 | *Gprin1* | G protein-regulated inducer of neurite outgrowth 1, mRNA (cDNA clone IMAGE:4481842) | 3.82 | 3.92 |
| 192 | *Gramd1a* | GRAM domain containing 1A, mRNA (cDNA clone MGC:19375 IMAGE:2646082) | 2.24 | 3.08 |
| 193 | *Grik5* | Glutamate receptor, ionotropic, kainate 5 (gamma 2) (Grik5), mRNA | 3.07 | 3.15 |
| 194 | *Gsbs* | G substrate | 9.09 | 13.06 |
| 195 | *Gsta4* | Glutathione S-transferase, alpha 4, mRNA (cDNA clone MGC:13725 IMAGE:3995378) | 6.43 | 6.36 |
| 196 | *Gtpbp5* | GTP binding protein 5, mRNA (cDNA clone IMAGE:1364804) | 2.45 | 4.37 |
| 197 | *Gys1* | Glycogen synthase 1, muscle, mRNA (cDNA clone MGC:25254 IMAGE:3663506) | 3.62 | 2.05 |
| 198 | *H2afy* | Core histone MacroH2A.1 | 2.91 | 2.48 |
| 199 | *Hba-x* | Hemoglobin X, alpha-like embryonic chain in Hba complex (Hba-x), mRNA | 26.53 | 16.71 |
| 200 | *Hbb-bh1* | Hemoglobin Z, beta-like embryonic chain (Hbb-bh1), mRNA | 16.47 | 8.77 |
| 201 | *Hbb-y* | Hemoglobin Y, beta-like embryonic chain (Hbb-y), mRNA | 36.54 | 17.01 |
| 202 | *Hes6* | Hairy and enhancer of split 6 (Drosophila), mRNA (cDNA clone MGC:18755 IMAGE:4011223) | 8.41 | 7.6 |
| 203 | *Hey1* | Brain cDNA, clone MNCb-2686, similar to Mus musculus hairy/enhancer-of-split related with YRPW motif1(Hey1), mRNA | 3.39 | 3.12 |
| 204 | *Hic1* | Hypermethylated in cancer 1 (Hic1) | 3.74 | 4.32 |
| 205 | *Hif1an* | Hypoxia-inducible factor 1, alpha subunit inhibitor, mRNA (cDNA clone MGC:161165 IMAGE:40140349) | 2.87 | 2.16 |
| 206 | *Hnrnph1* | Heterogeneous nuclear ribonucleoprotein H1 (Hnrnph1), mRNA | 2.58 | 2.54 |
| 207 | *Hnrnpr* | Heterogeneous nuclear ribonucleoprotein R, mRNA (cDNA clone MGC:46961 IMAGE:3500728) | 5.47 | 3.88 |
| 208 | *Hoxb3* | Homeo box B3 (Hoxb3), transcript variant 2, mRNA | 2.41 | 4.08 |
| 209 | *Hoxb5* | Homeo box B5 (Hoxb5), mRNA | 3.7 | 5.83 |
| 210 | *Hoxc6* | Homeo box C6 (Hoxc6), mRNA | 9.66 | 3.66 |
| 211 | *Hoxd13* | Homeo box D13 (Hoxd13), mRNA | 5.39 | 6.33 |
| 212 | *Hsd17b10* | Hydroxysteroid (17-beta) dehydrogenase 10, mRNA (cDNA clone MGC:41111 IMAGE:3376098) | 2.72 | 2.16 |
| 213 | *Hsp90ab1* | Heat shock protein 90 alpha (cytosolic), class B member 1 (Hsp90ab1), mRNA | 2.28 | 2.55 |
| 214 | *Igfbpl1* | Insulin-like growth factor binding protein-like 1 (Igfbpl1), mRNA | 10.38 | 13.25 |
| 215 | *Ikbkap* | Inhibitor of kappa light polypeptide enhancer in B-cells, kinase complex-associated protein, mRNA (cDNA clone IMAGE:5365773) | 2.87 | 2.07 |
| 216 | *Ina* | Internexin neuronal intermediate filament protein, alpha (Ina), mRNA | 13.95 | 16.79 |
| 217 | *Inpp5f* | Inositol polyphosphate-5-phosphatase F, mRNA (cDNA clone MGC:169324 IMAGE:8860719) | 2.77 | 2.36 |
| 218 | *Insm1* | Insulinoma-associated 1 (Insm1), mRNA | 4.71 | 11.24 |
| 219 | *Ints1* | Integrator complex subunit 1, mRNA (cDNA clone IMAGE:3601115) | 2.01 | 2.03 |
| 220 | *Ints3* | Integrator complex subunit 3 (Ints3), transcript variant 1, mRNA | 3.14 | 4.23 |
| 221 | *Ints5* | Integrator complex subunit 5, mRNA (cDNA clone MGC:31173 IMAGE:4191047) | 3.08 | 2.52 |
| 222 | *Ipo13* | Importin 13 (Ipo13), mRNA | 2.36 | 2.21 |
| 223 | *Irak1bp1* | Interleukin-1 receptor-associated kinase 1 binding protein 1 (Irak1bp1), mRNA | 3.33 | 3.08 |
| 224 | *Irf2bp1* | Interferon regulatory factor 2 binding protein 1, mRNA (cDNA clone MGC:37487 IMAGE:4984359) | 4.24 | 4.37 |
| 225 | *Isca2* | Iron-sulfur cluster assembly 2 homolog (S. cerevisiae), mRNA (cDNA clone MGC:31079 IMAGE:4037176) | 2.28 | 2.16 |
| 226 | *Isg20l2* | Interferon stimulated exonuclease gene 20-like 2, mRNA (cDNA clone MGC:90787 IMAGE:5720443) | 3.17 | 2.79 |
| 227 | *Isoc2a* | Isochorismatase domain containing 2a (Isoc2a), nuclear gene encoding mitochondrial protein, mRNA | 3.65 | 3.27 |
| 228 | *Kbtbd7* | Kelch repeat and BTB (POZ) domain containing 7 (Kbtbd7), mRNA | 4.01 | 2.72 |
| 229 | *Kcnd3* | Potassium voltage-gated channel, Shal-related family, member 3 (Kcnd3), transcript variant 2, mRNA | 2.27 | 2.39 |
| 230 | *Kel* | Kell blood group (Kel), mRNA | 5.74 | 4.65 |
| 231 | *Khdrbs3* | KH domain containing, RNA binding, signal transduction associated 3, mRNA (cDNA clone MGC:21726 IMAGE:4500976) | 5.06 | 4.56 |
| 232 | *Kif5c* | Kinesin family member 5C (Kif5c), mRNA | 5.12 | 7.22 |
| 233 | *Klc4* | Kinesin light chain 4, mRNA (cDNA clone MGC:11935 IMAGE:3599898) | 2.61 | 2.11 |
| 234 | *Klhdc10* | Kelch domain containing 10 (Klhdc10), mRNA | 2.35 | 2.09 |
| 235 | *Klhdc2* | Kelch domain containing 2 (Klhdc2), mRNA | 2.79 | 2.18 |
| 236 | *Klhl29* | Kelch-like 29 (Drosophila), mRNA (cDNA clone MGC:169907 IMAGE:8861302) | 2.45 | 2.27 |
| 237 | *L1cam* | L1 cell adhesion molecule (L1cam), mRNA | 2.05 | 2.1 |
| 238 | *Lama1* | Laminin, alpha 1 (Lama1), mRNA | 3.65 | 2.4 |
| 239 | *Ldb1* | LIM domain binding 1, mRNA (cDNA clone MGC:11481 IMAGE:3708169) | 2.66 | 2.89 |
| 240 | *Ldlrad3* | Low density lipoprotein receptor class A domain containing 3, mRNA (cDNA clone MGC:178733 IMAGE:9053725) | 2.26 | 2.65 |
| 241 | *Ldoc1l* | Leucine zipper, down-regulated in cancer 1-like, mRNA (cDNA clone IMAGE:5361398) | 2.99 | 3.26 |
| 242 | *Lrrc4b* | Leucine rich repeat containing 4B (Lrrc4b), mRNA | 2.69 | 2.58 |
| 243 | *Lrrn3* | Leucine rich repeat protein 3, neuronal, mRNA (cDNA clone MGC:67223 IMAGE:5698788) | 3.43 | 2.3 |
| 244 | *Lsm14b* | LSM14 homolog B (SCD6, S. cerevisiae), mRNA (cDNA clone MGC:117552 IMAGE:30933544) | 2.49 | 2.69 |
| 245 | *Lztr1* | Leucine-zipper-like transcriptional regulator, 1, mRNA (cDNA clone MGC:35711 IMAGE:2651456) | 2.58 | 2.19 |
| 246 | *Lzts2* | Leucine zipper, putative tumor suppressor 2 (Lzts2), transcript variant 1, mRNA | 3.1 | 3.56 |
| 247 | *Mab21l1* | Mab-21-like 1 (C. elegans), mRNA (cDNA clone MGC:25223 IMAGE:4526962) | 9.22 | 8.02 |
| 248 | *Magee1* | Melanoma antigen, family E, 1 (Magee1), mRNA | 6.38 | 2.78 |
| 249 | *Map2k5* | Mitogen-activated protein kinase kinase 5, mRNA (cDNA clone IMAGE:3666559) | 2.14 | 2.29 |
| 250 | *Mapk4* | Mitogen-activated protein kinase 4, mRNA (cDNA clone IMAGE:6839524) | 3.18 | 2.25 |
| 251 | *Mapk8ip1* | Protein kinase mitogen-activated 8 interacting protein [Mus musculus], mRNA sequence | 3.74 | 4.44 |
| 252 | *Mapt* | Strain ILS microtubule binding protein tau | 3.05 | 3.95 |
| 253 | *Mbip* | MAP3K12 binding inhibitory protein 1 (Mbip), mRNA | 2.81 | 2.7 |
| 254 | *Mcm5* | Minichromosome maintenance deficient 5, cell division cycle 46 (S. cerevisiae) (Mcm5), mRNA | 3.42 | 3.81 |
| 255 | *Mecr* | Mitochondrial trans-2-enoyl-CoA reductase, mRNA (cDNA clone MGC:6611 IMAGE:3488760) | 2.23 | 2.04 |
| 256 | *Meis2* | Meis homeobox 2, mRNA (cDNA clone MGC:13943 IMAGE:4191098) | 4.52 | 4.11 |
| 257 | *Mgat3* | Mannoside acetylglucosaminyltransferase 3 (Mgat3), mRNA | 3.85 | 7.02 |
| 258 | *Mks1* | Meckel syndrome, type 1, mRNA (cDNA clone MGC:183908 IMAGE:9087908) | 3.06 | 2.06 |
| 259 | *Mllt11* | Myeloid/lymphoid or mixed-lineage leukemia (trithorax homolog, Drosophila); translocated to, 11, mRNA (cDNA clone MGC:35902 I | 5.98 | 4.8 |
| 260 | *Mmp7* | Matrix metallopeptidase 7 (Mmp7), mRNA | 2.68 | 2.67 |
| 261 | *Mn1* | Meningioma 1 (Mn1), mRNA | 2.84 | 2.18 |
| 262 | *Morc2a* | Microrchidia 2A, mRNA (cDNA clone IMAGE:3329423) | 3.02 | 2.37 |
| 263 | *Mpnd* | MPN domain containing (Mpnd), mRNA | 2.95 | 3.56 |
| 264 | *Mpp2* | Membrane protein, palmitoylated 2 (MAGUK p55 subfamily member 2) (Mpp2), mRNA | 3.68 | 3.04 |
| 265 | *Mpp3* | Membrane protein, palmitoylated 3 (MAGUK p55 subfamily member 3) (Mpp3), mRNA | 9.37 | 2.76 |
| 266 | *Mprip* | Expressed sequence AA536749, mRNA (cDNA clone IMAGE:4018524) | 2.56 | 2.23 |
| 267 | *Mrpl28* | Mitochondrial ribosomal protein L28, mRNA (cDNA clone IMAGE:3707759) | 2.1 | 2.65 |
| 268 | *Mrpl46* | Mitochondrial ribosomal protein L46 (Mrpl46), nuclear gene encoding mitochondrial protein, mRNA | 2.1 | 2.15 |
| 269 | *Mtap1b* | Microtubule-associated protein 1B (Mtap1b), mRNA | 3.29 | 2.65 |
| 270 | *Mtpap* | Mitochondrial poly(A) polymerase (Mtpap), nuclear gene encoding mitochondrial protein, mRNA | 2.31 | 2.45 |
| 271 | *Mus81* | MUS81 endonuclease homolog (yeast), mRNA (cDNA clone MGC:36246 IMAGE:5038349) | 3.7 | 2.98 |
| 272 | *Myl9* | Myosin, light polypeptide 9, regulatory (Myl9), mRNA | 3.45 | 2.54 |
| 273 | *Mypop* | Myb-related transcription factor, partner of profilin (Mypop), mRNA | 2.38 | 2.56 |
| 274 | *Myst1* | MYST histone acetyltransferase 1, mRNA (cDNA clone MGC:30369 IMAGE:5136304) | 2.98 | 2.64 |
| 275 | *Myst4* | MYST histone acetyltransferase monocytic leukemia 4 (Myst4), mRNA | 2.09 | 2.23 |
| 276 | *Myt1* | Neural zinc finger protein NZF-2b | 4.51 | 7.08 |
| 277 | *Nap1l2* | Nucleosome assembly protein 1-like 2 (Nap1l2), mRNA | 4.49 | 5.89 |
| 278 | *Narfl* | Nuclear prelamin A recognition factor-like (Narfl), mRNA | 2.13 | 2.38 |
| 279 | *Ncan* | Neurocan (Ncan), mRNA | 6.55 | 6.23 |
| 280 | *Negr1* | Neuronal growth regulator 1, mRNA (cDNA clone MGC:175892 IMAGE:40131308) | 2.34 | 3.12 |
| 281 | *Nfatc4* | Nuclear factor of activated T-cells, cytoplasmic, calcineurin-dependent 4, mRNA (cDNA clone MGC:25538 IMAGE:3670623) | 3.03 | 2.63 |
| 282 | *Nisch* | Imidazoline receptor I-1-like protein | 2.63 | 2.04 |
| 283 | *Nkiras2* | NFKB inhibitor interacting Ras-like protein 2 (Nkiras2), mRNA | 5.35 | 5.96 |
| 284 | *Nphp1* | Nephronophthisis 1 (juvenile) homolog (human) (Nphp1), mRNA | 2.36 | 2.02 |
| 285 | *Nr2f1* | Nuclear receptor subfamily 2, group F, member 1 (Nr2f1), mRNA | 6.8 | 7.07 |
| 286 | *Nrbp1* | Nuclear receptor binding protein 1 (Nrbp1), mRNA | 2.02 | 2.42 |
| 287 | *Nrxn2* | Neurexin II, mRNA (cDNA clone MGC:179067 IMAGE:9054059) | 2.71 | 3.72 |
| 288 | *Ntf3* | Neurotrophin 3 (Ntf3), mRNA | 3.81 | 2.03 |
| 289 | *Nthl1* | Nth (endonuclease III)-like 1 (E.coli) (Nthl1), mRNA | 2.95 | 2.98 |
| 290 | *Nudt14* | Nudix (nucleoside diphosphate linked moiety X)-type motif 14 (Nudt14), mRNA | 2.12 | 2.36 |
| 291 | *Nup107* | Nucleoporin 107, mRNA (cDNA clone IMAGE:3497824) | 2.41 | 2.28 |
| 292 | *Nup133* | Nucleoporin 133, mRNA (cDNA clone MGC:38205 IMAGE:5323157) | 2.8 | 2.3 |
| 293 | *Olfr437* | Olfactory receptor 437 (Olfr437), mRNA | 4.57 | 7.55 |
| 294 | *Pak2* | P21 (CDKN1A)-activated kinase 2, mRNA (cDNA clone IMAGE:4208039) | 2.15 | 2 |
| 295 | *Parp1* | Poly (ADP-ribose) polymerase family, member 1, mRNA (cDNA clone MGC:6498 IMAGE:2648390) | 3.46 | 2.21 |
| 296 | *Parp2* | Poly (ADP-ribose) polymerase family, member 2 (Parp2), mRNA | 3.44 | 3.93 |
| 297 | *Pax1* | Paired box gene 1 (Pax1), mRNA | 9.47 | 8.94 |
| 298 | *Pcbp3* | Poly(rC) binding protein 3 (Pcbp3), mRNA | 4.74 | 5.5 |
| 299 | *Pcbp4* | Poly(rC) binding protein 4, mRNA (cDNA clone MGC:6891 IMAGE:2654291) | 5.74 | 4.43 |
| 300 | *Pcdhga2* | Protocadherin gamma subfamily A, 2 (Pcdhga2), mRNA | 4.03 | 4.45 |
| 301 | *Pcid2* | PCI domain containing 2, mRNA (cDNA clone MGC:143948 IMAGE:40095328) | 2.17 | 2.53 |
| 302 | *Pcnxl3* | Pecanex-like 3 (Drosophila) (Pcnxl3), mRNA | 2.25 | 2.74 |
| 303 | *Pcp4* | Purkinje cell protein 4 (Pcp4), mRNA | 5.1 | 8.12 |
| 304 | *Pde1b* | Phosphodiesterase 1B, Ca2+-calmodulin dependent (Pde1b), mRNA | 3.17 | 4.97 |
| 305 | *Pdk2* | Pyruvate dehydrogenase kinase, isoenzyme 2 (Pdk2), mRNA | 5.93 | 2.69 |
| 306 | *Pdlim7* | PDZ and LIM domain 7 (Pdlim7), transcript variant c, mRNA | 9.36 | 2.59 |
| 307 | *Pdxp* | Pyridoxal (pyridoxine, vitamin B6) phosphatase (Pdxp), mRNA | 9.9 | 7.61 |
| 308 | *Pdzrn4* | PDZ domain containing RING finger 4, mRNA (cDNA clone MGC:183947 IMAGE:9087947) | 6.5 | 2.07 |
| 309 | *Pea15a* | Phosphoprotein enriched in astrocytes 15A, mRNA (cDNA clone MGC:47406 IMAGE:4500957) | 2.99 | 2.9 |
| 310 | *Pes1* | Pescadillo homolog 1, containing BRCT domain (zebrafish), mRNA (cDNA clone MGC:18823 IMAGE:4207164) | 3.18 | 3.14 |
| 311 | *Pex6* | Peroxisomal biogenesis factor 6 (Pex6), mRNA | 2.63 | 2.42 |
| 312 | *Pfkl* | Phosphofructokinase, liver, B-type, mRNA (cDNA clone MGC:28316 IMAGE:4014395) | 2.38 | 2.17 |
| 313 | *Phc2* | Edr2 p36 mRNA for early development regulator 2 p36 | 2.92 | 4.58 |
| 314 | *Phox2b* | Paired-like homeobox 2b (Phox2b), mRNA | 9.39 | 7.11 |
| 315 | *Pim3* | Proviral integration site 3 (Pim3), mRNA | 3.87 | 6.32 |
| 316 | *Pitx1* | Paired-like homeodomain transcription factor 1, mRNA (cDNA clone MGC:13954 IMAGE:4192818) | 7.34 | 6.57 |
| 317 | *Pkdcc* | Pkdcc mRNA for putative protein kinase | 4.11 | 3.96 |
| 318 | *Pknox2* | PREP2 mRNA for TALE homeodomain transcription factor Prep2 | 6.97 | 3.55 |
| 319 | *Plscr3* | Phospholipid scramblase 3, mRNA (cDNA clone MGC:28107 IMAGE:3967872) | 3.27 | 4.21 |
| 320 | *Pnck* | Pregnancy upregulated nonubiquitous Ca2+/calmodulin-dependent kinase Pnck | 4.87 | 3.48 |
| 321 | *Pold1* | Polymerase (DNA directed), delta 1, catalytic subunit, mRNA (cDNA clone MGC:6109 IMAGE:3257174) | 2.35 | 2.04 |
| 322 | *Poll* | Polymerase (DNA directed), lambda, mRNA (cDNA clone MGC:7039 IMAGE:3156259) | 3.14 | 2.7 |
| 323 | *Polr2e* | Polymerase (RNA) II (DNA directed) polypeptide E, mRNA (cDNA clone IMAGE:3156885) | 2.4 | 2.98 |
| 324 | *Polr2h* | Polymerase (RNA) II (DNA directed) polypeptide H (Polr2h), mRNA | 3.29 | 2.41 |
| 325 | *Ppm1e* | Protein phosphatase 1E (PP2C domain containing), mRNA (cDNA clone MGC:170530 IMAGE:8861925) | 3.66 | 3.24 |
| 326 | *Ppm1g* | Protein phosphatase 1G (formerly 2C), magnesium-dependent, gamma isoform, mRNA (cDNA clone MGC:6666 IMAGE:3499081) | 3.92 | 4.42 |
| 327 | *Ppm1l* | Protein phosphatase 1 (formerly 2C)-like, mRNA (cDNA clone MGC:106606 IMAGE:6837066) | 13.37 | 3.85 |
| 328 | *Ppp1r14b* | Protein phosphatase 1, regulatory (inhibitor) subunit 14B (Ppp1r14b), mRNA | 3.34 | 5.04 |
| 329 | *Ppp2r2b* | Protein phosphatase 2A regulatory PR55/B beta.2 splice variant mRNA, complete cds; alternatively spliced | 8.18 | 5.76 |
| 330 | *Ppp2r2d* | Protein phosphatase 2, regulatory subunit B, delta isoform (Ppp2r2d), mRNA | 2.51 | 2.65 |
| 331 | *Ppp2r4* | Protein phosphatase 2A, regulatory subunit B (PR 53) (Ppp2r4), mRNA | 3.46 | 6.09 |
| 332 | *Ppp5c* | Protein phosphatase 5, catalytic subunit, mRNA (cDNA clone MGC:5847 IMAGE:3590322) | 2.26 | 2.28 |
| 333 | *Prkg1* | CGMP-dependent protein kinase type Ib (Prkg1b) | 2.9 | 4.29 |
| 334 | *Prmt6* | Protein arginine N-methyltransferase 6, mRNA (cDNA clone MGC:30554 IMAGE:5067159) | 2.84 | 2.58 |
| 335 | *Prosc* | Proline synthetase co-transcribed (Prosc), transcript variant 1, mRNA | 2.54 | 3.26 |
| 336 | *Prpf3* | PRP3 pre-mRNA processing factor 3 homolog (yeast) (Prpf3), mRNA | 3.64 | 2.6 |
| 337 | *Prps1* | Phosphoribosyl pyrophosphate synthetase 1 (Prps1), mRNA | 2.4 | 2.18 |
| 338 | *Prr12* | Proline rich 12, mRNA (cDNA clone IMAGE:5036822) | 3.89 | 3.44 |
| 339 | *Pskh1* | Protein serine kinase H1, mRNA (cDNA clone MGC:60799 IMAGE:30024345) | 2.17 | 2.11 |
| 340 | *Psmb2* | Proteasome (prosome, macropain) subunit, beta type 2, mRNA (cDNA clone MGC:6558 IMAGE:2811010) | 2.43 | 2.22 |
| 341 | *Psmb3* | Proteasome (prosome, macropain) subunit, beta type 3, mRNA (cDNA clone MGC:5639 IMAGE:3500033) | 2.23 | 2.39 |
| 342 | *Ptbp2* | Polypyrimidine tract binding protein 2, mRNA (cDNA clone MGC:11671 IMAGE:3709255) | 3.72 | 2.62 |
| 343 | *Ptov1* | Prostate tumor over expressed gene 1 (Ptov1), mRNA | 3.52 | 2.47 |
| 344 | *Ptpn5* | Protein tyrosine phosphatase, non-receptor type 5 (Ptpn5), mRNA | 3.29 | 7.26 |
| 345 | *Pvrl2* | Poliovirus receptor-related 2, mRNA (cDNA clone MGC:6320 IMAGE:2812478) | 2.37 | 2.09 |
| 346 | *Pwp2* | PWP2 periodic tryptophan protein homolog (yeast), mRNA (cDNA clone IMAGE:3991539) | 2.51 | 2.48 |
| 347 | *Pycrl* | Pyrroline-5-carboxylate reductase-like, mRNA (cDNA clone MGC:35759 IMAGE:4987954) | 3.15 | 3.08 |
| 348 | *R3hcc1* | R3H domain and coiled-coil containing 1 (R3hcc1), mRNA | 2.4 | 2.99 |
| 349 | *Rab33b* | RAB33B, member of RAS oncogene family (Rab33b), mRNA | 2.6 | 2.32 |
| 350 | *Rab3ip* | RAB3A interacting protein (Rab3ip), mRNA | 2.76 | 2.45 |
| 351 | *Raver1* | Ribonucleoprotein, PTB-binding 1, mRNA (cDNA clone IMAGE:5343087) | 2.66 | 2.58 |
| 352 | *Rbm18* | RNA binding motif protein 18, mRNA (cDNA clone MGC:8172 IMAGE:3590164) | 2.32 | 2.4 |
| 353 | *Rbm4b* | RNA binding motif protein 4B (Rbm4b), mRNA | 2.64 | 2.68 |
| 354 | *Rbpms2* | RNA binding protein with multiple splicing 2 (Rbpms2), mRNA | 4.13 | 2.9 |
| 355 | *Rcan2* | Calcineurin inhibitory protein ZAKI-4 | 2.37 | 2.53 |
| 356 | *Rexo1* | REX1, RNA exonuclease 1 homolog (S. cerevisiae) (Rexo1), transcript variant 1, mRNA | 2.76 | 3.07 |
| 357 | *Rnf139* | Ring finger protein 139, mRNA (cDNA clone MGC:60714 IMAGE:30070643) | 2.88 | 2.57 |
| 358 | *Rpa2* | Replication protein A2, mRNA (cDNA clone MGC:6146 IMAGE:3586727) | 3.49 | 3.61 |
| 359 | *Rpap1* | RNA polymerase II associated protein 1, mRNA (cDNA clone IMAGE:3484208) | 2.16 | 2.13 |
| 360 | *Rpl8* | Ribosomal protein L8 (Rpl8), mRNA | 4.64 | 2.62 |
| 361 | *Rplp1* | Ribosomal protein, large, P1 (Rplp1), mRNA | 3.51 | 2.35 |
| 362 | *Rps6ka2* | Ribosomal protein S6 kinase, polypeptide 2, mRNA (cDNA clone IMAGE:4505898) | 2.55 | 2.49 |
| 363 | *Rtn1* | Reticulon 1 (Rtn1), transcript variant 1, mRNA | 4.72 | 10.09 |
| 364 | *Sbk1* | SH3-binding kinase 1 (Sbk1), mRNA | 3 | 3.81 |
| 365 | *Scg3* | Secretogranin III, mRNA (cDNA clone MGC:36181 IMAGE:5362975) | 8.27 | 26.75 |
| 366 | *Scx* | CDNA pooled tissues:(tissue_type=brain,dev_stage=8-12 days neonate,strain=BALB/c),(tissue_type=testis,dev_stage=adult, strain | 3.89 | 6.21 |
| 367 | *Sdhd* | Succinate dehydrogenase complex, subunit D, integral membrane protein, mRNA (cDNA clone MGC:175620 IMAGE:40131036) | 3.38 | 2 |
| 368 | *Setd1a* | SET domain containing 1A, mRNA (cDNA clone IMAGE:3155990) | 2.1 | 2.61 |
| 369 | *Sf3a2* | Splicing factor 3a, subunit 2 (Sf3a2), mRNA | 2.85 | 2.44 |
| 370 | *Sfrs7* | Splicing factor, arginine/serine-rich 7 (Sfrs7), mRNA | 5.12 | 2.76 |
| 371 | *Sh2b2* | SH2B adaptor protein 2 (Sh2b2), mRNA | 4.9 | 2.64 |
| 372 | *Sh2d3c* | Chat-H | 2.86 | 2.36 |
| 373 | *Shc1* | Shcp52 (Shc) | 2.01 | 2.16 |
| 374 | *Shd* | Src homology 2 domain-containing transforming protein D (Shd), mRNA | 8.71 | 7.56 |
| 375 | *Shisa2* | Shisa homolog 2 (Xenopus laevis) (Shisa2), mRNA | 3.69 | 2.31 |
| 376 | *Shisa4* | Shisa homolog 4 (Xenopus laevis) (Shisa4), mRNA | 4.26 | 3.46 |
| 377 | *Shmt2* | Serine hydroxymethyltransferase 2 (mitochondrial) (Shmt2), nuclear gene encoding mitochondrial protein, mRNA | 2.61 | 2.14 |
| 378 | *Sipa1l2* | Signal-induced proliferation-associated 1 like 2, mRNA (cDNA clone MGC:183593 IMAGE:9087593) | 2.1 | 2.9 |
| 379 | *Six1* | Sine oculis-related homeobox 1 homolog (Drosophila), mRNA (cDNA clone MGC:30484 IMAGE:4188451) | 8.29 | 4.07 |
| 380 | *Six2* | Six2, complete sequence | 10.96 | 15.18 |
| 381 | *Slc17a6* | Solute carrier family 17 (sodium-dependent inorganic phosphate cotransporter), member 6, mRNA (cDNA clone MGC:36456 IMAGE:535 | 4.47 | 8.95 |
| 382 | *Slc25a23* | Solute carrier family 25 (mitochondrial carrier; phosphate carrier), member 23, mRNA (cDNA clone IMAGE:4501494) | 2.13 | 2.65 |
| 383 | *Slc2a3* | Solute carrier family 2 (facilitated glucose transporter), member 3, mRNA (cDNA clone MGC:31655 IMAGE:4527667) | 4.07 | 4.43 |
| 384 | *Slc35a4* | Solute carrier family 35, member A4, mRNA (cDNA clone MGC:8198 IMAGE:3590779) | 3.33 | 2.77 |
| 385 | *Slc44a2* | Solute carrier family 44, member 2 (Slc44a2), mRNA | 3.33 | 4.21 |
| 386 | *Smad3* | MAD homolog 3 (Drosophila) (Smad3), mRNA | 3.06 | 2.08 |
| 387 | *Smarcd1* | SWI/SNF related, matrix associated, actin dependent regulator of chromatin, subfamily d, member 1, mRNA (cDNA clone MGC:30227 | 3.18 | 6.19 |
| 388 | *Smo* | Smoothened homolog (Drosophila), mRNA (cDNA clone IMAGE:5374849) | 2.28 | 2.5 |
| 389 | *Snap47* | Synaptosomal-associated protein, 47, mRNA (cDNA clone MGC:32377 IMAGE:5036036) | 3.12 | 2.93 |
| 390 | *Snrnp40* | Small nuclear ribonucleoprotein 40 (U5) (Snrnp40), mRNA | 3.01 | 3.72 |
| 391 | *Snrnp70* | Small nuclear ribonucleoprotein 70 (U1), mRNA (cDNA clone IMAGE:3486572) | 2 | 2.4 |
| 392 | *Sox2* | SRY-box containing gene 2 (Sox2), mRNA | 6.34 | 5.58 |
| 393 | *Spcs2* | Signal peptidase complex subunit 2 homolog (S. cerevisiae), mRNA (cDNA clone IMAGE:3582190) | 2.37 | 2.97 |
| 394 | *Spin2* | Spindlin family, member 2 (Spin2), mRNA | 2.82 | 3.23 |
| 395 | *Spock2* | Sparc/osteonectin, cwcv and kazal-like domains proteoglycan 2 (Spock2), mRNA | 4.29 | 5.64 |
| 396 | *Ss18l1* | MKIAA0693 protein | 2.15 | 2.25 |
| 397 | *Ssbp3* | Single-stranded DNA-binding protein | 2.17 | 2.65 |
| 398 | *St3gal3* | ST3 beta-galactoside alpha-2,3-sialyltransferase 3, mRNA (cDNA clone MGC:5896 IMAGE:3584323) | 2.2 | 2.25 |
| 399 | *Stmn3* | Stathmin-like 3 (Stmn3), mRNA | 14.31 | 38.97 |
| 400 | *Suclg2* | Succinyl-CoA synthetase GTP-specific beta subunit mRNA, 3UTR | 2.55 | 2.02 |
| 401 | *Suv420h2* | Suppressor of variegation 4-20 homolog 2 (Drosophila) (Suv420h2), transcript variant 1, mRNA | 3.27 | 3.12 |
| 402 | *Synpo* | Putative synaptopodin, (ORF1) | 2.73 | 2.9 |
| 403 | *Syp* | Synaptophysin, mRNA (cDNA clone MGC:25678 IMAGE:4507116) | 4.57 | 8.3 |
| 404 | *Taf11* | TAF11 RNA polymerase II, TATA box binding protein (TBP)-associated factor, mRNA (cDNA clone MGC:6331 IMAGE:3483315) | 2.78 | 3.51 |
| 405 | *Tbc1d1* | TBC1 domain family, member 1, mRNA (cDNA clone IMAGE:3500261) | 2.23 | 2.63 |
| 406 | *Tcp11l1* | T-complex 11 like 1, mRNA (cDNA clone MGC:151303 IMAGE:40126245) | 2.68 | 2.17 |
| 407 | *Tead2* | TEA domain family member 2 (Tead2), mRNA | 10.27 | 5.64 |
| 408 | *Tet3* | CDNA clone IMAGE:40058642 | 2.41 | 2.12 |
| 409 | *Th* | Tyrosine hydroxylase (Th), mRNA | 11.31 | 9.3 |
| 410 | *Thap11* | THAP domain containing 11, mRNA (cDNA clone IMAGE:3487400) | 2.69 | 2.98 |
| 411 | *Thumpd2* | PREDICTED: Mus musculus THUMP domain containing 2, transcript variant 3 (Thumpd2), mRNA | 2.23 | 2.21 |
| 412 | *Tjap1* | Tight junction associated protein 1, mRNA (cDNA clone MGC:28243 IMAGE:3993257) | 4.85 | 3.96 |
| 413 | *Tmem106c* | Transmembrane protein 106C (Tmem106c), mRNA | 3.11 | 2.99 |
| 414 | *Tmem129* | Transmembrane protein 129, mRNA (cDNA clone MGC:27943 IMAGE:3587662) | 5.57 | 4.9 |
| 415 | *Tmem201* | Transmembrane protein 201 (Tmem201), transcript variant 1, mRNA | 3.26 | 2.06 |
| 416 | *Tmem223* | Transmembrane protein 223 (Tmem223), mRNA | 3.4 | 3.22 |
| 417 | *Tmem35* | Transmembrane protein 35 (Tmem35), mRNA | 3.44 | 6.2 |
| 418 | *Tmem98* | Transmembrane protein 98 (Tmem98), mRNA | 3.49 | 3.98 |
| 419 | *Tnfrsf21* | Tumor necrosis factor receptor superfamily, member 21, mRNA (cDNA clone MGC:25901 IMAGE:4220624) | 2.97 | 2.43 |
| 420 | *Traf4* | TNF receptor associated factor 4 (traf4) | 4.59 | 3.14 |
| 421 | *Traf7* | TNF receptor-associated factor 7 (Traf7), mRNA | 4.03 | 4.33 |
| 422 | *Trim32* | Tripartite motif-containing 32, mRNA (cDNA clone MGC:31538 IMAGE:4502346) | 2.61 | 2.53 |
| 423 | *Trim37* | Tripartite motif protein 37, mRNA (cDNA clone IMAGE:5067215) | 2.52 | 2.22 |
| 424 | *Trim44* | Tripartite motif-containing 44 (Trim44), mRNA | 2.6 | 2.04 |
| 425 | *Trp53bp1* | Transformation related protein 53 binding protein 1, mRNA (cDNA clone MGC:30704 IMAGE:3979746) | 2.55 | 2.23 |
| 426 | *Trp53i11* | Trp53 inducible protein 11, mRNA (cDNA clone IMAGE:4984921) | 2.41 | 2.79 |
| 427 | *Tsen15* | TRNA splicing endonuclease 15 homolog (S. cerevisiae), mRNA (cDNA clone MGC:35720 IMAGE:5002659) | 2.52 | 2.43 |
| 428 | *Tspan13* | Tetraspanin 13, mRNA (cDNA clone MGC:18777 IMAGE:4166625) | 3.43 | 2.32 |
| 429 | *Tspan6* | Tetraspanin 6, mRNA (cDNA clone MGC:5801 IMAGE:3590862) | 4.46 | 3.49 |
| 430 | *Tspyl4* | TSPY-like 4, mRNA (cDNA clone MGC:38849 IMAGE:5361000) | 3.99 | 3.49 |
| 431 | *Tubgcp2* | Tubulin, gamma complex associated protein 2, mRNA (cDNA clone MGC:38266 IMAGE:5324981) | 2.79 | 2.36 |
| 432 | *U2af2* | U2 small nuclear ribonucleoprotein auxiliary factor (U2AF) 2 (U2af2), mRNA | 2.93 | 3.44 |
| 433 | *Uba2* | Ubiquitin-like modifier activating enzyme 2 (Uba2), mRNA | 2.61 | 3.08 |
| 434 | *Ubfd1* | Ubiquitin family domain containing 1 (Ubfd1), mRNA | 4.53 | 3.48 |
| 435 | *Uchl1* | Ubiquitin carboxyl-terminal hydrolase PGP9.5 (Uch-L1) | 14.29 | 9.68 |
| 436 | *Unk* | Unkempt homolog (Drosophila), mRNA (cDNA clone IMAGE:3585111) | 4.03 | 2.5 |
| 437 | *Uros* | Uroporphyrinogen III synthase (Uros), mRNA | 2.11 | 2.35 |
| 438 | *Usp22* | MKIAA1063 protein | 2.73 | 2.44 |
| 439 | *Vac14* | Vac14 homolog (S. cerevisiae) (Vac14), mRNA | 2.02 | 3.19 |
| 440 | *Vdac1* | Voltage-dependent anion channel 1 (Vdac1), mRNA | 3.38 | 2.41 |
| 441 | *Vps4a* | Vacuolar protein sorting 4a (yeast) (Vps4a), mRNA | 2.86 | 3.94 |
| 442 | *Wdr18* | WD repeat domain 18, mRNA (cDNA clone IMAGE:1195713) | 2.44 | 3.02 |
| 443 | *Wdr42a* | WD repeat domain 42A (Wdr42a), mRNA | 3.34 | 3.47 |
| 444 | *Wee1* | WEE 1 homolog 1 (S. pombe), mRNA (cDNA clone MGC:11504 IMAGE:3156953) | 3.24 | 2.84 |
| 445 | *Wiz* | Widely-interspaced zinc finger motifs (Wiz), transcript variant 2, mRNA | 3.19 | 4.08 |
| 446 | *Wtip* | WT1-interacting protein (Wtip), mRNA | 3.19 | 2.82 |
| 447 | *Xpo6* | Exportin 6 (Xpo6), mRNA | 2.41 | 2.61 |
| 448 | *Yeats4* | YEATS domain containing 4, mRNA (cDNA clone MGC:28311 IMAGE:4013548) | 3.39 | 3.17 |
| 449 | *Zbtb12* | Zinc finger and BTB domain containing 12 (Zbtb12), mRNA | 2.3 | 2.3 |
| 450 | *Zbtb5* | Transcription factor ZNF-POZ | 2.7 | 2.41 |
| 451 | *Zc3h7b* | Zinc finger CCCH type containing 7B (Zc3h7b), mRNA | 2.49 | 2.34 |
| 452 | *Zc3hc1* | Zinc finger, C3HC type 1, mRNA (cDNA clone IMAGE:3967759) | 2.11 | 2.04 |
| 453 | *Zcchc11* | CDNA clone IMAGE:9054070 | 3.19 | 3.03 |
| 454 | *Zdhhc16* | Zinc finger, DHHC domain containing 16, mRNA (cDNA clone MGC:19358 IMAGE:4239913) | 2.8 | 2.61 |
| 455 | *Zfand3* | Zinc finger, AN1-type domain 3 (Zfand3), mRNA | 2.09 | 2.25 |
| 456 | *Zfp105* | Zinc finger protein 105 (Zfp105), mRNA | 3.73 | 3.68 |
| 457 | *Zfp217* | Zinc finger protein 217, mRNA (cDNA clone MGC:144027 IMAGE:40096805) | 11.32 | 3.4 |
| 458 | *Zfp238* | Transcriptional repressor RP58 (rp58) | 2.7 | 2.24 |
| 459 | *Zfp362* | Zinc finger protein 362, mRNA (cDNA clone MGC:183875 IMAGE:9087875) | 3.36 | 2.76 |
| 460 | *Zfp41* | Zinc finger protein 41 (Zfp41), transcript variant 2, mRNA | 4.78 | 3.85 |
| 461 | *Zfp512* | Zinc finger protein 512, mRNA (cDNA clone MGC:99957 IMAGE:5390706) | 2.05 | 2.63 |
| 462 | *Zfp629* | Zinc finger protein 629, mRNA (cDNA clone MGC:40695 IMAGE:4481313) | 2.26 | 2.19 |
| 463 | *Zfp821* | RIKEN cDNA 4930566A11 gene, mRNA (cDNA clone IMAGE:5323197) | 3.31 | 4.28 |
| 464 | *Zfp827* | Zinc finger protein 827, mRNA (cDNA clone MGC:184083 IMAGE:9088072) | 2.7 | 2.05 |
| 465 | *Znrf1* | Zinc and ring finger 1 (Znrf1), mRNA | 2.36 | 2.74 |
