# Supplementary material for: Neotenic phenomenon in gene expression in the skin of Foxn1- deficient (nude) mice - a projection for regenerative skin wound healing
Source: BMC Genomics. 2017 Jan 9;18:56. doi: 10.1186/s12864-016-3401-z (PMC5223329; doi:10.1186/s12864-016-3401-z)
Supplement: Additional file 3: Table S3. — Genes that are up-regulated in nude (vs B6) skin and epidermis, and not in common with E14 (vs E18). (DOCX 103 kb) [file 12864_2016_3401_MOESM3_ESM.docx]

Table S3.

Genes that are up-regulated in nude (vs B6) skin and epidermis, and uncommon with E14 (vs E18).

| No. | Gene | Gene name | Up in nude skin | Up in nude epidermis |
| --- | --- | --- | --- | --- |
| 1 | *14187* | aldo-keto reductase family 1, member B8 | 2.48 | 2.59 |
| 2 | *15953* | interferon gamma inducible protein 47 | 4.99 | 3.89 |
| 3 | *40969* | Membrane-associated ring finger (C3HC4) 1, mRNA (cDNA clone MGC:86120 IMAGE:5703753) | 4.55 |  |
| 4 | *53622* | keratin 85 | 3.5 |  |
| 5 | *69583* | tumor necrosis factor (ligand) superfamily, member 13 | 2.5 | 4 |
| 6 | *73716* |  | 9.43 |  |
| 7 | *102084* |  | 11.41 |  |
| 8 | *107605* | retinol dehydrogenase 1 (all trans) | 3.07 |  |
| 9 | *110557* | histocompatibility 2, Q region locus 6 | 6.13 |  |
| 10 | *209589* | predicted gene 4761 |  | 3.62 |
| 11 | *227588* | predicted gene 13368 |  | 2.28 |
| 12 | *227712* |  | 6.85 |  |
| 13 | *236604* | phosphatidylserine decarboxylase, pseudogene 1 | 3.06 |  |
| 14 | *269105* | predicted gene 5048 | 2.08 |  |
| 15 | *385356* | predicted gene 5388 |  | 6.55 |
| 16 | *433230* | predicted gene 5515 | 2.56 |  |
| 17 | *434782* | predicted gene 5637 | 2.14 | 3.03 |
| 18 | *435489* | predicted gene 5678 | 13.41 | 5.75 |
| 19 | *435565* | interferon-inducible GTPase-like | 6.1 | 4.44 |
| 20 | *436493* | MHC class I like protein GS10 | 4.1 |  |
| 21 | *494448* | chromobox homolog 6 |  | 3.63 |
| 22 | *545369* | predicted gene 5835 | 2.1 | 4.36 |
| 23 | *545767* | predicted gene 5869 |  | 15.31 |
| 24 | *547345* | similar to archain | 9.74 |  |
| 25 | *547361* | similar to Interferon-activatable protein 203 (Ifi-203) (Interferon-inducible protein p203) | 3.2 |  |
| 26 | *619441* | Tnfsf12-tnfsf13 readthrough transcript | 2.49 | 3.71 |
| 27 | *619734* | predicted gene 6096 | 2.79 |  |
| 28 | *620603* | predicted gene 6166 | 7.5 | 6.22 |
| 29 | *621495* | keratin associated protein 28-10 | 7.41 |  |
| 30 | *623453* | predicted gene 13669 | 2.33 |  |
| 31 | *624889* | predicted gene 6535 | 2.35 |  |
| 32 | *627695* | predicted gene 6780 |  | 6.33 |
| 33 | *627998* | ATP synthase, H+ transporting, mitochondrial F0 complex, subunit g pseudogene | 2.08 |  |
| 34 | *629974* |  | 2.96 |  |
| 35 | *640549* | predicted gene 7301 | 3.66 |  |
| 36 | *664840* | predicted gene 12643 |  | 2.92 |
| 37 | *665380* | predicted gene 11989 |  | 2.24 |
| 38 | *666622* | predicted gene 8195 | 2.87 |  |
| 39 | *666941* | predicted gene 8372 | 2.01 |  |
| 40 | *667230* | predicted gene 8526 | 5.68 |  |
| 41 | *667287* | predicted gene 8557 | 3.79 |  |
| 42 | *667606* | predicted gene 8722 |  | 5.45 |
| 43 | *667617* | predicted gene 8729 | 2.42 |  |
| 44 | *668063* | predicted gene 8956 |  | 11.88 |
| 45 | *668181* | neutrophil cytosolic factor 2 related sequence | 2.79 |  |
| 46 | *668548* | predicted gene 9234 |  | 2.06 |
| 47 | *668701* | predicted gene 9309 |  | 10.25 |
| 48 | *669005* | similar to NFkB interacting protein 1 | 3.37 |  |
| 49 | *669792* | ribosomal protein L21 pseudogene |  | 3.22 |
| 50 | *672660* | predicted gene 9572 | 5.29 |  |
| 51 | *674228* | predicted gene 9616 | 2.8 |  |
| 52 | *675238* |  | 6.14 |  |
| 53 | *676933* | predicted gene 9698 | 4.31 |  |
| 54 | *100038890* |  | 3.13 |  |
| 55 | *100039028* | major urinary protein 11 | 75.68 |  |
| 56 | *100039092* |  | 2.28 |  |
| 57 | *100039117* | predicted gene 2051 | 5.79 |  |
| 58 | *100039247* | major urinary protein 19 | 38.51 |  |
| 59 | *100039583* | predicted gene 2326 | 6.99 | 5.03 |
| 60 | *100039716* | predicted gene 15131 |  | 8.92 |
| 61 | *100040069* |  | 3.12 |  |
| 62 | *100040340* | cytochrome c oxidase, subunit Vb pseudogene |  | 2.17 |
| 63 | *100040742* |  | 2.85 |  |
| 64 | *100041265* | Tu translation elongation factor pseudogene | 2.84 |  |
| 65 | *100041464* | predicted gene 3355 | 3.37 |  |
| 66 | *100041878* |  | 3.38 |  |
| 67 | *100041953* | predicted gene 10094 | 4.3 | 2.08 |
| 68 | *100042229* | predicted gene 3735 | 2.75 |  |
| 69 | *100042424* | PREDICTED: Mus musculus similar to TF-1 apoptosis related protein 19 (LOC100042424), mRNA |  | 2.07 |
| 70 | *100042581* | PREDICTED: Mus musculus similar to CG4877-PA (LOC100042581), mRNA | 83.49 | 5.85 |
| 71 | *100042757* | predicted gene 4013 | 6.7 |  |
| 72 | *100042918* | predicted gene 9763 | 3.09 |  |
| 73 | *100043039* | PREDICTED: Mus musculus similar to SEC61 gamma (LOC100043039), mRNA | 2.48 |  |
| 74 | *100043256* | suppressor of cytokine signaling 2 pseudogene | 2.24 |  |
| 75 | *100043504* | predicted gene 4484 | 7.07 |  |
| 76 | *100043657* | predicted gene 4573 | 3.84 |  |
| 77 | *100043798* | predicted gene 4655 | 2.03 |  |
| 78 | *100043947* | predicted gene 4728 | 5.24 |  |
| 79 | *100044019* | similar to H-2 class I histocompatibility antigen, Q7 alpha chain precursor (QA-2 antigen) | 5.46 |  |
| 80 | *100044021* | similar to MHC class Ib antigen | 3.93 |  |
| 81 | *100044041* | similar to coat protein delta-cop | 7.41 |  |
| 82 | *100044042* | similar to Archain 1 | 9.95 |  |
| 83 | *100044068* | similar to interferon-inducible Ifi202b | 2.81 |  |
| 84 | *100048863* | similar to adenomatosis polyposis coli |  | 3.23 |
| 85 | *100048884* | novel member of the major urinary protein (Mup) gene family | 38.52 |  |
| 86 | *0610031J06Rik* | RIKEN cDNA 0610031J06 gene, mRNA (cDNA clone MGC:27637 IMAGE:4507218) | 2.27 |  |
| 87 | *1100001G20Rik* | RIKEN cDNA 1100001G20 gene, mRNA (cDNA clone IMAGE:1364952) | 8.27 |  |
| 88 | *1110008P14Rik* | RIKEN cDNA 1110008P14 gene, mRNA (cDNA clone MGC:19388 IMAGE:2812475) |  | 4.59 |
| 89 | *1110032A04Rik* | RIKEN cDNA 1110032A04 gene (1110032A04Rik), mRNA | 5.06 |  |
| 90 | *1200002N14Rik* | RIKEN cDNA 1200002N14 gene (1200002N14Rik), mRNA | 3.48 |  |
| 91 | *1600029D21Rik* | RIKEN cDNA 1600029D21 gene (1600029D21Rik), mRNA | 31.47 | 2.4 |
| 92 | *1700012B15Rik* | PREDICTED: Mus musculus similar to 1700012B15Rik protein (LOC100041946), mRNA | 2.63 |  |
| 93 | *1700112E06Rik* | PREDICTED: Mus musculus RIKEN cDNA 1700112E06 gene, transcript variant 2 (1700112E06Rik), mRNA | 2.12 |  |
| 94 | *1810011O10Rik* | RIKEN cDNA 1810011O10 gene (1810011O10Rik), mRNA |  | 3.23 |
| 95 | *1810013D10Rik* | RIKEN cDNA 1810013D10 gene, mRNA (cDNA clone IMAGE:1379146) |  | 7.3 |
| 96 | *1810074P20Rik* | RIKEN cDNA 1810074P20 gene (1810074P20Rik), mRNA |  | 2.06 |
| 97 | *2300005B03Rik* | SLURP-2 mRNA for secreted Ly6/uPAR related protein-2 | 11.04 | 23.09 |
| 98 | *2310005E10Rik* | RIKEN cDNA 2310005E10 gene, mRNA (cDNA clone MGC:46975 IMAGE:3661180) | 2.07 |  |
| 99 | *2310007H09Rik* | RIKEN cDNA 2310007H09 gene (2310007H09Rik), mRNA | 3.08 | 2.19 |
| 100 | *2310008H09Rik* | RIKEN cDNA 2310008H09 gene (2310008H09Rik), mRNA |  | 3.56 |
| 101 | *2310046K01Rik* | RIKEN cDNA 2310046K01 gene (2310046K01Rik), mRNA | 5.02 |  |
| 102 | *2310047M10Rik* | RIKEN cDNA 2310047M10 gene (2310047M10Rik), mRNA | 2.15 |  |
| 103 | *2310047O13Rik* | RIKEN cDNA 2310047O13 gene, mRNA (cDNA clone MGC:28605 IMAGE:4217391) | 2.06 |  |
| 104 | *2310061C15Rik* | RIKEN cDNA 2310061C15 gene (2310061C15Rik), mRNA |  | 2.39 |
| 105 | *2510049J12Rik* | RIKEN cDNA 2510049J12 gene (2510049J12Rik), mRNA | 18.12 | 3.87 |
| 106 | *2810004N23Rik* | RIKEN cDNA 2810004N23 gene, mRNA (cDNA clone MGC:35729 IMAGE:5354472) |  | 2.57 |
| 107 | *2810417H13Rik* | RIKEN cDNA 2810417H13 gene, mRNA (cDNA clone MGC:29306 IMAGE:5006272) |  | 2.18 |
| 108 | *2900064A13Rik* | RIKEN cDNA 2900064A13 gene (2900064A13Rik), mRNA | 2.42 |  |
| 109 | *3110003A17Rik* | RIKEN cDNA 3110003A17 gene, mRNA (cDNA clone IMAGE:1448067) | 3.01 |  |
| 110 | *4632428N05Rik* | RIKEN cDNA 4632428N05 gene, mRNA (cDNA clone MGC:7407 IMAGE:3488482) | 3.06 |  |
| 111 | *4732429D16Rik* | RIKEN cDNA 4732429D16 gene (4732429D16Rik), mRNA | 6.37 |  |
| 112 | *4921525H12Rik* | PREDICTED: Mus musculus RIKEN cDNA 4921525H12 gene (4921525H12Rik), mRNA |  | 2.87 |
| 113 | *4930402E16Rik* | RIKEN cDNA 4930402E16 gene, mRNA (cDNA clone MGC:159128 IMAGE:40129940) | 2.99 |  |
| 114 | *4930506M07Rik* | RIKEN cDNA 4930506M07 gene, mRNA (cDNA clone MGC:40679 IMAGE:2599101) | 2.67 |  |
| 115 | *4930526A20Rik* | PREDICTED: Mus musculus RIKEN cDNA 4930526A20 gene (4930526A20Rik), misc RNA | 3.47 |  |
| 116 | *4930562F07Rik* | PREDICTED: Mus musculus RIKEN cDNA 4930562F07 gene (4930562F07Rik), mRNA | 5.01 |  |
| 117 | *4933409G03Rik* | RIKEN cDNA 4933409G03 gene (4933409G03Rik), mRNA | 3.27 |  |
| 118 | *4933427G17Rik* | PREDICTED: Mus musculus RIKEN cDNA 4933427G17 gene, transcript variant 1 (4933427G17Rik), mRNA | 2.24 |  |
| 119 | *4933433P14Rik* | RIKEN cDNA 4933433P14 gene, mRNA (cDNA clone MGC:27787 IMAGE:3156862) | 2.21 |  |
| 120 | *5430427O19Rik* | RIKEN cDNA 5430427O19 gene, mRNA (cDNA clone MGC:144455 IMAGE:40102201) | 2.83 |  |
| 121 | *5430435G22Rik* | RIKEN cDNA 5430435G22 gene (5430435G22Rik), mRNA | 4.86 |  |
| 122 | *5730410E15Rik* | M-Golsyn mRNA for Golgi-localized syntaphilin-related protein A | 2.25 |  |
| 123 | *5730590G19Rik* | RIKEN cDNA 5730590G19 gene, mRNA (cDNA clone MGC:189993 IMAGE:9088180) |  | 3.71 |
| 124 | *6330409N04Rik* | RIKEN cDNA 6330409N04 gene, mRNA (cDNA clone MGC:47174 IMAGE:5344409) | 2.57 |  |
| 125 | *9030619P08Rik* | RIKEN cDNA 9030619P08 gene, mRNA (cDNA clone MGC:182209 IMAGE:9056103) | 6.9 |  |
| 126 | *9130008F23Rik* | RIKEN cDNA 9130008F23 gene, mRNA (cDNA clone MGC:163696 IMAGE:40130342) | 3.11 |  |
| 127 | *A430005L14Rik* | RIKEN cDNA A430005L14 gene (A430005L14Rik), mRNA | 2.08 |  |
| 128 | *A630001G21Rik* | PREDICTED: Mus musculus RIKEN cDNA A630001G21 gene, transcript variant 3 (A630001G21Rik), mRNA | 3.31 |  |
| 129 | *AA467197* | Expressed sequence AA467197 (AA467197), mRNA | 59.78 | 9.83 |
| 130 | *Aadac* | Arylacetamide deacetylase (esterase), mRNA (cDNA clone MGC:28468 IMAGE:4162194) | 6.49 | 2.62 |
| 131 | *Aadacl1* | Arylacetamide deacetylase-like 1, mRNA (cDNA clone MGC:105251 IMAGE:30550100) | 2.81 |  |
| 132 | *Abcc1* | ATP-binding cassette protein (Abcc1b) | 2.56 |  |
| 133 | *Abcc4* | ATP-binding cassette, sub-family C (CFTR/MRP), member 4, mRNA (cDNA clone MGC:183733 IMAGE:9087733) | 2.06 |  |
| 134 | *Abcf2* | ATP-binding cassette, sub-family F (GCN20), member 2 (Abcf2), nuclear gene encoding mitochondrial protein, mRNA |  | 6.8 |
| 135 | *Abcg2* | Breast cancer resistance protein 1 (Bcrp1) |  | 3.1 |
| 136 | *Abhd5* | Abhydrolase domain containing 5, mRNA (cDNA clone MGC:46776 IMAGE:4985992) | 2.99 |  |
| 137 | *Abhd6* | Abhydrolase domain containing 6, mRNA (cDNA clone MGC:35771 IMAGE:5369937) | 2.56 |  |
| 138 | *Abi3* | Phosphatase, orphan 1 (Phospho1), mRNA | 2.03 |  |
| 139 | *Acaa1a* | Acetyl-Coenzyme A acyltransferase 1A, mRNA (cDNA clone IMAGE:5007262) | 2.53 |  |
| 140 | *Acer1* | Alkaline ceramidase | 8.12 |  |
| 141 | *Aco1* | Aconitase 1, mRNA (cDNA clone MGC:6247 IMAGE:3494686) |  | 2.06 |
| 142 | *Acot1* | Acyl-CoA thioesterase 1, mRNA (cDNA clone MGC:40635 IMAGE:5100478) | 3.42 | 2.45 |
| 143 | *Acot7* | BACH mRNA for acyl-CoA hydrolase, complete cds, isoform mBACHb |  | 2.86 |
| 144 | *Acox1* | Acyl-Coenzyme A oxidase 1, palmitoyl (Acox1), mRNA | 2.06 |  |
| 145 | *Acox2* | Acyl-Coenzyme A oxidase 2, branched chain, mRNA (cDNA clone MGC:29247 IMAGE:5052291) | 14.28 | 2.49 |
| 146 | *Acp5* | Acid phosphatase 5, tartrate resistant, mRNA (cDNA clone MGC:7956 IMAGE:3584530) |  | 4.8 |
| 147 | *Acpp* | Acid phosphatase, prostate (Acpp), transcript variant 2, mRNA | 5.66 |  |
| 148 | *Acsbg2* | Acyl-CoA synthetase bubblegum family member 2 (Acsbg2), mRNA |  | 2.84 |
| 149 | *Acsl4* | Acyl-CoA synthetase long-chain family member 4, mRNA (cDNA clone MGC:5674 IMAGE:3157957) | 2.09 | 2.1 |
| 150 | *Acsl5* | Acyl-CoA synthetase long-chain family member 5, mRNA (cDNA clone MGC:18968 IMAGE:3987201) | 3.63 |  |
| 151 | *Acss2* | CDNA fis, clone TRACH2001275, highly similar to Mus musculus acetyl-CoA synthetase mRNA |  | 2.02 |
| 152 | *Actn4* | Actinin alpha 4, mRNA (cDNA clone MGC:18652 IMAGE:3157024) |  | 5.49 |
| 153 | *Ada* | Strain ILS adenosine deaminase | 2.97 | 4.29 |
| 154 | *Adamts2* | A disintegrin-like and metallopeptidase (reprolysin type) with thrombospondin type 1 motif, 2, mRNA (cDNA clone IMAGE:4004191 | 3.06 |  |
| 155 | *Adfp* | C57BL/6J adipose differentiation-related protein |  | 3.67 |
| 156 | *Adh7* | C57BL/6J alcohol dehydrogenase class 4 (Adh4) | 6.73 |  |
| 157 | *Ado* | 2-aminoethanethiol (cysteamine) dioxygenase (Ado), mRNA |  | 2.33 |
| 158 | *AF251705* | Polymeric immunoglobulin receptor 5 precursor (Pigr5) | 8.35 |  |
| 159 | *Agpat3* | 1-acylglycerol-3-phosphate O-acyltransferase 3 (Agpat3), mRNA |  | 2.48 |
| 160 | *Ahrr* | Aryl-hydrocarbon receptor repressor (Ahrr), mRNA | 2.18 |  |
| 161 | *AI317395* | Expressed sequence AI317395 (AI317395), mRNA | 2.08 |  |
| 162 | *AI413582* | Expressed sequence AI413582, mRNA (cDNA clone MGC:41609 IMAGE:1264774) | 3.91 |  |
| 163 | *AI597468* | Expressed sequence AI597468 (AI597468), mRNA |  | 2.51 |
| 164 | *Aifm2* | Apoptosis-inducing factor, mitochondrion-associated 2 (Aifm2), nuclear gene encoding mitochondrial protein, transcript varian |  | 2.1 |
| 165 | *Ak3* | Adenylate kinase 3 (Ak3), nuclear gene encoding mitochondrial protein, mRNA |  | 2.85 |
| 166 | *Akap2* | A kinase (PRKA) anchor protein 2, mRNA (cDNA clone MGC:5810 IMAGE:3493391) |  | 2.84 |
| 167 | *Akirin1* | Akirin 1, mRNA (cDNA clone MGC:7743 IMAGE:3498569) |  | 3.81 |
| 168 | *Akr1c13* | Aldo-keto reductase family 1, member C13, mRNA (cDNA clone MGC:36009 IMAGE:5100366) | 2.53 |  |
| 169 | *Alcam* | Activated leukocyte cell adhesion molecule, mRNA (cDNA clone MGC:27910 IMAGE:3501215) |  | 2.13 |
| 170 | *Aldh1a3* | Aldehyde dehydrogenase family 1, subfamily A3, mRNA (cDNA clone IMAGE:5005066) | 2.27 |  |
| 171 | *Aldh1l1* | Aldehyde dehydrogenase 1 family, member L1, mRNA (cDNA clone MGC:36387 IMAGE:5098789) | 4.53 |  |
| 172 | *Aldh3a1* | Aldehyde dehydrogenase family 3, subfamily A1 (Aldh3a1), transcript variant 1, mRNA | 29.82 | 2.83 |
| 173 | *Aldoa* | Aldolase A, fructose-bisphosphate (Aldoa), mRNA |  | 3.71 |
| 174 | *Aldob* | Aldolase 2, B isoform, mRNA (cDNA clone IMAGE:4972777) | 2.02 |  |
| 175 | *Aldoc* | Aldolase C, fructose-bisphosphate, mRNA (cDNA clone MGC:6732 IMAGE:3589205) | 2.09 |  |
| 176 | *Alg13* | Asparagine-linked glycosylation 13 homolog (S. cerevisiae) (Alg13), mRNA | 2.03 | 2 |
| 177 | *Alkbh7* | AlkB, alkylation repair homolog 7 (E. coli) (Alkbh7), mRNA |  | 2.84 |
| 178 | *Alox12e* | Arachidonate lipoxygenase, epidermal (Alox12e), mRNA | 23.82 | 5.49 |
| 179 | *Alox5ap* | Arachidonate 5-lipoxygenase activating protein, mRNA (cDNA clone MGC:41698 IMAGE:1349819) | 4.81 |  |
| 180 | *Ammecr1* | Alport syndrome, mental retardation, midface hypoplasia and elliptocytosis chromosomal region gene 1 homolog (human), mRNA (c |  | 4.89 |
| 181 | *Amy1* | Amylase 1, salivary, mRNA (cDNA clone MGC:11568 IMAGE:3710941) | 5.33 |  |
| 182 | *Ankrd13a* | Ankyrin repeat domain 13a (Ankrd13a), mRNA |  | 4.83 |
| 183 | *Ankrd22* | Ankyrin repeat domain 22, mRNA (cDNA clone MGC:6812 IMAGE:2648395) | 4.88 |  |
| 184 | *Ankrd34a* | Ankyrin repeat domain 34A, mRNA (cDNA clone MGC:182823 IMAGE:9087437) | 3.73 |  |
| 185 | *Ankrd37* | Ankyrin repeat domain 37, mRNA (cDNA clone MGC:49359 IMAGE:5352548) | 2.72 |  |
| 186 | *Ankrd49* | Ankyrin repeat domain 49, mRNA (cDNA clone MGC:25252 IMAGE:3969390) |  | 2.33 |
| 187 | *Ankrd9* | Ankyrin repeat domain 9, mRNA (cDNA clone MGC:38320 IMAGE:5343549) |  | 2.58 |
| 188 | *Anp32b* | Acidic (leucine-rich) nuclear phosphoprotein 32 family, member B (Anp32b), mRNA |  | 2.34 |
| 189 | *Anxa1* | Annexin A1, mRNA (cDNA clone MGC:8074 IMAGE:3587973) | 4.51 |  |
| 190 | *Anxa4* | Annexin A4 (Anxa4), mRNA | 3.97 |  |
| 191 | *Ap4s1* | Adaptor-related protein complex AP-4, sigma 1 (Ap4s1), mRNA | 3.35 |  |
| 192 | *Apc* | Adenomatosis polyposis coli (Apc), mRNA |  | 2.17 |
| 193 | *Apob48r* | Apolipoprotein B48 receptor, mRNA (cDNA clone MGC:28748 IMAGE:4482457) | 4.77 |  |
| 194 | *Apobec3* | Apolipoprotein B mRNA editing enzyme, catalytic polypeptide 3, mRNA (cDNA clone MGC:7002 IMAGE:3155422) |  | 3.27 |
| 195 | *Apod* | Apolipoprotein D (Apod), mRNA | 3.8 |  |
| 196 | *Apoe* | Apolipoprotein E, mRNA (cDNA clone MGC:36251 IMAGE:5136415) | 5.1 |  |
| 197 | *App* | Amyloid beta (A4) precursor protein, mRNA (cDNA clone IMAGE:3486773) | 2.21 |  |
| 198 | *Aqp3* | Aquaporin 3, mRNA (cDNA clone MGC:35966 IMAGE:4951027) | 9.43 |  |
| 199 | *Aqp9* | Aquaporin 9, mRNA (cDNA clone MGC:35844 IMAGE:5096952) | 7.87 |  |
| 200 | *Arap2* | ArfGAP with RhoGAP domain, ankyrin repeat and PH domain 2, mRNA (cDNA clone IMAGE:3710592) | 4.59 |  |
| 201 | *Areg* | Amphiregulin, mRNA (cDNA clone MGC:11536 IMAGE:3597695) | 8.77 |  |
| 202 | *Arg2* | Arginase type II, mRNA (cDNA clone MGC:31006 IMAGE:5254031) | 3.5 |  |
| 203 | *Arhgap9* | Rho GTPase activating protein 9 (Arhgap9), mRNA | 3.13 |  |
| 204 | *Arl5c* | ADP-ribosylation factor-like 5C (Arl5c), mRNA | 4.43 |  |
| 205 | *Arl8b* | ADP-ribosylation factor-like 8B (Arl8b), mRNA | 2.03 |  |
| 206 | *Arpc3* | Actin related protein 2/3 complex, subunit 3, mRNA (cDNA clone MGC:18996 IMAGE:4013537) |  | 2.34 |
| 207 | *Arsg* | Arylsulfatase G, mRNA (cDNA clone IMAGE:5042793) | 2.06 |  |
| 208 | *Art3* | ART3 mono(ADP-ribosyl)transferase (art3 gene), splice variant 1 |  | 2.92 |
| 209 | *Atf4* | Activating transcription factor 4 (Atf4), mRNA |  | 2.89 |
| 210 | *Atf5* | NRIF3-associated protein (Nap1) |  | 4.48 |
| 211 | *Atg5* | Autophagy-related 5 (yeast) (Atg5), mRNA |  | 2.03 |
| 212 | *Atox1* | ATX1 (antioxidant protein 1) homolog 1 (yeast), mRNA (cDNA clone MGC:41183 IMAGE:1328907) | 2.08 |  |
| 213 | *Atp10b* | MKIAA0715 protein | 5.76 |  |
| 214 | *Atp12a* | ATPase, H+/K+ transporting, nongastric, alpha polypeptide (Atp12a), mRNA | 57.57 | 3.49 |
| 215 | *Atp5e* | ATP synthase, H+ transporting, mitochondrial F1 complex, epsilon subunit, mRNA (cDNA clone MGC:35685 IMAGE:4981796) |  | 2.57 |
| 216 | *Atp5j* | ATP synthase, H+ transporting, mitochondrial F0 complex, subunit F, mRNA (cDNA clone MGC:18567 IMAGE:4219715) |  | 2.27 |
| 217 | *Atp5j2* | ATP synthase, H+ transporting, mitochondrial F0 complex, subunit f, isoform 2, mRNA (cDNA clone MGC:35884 IMAGE:2651401) | 2.58 |  |
| 218 | *Atp6v0a4* | Vacuolar proton translocating ATPase a4 isoform | 2.98 |  |
| 219 | *Atp6v1a* | ATPase, H+ transporting, lysosomal V1 subunit A, mRNA (cDNA clone MGC:6531 IMAGE:2651677) |  | 2.26 |
| 220 | *Atp6v1c2* | ATPase, H+ transporting, lysosomal V1 subunit C2 (Atp6v1c2), mRNA |  | 8.33 |
| 221 | *Atp6v1e1* | V-ATPase E2 subunit | 3.63 |  |
| 222 | *Atxn1* | Ataxin-1 |  | 2.78 |
| 223 | *AU018091* | Cat5 mRNA for cationic amino acid transporter 5 | 5.73 |  |
| 224 | *Avpi1* | Arginine vasopressin-induced 1, mRNA (cDNA clone MGC:41363 IMAGE:1344413) | 3.11 |  |
| 225 | *AW112010* | Small secreted protein interferon-induced | 25.17 | 4.66 |
| 226 | *Awat1* | Diacylglycerol O-acyltransferase 2-like 3, mRNA (cDNA clone MGC:182759 IMAGE:9056653) | 47.68 | 4.34 |
| 227 | *Awat2* | Diacylglycerol O-acyltransferase 2-like 4, mRNA (cDNA clone MGC:169709 IMAGE:8861104) | 31.68 | 2.83 |
| 228 | *B2m* | Beta-2 microglobulin mRNA, segment 1, clones pBRcB-(1-3). | 7.26 |  |
| 229 | *B3gnt2* | Beta-1,3-N-acetylglucosaminyltransferase | 2.36 |  |
| 230 | *B4galnt1* | Beta-1,4-N-acetyl-galactosaminyl transferase 1, mRNA (cDNA clone MGC:46928 IMAGE:5038560) | 4.16 | 3 |
| 231 | *B630005N14Rik* | RIKEN cDNA B630005N14 gene, mRNA (cDNA clone IMAGE:5345159) | 2.71 |  |
| 232 | *Bak1* | BCL2-antagonist/killer 1 (Bak1), mRNA |  | 7.46 |
| 233 | *Batf* | Basic leucine zipper transcription factor, ATF-like (Batf), mRNA | 5.25 |  |
| 234 | *Batf2* | Basic leucine zipper transcription factor, ATF-like 2, mRNA (cDNA clone MGC:37488 IMAGE:4984403) | 4.34 |  |
| 235 | *Bbc3* | BCL2 binding component 3 (Bbc3), mRNA | 2.09 |  |
| 236 | *Bbox1* | Butyrobetaine (gamma), 2-oxoglutarate dioxygenase 1 (gamma-butyrobetaine hydroxylase) (Bbox1), mRNA | 6.73 |  |
| 237 | *BC003267* | CDNA sequence BC003267 (BC003267), mRNA | 2.59 |  |
| 238 | *BC016579* | CDNA sequence, BC016579 (BC016579), mRNA | 6.24 |  |
| 239 | *BC021614* | CDNA sequence BC021614, mRNA (cDNA clone MGC:19096 IMAGE:4196879) |  | 6.16 |
| 240 | *BC037703* | CDNA sequence BC037703, mRNA (cDNA clone MGC:47256 IMAGE:4166596) |  | 3.06 |
| 241 | *BC055004* | CDNA sequence BC055004, mRNA (cDNA clone MGC:118496 IMAGE:6396133) | 11.47 |  |
| 242 | *BC066028* | CDNA sequence BC066028 (BC066028), mRNA |  | 3.05 |
| 243 | *Bcl2l15* | BCLl2-like 15, mRNA (cDNA clone IMAGE:1247650) | 9.57 |  |
| 244 | *Bcl3* | B-cell leukemia/lymphoma 3 (Bcl3), mRNA | 3.2 | 5.36 |
| 245 | *Bcl7b* | B-cell CLL/lymphoma 7B, mRNA (cDNA clone MGC:18340 IMAGE:3660754) | 3.01 |  |
| 246 | *Bdh1* | 3-hydroxybutyrate dehydrogenase, type 1, mRNA (cDNA clone IMAGE:5051325) | 2.17 | 3.16 |
| 247 | *Bdh2* | 3-hydroxybutyrate dehydrogenase, type 2 (Bdh2), mRNA | 2.32 |  |
| 248 | *Becn1* | Beclin 1, autophagy related, mRNA (cDNA clone MGC:6843 IMAGE:2650059) |  | 2.44 |
| 249 | *Bhlhe40* | Basic helix-loop-helix family, member e40, mRNA (cDNA clone MGC:11663 IMAGE:3707474) |  | 5.41 |
| 250 | *Bicc1* | Bicaudal C homolog 1 (Drosophila) (Bicc1), mRNA | 2.77 |  |
| 251 | *Bicd2* | Bicaudal D homolog 2 (Drosophila), mRNA (cDNA clone MGC:38246 IMAGE:5324113) | 3.02 |  |
| 252 | *Bin3* | Bridging integrator 3, mRNA (cDNA clone MGC:35876 IMAGE:5040094) | 2.93 |  |
| 253 | *Bnc1* | Basonuclin |  | 2.44 |
| 254 | *Bok* | BCL2-related ovarian killer protein, mRNA (cDNA clone MGC:41110 IMAGE:2936938) | 2.54 | 2.01 |
| 255 | *Bola2* | BolA-like 2 (E. coli), mRNA (cDNA clone MGC:74338 IMAGE:6707992) | 2.25 |  |
| 256 | *Brunol4* | BRUL4 (Brul4) |  | 2.51 |
| 257 | *Bst1* | Bone marrow stromal cell antigen 1, mRNA (cDNA clone IMAGE:5356458) | 9.56 |  |
| 258 | *Bst2* | Bone marrow stromal cell antigen 2, mRNA (cDNA clone MGC:28276 IMAGE:4009434) |  | 3.35 |
| 259 | *Bzw1* | Basic leucine zipper and W2 domains 1, mRNA (cDNA clone MGC:25505 IMAGE:4910833) |  | 2.28 |
| 260 | *Bzw2* | Basic leucine zipper and W2 domains 2, mRNA (cDNA clone MGC:7203 IMAGE:3482251) |  | 4.81 |
| 261 | *C1qb* | Complement component 1, q subcomponent, beta polypeptide (C1qb), mRNA | 5.15 | 2.51 |
| 262 | *C1qc* | Complement component 1, q subcomponent, C chain (C1qc), mRNA | 2.47 |  |
| 263 | *C1qtnf3* | C1q and tumor necrosis factor related protein 3, mRNA (cDNA clone IMAGE:3989958) | 5.91 |  |
| 264 | *C1r* | Complement component 1, r subcomponent, mRNA (cDNA clone MGC:6404 IMAGE:3586055) | 6.62 |  |
| 265 | *C3ar1* | Complement component 3a receptor 1, mRNA (cDNA clone MGC:5787 IMAGE:3593758) | 4.08 |  |
| 266 | *C5ar1* | Complement component 5a receptor 1 (C5ar1), mRNA | 3.52 |  |
| 267 | *Cab39* | Calcium binding protein 39, mRNA (cDNA clone IMAGE:4952585) |  | 2.05 |
| 268 | *Cacna2d2* | Voltage-dependent calcium channel alpha-2-delta-2 mutant subunit 1 (Cacna2d2) |  | 2.38 |
| 269 | *Cadps2* | Ca2+-dependent activator protein for secretion 2, mRNA (cDNA clone IMAGE:5324294) | 2.68 |  |
| 270 | *Calcb* | Calcitonin-related polypeptide, beta (Calcb), mRNA | 8.83 |  |
| 271 | *Capn2* | Calpain 2 (Capn2), mRNA |  | 2.85 |
| 272 | Capns1 | Calpain, small subunit 1, mRNA (cDNA clone MGC:25263 IMAGE:3156021) |  | 2 |
| 273 | *Car2* | Carbonic anhydrase 2 (Car2), mRNA |  | 9.33 |
| 274 | *Car6* | Carbonic anhydrase 6 (Car6), mRNA | 3.96 |  |
| 275 | *Car9* | Carbonic anhydrase 9 (Car9), mRNA |  | 4.36 |
| 276 | *Card14* | Caspase recruitment domain family, member 14, mRNA (cDNA clone MGC:28122 IMAGE:3979883) | 3.69 |  |
| 277 | *Casp1* | Caspase 1, mRNA (cDNA clone MGC:6106 IMAGE:3583883) | 9.81 | 3.64 |
| 278 | *Casp12* | Caspase 12, mRNA (cDNA clone MGC:36090 IMAGE:5375109) | 3.13 |  |
| 279 | *Casp4* | Caspase 4, apoptosis-related cysteine peptidase (Casp4), mRNA | 7.11 | 5.47 |
| 280 | *Casp8* | Caspase 8, mRNA (cDNA clone MGC:11440 IMAGE:3964119) |  | 2.22 |
| 281 | *Cbr1* | Carbonyl reductase 1, mRNA (cDNA clone MGC:14021 IMAGE:4217206) | 2.6 |  |
| 282 | *Cbr2* | Carbonyl reductase 2, mRNA (cDNA clone MGC:18465 IMAGE:4219349) | 4.03 |  |
| 283 | *Cbr3* | Carbonyl reductase 3, mRNA (cDNA clone MGC:41226 IMAGE:1313642) | 4.13 |  |
| 284 | *Cbx3* | Chromobox homolog 3 (Drosophila HP1 gamma), mRNA (cDNA clone MGC:25433 IMAGE:3990224) |  | 2.46 |
| 285 | Ccar1 | Cell division cycle and apoptosis regulator 1, mRNA (cDNA clone IMAGE:5009064) |  | 2 |
| 286 | *Ccdc34* | Coiled-coil domain containing 34 (Ccdc34), mRNA |  | 2.49 |
| 287 | *Ccdc49* | Coiled-coil domain containing 49 (Ccdc49), mRNA |  | 2.71 |
| 288 | *Ccdc58* | Coiled-coil domain containing 58, mRNA (cDNA clone MGC:36453 IMAGE:5356370) |  | 2.17 |
| 289 | *Ccl1* | Chemokine (C-C motif) ligand 1 (Ccl1), mRNA | 26.7 | 10.31 |
| 290 | *Ccl11* | Chemokine (C-C motif) ligand 11, mRNA (cDNA clone MGC:41147 IMAGE:1527856) | 9.84 |  |
| 291 | *Ccl12* | Chemokine (C-C motif) ligand 12, mRNA (cDNA clone MGC:41146 IMAGE:1548072) | 7.21 |  |
| 292 | *Ccl2* | Strain NOD/LtJ small inducible cytokine A2 precursor (Scya2) | 16.85 | 5.68 |
| 293 | *Ccl20* | Chemokine (C-C motif) ligand 20, mRNA (cDNA clone MGC:41109 IMAGE:1380543) | 48.36 | 7.66 |
| 294 | *Ccl22* | Chemokine (C-C motif) ligand 22, mRNA (cDNA clone MGC:13812 IMAGE:4192393) | 13.18 | 5.65 |
| 295 | *Ccl24* | Eotaxin-2 (Scya24) | 2.52 |  |
| 296 | *Ccl27a* | Chemokine (C-C motif) ligand 27A, mRNA (cDNA clone MGC:41145 IMAGE:3471454) | 10.25 | 3.06 |
| 297 | *Ccl6* | Chemokine (C-C motif) ligand 6, mRNA (cDNA clone MGC:6215 IMAGE:3492808) | 9.69 |  |
| 298 | *Ccl7* | Chemokine (C-C motif) ligand 7 (Ccl7), mRNA | 3.23 |  |
| 299 | *Ccl8* | Chemokine (C-C motif) ligand 8 (Ccl8), mRNA | 236.67 | 2.74 |
| 300 | *Ccl9* | Strain SJL/J small inducible cytokine A10 (ScyA10) | 6.8 |  |
| 301 | *Ccnb1* | Cyclin B1, mRNA (cDNA clone MGC:18763 IMAGE:4014448) |  | 2.34 |
| 302 | *Ccr2* | Chemokine (C-C motif) receptor 2 (Ccr2), mRNA | 6.94 |  |
| 303 | *Ccr5* | Chemokine (C-C motif) receptor 5 (Ccr5), mRNA | 9.84 |  |
| 304 | *Ccr7* | Chemokine (C-C motif) receptor 7 (Ccr7), mRNA | 8.35 |  |
| 305 | *Ccr9* | Chemokine receptor (CCR9 gene) | 2.12 |  |
| 306 | *Ccrl1* | Chemokine (C-C motif) receptor-like 1 (Ccrl1), mRNA | 3.34 |  |
| 307 | *Cd163* | CD163 antigen (Cd163), mRNA | 9.77 |  |
| 308 | *Cd207* | CD207 antigen (Cd207), mRNA | 40.74 |  |
| 309 | *Cd209a* | CD209a antigen (Cd209a), mRNA | 12.94 |  |
| 310 | *Cd209b* | Type II transmembrane receptor OtB7 | 6.01 |  |
| 311 | *Cd209d* | CD209d antigen (Cd209d), mRNA | 19.38 |  |
| 312 | *Cd209f* | CD209f antigen, mRNA (cDNA clone IMAGE:1383444) | 17.92 |  |
| 313 | *Cd209g* | SIGNR7 protein precursor | 9.95 |  |
| 314 | *Cd302* | CD302 antigen, mRNA (cDNA clone MGC:7450 IMAGE:3489789) | 3.28 |  |
| 315 | *Cd37* | CD37 antigen, mRNA (cDNA clone IMAGE:3585638) | 3.42 |  |
| 316 | *Cd3eap* | CD3E antigen, epsilon polypeptide associated protein (Cd3eap), mRNA |  | 2.65 |
| 317 | *Cd48* | CD48 antigen (Cd48), mRNA | 9.34 |  |
| 318 | *Cd63* | CD63 antigen, mRNA (cDNA clone MGC:6087 IMAGE:3157993) | 2.31 |  |
| 319 | *Cd74* | CD74 antigen (invariant polypeptide of major histocompatibility complex, class II antigen-associated), mRNA (cDNA clone MGC:6 | 30.6 |  |
| 320 | *Cd86* | CD86 antigen, mRNA (cDNA clone MGC:18471 IMAGE:4008635) | 5.75 |  |
| 321 | *Cdc42ep4* | CDC42 effector protein (Rho GTPase binding) 4, mRNA (cDNA clone MGC:6584 IMAGE:3484427) | 2.07 |  |
| 322 | *Cdk7* | Cyclin-dependent kinase 7 (homolog of Xenopus MO15 cdk-activating kinase), mRNA (cDNA clone MGC:6069 IMAGE:3585145) |  | 2.52 |
| 323 | *Cdkn2a* | Cyclin-dependent kinase inhibitor 2A (Cdkn2a), transcript variant 1, mRNA |  | 3.22 |
| 324 | *Cdkn2b* | Cyclin-dependent kinase inhibitor 2B (p15, inhibits CDK4), mRNA (cDNA clone MGC:5793 IMAGE:3495097) | 7.34 |  |
| 325 | *Ces8* | Carboxylesterase 8 (putative) (Ces8), mRNA | 48.91 | 5.21 |
| 326 | *Cfd* | Complement factor D (adipsin) (Cfd), mRNA | 4.76 |  |
| 327 | *Cfh* | Complement component factor h (Cfh), mRNA | 2.53 |  |
| 328 | *Cfl1* | Cofilin 1, non-muscle (Cfl1), mRNA |  | 2.21 |
| 329 | *Cfp* | Properdin (AA 5 - 441) | 7.5 |  |
| 330 | *Chchd10* | Unknown mRNA |  | 4.45 |
| 331 | *Chchd8* | Coiled-coil-helix-coiled-coil-helix domain containing 8, mRNA (cDNA clone MGC:151372 IMAGE:40126314) |  | 2.57 |
| 332 | *Chi3l3* | Chitinase 3-like 3 (Chi3l3), mRNA | 17.33 |  |
| 333 | *Chic2* | Cysteine-rich hydrophobic domain 2 (Chic2), mRNA | 2.78 |  |
| 334 | *Chsy1* | Chondroitin sulfate synthase 1, mRNA (cDNA clone IMAGE:40061623) |  | 2.05 |
| 335 | *Cib1* | Calcium and integrin binding 1 (calmyrin), mRNA (cDNA clone MGC:5709 IMAGE:3599986) |  | 2.64 |
| 336 | *Cidea* | Cell death-inducing DNA fragmentation factor, alpha subunit-like effector A (Cidea), mRNA |  | 4.55 |
| 337 | *Cidec* | Adipocyte-specific | 8.71 |  |
| 338 | *Cilp* | Cartilage intermediate layer protein, nucleotide pyrophosphohydrolase, mRNA (cDNA clone MGC:90919 IMAGE:4934956) | 13.47 |  |
| 339 | *Cldn1* | Claudin 1, mRNA (cDNA clone MGC:5767 IMAGE:3491319) | 3.61 |  |
| 340 | *Clec12a* | C-type lectin domain family 12, member a, mRNA (cDNA clone MGC:107404 IMAGE:30044572) | 5.36 |  |
| 341 | *Clec2d* | C-type lectin domain family 2, member d (Clec2d), mRNA | 5.92 |  |
| 342 | *Clec4a1* | Dendritic cell inhibitory receptor 4 (Dcir4) | 5.89 |  |
| 343 | *Clec4a2* | Dendritic cell immunoreceptor | 4.88 |  |
| 344 | *Clec4a3* | C-type lectin domain family 4, member a3 (Clec4a3), mRNA | 3.8 |  |
| 345 | *Clec4n* | C-type lectin domain family 4, member n, mRNA (cDNA clone MGC:35901 IMAGE:5376247) | 9.47 |  |
| 346 | *Clic4* | Intracellular chloride channel protein (Clic) mRNA, nuclear gene encoding mitochondrial protein |  | 2.89 |
| 347 | *Clk1* | CDC-like kinase 1 (Clk1), transcript variant 2, mRNA | 2.07 |  |
| 348 | *Clstn3* | Calsyntenin 3 (Clstn3), mRNA |  | 4.82 |
| 349 | *Clu* | Sulfated glycoprotein-2 isoform 2 | 6.76 |  |
| 350 | *Cmah* | CMAH mRNA for CMP-NeuAc hydroxylase, complete cds, major form of alternative splicing | 3.72 |  |
| 351 | *Cmtm4* | CKLF-like MARVEL transmembrane domain containing 4 (Cmtm4), mRNA | 2.05 |  |
| 352 | *Cnbp* | Cellular nucleic acid binding protein (Cnbp), transcript variant 1, mRNA |  | 2.45 |
| 353 | *Cnih4* | Cornichon homolog 4 (Drosophila) (Cnih4), mRNA | 2.07 |  |
| 354 | *Commd9* | COMM domain containing 9 (Commd9), mRNA |  | 2.14 |
| 355 | *Copb2* | Coatomer protein complex, subunit beta 2 (beta prime), mRNA (cDNA clone MGC:6378 IMAGE:3499585) |  | 2.13 |
| 356 | *Cops7a* | COP9 (constitutive photomorphogenic) homolog, subunit 7a (Arabidopsis thaliana), mRNA (cDNA clone MGC:5772 IMAGE:3593979) | 3.3 |  |
| 357 | *Cotl1* | Coactosin-like 1 (Dictyostelium) (Cotl1), mRNA |  | 12.01 |
| 358 | *Cox6b1* | Cytochrome c oxidase, subunit VIb polypeptide 1, mRNA (cDNA clone MGC:35725 IMAGE:4989436) |  | 2.21 |
| 359 | *Cp* | Ceruloplasmin (Cp), transcript variant 2, mRNA | 4.36 |  |
| 360 | *Cplx2* | Complexin 2 (Cplx2), mRNA |  | 2.03 |
| 361 | *Cpt1a* | Liver carnitine palmitoyltransferase I | 3.74 |  |
| 362 | *Cr1l* | Complement component (3b/4b) receptor 1-like, mRNA (cDNA clone MGC:36005 IMAGE:5371665) | 2.85 |  |
| 363 | *Crat* | Carnitine acetyltransferase, mRNA (cDNA clone MGC:6046 IMAGE:3499169) |  | 2.12 |
| 364 | *Creg1* | Cellular repressor of E1A-stimulated genes 1, mRNA (cDNA clone MGC:36084 IMAGE:3670649) | 3.44 |  |
| 365 | *Crip1* | Cysteine-rich protein 1 (intestinal), mRNA (cDNA clone MGC:18577 IMAGE:4206322) | 7.45 |  |
| 366 | *Crnn* | Cornulin (Crnn), mRNA | 6.58 |  |
| 367 | *Cryba4* | Crystallin, beta A4 (Cryba4), mRNA | 7.57 |  |
| 368 | *Crybb1* | Crystallin, beta B1, mRNA (cDNA clone MGC:35800 IMAGE:5358714) | 4.57 |  |
| 369 | *Csf2ra* | Colony stimulating factor 2 receptor, alpha, low-affinity (granulocyte-macrophage), mRNA (cDNA clone MGC:183607 IMAGE:9087607 | 2.67 |  |
| 370 | *Csnk1e* | Casein kinase 1, epsilon, mRNA (cDNA clone MGC:13740 IMAGE:4010696) |  | 2.72 |
| 371 | *Cst6* | Cystatin E/M (Cst6), mRNA | 92.83 | 5.67 |
| 372 | *Cstb* | Cystatin B (Cstb), mRNA | 5.04 | 4.76 |
| 373 | *Ctbp2* | C-terminal binding protein 2, mRNA (cDNA clone MGC:27651 IMAGE:4511826) |  | 3.23 |
| 374 | *Ctnnb1* | Catenin (cadherin associated protein), beta 1, mRNA (cDNA clone IMAGE:3156732) |  | 4.9 |
| 375 | *Ctsb* | Cathepsin B, mRNA (cDNA clone MGC:6211 IMAGE:3500700) | 3.38 |  |
| 376 | *Ctsd* | Cathepsin D, mRNA (cDNA clone IMAGE:4500905) | 3.12 | 2.37 |
| 377 | *Ctse* | Cathepsin E, mRNA (cDNA clone MGC:5924 IMAGE:3601519) | 5.97 |  |
| 378 | *Ctsh* | Cathepsin H, mRNA (cDNA clone MGC:6103 IMAGE:2647229) | 5.95 |  |
| 379 | *Ctsk* | Cathepsin K (Ctsk), mRNA | 7.16 |  |
| 380 | *Ctsl* | Cell-line LXB2 cathepsin L | 4.45 |  |
| 381 | *Ctss* | Cathepsin S, mRNA (cDNA clone MGC:6643 IMAGE:3495719) | 19.81 |  |
| 382 | *Ctsz* | Cathepsin Z, mRNA (cDNA clone MGC:5694 IMAGE:3588457) |  | 2.28 |
| 383 | *Cxadr* | Coxsackie virus and adenovirus receptor, mRNA (cDNA clone MGC:25298 IMAGE:4216727) |  | 2.04 |
| 384 | *Cxcl10* | Chemokine (C-X-C motif) ligand 10, mRNA (cDNA clone MGC:41087 IMAGE:1446589) |  | 4.58 |
| 385 | *Cxcl2* | Chemokine (C-X-C motif) ligand 2 (Cxcl2), mRNA | 8.56 |  |
| 386 | *Cxcl9* | Chemokine (C-X-C motif) ligand 9, mRNA (cDNA clone MGC:6179 IMAGE:3257716) | 5.26 | 7.16 |
| 387 | *Cxcr6* | Chemokine (C-X-C motif) receptor 6 (Cxcr6), mRNA | 2.9 |  |
| 388 | *Cyb5* | Cytochrome b-5, mRNA (cDNA clone MGC:35713 IMAGE:4973059) | 2.55 |  |
| 389 | *Cyb561* | Cytochrome b-561, mRNA (cDNA clone MGC:11696 IMAGE:3963612) | 2.89 |  |
| 390 | *Cyb561d2* | Cytochrome b-561 domain containing 2, mRNA (cDNA clone MGC:25578 IMAGE:3993231) | 2.44 |  |
| 391 | *Cyba* | Cytochrome b-245, alpha polypeptide (Cyba), mRNA | 4.28 |  |
| 392 | Cybb | Cytochrome b-245, beta polypeptide (Cybb), mRNA | 2 |  |
| 393 | *Cyp17a1* | Cytochrome P450, family 17, subfamily a, polypeptide 1 (Cyp17a1), mRNA | 5.85 | 3.01 |
| 394 | *Cyp1b1* | Cytochrome P450, family 1, subfamily b, polypeptide 1 (Cyp1b1), mRNA | 6.68 | 3.53 |
| 395 | *Cyp2b10* | Cyp2b10-like pseudogene, mRNA sequence | 8.77 |  |
| 396 | *Cyp2f2* | Cytochrome P450, family 2, subfamily f, polypeptide 2, mRNA (cDNA clone MGC:29973 IMAGE:5123751) | 12.94 |  |
| 397 | *Cyp2g1* | Olfactory-specific steroid hydroxylase (Cyp2g1) | 6.15 | 3.06 |
| 398 | *Cyp2j9* | Cytochrome P450, family 2, subfamily j, polypeptide 9 (Cyp2j9), mRNA | 2.63 |  |
| 399 | *Cyp4f16* | Cytochrome P450, family 4, subfamily f, polypeptide 16, mRNA (cDNA clone MGC:35789 IMAGE:4982322) |  | 2.1 |
| 400 | *Cyp4f18* | Cytochrome P450, family 4, subfamily f, polypeptide 18, mRNA (cDNA clone MGC:19144 IMAGE:4218398) | 5.88 |  |
| 401 | *Cyp7b1* | Cytochrome P450, family 7, subfamily b, polypeptide 1, mRNA (cDNA clone MGC:46910 IMAGE:5098739) | 2.14 | 4.82 |
| 402 | *Cyth4* | Cytohesin 4, mRNA (cDNA clone IMAGE:5253396) | 5.5 |  |
| 403 | *Cytip* | Cytohesin 1 interacting protein (Cytip), mRNA | 5.74 |  |
| 404 | *D14Ertd668e* | DNA segment, Chr 14, ERATO Doi 668, expressed, mRNA (cDNA clone MGC:29273 IMAGE:5067268) | 5.73 |  |
| 405 | *D4Bwg0951e* | DNA segment, Chr 4, Brigham & Womens Genetics 0951 expressed, mRNA (cDNA clone MGC:40727 IMAGE:5354204) |  | 5.18 |
| 406 | *D730001G18Rik* | RIKEN cDNA D730001G18 gene, mRNA (cDNA clone IMAGE:4187360) | 5.97 |  |
| 407 | *Dapp1* | Dual adaptor for phosphotyrosine and 3-phosphoinositides 1, mRNA (cDNA clone MGC:25226 IMAGE:4486834) | 3.29 |  |
| 408 | *Dbndd2* | Dysbindin (dystrobrevin binding protein 1) domain containing 2, mRNA (cDNA clone MGC:40737 IMAGE:5364949) |  | 2.12 |
| 409 | *Dclk3* | CLICK-I,II-related protein | 3.17 |  |
| 410 | *Ddi2* | DNA-damage inducible protein 2 (Ddi2), mRNA | 2.89 |  |
| 411 | *Ddx3x* | DEAD/H (Asp-Glu-Ala-Asp/His) box polypeptide 3, X-linked (Ddx3x), mRNA |  | 3.56 |
| 412 | *Ddx51* | DEAD (Asp-Glu-Ala-Asp) box polypeptide 51, mRNA (cDNA clone IMAGE:5345562) |  | 2.55 |
| 413 | *Dedd2* | Death effector domain-containing DNA-binding protein 2 (Dedd2) | 2.68 |  |
| 414 | *Defb6* | Defensin beta 6 (Defb6), mRNA | 107.85 | 2.28 |
| 415 | *Dhcr24* | 24-dehydrocholesterol reductase, mRNA (cDNA clone MGC:30547 IMAGE:5054108) | 5.26 | 2.95 |
| 416 | *Dhrs7* | Dehydrogenase/reductase (SDR family) member 7 (Dhrs7), mRNA |  | 2.67 |
| 417 | *Dhx58* | DEXH (Asp-Glu-X-His) box polypeptide 58, mRNA (cDNA clone MGC:35613 IMAGE:2651254) | 2.26 |  |
| 418 | *Dkc1* | Dyskeratosis congenita 1, dyskerin homolog (human) (Dkc1), mRNA |  | 3.76 |
| 419 | *Dlgap5* | Hepatoma up-regulated protein |  | 2.41 |
| 420 | *Dnajb14* | PREDICTED: Mus musculus DnaJ (Hsp40) homolog, subfamily B, member 14 (Dnajb14), mRNA | 2.02 |  |
| 421 | *Dnajc3* | DnaJ (Hsp40) homolog, subfamily C, member 3, mRNA (cDNA clone MGC:6474 IMAGE:2646147) | 2.28 |  |
| 422 | *Dnase1l3* | Deoxyribonuclease 1-like 3, mRNA (cDNA clone MGC:13854 IMAGE:4160709) | 5.78 |  |
| 423 | *Dnmt3l* | DNA (cytosine-5-)-methyltransferase 3-like (Dnmt3l), transcript variant 1, mRNA | 10.02 | 14.18 |
| 424 | *Dock2* | Dedicator of cyto-kinesis 2 (Dock2), mRNA | 2.26 |  |
| 425 | *Dock8* | Dedicator of cytokinesis 8, mRNA (cDNA clone IMAGE:5371956) | 4.32 |  |
| 426 | *Dpep1* | Dipeptidase 1 (renal), mRNA (cDNA clone MGC:6318 IMAGE:2812088) | 4.06 |  |
| 427 | *Dsg3* | Desmoglein 3, mRNA (cDNA clone MGC:164270 IMAGE:40130916) | 3.92 |  |
| 428 | *Dstn* | Destrin (Dstn), mRNA | 2.22 |  |
| 429 | *Dtnbp1* | Dystrobrevin binding protein 1, mRNA (cDNA clone MGC:25855 IMAGE:4195234) |  | 2.76 |
| 430 | *Dtx3l* | Deltex 3-like (Drosophila), mRNA (cDNA clone MGC:169318 IMAGE:8860713) | 2.96 |  |
| 431 | *Duoxa1* | Dual oxidase maturation factor 1 (Duoxa1), mRNA | 6.47 | 2.08 |
| 432 | *Dusp22* | Dual specificity phosphatase 22 (Dusp22), transcript variant 2, mRNA | 2.27 |  |
| 433 | *Dusp5* | Dual specificity phosphatase 5 (Dusp5), mRNA | 2.87 |  |
| 434 | *Dynlt3* | Dynein light chain Tctex-type 3, mRNA (cDNA clone MGC:41029 IMAGE:1332100) | 2.11 |  |
| 435 | *E330016A19Rik* | CDNA clone IMAGE:3603540 | 2.1 |  |
| 436 | *Ebp* | Phenylalkylamine Ca2+ antagonist (emopamil) binding protein, mRNA (cDNA clone MGC:7785 IMAGE:3499265) | 3.03 |  |
| 437 | *Ecm2* | Extracellular matrix protein 2, female organ and adipocyte specific (Ecm2), mRNA | 3.69 |  |
| 438 | *Ednrb* | Endothelin receptor type B, mRNA (cDNA clone MGC:36102 IMAGE:4971909) | 2.45 | 2.01 |
| 439 | *Eea1* | Accn1/Eea1 trans-spliced mRNA sequence | 2.15 |  |
| 440 | *EG620155* | Predicted gene, EG620155, mRNA (cDNA clone MGC:189886 IMAGE:9007386) | 3.11 |  |
| 441 | *EG623286* | PREDICTED: Mus musculus similar to NADH dehydrogenase (ubiquinone) Fe-S protein 6 (LOC675068), misc RNA | 2.12 |  |
| 442 | *EG665033* | Collagen typeVI alpha 5 (Col6a5 gene) | 3.9 |  |
| 443 | *EG668139* | PREDICTED: Mus musculus predicted gene, EG668139 (EG668139), misc RNA | 3.6 |  |
| 444 | *EG668343* | PREDICTED: Mus musculus predicted gene, EG668343 (EG668343), misc RNA | 2.6 |  |
| 445 | *EG668450* | PREDICTED: Mus musculus predicted gene, EG668450 (EG668450), mRNA |  | 2.24 |
| 446 | *Egr1* | Early growth response 1 (Egr1), mRNA | 5.71 |  |
| 447 | *Egr2* | Early growth response 2 (Egr2), mRNA | 11.61 | 3.82 |
| 448 | *Eif1ay* | Eukaryotic translation initiation factor 1A, Y-linked, mRNA (cDNA clone MGC:29011 IMAGE:3156134) |  | 2.16 |
| 449 | *Eif3c* | Eukaryotic translation initiation factor 3, subunit C (Eif3c), mRNA |  | 3.19 |
| 450 | *Eif3h* | Eukaryotic translation initiation factor 3, subunit H (Eif3h), mRNA |  | 2.87 |
| 451 | *Eif4ebp1* | Eukaryotic translation initiation factor 4E binding protein 1, mRNA (cDNA clone MGC:6033 IMAGE:3592595) |  | 2.15 |
| 452 | *Elk3* | ELK3, member of ETS oncogene family, mRNA (cDNA clone MGC:11528 IMAGE:3155850) |  | 2.31 |
| 453 | *Elovl3* | Elongation of very long chain fatty acids (FEN1/Elo2, SUR4/Elo3, yeast)-like 3, mRNA (cDNA clone MGC:18360 IMAGE:4196583) | 29.56 | 7.72 |
| 454 | *Elovl4* | Elongation of very long chain fatty acids (FEN1/Elo2, SUR4/Elo3, yeast)-like 4, mRNA (cDNA clone MGC:47132 IMAGE:4503493) | 7.1 |  |
| 455 | *Elovl6* | Myelination associated SUR4-like protein (Masr) | 3.48 | 2.82 |
| 456 | *Emb* | Embigin, mRNA (cDNA clone MGC:21425 IMAGE:4500922) | 3.43 |  |
| 457 | *Emr1* | Emr1 | 4.72 |  |
| 458 | *Enah* | Enabled homolog (Drosophila) (Enah), transcript variant 1, mRNA |  | 2.47 |
| 459 | *Endod1* | Endonuclease domain containing 1, mRNA (cDNA clone IMAGE:5007670) | 5.58 | 2.64 |
| 460 | *Enpp2* | Ectonucleotide pyrophosphatase/phosphodiesterase 2, mRNA (cDNA clone MGC:6665 IMAGE:3499038) | 2.62 |  |
| 461 | *ENSMUSG00000043472* | PREDICTED: Mus musculus hypothetical LOC630994 (LOC630994), mRNA | 72.93 | 6.36 |
| 462 | *ENSMUSG00000050299* | PREDICTED: Mus musculus similar to fau (LOC100039316), mRNA |  | 2.06 |
| 463 | *Entpd1* | Ectonucleoside triphosphate diphosphohydrolase 1, mRNA (cDNA clone MGC:18369 IMAGE:3674983) | 3.07 |  |
| 464 | *Epcam* | Epithelial cell adhesion molecule, mRNA (cDNA clone MGC:11680 IMAGE:3711069) | 4.63 |  |
| 465 | *Epgn* | Epithelial mitogen (Epgn), mRNA | 11.1 | 2.29 |
| 466 | *Ephx3* | Abhydrolase domain containing 9, mRNA (cDNA clone MGC:175684 IMAGE:40131100) | 4.91 |  |
| 467 | *Eps8* | Epidermal growth factor receptor pathway substrate 8, mRNA (cDNA clone MGC:18488 IMAGE:4240899) | 2.17 |  |
| 468 | *Epsti1* | Epithelial stromal interaction 1 (breast), mRNA (cDNA clone MGC:28189 IMAGE:3988503) | 4.62 |  |
| 469 | *Ereg* | Epiregulin, mRNA (cDNA clone MGC:36144 IMAGE:5325124) | 8.38 |  |
| 470 | *Etf1* | Eukaryotic translation termination factor 1 (Etf1), mRNA | 2.02 |  |
| 471 | *Etv3* | Mitogenic Ets transcriptional suppressor METS | 2.2 |  |
| 472 | *Eya2* | Eyes absent 2 homolog (Drosophila), mRNA (cDNA clone MGC:5883 IMAGE:3497245) | 3.51 | 9.78 |
| 473 | *Ezr* | Ezrin (Ezr), mRNA | 2.01 |  |
| 474 | *F5* | Coagulation factor V (F5), mRNA | 6.46 |  |
| 475 | *Faah* | Fatty acid amide hydrolase, mRNA (cDNA clone MGC:11634 IMAGE:3595191) | 4.73 |  |
| 476 | *Fam102b* | CDNA clone IMAGE:40058901 | 2.68 |  |
| 477 | *Fam115c* | Family with sequence similarity 115, member C (Fam115c), mRNA | 4.99 | 3.03 |
| 478 | *Fam132a* | Family with sequence similarity 132, member A, mRNA (cDNA clone MGC:25716 IMAGE:3966551) |  | 5.14 |
| 479 | *Fam134b* | Family with sequence similarity 134, member B, mRNA (cDNA clone MGC:28735 IMAGE:4460992) | 2.34 | 2.11 |
| 480 | *Fam160b2* | PREDICTED: Mus musculus retinoic acid induced 16 (Rai16), mRNA | 2.08 |  |
| 481 | *Fam167a* | Family with sequence similarity 167, member A, mRNA (cDNA clone MGC:86083 IMAGE:6849398) | 4.43 | 2.72 |
| 482 | *Fam26e* | Family with sequence similarity 26, member E, mRNA (cDNA clone MGC:32360 IMAGE:5031155) | 7.73 |  |
| 483 | *Fam3b* | Family with sequence similarity 3, member B (Fam3b), mRNA | 5.43 | 3.75 |
| 484 | *Fam43a* | CDNA sequence BC022623, mRNA (cDNA clone IMAGE:1178417) | 2.85 | 2.32 |
| 485 | *Fam65c* | Family with sequence similarity 65, member C (Fam65c), mRNA | 2.16 |  |
| 486 | *Fam84a* | Family with sequence similarity 84, member A (Fam84a), mRNA | 4.91 |  |
| 487 | *Fam89a* | Family with sequence similarity 89, member A, mRNA (cDNA clone IMAGE:5006751) | 4.81 |  |
| 488 | *Fars2* | Phenylalanine-tRNA synthetase 2 (mitochondrial) (Fars2), nuclear gene encoding mitochondrial protein, transcript variant 1, m |  | 2.65 |
| 489 | *Fau* | Monoclonal non-specific suppressor factor beta | 2.34 |  |
| 490 | *Fbln7* | Fibulin 7, mRNA (cDNA clone IMAGE:3592288) | 3.23 |  |
| 491 | *Fbrs* | Fibrosin, mRNA (cDNA clone IMAGE:4205868) | 2.08 |  |
| 492 | *Fcer1a* | Fc receptor, IgE, high affinity I, alpha polypeptide (Fcer1a), mRNA | 16.63 |  |
| 493 | *Fcgr1* | Fc receptor, IgG, high affinity I, mRNA (cDNA clone IMAGE:5249690) | 3.34 |  |
| 494 | *Fcgr2b* | Fc receptor, IgG, low affinity IIb, mRNA (cDNA clone MGC:46888 IMAGE:3993915) | 14.01 |  |
| 495 | *Fcgrt* | Fc receptor, IgG, alpha chain transporter, mRNA (cDNA clone MGC:6014 IMAGE:3485649) | 3.81 |  |
| 496 | *Fcrls* | IFGP2 | 4.06 |  |
| 497 | *Fdft1* | Farnesyl diphosphate farnesyl transferase 1 (Fdft1), mRNA |  | 3.34 |
| 498 | *Ffar2* | Free fatty acid receptor 2 (Ffar2), mRNA | 5.38 |  |
| 499 | *Fgd2* | FYVE, RhoGEF and PH domain containing 2, mRNA (cDNA clone MGC:30923 IMAGE:4016697) | 4.81 |  |
| 500 | *Fgd6* | FYVE, RhoGEF and PH domain containing 6, mRNA (cDNA clone IMAGE:3979481) | 3.19 |  |
| 501 | *Fgf1* | Fibroblast growth factor 1, mRNA (cDNA clone MGC:46904 IMAGE:5137246) | 2.72 |  |
| 502 | *Fgl2* | Fibrinogen-like protein 2, mRNA (cDNA clone MGC:19044 IMAGE:4189071) | 4.42 |  |
| 503 | *Fhl2* | Four and a half LIM domains 2, mRNA (cDNA clone MGC:29060 IMAGE:5066565) |  | 5.47 |
| 504 | *Fhl3* | Four and half LIM domain protein 3 (Fhl3) |  | 2.84 |
| 505 | *Fkbp1a* | Strain ILS FK506-binding protein |  | 2.54 |
| 506 | *Flrt3* | Strain C57BL/6xCBA fibronectin leucine rich transmembrane protein 3 (Flrt3) | 2.31 |  |
| 507 | *Fmnl2* | MKIAA1902 protein |  | 2.2 |
| 508 | *Fmo5* | Flavin containing monooxygenase 5, mRNA (cDNA clone MGC:29923 IMAGE:5123876) | 3.77 |  |
| 509 | *Folr2* | Folate receptor 2 (fetal), mRNA (cDNA clone MGC:36142 IMAGE:5102303) | 7.02 |  |
| 510 | *Foxc1* | Forkhead box C1 (Foxc1), mRNA |  | 2.45 |
| 511 | *Frg1* | FSHD region gene 1, mRNA (cDNA clone MGC:5909 IMAGE:3487998) |  | 2.16 |
| 512 | *Fscn1* | Fascin homolog 1, actin bundling protein (Strongylocentrotus purpuratus) (Fscn1), mRNA |  | 4.24 |
| 513 | *Fxyd2* | FXYD domain-containing ion transport regulator 2, mRNA (cDNA clone MGC:25914 IMAGE:4222609) | 4.3 |  |
| 514 | *Fyb* | FYN binding protein FYB-130 | 2.59 |  |
| 515 | *Fzd1* | Frizzled homolog 1 (Drosophila) (Fzd1), mRNA | 2.18 |  |
| 516 | *Fzd6* | Frizzled homolog 6 (Drosophila), mRNA (cDNA clone MGC:14004 IMAGE:3983985) | 2.02 |  |
| 517 | *Gabarapl1* | Gamma-aminobutyric acid (GABA(A)) receptor-associated protein-like 1, mRNA (cDNA clone MGC:30260 IMAGE:3711973) | 2.47 | 2.03 |
| 518 | *Gabra1* | Gamma-aminobutyric acid (GABA-A) receptor, subunit alpha 1 (Gabra1), mRNA | 2.28 |  |
| 519 | *Gal3st4* | Galactose-3-O-sulfotransferase 4 (Gal3st4), mRNA | 2.81 |  |
| 520 | *Galk1* | Galactokinase 1, mRNA (cDNA clone MGC:29019 IMAGE:3490224) |  | 2.56 |
| 521 | *Gapt* | Grb2-binding adaptor, transmembrane, mRNA (cDNA clone MGC:141259 IMAGE:40057167) | 10.68 |  |
| 522 | *Gata6* | Transcription factor GATA-6 (Gata6) |  | 2.59 |
| 523 | *Gbp2* | Guanylate binding protein 2, mRNA (cDNA clone MGC:41173 IMAGE:1230883) | 8.13 |  |
| 524 | *Gch1* | GTP cyclohydrolase 1, mRNA (cDNA clone MGC:12095 IMAGE:3709238) | 6.48 |  |
| 525 | *Gclm* | Glutamate-cysteine ligase, modifier subunit (Gclm), mRNA |  | 2.03 |
| 526 | *Gcnt1* | Glucosaminyl (N-acetyl) transferase 1, core 2, mRNA (cDNA clone MGC:11452 IMAGE:3154671) | 2.87 |  |
| 527 | *Gcnt2* | Glucosaminyl (N-acetyl) transferase 2, I-branching enzyme (Gcnt2), transcript variant 2, mRNA | 2.9 |  |
| 528 | *Gda* | Guanine deaminase [Mus spretus], mRNA sequence | 6.1 | 3.06 |
| 529 | *Ghr* | Growth hormone receptor, mRNA (cDNA clone IMAGE:4976030) | 2.31 |  |
| 530 | *Gjb4* | Gap junction protein, beta 4 (Gjb4), mRNA | 10.81 |  |
| 531 | *Gli2* | GLI-Kruppel family member GLI2, mRNA (cDNA clone IMAGE:4985978) |  | 2.36 |
| 532 | *Glis2* | GLIS family zinc finger 2, mRNA (cDNA clone MGC:36775 IMAGE:5352352) |  | 2.17 |
| 533 | *Glo1* | Glyoxalase 1 (Glo1), transcript variant 1, mRNA |  | 2.05 |
| 534 | *Glul* | Glutamine synthetase (Glul) |  | 2.06 |
| 535 | *Gm438* | Gene model 438, (NCBI) (Gm438), mRNA | 7.32 |  |
| 536 | *Gm885* | CDNA clone MGC:179156 IMAGE:9054148 | 4.9 |  |
| 537 | *Gmps* | Guanine monphosphate synthetase, mRNA (cDNA clone IMAGE:4950527) | 4.09 | 2.04 |
| 538 | *Gna15* | Guanine nucleotide binding protein, alpha 15, mRNA (cDNA clone MGC:18931 IMAGE:3967497) | 6.53 |  |
| 539 | *Golga7b* | Golgi autoantigen, golgin subfamily a, 7B (Golga7b), transcript variant 1, mRNA | 6.95 |  |
| 540 | *Golph3l* | Golgi phosphoprotein 3-like (Golph3l), mRNA | 3.69 |  |
| 541 | *Gpc1* | Glypican 1 (Gpc1), mRNA |  | 3.63 |
| 542 | *Gpnmb* | Glycoprotein (transmembrane) nmb, mRNA (cDNA clone MGC:31121 IMAGE:4164706) | 14.12 |  |
| 543 | *Gpr141* | G protein-coupled receptor 141, mRNA (cDNA clone MGC:159288 IMAGE:40130100) | 2.08 |  |
| 544 | *Gpr160* | G protein-coupled receptor 160 (Gpr160), transcript variant 3, mRNA | 3.96 |  |
| 545 | *Gpr171* | G protein-coupled receptor 171, mRNA (cDNA clone MGC:36313 IMAGE:5068166) | 4.27 |  |
| 546 | *Gpr34* | G protein-coupled receptor 34 (Gpr34), mRNA | 7.89 |  |
| 547 | *Gpr56* | G protein-coupled receptor 56, mRNA (cDNA clone MGC:11582 IMAGE:3709247) | 2.24 |  |
| 548 | *Gprc5d* | G protein-coupled receptor longer splice variant | 37.21 |  |
| 549 | *Gpsm3* | G-protein signalling modulator 3 (AGS3-like, C. elegans) (Gpsm3), mRNA | 2.44 |  |
| 550 | *Gpx2* | Glutathione peroxidase 2 (Gpx2), mRNA | 8.79 | 6.31 |
| 551 | *Gpx5* | Glutathione peroxidase 5 (Gpx5), mRNA | 2.46 |  |
| 552 | *Grhl3* | Grainyhead-like 3 (Drosophila) (Grhl3), mRNA | 3.61 |  |
| 553 | *Gstk1* | Glutathione S-transferase kappa 1 (Gstk1), mRNA |  | 2.32 |
| 554 | *Gstm7* | Glutathione S-transferase, mu 7, mRNA (cDNA clone MGC:30483 IMAGE:4166881) |  | 2.07 |
| 555 | *Gtf2f1* | General transcription factor IIF, polypeptide 1, mRNA (cDNA clone MGC:36237 IMAGE:5026448) |  | 4.38 |
| 556 | *Gtpbp4* | GTP binding protein 4, mRNA (cDNA clone MGC:29311 IMAGE:5007174) |  | 3.66 |
| 557 | *Gzmc* | Granzyme C (Gzmc), mRNA | 26.32 | 20.51 |
| 558 | *H13* | Presenilin-like protein 3 (PSL3 gene) | 2.92 |  |
| 559 | *H2-Aa* | Histocompatibility 2, class II antigen A, alpha, mRNA (cDNA clone MGC:25391 IMAGE:3670758) | 34.65 | 2.08 |
| 560 | *H2-Ab1* | Histocompatibility 2, class II antigen A, beta 1, mRNA (cDNA clone MGC:6297 IMAGE:2651058) | 24.21 | 2.1 |
| 561 | *H2afj* | H2A histone family, member J, mRNA (cDNA clone MGC:36202 IMAGE:5055276) | 2.32 |  |
| 562 | *H2-D1* | Histocompatibility 2, T region locus 3 (H2-T3), mRNA | 7.86 |  |
| 563 | *H2-DMa* | Histocompatibility 2, class II, locus DMa, mRNA (cDNA clone MGC:5743 IMAGE:3591389) | 5.78 |  |
| 564 | *H2-DMb2* | Histocompatibility 2, class II, locus Mb2 (H2-DMb2), mRNA | 5.78 |  |
| 565 | *H2-Eb1* | Histocompatibility 2, class II antigen E beta (H2-Eb1), mRNA | 29.88 |  |
| 566 | *H2-K1* | Histocompatibility 2, K1, K region, mRNA (cDNA clone MGC:7052 IMAGE:3156482) | 8.67 |  |
| 567 | *H2-M2* | Histocompatibility 2, M region locus 2, mRNA (cDNA clone MGC:183587 IMAGE:9087587) | 8.72 | 2.32 |
| 568 | *H2-M3* | Histocompatibility 2, M region locus 3, mRNA (cDNA clone MGC:18978 IMAGE:4007538) | 3.55 |  |
| 569 | *H2-Q7* | Histocompatibility 2, Q region locus 7 (H2-Q7), mRNA | 8.23 |  |
| 570 | *H2-T22* | Histocompatibility 2, T region locus 10, mRNA (cDNA clone MGC:25390 IMAGE:4165944) |  | 5 |
| 571 | *Havcr2* | Hepatitis A virus cellular receptor 2 (Havcr2), mRNA | 3.8 |  |
| 572 | *Hbegf* | Heparin-binding EGF-like growth factor (Hbegf), mRNA | 2.68 | 2 |
| 573 | *Hebp2* | Heme binding protein 2, mRNA (cDNA clone MGC:11934 IMAGE:3599858) | 2.47 |  |
| 574 | *Hectd3* | HECT domain containing 3, mRNA (cDNA clone IMAGE:4981502) | 2.43 |  |
| 575 | *Hfe* | Hemochromatosis protein (Hfe gene), 3 UTR long form | 2.58 |  |
| 576 | *Hhex* | Hematopoietically expressed homeobox (Hhex), mRNA | 2.72 |  |
| 577 | *Hmgb2* | High mobility group box 2, mRNA (cDNA clone MGC:6061 IMAGE:3489780) |  | 2.72 |
| 578 | *Hmha1* | Histocompatibility (minor) HA-1 (Hmha1), transcript variant 1, mRNA | 2.38 |  |
| 579 | *Hmox1* | Heme oxygenase (decycling) 1, mRNA (cDNA clone MGC:18463 IMAGE:4235374) | 3.38 |  |
| 580 | *Hnrnpab* | CArG-binding factor A mRNA, partial cds, alternatively spliced |  | 2.05 |
| 581 | *Homer2* | Homer homolog 2 (Drosophila), mRNA (cDNA clone IMAGE:1395337) | 2.82 |  |
| 582 | *Hoxc9* | Homeo box C9 (Hoxc9), mRNA | 2.72 |  |
| 583 | *Hp* | Haptoglobin (Hp), mRNA | 10.11 |  |
| 584 | *Hr* | Hairless, mRNA (cDNA clone IMAGE:4953487) | 6.61 |  |
| 585 | *Hrh1* | Strain CBA/J histamine receptor H1 |  | 4.55 |
| 586 | *Hsd11b1* | Hydroxysteroid 11-beta dehydrogenase 1 (Hsd11b1), transcript variant 1, mRNA | 5.27 |  |
| 587 | *Hsd17b11* | Hydroxysteroid (17-beta) dehydrogenase 11, mRNA (cDNA clone MGC:28723 IMAGE:4458725) | 2.38 |  |
| 588 | *Hsd17b2* | Hydroxysteroid (17-beta) dehydrogenase 2, mRNA (cDNA clone MGC:13900 IMAGE:4217153) | 3.04 |  |
| 589 | *Hsd3b6* | 3 beta-hydroxysteroid dehydrogenase isomerase VI (Hsd3b6) |  | 4.75 |
| 590 | Hsdl2 | Hypothetical protein (ORF1), clone Telethon(Italy_B41)_Strait00295_FL661-C7 | 2 |  |
| 591 | *Hspa1a* | Heat shock protein (hsp68) mRNA, clone MHS213 | 4.48 |  |
| 592 | *Hspa4l* | Heat shock protein 4 like, mRNA (cDNA clone MGC:14013 IMAGE:4190161) |  | 3.37 |
| 593 | *Htatip2* | HIV-1 tat interactive protein 2, homolog (human), mRNA (cDNA clone MGC:13788 IMAGE:4013057) | 2.49 |  |
| 594 | *Htra1* | HtrA serine peptidase 1, mRNA (cDNA clone MGC:19188 IMAGE:4235912) | 2.98 |  |
| 595 | Iah1 | Isoamyl acetate-hydrolyzing esterase 1 homolog (S. cerevisiae) (Iah1), mRNA | 2 |  |
| 596 | *Icam1* | Intercellular adhesion molecule 1 (Icam1), mRNA | 4.49 | 3.89 |
| 597 | *Ide* | Insulin degrading enzyme (Ide), mRNA | 2.33 |  |
| 598 | *Idh1* | Isocitrate dehydrogenase 1 (NADP+), soluble (Idh1), transcript variant 2, mRNA |  | 2.61 |
| 599 | *Ier3* | Immediate early response 3 (Ier3), mRNA | 3.03 |  |
| 600 | *Ifi203* | Interferon activated gene 203, mRNA (cDNA clone MGC:5842 IMAGE:3257492) | 3.03 |  |
| 601 | *Ifi27l2a* | Interferon stimulated gene 12 (Isg12) | 11.68 | 3.44 |
| 602 | *Ifi30* | Interferon gamma inducible protein 30 (Ifi30), mRNA | 3.82 |  |
| 603 | *Ifi35* | Interferon-induced protein 35 (Ifi35), mRNA | 5.1 | 2.4 |
| 604 | *Ifih1* | Interferon induced with helicase C domain 1 (Ifih1), mRNA | 3.43 |  |
| 605 | *Ifit3* | Interferon-induced protein with tetratricopeptide repeats 3, mRNA (cDNA clone MGC:6081 IMAGE:3487345) | 4.5 |  |
| 606 | *Ifitm3* | Interferon induced transmembrane protein 3 (Ifitm3), mRNA |  | 12.25 |
| 607 | *Igfbp2* | Insulin-like growth factor binding protein 2, mRNA (cDNA clone MGC:14074 IMAGE:2654907) |  | 4.35 |
| 608 | *Igfbp4* | Insulin-like growth factor binding protein 4, mRNA (cDNA clone MGC:29917 IMAGE:5123738) |  | 4.92 |
| 609 | *Igfbp7* | Insulin-like growth factor binding protein 7 (Igfbp7), mRNA | 4.97 |  |
| 610 | *Igsf3* | Immunoglobulin superfamily, member 3 (Igsf3), mRNA |  | 3.8 |
| 611 | *Igtp* | Interferon gamma induced GTPase (Igtp), mRNA |  | 2.92 |
| 612 | *Iigp1* | Interferon inducible GTPase 1 (Iigp1), mRNA | 6.1 | 4.44 |
| 613 | *Il10rb* | C57BL/6 interleukin 10 receptor 2 precursor (Il10r2) | 2.43 |  |
| 614 | *Il16* | Interleukin 16, mRNA (cDNA clone IMAGE:4167410) | 2.5 |  |
| 615 | *Il18r1* | Interleukin 18 receptor 1, mRNA (cDNA clone MGC:25319 IMAGE:4483261) | 4.99 |  |
| 616 | *Il1f5* | Interleukin 1 family, member 5 (delta) (Il1f5), transcript variant 2, mRNA | 6.87 | 3.94 |
| 617 | *Il1rap* | Interleukin 1 receptor accessory protein (Il1rap), transcript variant 2, mRNA | 2.86 |  |
| 618 | *Il1rn* | Interleukin 1 receptor antagonist (Il1rn), transcript variant 1, mRNA | 4.6 |  |
| 619 | *Il27ra* | Interleukin 27 receptor, alpha, mRNA (cDNA clone MGC:41112 IMAGE:1244998) | 3.58 |  |
| 620 | *Il2ra* | Interleukin 2 receptor, alpha chain (Il2ra), mRNA | 3.01 |  |
| 621 | *Il2rg* | Interleukin 2 receptor, gamma chain, mRNA (cDNA clone MGC:25334 IMAGE:4924951) | 3.24 | 2.03 |
| 622 | *Il6ra* | Interleukin 6 receptor, alpha (Il6ra), mRNA | 2.65 |  |
| 623 | *Impdh1* | Inosine 5-phosphate dehydrogenase 1 (Impdh1), mRNA |  | 3.44 |
| 624 | *Inhbb* | Inhibin beta-B subunit | 4.43 | 3.06 |
| 625 | *Iqck* | IQ motif containing K (Iqck), mRNA | 2.1 | 2.86 |
| 626 | *Iqgap1* | IQ motif containing GTPase activating protein 1 (Iqgap1), mRNA | 2.16 |  |
| 627 | *Irak3* | Interleukin-1 receptor-associated kinase M | 3.17 | 3.48 |
| 628 | *Irak4* | Interleukin-1 receptor associated kinase 4 (Irak4) | 2.13 |  |
| 629 | *Irf1* | Interferon regulatory factor 1, mRNA (cDNA clone MGC:6190 IMAGE:3600525) | 2.2 |  |
| 630 | *Irf2* | Interferon regulatory factor 2, mRNA (cDNA clone MGC:6191 IMAGE:3586586) | 2.5 |  |
| 631 | *Irf6* | Interferon regulatory factor 6, mRNA (cDNA clone MGC:5918 IMAGE:3592582) | 4.15 |  |
| 632 | *Irf7* | Interferon regulatory factor 7 (Irf7), mRNA | 4.5 |  |
| 633 | *Irf8* | Interferon regulatory factor 8, mRNA (cDNA clone MGC:6194 IMAGE:3487214) | 3.44 | 2.46 |
| 634 | *Irx3* | Iroquois related homeobox 3 (Drosophila) (Irx3), mRNA |  | 2.32 |
| 635 | *Itga11* | Integrin alpha 11, mRNA (cDNA clone MGC:76390 IMAGE:6842988) | 2.19 |  |
| 636 | *Itga7* | Integrin alpha 7 (Itga7), mRNA |  | 4.5 |
| 637 | *Itgae* | Integrin alpha E, epithelial-associated (Itgae), transcript variant 1, mRNA | 3.92 |  |
| 638 | *Itgam* | Integrin alpha M (Itgam), transcript variant 2, mRNA | 2.94 |  |
| 639 | *Itgax* | Integrin alpha X (Itgax), mRNA | 13.89 |  |
| 640 | *Itgb2* | Integrin beta 2 (Itgb2), mRNA | 4.66 |  |
| 641 | *Itgb6* | Integrin beta 6 (Itgb6), mRNA |  | 2.76 |
| 642 | *Itgbl1* | Integrin, beta-like 1 (Itgbl1), mRNA | 8.41 |  |
| 643 | *Itm2b* | Integral membrane protein 2B, mRNA (cDNA clone MGC:27534 IMAGE:4458918) | 2.89 |  |
| 644 | *Itpk1* | Inositol 1,3,4-triphosphate 5/6 kinase, mRNA (cDNA clone IMAGE:4988501) |  | 3.02 |
| 645 | *Itprip* | MKIAA1754 protein | 2.76 |  |
| 646 | *Iws1* | IWS1 homolog (S. cerevisiae), mRNA (cDNA clone MGC:93016 IMAGE:30639737) |  | 2.12 |
| 647 | *Jtb* | Jumping translocation breakpoint, mRNA (cDNA clone MGC:5746 IMAGE:3582930) | 2.74 |  |
| 648 | *Junb* | Jun-B oncogene, mRNA (cDNA clone MGC:6021 IMAGE:3592704) | 3.13 |  |
| 649 | *Kars* | Lysyl-tRNA synthetase, mRNA (cDNA clone MGC:6923 IMAGE:2811286) |  | 2.17 |
| 650 | *Kcnh1* | Potassium voltage-gated channel, subfamily H (eag-related), member 1 (Kcnh1), transcript variant 1, mRNA | 2.2 | 3.12 |
| 651 | *Kcnmb4* | Potassium large conductance calcium-activated channel, subfamily M, beta member 4 (Kcnmb4), mRNA |  | 3.26 |
| 652 | *Kif26a* | CDNA clone IMAGE:40099548 |  | 3.72 |
| 653 | *Kif26b* | CDNA sequence BC056349, mRNA (cDNA clone IMAGE:30356461) |  | 4.17 |
| 654 | *Kif5b* | Kinesin family member 5B (Kif5b), mRNA |  | 3.58 |
| 655 | *Kit* | Kit oncogene (Kit), transcript variant 2, mRNA |  | 2.63 |
| 656 | *Klf10* | Kruppel-like factor 10, mRNA (cDNA clone MGC:7000 IMAGE:3155247) | 3.26 |  |
| 657 | *Klf3* | Kruppel-like factor 3 (basic) (Klf3), mRNA | 2.09 |  |
| 658 | *Klf4* | Kruppel-like factor 4 (gut), mRNA (cDNA clone MGC:11479 IMAGE:3156339) | 3.33 |  |
| 659 | *Klhl18* | Mus musculus, clone IMAGE:5363746, mRNA | 2.15 |  |
| 660 | *Klk13* | Kallikrein related-peptidase 13 (Klk13), mRNA | 3.88 |  |
| 661 | *Klk6* | Kallikrein 6, mRNA (cDNA clone MGC:36051 IMAGE:4954976) | 337.87 | 16.38 |
| 662 | *Klrb1b* | Killer cell lectin-like receptor subfamily B member 1B (Klrb1b), mRNA | 15.32 |  |
| 663 | *Klrc2* | Natural killer cell protein group 2-C2 | 3.38 |  |
| 664 | *Klrk1* | Killer cell lectin-like receptor subfamily K, member 1 (Klrk1), transcript variant 1, mRNA | 4.72 |  |
| 665 | *Krt25* | Keratin 25 (Krt25), mRNA | 13.14 |  |
| 666 | *Krt26* | Keratin 26, mRNA (cDNA clone MGC:144894 IMAGE:40107596) | 16.04 |  |
| 667 | *Krt31* | Keratin 31 (Krt31), mRNA | 13.41 |  |
| 668 | *Krt33a* | Keratin 33A, mRNA (cDNA clone MGC:35623 IMAGE:4166206) | 35.52 |  |
| 669 | *Krt34* | Keratin 34 (Krt34), mRNA | 18.62 |  |
| 670 | *Krt6a* | Keratin 6A (Krt6a), mRNA | 29.78 |  |
| 671 | *Krt6b* | Keratin 6B (Krt6b), mRNA | 91.24 | 4.75 |
| 672 | *Krt7* | Keratin 7 (Krt7) |  | 3.12 |
| 673 | *Krt71* | Keratin protein K6irs | 14.83 |  |
| 674 | *Krt75* | Cytokeratin KRT2-6HF (Krt2-6hf) | 2.29 |  |
| 675 | *Krt79* | Keratin 79 (Krt79), mRNA | 35.67 | 9.07 |
| 676 | *Krtap13* | Keratin associated protein 13 (Krtap13), mRNA | 21.88 |  |
| 677 | *Krtap1-3* | Keratin associated protein 1-3 (Krtap1-3), mRNA | 23.63 |  |
| 678 | *Krtap14* | Keratin associated protein 14 (Krtap14), mRNA | 8.31 |  |
| 679 | *Krtap15* | Keratin associated protein 15 (Krtap15), mRNA | 20.6 |  |
| 680 | *Krtap16-8* | Keratin associated protein 16-8 (Krtap16-8), mRNA | 12.23 |  |
| 681 | *Krtap2-4* | Keratin associated protein 2-4, mRNA (cDNA clone MGC:157549 IMAGE:40135467) | 7.21 |  |
| 682 | *Krtap3-1* | High-sulfur keratin protein | 45.96 | 2.03 |
| 683 | *Krtap3-3* | Keratin associated protein 3-3 (Krtap3-3), mRNA | 46.42 | 3.02 |
| 684 | *Krtap4-16* | Keratin associated protein 4-16, mRNA (cDNA clone MGC:28878 IMAGE:4910141) | 13 |  |
| 685 | *Krtap4-6* | Keratin associated protein 4-6 (Krtap4-6), mRNA | 11.83 |  |
| 686 | *Krtap6-2* | Keratin associated protein 6-2 (Krtap6-2), mRNA | 12.42 |  |
| 687 | *Krtap7-1* | Keratin associated protein 7-1 (Krtap7-1), mRNA | 7.47 |  |
| 688 | *Lair1* | Leukocyte-associated immunoglobulin-like receptor (Lair) | 2.51 |  |
| 689 | *Lama4* | Laminin, alpha 4, mRNA (cDNA clone IMAGE:3490870) |  | 2.96 |
| 690 | *Lamp2* | Lysosomal-associated membrane protein 2 (Lamp2), transcript variant 2, mRNA | 2.02 |  |
| 691 | *Larp2* | La ribonucleoprotein domain family, member 2, mRNA (cDNA clone MGC:38253 IMAGE:5324328) | 2.17 |  |
| 692 | *Larp5* | La ribonucleoprotein domain family, member 5, mRNA (cDNA clone MGC:78318 IMAGE:6506267) |  | 4.05 |
| 693 | *Lass4* | TRH1 | 6.89 | 4.66 |
| 694 | *Layn* | Layilin, mRNA (cDNA clone MGC:161835 IMAGE:40140447) | 3.86 |  |
| 695 | *Lbh* | Limb-bud and heart (Lbh), mRNA |  | 3.53 |
| 696 | *Lce3a* | Late cornified envelope 3A (Lce3a), mRNA | 97.68 |  |
| 697 | *Lcp1* | Lymphocyte cytosolic protein 1, mRNA (cDNA clone MGC:30234 IMAGE:3486993) | 4.89 |  |
| 698 | *Ldha* | Lactate dehydrogenase A, mRNA (cDNA clone IMAGE:3491850) |  | 3.1 |
| 699 | *Lgals3bp* | Lectin, galactoside-binding, soluble, 3 binding protein (Lgals3bp), mRNA | 3.44 |  |
| 700 | *Lgi4* | LGI1-like protein 3 (Lgil3 gene) | 4.91 |  |
| 701 | *Lgr5* | Leucine rich repeat containing G protein coupled receptor 5 (Lgr5), mRNA | 2.18 | 2.35 |
| 702 | *Lilrb3* | Paired-Ig-like receptor A6 (Pira6), transcript variant 2, mRNA | 16.43 |  |
| 703 | *Lingo1* | Putative transmembrane protein mV/BamHI#3 |  | 7.44 |
| 704 | *Litaf* | LPS-induced TN factor, mRNA (cDNA clone MGC:6569 IMAGE:2812674) | 2.14 |  |
| 705 | *Lmo1* | LIM domain only 1 (Lmo1), mRNA |  | 3.89 |
| 706 | *LOC100040214* | Glycine tyrosine-rich hair keratin protein | 6.97 |  |
| 707 | *LOC100040287* | PREDICTED: Mus musculus similar to ubiquinol-cytochrome c reductase subunit (LOC100040287), mRNA |  | 2.15 |
| 708 | *LOC100042514* | PREDICTED: Mus musculus hypothetical protein LOC100042514 (LOC100042514), mRNA | 42.8 | 28.24 |
| 709 | *Lpar1* | Lysophosphatidic acid receptor 1, mRNA (cDNA clone MGC:29102 IMAGE:5009055) |  | 3 |
| 710 | *Lpcat3* | CDNA fis, clone TRACH2004292,highly similar to Human C3f mRNA | 2.07 |  |
| 711 | *Lpgat1* | Lysophosphatidylglycerol acyltransferase 1, mRNA (cDNA clone IMAGE:5362498) | 2.01 |  |
| 712 | *Lpin1* | Lipin 1-b (Lpin1) |  | 2.19 |
| 713 | *Lrg1* | Leucine-rich alpha-2-glycoprotein 1, mRNA (cDNA clone MGC:37928 IMAGE:5123909) | 10.5 |  |
| 714 | *Lrig1* | Leucine-rich repeats and immunoglobulin-like domains 1 (Lrig1), mRNA |  | 2.15 |
| 715 | *Lrrc1* | Leucine rich repeat containing 1, mRNA (cDNA clone IMAGE:4167448) | 2.15 |  |
| 716 | *Lrrc25* | Leucine rich repeat containing 25 (Lrrc25), mRNA | 4.08 |  |
| 717 | *Lrrc59* | Leucine rich repeat containing 59 (Lrrc59), mRNA |  | 2.1 |
| 718 | *Lrsam1* | Leucine rich repeat and sterile alpha motif containing 1 (Lrsam1), mRNA | 3.23 |  |
| 719 | *Lsp1* | Lymphocyte specific 1, mRNA (cDNA clone MGC:6047 IMAGE:3488528) | 2.15 |  |
| 720 | *Lss* | Lanosterol synthase (Lss), mRNA | 2.48 |  |
| 721 | *Ltb* | Lymphotoxin B (Ltb), mRNA |  | 2.57 |
| 722 | *Ly6a* | Lymphocyte antigen 6 complex, locus A, mRNA (cDNA clone MGC:6188 IMAGE:3486025) | 9.4 |  |
| 723 | *Ly6c2* | Lymphocyte differentiation antigen Ly-6C.2 | 8.37 |  |
| 724 | *Ly6g5b* | Lymphocyte antigen 6 complex, locus G5B (Ly6g5b), mRNA | 2.9 |  |
| 725 | *Ly75* | Strain C57BL/6 DEC-205 (Ly75) | 2.36 |  |
| 726 | *Ly86* | Lymphocyte antigen 86 (Ly86), mRNA | 4.03 |  |
| 727 | *Lyl1* | Lymphoblastomic leukemia 1, mRNA (cDNA clone MGC:11650 IMAGE:3598911) | 2.29 |  |
| 728 | *Lyz1* | Lysozyme 1 (Lyz1), mRNA | 25.73 |  |
| 729 | *Macc1* | PREDICTED: Mus musculus RIKEN cDNA 4732474O15 gene (4732474O15Rik), mRNA | 3.4 |  |
| 730 | *Mal2* | Mal, T-cell differentiation protein 2, mRNA (cDNA clone IMAGE:4022528) | 3.52 | 2.09 |
| 731 | *Man1a* | Mannosidase 1, alpha, mRNA (cDNA clone MGC:18448 IMAGE:4223319) | 3.75 |  |
| 732 | *Man1c1* | Mannosidase, alpha, class 1C, member 1, mRNA (cDNA clone IMAGE:5373083) | 2.38 |  |
| 733 | *Map1lc3b* | CDNA clone IMAGE:5149237 | 2.13 |  |
| 734 | *Map3k6* | Mitogen-activated protein kinase kinase kinase 6 (Map3k6), mRNA |  | 3.2 |
| 735 | *Map4k1* | Mitogen-activated protein kinase kinase kinase kinase 1, mRNA (cDNA clone IMAGE:5356534) | 2.37 |  |
| 736 | *Marcks* | Myristoylated alanine rich protein kinase C substrate (Marcks), mRNA |  | 4.54 |
| 737 | *Mark1* | MAP/microtubule affinity-regulating kinase 1 (Mark1), mRNA |  | 3.65 |
| 738 | *Matk* | Megakaryocyte-associated tyrosine kinase (Matk), mRNA | 4.85 |  |
| 739 | *Mbnl1* | Muscleblind-like 1 (Drosophila), mRNA (cDNA clone IMAGE:1511990) | 2.79 |  |
| 740 | *Mbnl2* | MKIAA4072 protein |  | 2.45 |
| 741 | Mcfd2 | Multiple coagulation factor deficiency 2 (Mcfd2), transcript variant 2, mRNA | 2 |  |
| 742 | *mCG_21548* | Nedd4-binding brain specific protein BEAN | 3.36 |  |
| 743 | *Mcoln2* | Mucolipin 2 (Mcoln2) | 2.03 |  |
| 744 | *Med10* | DNA segment, Chr 13, Wayne State University 50, expressed, mRNA (cDNA clone IMAGE:6409750) | 2.24 |  |
| 745 | *Med11* | Mediator of RNA polymerase II transcription, subunit 11 homolog (S. cerevisiae), mRNA (cDNA clone MGC:35762 IMAGE:4238297) | 6.7 | 2.64 |
| 746 | *Melk* | Maternal embryonic leucine zipper kinase (Melk), mRNA |  | 2.95 |
| 747 | *Mfap3l* | Microfibrillar-associated protein 3-like, mRNA (cDNA clone MGC:106286 IMAGE:5025281) | 2.99 |  |
| 748 | *Mfap4* | Microfibrillar-associated protein 4 (Mfap4), mRNA | 2.78 |  |
| 749 | *Mfge8* | Milk fat globule-EGF factor 8 protein, mRNA (cDNA clone MGC:6771 IMAGE:3601565) |  | 5.47 |
| 750 | *Mfsd6* | RIKEN cDNA 2210010L05 gene, mRNA (cDNA clone IMAGE:3595801) | 3.06 |  |
| 751 | *Mgl2* | Macrophage galactose-type C-type lectin 2 (Mgl2) | 8.29 |  |
| 752 | *Mgll* | Monoglyceride lipase (Mgll gene), transcript 2 | 4.36 | 2.83 |
| 753 | *Mgst1* | Microsomal glutathione S-transferase 1, mRNA (cDNA clone MGC:6549 IMAGE:2655738) | 4.87 | 4.58 |
| 754 | *Mill1* | MHC I like leukocyte 1 (Mill1), mRNA | 17.73 | 2.1 |
| 755 | *Mitd1* | MIT, microtubule interacting and transport, domain containing 1, mRNA (cDNA clone MGC:28063 IMAGE:3709157) | 2.64 |  |
| 756 | *Mlana* | Melan-A (Mlana), mRNA | 5.91 |  |
| 757 | *Mlkl* | Mixed lineage kinase domain-like, mRNA (cDNA clone MGC:38580 IMAGE:5354568) | 6.83 | 2.65 |
| 758 | *Mmp11* | Matrix metallopeptidase 11 (Mmp11), mRNA | 2.37 |  |
| 759 | *Mmp12* | Matrix metallopeptidase 12, mRNA (cDNA clone MGC:29351 IMAGE:5037674) | 64.36 | 3.89 |
| 760 | *Mmp13* | Matrix metallopeptidase 13 (Mmp13), mRNA | 5.46 |  |
| 761 | *Mmp2* | Matrix metallopeptidase 2 (Mmp2), mRNA | 3.02 | 2.21 |
| 762 | *Mmp25* | Matrix metallopeptidase 25, mRNA (cDNA clone MGC:132821 IMAGE:40059601) | 2.02 |  |
| 763 | *Mmp3* | Matrix metallopeptidase 3, mRNA (cDNA clone MGC:11554 IMAGE:3962288) | 21.83 |  |
| 764 | *Mmp8* | Matrix metallopeptidase 8 (Mmp8), mRNA | 4.71 |  |
| 765 | *Mmp9* | Matrix metallopeptidase 9 (Mmp9), mRNA | 3.28 |  |
| 766 | *Mobkl1a* | MOB1, Mps One Binder kinase activator-like 1A (yeast), mRNA (cDNA clone MGC:129511 IMAGE:40050503) |  | 2.17 |
| 767 | *Mpa2l* | Guanylate-binding protein 10 (Gbp10) | 3.42 |  |
| 768 | *Mpeg1* | MPS1 gene and mRNA, 3end | 9.78 |  |
| 769 | *Mphosph10* | M-phase phosphoprotein 10 (U3 small nucleolar ribonucleoprotein), mRNA (cDNA clone IMAGE:4504841) |  | 3.29 |
| 770 | *Mpi* | Mannose phosphate isomerase, mRNA (cDNA clone MGC:6520 IMAGE:2650791) |  | 2.23 |
| 771 | *Mpp6* | Membrane protein, palmitoylated 6 (MAGUK p55 subfamily member 6) (Mpp6), mRNA |  | 2.03 |
| 772 | *Mreg* | Dilute suppressor protein (Dsu) | 2.66 |  |
| 773 | *Mrfap1* | Morf4 family associated protein 1, mRNA (cDNA clone MGC:7196 IMAGE:3482091) |  | 2.17 |
| 774 | *Mrgprg* | MAS-related GPR, member G, mRNA (cDNA clone MGC:117497 IMAGE:30522259) | 4.19 |  |
| 775 | *Mrgprx2* | MAS-related GPR, member X2, mRNA (cDNA clone MGC:176186 IMAGE:9055837) |  | 3.04 |
| 776 | *Mrpl36* | Mitochondrial ribosomal protein L36 (Mrpl36), nuclear gene encoding mitochondrial protein, mRNA | 2.16 |  |
| 777 | *Mrpl39* | Mitochondrial ribosomal protein L39, mRNA (cDNA clone MGC:27731 IMAGE:2645316) |  | 2.18 |
| 778 | *Mrpl48* | Mitochondrial ribosomal protein L48, mRNA (cDNA clone MGC:47257 IMAGE:4168034) |  | 2.29 |
| 779 | *Mrps22* | Mitochondrial ribosomal protein S22, mRNA (cDNA clone IMAGE:3588473) |  | 2.21 |
| 780 | *Mrps35* | Mitochondrial ribosomal protein S35 (Mrps35), nuclear gene encoding mitochondrial protein, mRNA |  | 2.52 |
| 781 | *Ms4a4c* | Membrane-spanning 4-domains, subfamily A, member 4C, mRNA (cDNA clone MGC:41005 IMAGE:1383416) | 5.84 |  |
| 782 | *Ms4a4d* | MS4A4D protein | 3.78 |  |
| 783 | *Ms4a7* | Membrane-spanning 4-domains, subfamily A, member 7, mRNA (cDNA clone MGC:36243 IMAGE:5035752) | 15.69 |  |
| 784 | *Msn* | Moesin (Msn), mRNA |  | 2.63 |
| 785 | *Msrb3* | Methionine sulfoxide reductase B3, mRNA (cDNA clone MGC:106192 IMAGE:6310686) |  | 2.12 |
| 786 | *Mt2* | Metallothionein 2, mRNA (cDNA clone MGC:19383 IMAGE:2651471) | 5.49 |  |
| 787 | *Mta1* | MTA1 (Mta1) | 2.2 |  |
| 788 | *Mvp* | Major vault protein (Mvp), mRNA |  | 5.37 |
| 789 | *Myc* | Myelocytomatosis oncogene, mRNA (cDNA clone IMAGE:3962047) | 2.48 |  |
| 790 | *Mycn* | V-myc myelocytomatosis viral related oncogene, neuroblastoma derived (avian), mRNA (cDNA clone MGC:6240 IMAGE:3495446) |  | 3.43 |
| 791 | *Myd88* | Myeloid differentiation primary response gene 88, mRNA (cDNA clone MGC:5902 IMAGE:3482246) | 3.47 |  |
| 792 | *Myo1g* | Myosin IG, mRNA (cDNA clone IMAGE:1327573) | 4.92 |  |
| 793 | *Myof* | Myoferlin, mRNA (cDNA clone IMAGE:5324940) | 2.46 |  |
| 794 | *Nanos3* | Nanos homolog 3 (Drosophila), mRNA (cDNA clone MGC:141147 IMAGE:40055381) |  | 2.67 |
| 795 | *Nckap1l* | NCK associated protein 1 like (Nckap1l), mRNA | 2.99 |  |
| 796 | *Ncl* | Nucleolin, mRNA (cDNA clone MGC:6363 IMAGE:3495665) |  | 2.01 |
| 797 | Ndel1 | Nuclear distribution gene E-like homolog 1 (A. nidulans), mRNA (cDNA clone MGC:29336 IMAGE:5029832) | 2 |  |
| 798 | *Ndrg2* | N-myc downstream regulated gene 2, mRNA (cDNA clone MGC:13746 IMAGE:4211421) |  | 3.16 |
| 799 | *Ndufa1* | NADH dehydrogenase (ubiquinone) 1 alpha subcomplex, 1, mRNA (cDNA clone MGC:35935 IMAGE:5031206) |  | 2.46 |
| 800 | *Ndufa4* | Mlrq-like protein | 2.38 |  |
| 801 | *Ndufa6* | NADH dehydrogenase (ubiquinone) 1 alpha subcomplex, 6 (B14) (Ndufa6), nuclear gene encoding mitochondrial protein, mRNA | 2.22 |  |
| 802 | *Ndufaf2* | CDNA clone IMAGE:9053264 |  | 2.35 |
| 803 | *Ndufb10* | NADH dehydrogenase (ubiquinone) 1 beta subcomplex, 10, mRNA (cDNA clone MGC:36216 IMAGE:4219237) |  | 3.96 |
| 804 | Ndufb7 | NADH dehydrogenase (ubiquinone) 1 beta subcomplex, 7, mRNA (cDNA clone MGC:35706 IMAGE:4913656) |  | 2 |
| 805 | *Ndufv2* | NADH dehydrogenase (ubiquinone) flavoprotein 2, mRNA (cDNA clone MGC:32165 IMAGE:5003524) |  | 2.52 |
| 806 | *Nedd9* | Neural precursor cell expressed, developmentally down-regulated gene 9, mRNA (cDNA clone IMAGE:3499250) |  | 3.21 |
| 807 | *Nek7* | NIMA (never in mitosis gene a)-related expressed kinase 7, mRNA (cDNA clone MGC:46827 IMAGE:3994871) |  | 2.21 |
| 808 | *Nelf* | Nasal embryonic LHRH factor, mRNA (cDNA clone MGC:7230 IMAGE:3483536) |  | 3.39 |
| 809 | *Nfam1* | NFAT activation molecule 1 (Nfam1) | 2.1 |  |
| 810 | *Nfkbie* | Nuclear factor of kappa light polypeptide gene enhancer in B-cells inhibitor, epsilon, mRNA (cDNA clone MGC:31667 IMAGE:49099 | 3.51 |  |
| 811 | *Nfkbiz* | INAP mRNA for IL-1 inducible nuclear ankyrin-repeat protein | 2.37 |  |
| 812 | *Ninj1* | Ninjurin 1 (Ninj1), mRNA | 2.53 |  |
| 813 | *Nod2* | Nucleotide-binding oligomerization domain containing 2 (Nod2), mRNA | 4.38 |  |
| 814 | *Nop14* | NOP14 nucleolar protein homolog (yeast), mRNA (cDNA clone MGC:32275 IMAGE:5012070) |  | 2.23 |
| 815 | *Nox4* | Superoxide-generating NADPH oxidase 4 (Nox4) | 2.35 |  |
| 816 | *Nqo1* | NAD(P)H dehydrogenase, quinone 1 (Nqo1), mRNA | 2.4 |  |
| 817 | *Nr2c2ap* | Nuclear receptor 2C2-associated protein (Nr2c2ap), transcript variant 2, mRNA | 2.37 |  |
| 818 | *Nrarp* | Notch-regulated ankyrin repeat protein (Nrarp), mRNA | 2.01 |  |
| 819 | *Nrbp2* | Nuclear receptor binding protein 2 (Nrbp2), mRNA | 2.19 |  |
| 820 | Nup43 | Nucleoporin 43 (Nup43), mRNA | 2 |  |
| 821 | *Oasl2* | 2-5 oligoadenylate synthetase-like 2, mRNA (cDNA clone MGC:6269 IMAGE:2646375) | 2.43 | 3.55 |
| 822 | *Ociad2* | OCIA domain containing 2 (Ociad2), mRNA | 2.73 |  |
| 823 | *Ocln* | Occludin (Ocln), mRNA | 2.23 |  |
| 824 | *Odz1* | Odd Oz/ten-m homolog 1 (Drosophila) (Odz1), mRNA | 2.21 |  |
| 825 | *Olah* | Oleoyl-ACP hydrolase (Olah), mRNA | 18.44 | 13.63 |
| 826 | *Olfr1495* | Olfactory receptor 1495 (Olfr1495), mRNA | 2.39 |  |
| 827 | *Olfr96* | Olfactory receptor 96, mRNA (cDNA clone MGC:155889 IMAGE:40129575) | 4.42 |  |
| 828 | *Omd* | Osteomodulin (Omd), mRNA | 4.25 |  |
| 829 | *Orai1* | ORAI calcium release-activated calcium modulator 1, mRNA (cDNA clone MGC:28390 IMAGE:4022503) | 3.26 |  |
| 830 | *Osbpl3* | Oxysterol binding protein-like 3 (Osbpl3), mRNA | 4.35 |  |
| 831 | *Osbpl9* | Nardilysin, N-arginine dibasic convertase, NRD convertase 1, mRNA (cDNA clone IMAGE:5344071) |  | 2.5 |
| 832 | *Osmr* | Oncostatin M receptor (Osmr), mRNA | 4.07 |  |
| 833 | *Otof* | Otoferlin (Otof), transcript variant 2, mRNA | 2.83 |  |
| 834 | *OTTMUSG00000000265* | PREDICTED: Mus musculus similar to ZH10 protein (LOC622491), mRNA | 2.22 |  |
| 835 | *OTTMUSG00000000712* | Predicted gene, OTTMUSG00000000712, mRNA (cDNA clone MGC:182827 IMAGE:9087441) | 157.49 |  |
| 836 | *OTTMUSG00000000971* | Activated macrophage/microglia WAP domain protein precursor (Amwap) | 33.89 |  |
| 837 | *OTTMUSG00000002191* | PREDICTED: Mus musculus predicted gene, OTTMUSG00000002191 (OTTMUSG00000002191), mRNA | 20 |  |
| 838 | *OTTMUSG00000004599* | PREDICTED: Mus musculus similar to heterogeneous nuclear ribonucleoprotein A3, transcript variant 16 (LOC545592), mRNA |  | 3.39 |
| 839 | *OTTMUSG00000007452* | PREDICTED: Mus musculus similar to adipose differentiation related protein (LOC100040388), mRNA | 46.26 |  |
| 840 | *OTTMUSG00000007480* | PREDICTED: Mus musculus similar to major urinary protein 1 (LOC100039206), mRNA | 77.77 |  |
| 841 | *OTTMUSG00000010750* | Predicted gene, OTTMUSG00000010750 (OTTMUSG00000010750), mRNA | 11.25 | 4.14 |
| 842 | *OTTMUSG00000010778* | PREDICTED: Mus musculus similar to LSM7 homolog, U6 small nuclear RNA associated (LOC100040219), mRNA | 2.54 |  |
| 843 | *P2ry10* | Purinergic receptor P2Y, G-protein coupled 10, mRNA (cDNA clone MGC:60872 IMAGE:30072257) | 3.97 |  |
| 844 | *P2ry14* | Purinergic receptor P2Y, G-protein coupled, 14, mRNA (cDNA clone MGC:38677 IMAGE:5356997) | 7.99 |  |
| 845 | *P2ry6* | Pyrimidinergic receptor P2Y, G-protein coupled, 6, mRNA (cDNA clone MGC:28142 IMAGE:3982042) | 3.49 |  |
| 846 | *Padi4* | Peptidyl arginine deiminase, type IV (Padi4), mRNA | 11.16 |  |
| 847 | *Palmd* | Palmdelphin (PALMD gene) |  | 4.14 |
| 848 | *Parp9* | Poly (ADP-ribose) polymerase family, member 9, mRNA (cDNA clone IMAGE:3595501) |  | 2.75 |
| 849 | *Parvg* | Parvin, gamma, mRNA (cDNA clone MGC:18790 IMAGE:4190510) | 4.38 |  |
| 850 | *Pbk* | PDZ binding kinase, mRNA (cDNA clone MGC:28045 IMAGE:3673060) |  | 2.09 |
| 851 | *Pctp* | Phosphatidylcholine transfer protein (Pctp) | 7.18 | 2.04 |
| 852 | *Pdcd6* | Programmed cell death 6 (Pdcd6), mRNA | 3.02 |  |
| 853 | *Pdia6* | Protein disulfide isomerase associated 6, mRNA (cDNA clone MGC:6472 IMAGE:2645183) |  | 2.1 |
| 854 | *Pdk4* | Pyruvate dehydrogenase kinase, isoenzyme 4, mRNA (cDNA clone MGC:13840 IMAGE:4188580) |  | 3.18 |
| 855 | *Pdlim1* | PDZ and LIM domain 1 (elfin), mRNA (cDNA clone MGC:5634 IMAGE:3588132) |  | 2.34 |
| 856 | *Pdlim2* | PDZ and LIM domain 2 (Pdlim2), mRNA | 3.59 |  |
| 857 | *Pdlim4* | PDZ and LIM domain 4, mRNA (cDNA clone MGC:41095 IMAGE:5144166) |  | 2.49 |
| 858 | *Pdss1* | Prenyl (solanesyl) diphosphate synthase, subunit 1, mRNA (cDNA clone MGC:28441 IMAGE:4039464) |  | 2.7 |
| 859 | *Pdzk1* | Hydrophilic CFTR-binding protein CAP70 (Cap70) | 4.93 | 3.44 |
| 860 | *Pecr* | Peroxisomal trans-2-enoyl-CoA reductase, mRNA (cDNA clone MGC:19210 IMAGE:4238679) |  | 2.09 |
| 861 | *Peli3* | Pellino 3, mRNA (cDNA clone MGC:30417 IMAGE:5037308) | 2.22 |  |
| 862 | *Pex26* | Peroxisomal biogenesis factor 26 (Pex26), mRNA | 2.05 |  |
| 863 | Pfdn1 | Prefoldin 1, mRNA (cDNA clone MGC:19010 IMAGE:4020579) | 2 |  |
| 864 | *Pfkl* | Phosphofructokinase, liver, B-type, mRNA (cDNA clone MGC:28316 IMAGE:4014395) |  | 2.7 |
| 865 | *Pfkp* | Phosphofructokinase, platelet, mRNA (cDNA clone IMAGE:4507452) | 3.59 |  |
| 866 | *Pgcp* | Plasma glutamate carboxypeptidase, mRNA (cDNA clone MGC:46857 IMAGE:4989536) | 3.47 |  |
| 867 | *Pgd* | Phosphogluconate dehydrogenase, mRNA (cDNA clone MGC:18955 IMAGE:3984654) |  | 2.63 |
| 868 | *Phax* | Phosphorylated adaptor for RNA export (Phax), mRNA |  | 2.2 |
| 869 | *Phlda1* | Pleckstrin homology-like domain, family A, member 1, mRNA (cDNA clone MGC:11486 IMAGE:3153799) |  | 3.7 |
| 870 | *Phyh* | Phytanoyl-CoA hydroxylase, mRNA (cDNA clone MGC:5835 IMAGE:3489939) | 3.49 |  |
| 871 | *Pias1* | Protein inhibitor of activated STAT 1 (Pias1), mRNA |  | 2.13 |
| 872 | *Pigg* | Phosphatidylinositol glycan anchor biosynthesis, class G (Pigg), mRNA |  | 2.07 |
| 873 | *Pik3cb* | Phosphatidylinositol 3-kinase, catalytic, beta polypeptide, mRNA (cDNA clone MGC:150132 IMAGE:40110218) | 2.59 |  |
| 874 | *Pilrb1* | Paired immunoglobin-like type 2 receptor beta 1 (Pilrb1), mRNA | 7.29 |  |
| 875 | *Pip4k2a* | Phosphatidylinositol-5-phosphate 4-kinase, type II, alpha, mRNA (cDNA clone MGC:18357 IMAGE:3672732) | 2.38 |  |
| 876 | *Pira3* | Paired-Ig-like receptor A3 (Pira3), mRNA | 5.79 |  |
| 877 | *Pkdcc* | Pkdcc mRNA for putative protein kinase |  | 10.38 |
| 878 | *Pkib* | Protein kinase inhibitor beta, cAMP dependent, testis specific (Pkib), transcript variant 1, mRNA | 7.01 |  |
| 879 | *Pkm2* | Pyruvate kinase, muscle, mRNA (cDNA clone MGC:11908 IMAGE:3598842) |  | 3.93 |
| 880 | *Pla2g12a* | Phospholipase A2, group XIIA, mRNA (cDNA clone MGC:25458 IMAGE:4456431) |  | 2.46 |
| 881 | *Pla2g4a* | Phospholipase A2, group IVA (cytosolic, calcium-dependent), mRNA (cDNA clone MGC:6168 IMAGE:3485483) | 2.94 |  |
| 882 | *Pla2r1* | Phospholipase A2 receptor 1 (Pla2r1), mRNA | 2.37 |  |
| 883 | *Plaur* | Plasminogen activator, urokinase receptor, mRNA (cDNA clone IMAGE:3158012) | 2.25 |  |
| 884 | *Plbd1* | RIKEN cDNA 1100001H23 gene, mRNA (cDNA clone MGC:30303 IMAGE:5133074) | 3.81 |  |
| 885 | *Pld3* | Phospholipase D family, member 3 (Pld3), mRNA | 3.12 |  |
| 886 | *Pld4* | Phospholipase D family, member 4, mRNA (cDNA clone MGC:68329 IMAGE:6442038) | 5.64 |  |
| 887 | *Plek2* | Pleckstrin 2, mRNA (cDNA clone MGC:18708 IMAGE:4208341) | 3.72 |  |
| 888 | *Plekha2* | PH domain-containing adaptor PHAD47 (Phad47) | 2.2 |  |
| 889 | *Pls3* | Plastin 3 (T-isoform) (Pls3), mRNA |  | 2.37 |
| 890 | *Plxdc2* | Plexin domain containing 2 (Plxdc2), mRNA | 2.05 |  |
| 891 | *Pmepa1* | Prostate transmembrane protein, androgen induced 1, mRNA (cDNA clone IMAGE:5038092) | 3.05 | 5.7 |
| 892 | *Pmm2* | Phosphomannomutase 2, mRNA (cDNA clone IMAGE:5352533) |  | 2.46 |
| 893 | *Pole3* | Polymerase (DNA directed), epsilon 3 (p17 subunit), mRNA (cDNA clone MGC:30997 IMAGE:5253060) | 2.12 |  |
| 894 | *Pop5* | Processing of precursor 5, ribonuclease P/MRP family (S. cerevisiae) (Pop5), mRNA | 2.5 |  |
| 895 | *Ppapdc2* | Phosphatidic acid phosphatase type 2 domain containing 2 (Ppapdc2), mRNA | 2.55 |  |
| 896 | *Ppard* | Peroxisome proliferator-activated receptor |  | 2.39 |
| 897 | *Ppargc1b* | Peroxisome proliferative activated receptor, gamma, coactivator 1 beta (Ppargc1b), mRNA |  | 3.2 |
| 898 | *Ppib* | Peptidylprolyl isomerase B, mRNA (cDNA clone MGC:6241 IMAGE:3483267) |  | 6.15 |
| 899 | *Ppif* | Peptidylprolyl isomerase F (cyclophilin F) (Ppif), nuclear gene encoding mitochondrial protein, mRNA |  | 2.17 |
| 900 | *Ppig* | Peptidyl-prolyl isomerase G (cyclophilin G), mRNA (cDNA clone MGC:183605 IMAGE:9087605) |  | 2.54 |
| 901 | *Ppp1r13l* | Protein phosphatase 1, regulatory (inhibitor) subunit 13 like (Ppp1r13l), mRNA | 3.36 |  |
| 902 | *Ppp1r14c* | Protein phosphatase 1, regulatory (inhibitor) subunit 14c (Ppp1r14c), mRNA |  | 2.19 |
| 903 | *Ppp2r5a* | Protein phosphatase 2, regulatory subunit B (B56), alpha isoform (Ppp2r5a), mRNA | 2.78 | 2.17 |
| 904 | *Pqlc1* | PQ loop repeat containing 1, mRNA (cDNA clone MGC:36695 IMAGE:3487991) | 5.05 | 2.02 |
| 905 | *Prex1* | Phosphatidylinositol-3,4,5-trisphosphate-dependent Rac exchange factor 1, mRNA (cDNA clone MGC:183987 IMAGE:9087987) | 2.71 |  |
| 906 | *Prkcd* | Protein kinase C delta | 2.02 |  |
| 907 | *Prnp* | Prion protein, mRNA (cDNA clone MGC:6164 IMAGE:3583938) | 5.3 |  |
| 908 | *Pros1* | Of protein S gene | 2.6 |  |
| 909 | *Prpf40a* | PRP40 pre-mRNA processing factor 40 homolog A (yeast) (Prpf40a), mRNA |  | 2.69 |
| 910 | *Prss22* | Protease, serine, 22 (Prss22), mRNA | 21.64 |  |
| 911 | *Psma1* | Proteasome (prosome, macropain) subunit, alpha type 1, mRNA (cDNA clone MGC:6546 IMAGE:2655483) |  | 3.02 |
| 912 | *Psma2* | Proteasome (prosome, macropain) subunit, alpha type 2, mRNA (cDNA clone MGC:25330 IMAGE:3156907) | 2.57 |  |
| 913 | *Psma7* | Proteasome (prosome, macropain) subunit, alpha type 7, mRNA (cDNA clone MGC:6440 IMAGE:2581897) |  | 3.19 |
| 914 | *Psmb10* | Proteasome (prosome, macropain) subunit, beta type 10, mRNA (cDNA clone MGC:5837 IMAGE:3583052) | 3.17 |  |
| 915 | *Psmb7* | Proteasome (prosome, macropain) subunit, beta type 7 (Psmb7), mRNA | 2.42 |  |
| 916 | *Psmb8* | Proteasome (prosome, macropain) subunit, beta type 8 (large multifunctional peptidase 7), mRNA (cDNA clone MGC:6535 IMAGE:265 | 5.45 | 2.25 |
| 917 | *Psmb9* | Proteasome (prosome, macropain) subunit, beta type 9 (large multifunctional peptidase 2) (Psmb9), mRNA |  | 2.19 |
| 918 | *Psmg2* | Proteasome (prosome, macropain) assembly chaperone 2 (Psmg2), mRNA |  | 2.59 |
| 919 | *Psph* | Phosphoserine phosphatase (Psph), mRNA | 2.77 | 2.52 |
| 920 | *Ptbp1* | Polypyrimidine tract binding protein 1, mRNA (cDNA clone MGC:25792 IMAGE:4022742) |  | 2.5 |
| 921 | *Ptcd2* | Pentatricopeptide repeat domain 2, mRNA (cDNA clone MGC:36809 IMAGE:3987809) |  | 5.88 |
| 922 | *Ptgr1* | Prostaglandin reductase 1, mRNA (cDNA clone MGC:6495 IMAGE:2648051) | 3.38 |  |
| 923 | *Ptk2b* | PTK2 protein tyrosine kinase 2 beta, mRNA (cDNA clone IMAGE:4022023) | 2.77 |  |
| 924 | *Ptplb* | Protein tyrosine phosphatase-like (proline instead of catalytic arginine), member b, mRNA (cDNA clone MGC:6798 IMAGE:2647850) | 2.33 |  |
| 925 | *Ptpn7* | CDNA clone MGC:178749 IMAGE:9053741 | 2.59 |  |
| 926 | *Ptprc* | Protein tyrosine phosphatase, receptor type, C (Ptprc), transcript variant 2, mRNA | 3.48 |  |
| 927 | *Ptprcap* | Ribosomal protein S6 kinase, polypeptide 2, mRNA (cDNA clone IMAGE:3597884) | 2.22 |  |
| 928 | *Ptpre* | Protein tyrosine phosphatase, receptor type, E (Ptpre), mRNA | 2.87 |  |
| 929 | *Ptpro* | Protein tyrosine phosphatase BK | 2.28 |  |
| 930 | *Pum1* | Pumilio 1 (Drosophila) (Pum1), mRNA |  | 2.09 |
| 931 | *Pycard* | PYD and CARD domain containing, mRNA (cDNA clone MGC:6811 IMAGE:2648391) | 3.58 |  |
| 932 | *Qrfp* | Pyroglutamylated RFamide peptide, mRNA (cDNA clone MGC:106072 IMAGE:3995356) | 4.72 |  |
| 933 | *Qsox1* | Quiescin Q6 sulfhydryl oxidase 1, mRNA (cDNA clone IMAGE:4920854) |  | 17.73 |
| 934 | *Rab34* | RAB34, member of RAS oncogene family, mRNA (cDNA clone MGC:47984 IMAGE:5058535) |  | 2.51 |
| 935 | *Rab38* | RAB38 | 3.32 | 2.04 |
| 936 | *Rab9* | RAB9, member RAS oncogene family, mRNA (cDNA clone MGC:11411 IMAGE:3964134) |  | 2.87 |
| 937 | *Rac2* | RAS-related C3 botulinum substrate 2, mRNA (cDNA clone MGC:6255 IMAGE:3495818) | 2.55 | 3.35 |
| 938 | *Rad51l1* | RAD51-like 1 (S. cerevisiae) (Rad51l1), mRNA | 3.08 | 4.11 |
| 939 | *Rarres1* | PREDICTED: Mus musculus retinoic acid receptor responder (tazarotene induced) 1 (Rarres1), mRNA | 10.65 |  |
| 940 | *Rars* | Arginyl-tRNA synthetase (Rars), mRNA | 3.04 |  |
| 941 | *Rasgrp4* | RAS guanyl releasing protein 4 (Rasgrp4), mRNA | 2.67 |  |
| 942 | *Rasl10a* | RAS-like, family 10, member A (Rasl10a), mRNA | 4.64 |  |
| 943 | *Rasl2-9* | RAS-like, family 2, locus 9 (Rasl2-9), mRNA |  | 2.67 |
| 944 | *Rassf5* | Ras association (RalGDS/AF-6) domain family member 5 (Rassf5), mRNA | 3.58 |  |
| 945 | *Rbl2* | Retinoblastoma-like 2, mRNA (cDNA clone MGC:18400 IMAGE:4240592) | 2.55 |  |
| 946 | *Rbms1* | RNA binding motif, single stranded interacting protein 1, mRNA (cDNA clone MGC:25244 IMAGE:4527742) |  | 2.17 |
| 947 | *Rbp1* | Retinol binding protein 1, cellular, mRNA (cDNA clone MGC:6258 IMAGE:3498107) | 4.24 | 5.86 |
| 948 | *Rbp4* | Retinol binding protein 4, plasma, mRNA (cDNA clone MGC:19321 IMAGE:4193984) | 2.95 |  |
| 949 | *Rcc2* | Regulator of chromosome condensation 2, mRNA (cDNA clone IMAGE:2654928) |  | 5.83 |
| 950 | Rcor1 | CDNA clone IMAGE:30466034 |  | 2 |
| 951 | *Rdh11* | Short-chain aldehyde dehydrogenase SCALD | 2.18 |  |
| 952 | *Rdh13* | Retinol dehydrogenase 13 (all-trans and 9-cis), mRNA (cDNA clone MGC:105205 IMAGE:30613493) |  | 2.16 |
| 953 | *Reep4* | Receptor accessory protein 4, mRNA (cDNA clone MGC:41264 IMAGE:1363539) | 3.55 |  |
| 954 | *Reep6* | Receptor accessory protein 6 (Reep6), mRNA | 2.48 | 2.36 |
| 955 | *Retnla* | Resistin like alpha, mRNA (cDNA clone MGC:35890 IMAGE:4189285) | 24.9 |  |
| 956 | *Retsat* | Retinol saturase (all trans retinol 13,14 reductase) (Retsat), mRNA | 2.37 |  |
| 957 | *Rexo2* | REX2, RNA exonuclease 2 homolog (S. cerevisiae) (Rexo2), mRNA |  | 4.62 |
| 958 | *Rgs10* | Regulator of G-protein signalling 10 (Rgs10), mRNA | 3.87 |  |
| 959 | *Rgs4* | Regulator of G-protein signaling 4, mRNA (cDNA clone MGC:6650 IMAGE:3496887) | 3.52 | 9.58 |
| 960 | *Rhoh* | Ras homolog gene family, member H (Rhoh), mRNA | 13.13 |  |
| 961 | *Ripk1* | Receptor (TNFRSF)-interacting serine-threonine kinase 1 (Ripk1), mRNA | 2.08 |  |
| 962 | *Ripk2* | Protein kinase RICK (Ripk2) | 2.1 |  |
| 963 | *Rnase4* | Ribonuclease, RNase A family 4, mRNA (cDNA clone MGC:11599 IMAGE:3967265) | 7.74 |  |
| 964 | *Rnf103* | Ring finger protein 103 (Rnf103), mRNA | 2.12 |  |
| 965 | *Rnf130* | Hypothetical LOC552912, mRNA (cDNA clone IMAGE:1493777) | 2.16 |  |
| 966 | *Rnf146* | CDNA clone IMAGE:4016974 | 3.75 | 2.63 |
| 967 | *Rnf181* | Ring finger protein 181 (Rnf181), mRNA |  | 5.78 |
| 968 | *Rod1* | ROD1 regulator of differentiation 1 (S. pombe) (Rod1), transcript variant 1, mRNA | 2.45 |  |
| 969 | *Romo1* | Reactive oxygen species modulator 1, mRNA (cDNA clone MGC:41030 IMAGE:1196408) |  | 4.09 |
| 970 | *Rpl11* | Ribosomal protein L11, mRNA (cDNA clone MGC:35693 IMAGE:2647882) | 3.4 |  |
| 971 | *Rpl30* | Ribosomal protein L30 (Rpl30), mRNA | 4.6 |  |
| 972 | *Rpl32* | Ribosomal protein L32, mRNA (cDNA clone MGC:107610 IMAGE:30122780) | 2.37 |  |
| 973 | *Rpl9* | Ribosomal protein L9 (Rpl9), mRNA | 2.02 |  |
| 974 | *Rps3a* | Ribosomal protein S3A (Rps3a), mRNA |  | 3.35 |
| 975 | *Rpsa* | Ribosomal protein SA, mRNA (cDNA clone MGC:47040 IMAGE:4500748) | 2.36 |  |
| 976 | *Rsl1d1* | Ribosomal L1 domain containing 1, mRNA (cDNA clone MGC:27753 IMAGE:2651674) |  | 3 |
| 977 | *Rtp4* | Receptor transporter protein 4, mRNA (cDNA clone MGC:28210 IMAGE:3990099) | 4.23 | 3.78 |
| 978 | *Runx1* | Runt related transcription factor 1 (Runx1), transcript variant 4, mRNA |  | 3.41 |
| 979 | *S100a11* | S100 calcium binding protein A11 (calgizzarin) (S100a11), mRNA | 3.31 |  |
| 980 | *S100a3* | S100 calcium binding protein A3 (S100a3), mRNA | 18.35 |  |
| 981 | *S100a4* | Mts1 gene | 17.53 |  |
| 982 | *S100a7a* | S100 calcium binding protein A7A, mRNA (cDNA clone MGC:130262 IMAGE:40053248) | 7.59 |  |
| 983 | *S100a8* | S100 calcium binding protein A8 (calgranulin A) (S100a8), mRNA | 12.87 | 6.93 |
| 984 | *S100a9* | S100 calcium binding protein A9 (calgranulin B), mRNA (cDNA clone MGC:41215 IMAGE:1332797) | 11.07 | 3.27 |
| 985 | *S1pr3* | Sphingosine-1-phosphate receptor 3 (S1pr3), mRNA |  | 2.15 |
| 986 | *Saa3* | Serum amyloid A 3 (Saa3), mRNA | 31.47 |  |
| 987 | *Sap25* | Sin3A-binding protein, SAP25 (Sap25), mRNA | 2.07 |  |
| 988 | *Sash3* | SAM and SH3 domain containing 3 (Sash3), mRNA | 2.73 |  |
| 989 | *Sc4mol* | Sterol-C4-methyl oxidase-like, mRNA (cDNA clone MGC:11745 IMAGE:3152545) | 4.27 |  |
| 990 | *Scand3* | SCAN domain containing 3, mRNA (cDNA clone MGC:169234 IMAGE:8860629) |  | 2.03 |
| 991 | *Scarb1* | Scavenger receptor class B, member 1 (Scarb1), mRNA | 2.14 | 2.5 |
| 992 | *Scarb2* | Scavenger receptor class B, member 2, mRNA (cDNA clone MGC:11451 IMAGE:3962669) | 3.27 |  |
| 993 | *Scel* | Sciellin (Scel), mRNA | 6.48 |  |
| 994 | *Scin* | Scinderin (Scin), mRNA | 10.83 |  |
| 995 | *Sdad1* | SDA1 domain containing 1, mRNA (cDNA clone IMAGE:3600976) |  | 2.06 |
| 996 | *Sdhd* | Succinate dehydrogenase complex, subunit D, integral membrane protein, mRNA (cDNA clone MGC:175620 IMAGE:40131036) |  | 2.26 |
| 997 | *Sec11c* | SEC11 homolog C (S. cerevisiae), mRNA (cDNA clone MGC:47104 IMAGE:3666130) | 2.79 |  |
| 998 | *Sec14l4* | SEC14-like 4 (S. cerevisiae), mRNA (cDNA clone IMAGE:5097361) | 3.63 |  |
| 999 | *Sectm1b* | Secreted and transmembrane 1B, mRNA (cDNA clone MGC:28332 IMAGE:4016267) | 6.04 |  |
| 1000 | *Selplg* | Selectin, platelet (p-selectin) ligand, mRNA (cDNA clone MGC:6637 IMAGE:3495181) | 9.4 | 2.77 |
| 1001 | *Sema4c* | Sema domain, immunoglobulin domain (Ig), transmembrane domain (TM) and short cytoplasmic domain, (semaphorin) 4C (Sema4c), mR |  | 2.36 |
| 1002 | *Sepp1* | Selenoprotein P, plasma, 1, mRNA (cDNA clone MGC:5722 IMAGE:3486761) | 3.51 |  |
| 1003 | *Serinc2* | Serine incorporator 2, mRNA (cDNA clone MGC:25877 IMAGE:4209575) | 2.86 | 2.94 |
| 1004 | *Serinc5* | Serine incorporator 5, mRNA (cDNA clone MGC:69958 IMAGE:6514513) | 2.57 |  |
| 1005 | *Serpinb6b* | NK13 (Serpinb6b) | 2.17 |  |
| 1006 | *Serping1* | Serine (or cysteine) peptidase inhibitor, clade G, member 1, mRNA (cDNA clone MGC:5908 IMAGE:3485810) | 3.93 |  |
| 1007 | *Sftpb* | Surfactant associated protein B (Sftpb), mRNA | 7.88 |  |
| 1008 | *Sgk1* | Serum/glucocorticoid regulated kinase 1, mRNA (cDNA clone MGC:11778 IMAGE:3594892) | 3.07 |  |
| 1009 | *Sgpp2* | Sphingosine-1-phosphate phosphotase 2, mRNA (cDNA clone MGC:182946 IMAGE:9087560) | 9.98 | 3.53 |
| 1010 | *Sgsh* | Mutant heparan N-sulfatase | 2.1 |  |
| 1011 | *Shc1* | Shcp52 (Shc) |  | 2.4 |
| 1012 | *Shc4* | SHC (Src homology 2 domain containing) family, member 4 (Shc4), mRNA | 2.35 |  |
| 1013 | Shfm1 | Split hand/foot malformation (ectrodactyly) type 1, mRNA (cDNA clone MGC:31011 IMAGE:5251089) | 2 |  |
| 1014 | *Siglecg* | Sialic acid binding immunoglobin-like lectin (Siglec-G) | 3.42 |  |
| 1015 | *Skil* | SKI-like, mRNA (cDNA clone IMAGE:3584831) |  | 2.53 |
| 1016 | *Skint11* | Skint 11 isoform a precursor (Skint11) mRNA, complete cds, alternatively spliced | 6.95 |  |
| 1017 | *Skint2* | Skint 2 isoform d precursor (Skint2) mRNA, complete cds, alternatively spliced | 8.95 | 2.57 |
| 1018 | *Slamf7* | Strain C57BL/6J leukocyte cell-surface antigen | 3.26 |  |
| 1019 | *Slc15a4* | Solute carrier family 15, member 4 (Slc15a4), mRNA | 2.03 |  |
| 1020 | *Slc16a1* | Solute carrier family 16 (monocarboxylic acid transporters), member 1, mRNA (cDNA clone MGC:5796 IMAGE:3499773) |  | 3.59 |
| 1021 | *Slc23a3* | Solute carrier family 23 (nucleobase transporters), member 3, mRNA (cDNA clone MGC:143681 IMAGE:40092152) | 15.45 |  |
| 1022 | *Slc25a13* | Solute carrier family 25 (mitochondrial carrier, adenine nucleotide translocator), member 13, mRNA (cDNA clone MGC:29043 IMAG |  | 2.92 |
| 1023 | *Slc25a45* | Solute carrier family 25, member 45 (Slc25a45), mRNA | 3.06 |  |
| 1024 | *Slc25a5* | Solute carrier family 25 (mitochondrial carrier, adenine nucleotide translocator), member 5 (Slc25a5), nuclear gene encoding | 2.04 | 2.07 |
| 1025 | *Slc27a4* | Solute carrier family 27 (fatty acid transporter), member 4, mRNA (cDNA clone MGC:28448 IMAGE:4158956) | 3.69 |  |
| 1026 | *Slc31a2* | Solute carrier family 31, member 2, mRNA (cDNA clone MGC:35783 IMAGE:4502064) | 2.49 |  |
| 1027 | *Slc35f2* | Solute carrier family 35, member F2 (Slc35f2), mRNA | 3.5 |  |
| 1028 | *Slc37a2* | Solute carrier family 37 (glycerol-3-phosphate transporter), member 2, mRNA (cDNA clone IMAGE:4913507) | 2.69 | 2.86 |
| 1029 | *Slc39a11* | Solute carrier family 39 (metal ion transporter), member 11 (Slc39a11), mRNA | 2.2 |  |
| 1030 | *Slc39a2* | Solute carrier family 39 (zinc transporter), member 2 (Slc39a2), mRNA | 2.71 | 2.55 |
| 1031 | *Slc40a1* | Solute carrier family 40 (iron-regulated transporter), member 1, mRNA (cDNA clone MGC:6489 IMAGE:2647365) | 2.13 |  |
| 1032 | *Slc41a1* | Solute carrier family 41, member 1, mRNA (cDNA clone IMAGE:4504894) | 2.08 |  |
| 1033 | *Slc43a2* | Slc43a2 mRNA for L-type amino acid transporter 4 | 2.83 |  |
| 1034 | *Slc44a1* | Solute carrier family 44, member 1 (Slc44a1), mRNA | 2.95 |  |
| 1035 | *Slc5a8* | Solute carrier family 5 (iodide transporter), member 8 (Slc5a8), mRNA | 6.13 | 2.14 |
| 1036 | *Slc6a14* | Na+ and Cl- coupled neutral and basic amino acid transporter ATB0,+ (Atb0,+) | 3.62 |  |
| 1037 | *Slc6a20a* | Solute carrier family 6 (neurotransmitter transporter), member 20A (Slc6a20a), mRNA | 4.69 |  |
| 1038 | *Slco2b1* | Solute carrier organic anion transporter family, member 2b1, mRNA (cDNA clone IMAGE:5133329) | 2.37 | 2.34 |
| 1039 | *Slfn2* | Schlafen 2 (Slfn2), mRNA | 3.32 |  |
| 1040 | *Slfn8* | Schlafen 8, mRNA (cDNA clone IMAGE:3982223) | 4.86 |  |
| 1041 | *Slk* | Ste20-related kinase SMAK (SMAK) | 2.13 |  |
| 1042 | *Slpi* | Secretory leukocyte peptidase inhibitor, mRNA (cDNA clone MGC:41142 IMAGE:1513866) | 41.86 | 8.01 |
| 1043 | *Smap1* | Stromal membrane-associated protein 1, mRNA (cDNA clone MGC:6912 IMAGE:2810817) | 2.31 | 2.72 |
| 1044 | *Smarca4* | SWI/SNF related, matrix associated, actin dependent regulator of chromatin, subfamily a, member 4, mRNA (cDNA clone IMAGE:502 |  | 2.17 |
| 1045 | *Smc4* | SMC4 structural maintenance of chromosomes 4-like 1 (yeast), mRNA (cDNA clone IMAGE:3589989) |  | 2.36 |
| 1046 | *Smox* | Polyamine oxidase-s | 2.43 | 2.63 |
| 1047 | *Smpdl3a* | Sphingomyelin phosphodiesterase, acid-like 3A (Smpdl3a), mRNA | 4.76 |  |
| 1048 | *Smpdl3b* | Sphingomyelin phosphodiesterase, acid-like 3B (Smpdl3b), mRNA | 2.67 | 3.06 |
| 1049 | *Smurf1* | SMAD specific E3 ubiquitin protein ligase 1, mRNA (cDNA clone MGC:28022 IMAGE:3660965) |  | 2.09 |
| 1050 | *Smyd3* | SET and MYND domain containing 3, mRNA (cDNA clone IMAGE:3493177) | 2.52 |  |
| 1051 | *Snap91* | Synaptosomal-associated protein 91, mRNA (cDNA clone MGC:25399 IMAGE:4511467) |  | 2.47 |
| 1052 | *Snhg11* | RIKEN cDNA E130013N09 gene, mRNA (cDNA clone IMAGE:4507176) | 2.42 |  |
| 1053 | *Snhg3* | RNA, U17d small nucleolar, mRNA (cDNA clone IMAGE:30918832) | 2.51 |  |
| 1054 | *Snw1* | SNW domain containing 1 (Snw1), mRNA |  | 4.96 |
| 1055 | *Snx18* | Sorting nexin 18 (Snx18), mRNA |  | 2.36 |
| 1056 | *Snx30* | Sorting nexin family member 30, mRNA (cDNA clone MGC:106086 IMAGE:4950374) | 2.65 |  |
| 1057 | *Soat1* | Sterol O-acyltransferase 1 (Soat1), mRNA | 2.37 |  |
| 1058 | *Socs5* | Suppressor of cytokine signaling 5 (Socs5), mRNA |  | 2.6 |
| 1059 | *Sord* | Sorbitol dehydrogenase (Sord), mRNA | 2.02 |  |
| 1060 | *Sox15* | SRY-box containing gene 15 (Sox15), mRNA |  | 2.57 |
| 1061 | *Sox21* | HMG-box protein (Sox21) | 2.38 |  |
| 1062 | *Sox4* | SRY-box containing gene 4 (Sox4), mRNA |  | 3.51 |
| 1063 | *Sox7* | SRY-box containing gene 7 (Sox7), mRNA | 2.47 | 3.39 |
| 1064 | *Sox9* | SRY-box containing gene 9, mRNA (cDNA clone MGC:38112 IMAGE:5320371) |  | 2.2 |
| 1065 | *Sp110* | Similar to Sp110 nuclear body protein, mRNA (cDNA clone IMAGE:3326350) | 6.95 |  |
| 1066 | *Spc24* | SPC24, NDC80 kinetochore complex component, homolog (S. cerevisiae) (Spc24), mRNA |  | 2.24 |
| 1067 | *Spink5* | Serine peptidase inhibitor, Kazal type 5 (Spink5), mRNA | 7.12 |  |
| 1068 | *Sprr1a* | Small proline-rich protein 1A, mRNA (cDNA clone MGC:41213 IMAGE:1362548) | 154.34 | 5.46 |
| 1069 | *Sprr1b* | Small proline-rich protein 1B (Sprr1b), mRNA | 18.01 | 33.51 |
| 1070 | *Sprr4* | Small proline-rich protein 4, mRNA (cDNA clone MGC:129389 IMAGE:40049167) | 67.7 | 9.52 |
| 1071 | *Spry2* | Sprouty 2 (Spry2) | 2.34 |  |
| 1072 | *Sqle* | Squalene epoxidase (Sqle), mRNA | 3.23 |  |
| 1073 | *Srp54a* | Signal recognition particle 54a, mRNA (cDNA clone MGC:30254 IMAGE:3708250) | 2.03 |  |
| 1074 | *Srpx2* | Sushi-repeat-containing protein, X-linked 2 (Srpx2), transcript variant 1, mRNA | 2.51 |  |
| 1075 | *Srxn1* | Sulfiredoxin 1 homolog (S. cerevisiae) (Srxn1), mRNA |  | 3.14 |
| 1076 | *Ssb* | Sjogren syndrome antigen B, mRNA (cDNA clone MGC:6189 IMAGE:3593742) |  | 2.75 |
| 1077 | *Ssr1* | Signal sequence receptor, alpha, mRNA (cDNA clone MGC:19285 IMAGE:4017600) |  | 2.33 |
| 1078 | *Stard5* | START domain-containing 5 protein (Stard5) | 2.6 |  |
| 1079 | *Stat1* | Signal transducer and activator of transcription 1, mRNA (cDNA clone MGC:6411 IMAGE:3587831) | 2.62 |  |
| 1080 | *Stat3* | Signal transducer and activator of transcription 3, mRNA (cDNA clone IMAGE:3665873) |  | 4.3 |
| 1081 | Stk17b | Serine/threonine kinase 17b (apoptosis-inducing) (Stk17b), mRNA | 2 |  |
| 1082 | *Stk38l* | Serine/threonine kinase 38 like, mRNA (cDNA clone MGC:70096 IMAGE:30135051) | 2.11 |  |
| 1083 | *Stom* | Stomatin, mRNA (cDNA clone MGC:6019 IMAGE:3593052) |  | 2.83 |
| 1084 | *Stra6* | Stimulated by retinoic acid gene 6 (Stra6), mRNA | 4.31 | 5.5 |
| 1085 | *Stx4a* | Syntaxin 4A (placental), mRNA (cDNA clone MGC:19006 IMAGE:4016871) | 3.73 |  |
| 1086 | *Stxbp3a* | Syntaxin binding protein 3A (Stxbp3a), mRNA | 2.03 |  |
| 1087 | *Surf1* | Surfeit gene 1, mRNA (cDNA clone MGC:6703 IMAGE:3584373) | 2.17 |  |
| 1088 | *Syt16* | Synaptotagmin XVI, mRNA (cDNA clone MGC:66541 IMAGE:5708126) | 2.55 |  |
| 1089 | *Tac4* | Tachykinin 4 (Tac4), mRNA | 11.3 |  |
| 1090 | *Taf3* | TAF3 RNA polymerase II, TATA box binding protein (TBP)-associated factor, mRNA (cDNA clone MGC:169241 IMAGE:8860636) |  | 2.11 |
| 1091 | *Tagln2* | Transgelin | 2.96 |  |
| 1092 | *Tank* | TRAF family member-associated Nf-kappa B activator, mRNA (cDNA clone MGC:13977 IMAGE:3492937) | 2.18 |  |
| 1093 | *Tax1bp1* | Tax1 (human T-cell leukemia virus type I) binding protein 1, mRNA (cDNA clone MGC:11692 IMAGE:3962810) | 2.73 |  |
| 1094 | *Tbl3* | Transducin (beta)-like 3 (Tbl3), mRNA |  | 2.3 |
| 1095 | *Tceal8* | Transcription elongation factor A (SII)-like 8, mRNA (cDNA clone MGC:27945 IMAGE:3588435) |  | 2.27 |
| 1096 | Tcfap2a | Transcription factor AP-2, alpha, mRNA (cDNA clone MGC:18479 IMAGE:3983850) | 2 |  |
| 1097 | *Tchh* | PREDICTED: Mus musculus trichohyalin (Tchh), mRNA | 17.44 |  |
| 1098 | *Tcp11* | T-complex protein 11 (Tcp11), transcript variant 1, mRNA | 3.36 |  |
| 1099 | *Tcp11l2* | T-complex 11 (mouse) like 2 (Tcp11l2), mRNA | 2.26 |  |
| 1100 | *Tfrc* | Transferrin receptor 1 (TFR1 gene) |  | 2.12 |
| 1101 | *Tgfbr2* | Transforming growth factor, beta receptor II (Tgfbr2), transcript variant 2, mRNA | 2.88 |  |
| 1102 | *Tgm4* | Experimental autoimmune prostatitis antigen 1 (Eapa1) | 3.28 |  |
| 1103 | *Tgm5* | Transglutaminase 5, mRNA (cDNA clone MGC:143848 IMAGE:40094000) | 3.17 |  |
| 1104 | *Tgm6* | Transglutaminase 6, mRNA (cDNA clone MGC:132929 IMAGE:40061392) | 8.88 |  |
| 1105 | *Tgoln1* | Trans-golgi network protein, mRNA (cDNA clone MGC:11627 IMAGE:3157708) | 3.13 |  |
| 1106 | *Thbs4* | Thrombospondin 4 (Thbs4), mRNA | 2.15 |  |
| 1107 | *Timm8b* | Translocase of inner mitochondrial membrane 8 homolog b (yeast), mRNA (cDNA clone MGC:35981 IMAGE:4237232) |  | 2.15 |
| 1108 | *Timp1* | Tissue inhibitor of metalloproteinase 1, mRNA (cDNA clone MGC:6151 IMAGE:3158222) | 3.58 |  |
| 1109 | *Tle1* | Transducin-like enhancer of split 1, homolog of Drosophila E(spl) (Tle1), mRNA |  | 2.58 |
| 1110 | *Tle6* | Transducin-like enhancer of split 6, homolog of Drosophila E(spl) (Tle6), mRNA | 3.63 | 4.78 |
| 1111 | *Tll2* | Tolloid-like-2 protein (Tll2) | 3.19 |  |
| 1112 | *Tlr4* | Toll-like receptor 4, mRNA (cDNA clone MGC:35879 IMAGE:3493732) | 2.52 |  |
| 1113 | *Tmbim4* | Transmembrane BAX inhibitor motif containing 4 (Tmbim4), mRNA | 3.34 |  |
| 1114 | *Tmco4* | Transmembrane and coiled-coil domains 4 (Tmco4), mRNA | 2.97 |  |
| 1115 | *Tmeff2* | Transmembrane protein with EGF-like and two follistatin-like domains 2, mRNA (cDNA clone MGC:41091 IMAGE:1397175) |  | 2.64 |
| 1116 | *Tmem106a* | Transmembrane protein 106A, mRNA (cDNA clone MGC:30290 IMAGE:5037750) | 3.03 |  |
| 1117 | *Tmem132e* | Transmembrane protein 132E (Tmem132e), mRNA | 2.11 |  |
| 1118 | *Tmem140* | Transmembrane protein 140, mRNA (cDNA clone MGC:38515 IMAGE:5352975) | 2.33 |  |
| 1119 | *Tmem158* | Transmembrane protein 158, mRNA (cDNA clone MGC:171054 IMAGE:8862449) |  | 2.44 |
| 1120 | *Tmem159* | Transmembrane protein 159 (Tmem159), mRNA | 5 |  |
| 1121 | *Tmem176a* | Transmembrane protein 176A (Tmem176a), transcript variant 1, mRNA | 2.95 | 2.45 |
| 1122 | *Tmem176b* | Transmembrane protein 176B, mRNA (cDNA clone MGC:13755 IMAGE:3582123) |  | 3.5 |
| 1123 | *Tmem218* | Transmembrane protein 218, mRNA (cDNA clone MGC:35754 IMAGE:5049687) |  | 2.24 |
| 1124 | *Tmem33* | Transmembrane protein 33, mRNA (cDNA clone MGC:36605 IMAGE:5342147) | 2.2 |  |
| 1125 | *Tmem45a* | Transmembrane protein 45a (Tmem45a), mRNA | 11.27 |  |
| 1126 | *Tmem45b* | Transmembrane protein 45b (Tmem45b), mRNA | 4.5 | 4.15 |
| 1127 | *Tmem51* | Transmembrane protein 51 (Tmem51), mRNA | 2.36 |  |
| 1128 | *Tmem56* | Transmembrane protein 56 (Tmem56), mRNA | 3.48 |  |
| 1129 | *Tmem71* | Transmembrane protein 71, mRNA (cDNA clone MGC:143747 IMAGE:40092864) | 2.43 |  |
| 1130 | *Tmem86a* | Transmembrane protein 86A (Tmem86a), mRNA | 3.72 |  |
| 1131 | *Tmprss11e* | Transmembrane protease, serine 11e, mRNA (cDNA clone MGC:143865 IMAGE:40094164) | 11.05 |  |
| 1132 | *Tmprss4* | Transmembrane protease, serine 4 (Tmprss4), mRNA | 8.01 |  |
| 1133 | *Tmsb4x* | Thymosin, beta 4, X chromosome, mRNA (cDNA clone MGC:7862 IMAGE:3501378) |  | 2.25 |
| 1134 | *Tnc* | Tenascin C (Tnc), mRNA |  | 4.05 |
| 1135 | *Tnfaip2* | Tumor necrosis factor, alpha-induced protein 2, mRNA (cDNA clone IMAGE:3709243) | 2.45 | 2.35 |
| 1136 | *Tnfrsf11b* | Tumor necrosis factor receptor superfamily, member 11b (osteoprotegerin) (Tnfrsf11b), mRNA | 2.1 |  |
| 1137 | *Tnfrsf1b* | Tumor necrosis factor receptor superfamily, member 1b (Tnfrsf1b), mRNA | 4.8 |  |
| 1138 | *Tnfrsf26* | Tumor necrosis factor receptor superfamily, member 26, mRNA (cDNA clone MGC:170447 IMAGE:8861842) | 2.17 |  |
| 1139 | *Tnfrsf4* | Tumor necrosis factor receptor superfamily, member 4 (Tnfrsf4), mRNA | 5.84 |  |
| 1140 | *Tnfsf10* | Tumor necrosis factor (ligand) superfamily, member 10 (Tnfsf10), mRNA | 4.01 | 2.56 |
| 1141 | *Tnfsf12* | Tumor necrosis factor (ligand) superfamily, member 13 (Tnfsf13), mRNA | 2.11 |  |
| 1142 | *Tnmd* | Chondromodulin-IB | 3.3 |  |
| 1143 | *Tns3* | Tensin 3, mRNA (cDNA clone IMAGE:5324278) |  | 2.19 |
| 1144 | *Tns4* | Tensin 4, mRNA (cDNA clone MGC:67703 IMAGE:4209161) | 7.77 |  |
| 1145 | *Tor3a* | Torsin family 3, member A (Tor3a), mRNA | 2.54 |  |
| 1146 | *Tox4* | RIKEN cDNA 5730589K01 gene, mRNA (cDNA clone IMAGE:3989816) |  | 2.24 |
| 1147 | *Tpi1* | Triosephosphate isomerase 1 (Tpi1), mRNA |  | 3.66 |
| 1148 | *Tpm4* | Tropomyosin 4 (Tpm4), mRNA |  | 5.52 |
| 1149 | *Tppp3* | Tubulin polymerization-promoting protein family member 3, mRNA (cDNA clone MGC:18840 IMAGE:4216285) |  | 2.68 |
| 1150 | *Trim10* | Tripartite motif protein TRIM10 (Trim10) | 2.05 | 3.47 |
| 1151 | *Trim12* | Tripartite motif-containing 12 (Trim12), mRNA | 2.86 |  |
| 1152 | *Trim30* | Tripartite motif-containing 30, mRNA (cDNA clone MGC:6159 IMAGE:3493256) | 3.61 |  |
| 1153 | *Trim62* | Tripartite motif-containing 62 (Trim62), mRNA | 2.27 |  |
| 1154 | *Trmt2b* | RIKEN cDNA 4732479N06 gene, mRNA (cDNA clone IMAGE:5401100) |  | 2.04 |
| 1155 | *Trnp1* | TMF1-regulated nuclear protein 1 (Trnp1), mRNA | 2.22 |  |
| 1156 | *Tslp* | Thymic stromal lymphopoietin (Tslp), mRNA | 3.34 |  |
| 1157 | *Tspan11* | Tetraspanin 11 (Tspan11), mRNA | 2.63 |  |
| 1158 | *Tspan4* | Tetraspanin 4 (Tspan4), mRNA | 2.44 |  |
| 1159 | *Tspo* | Translocator protein, mRNA (cDNA clone MGC:6086 IMAGE:3493196) | 3.61 | 2.32 |
| 1160 | *Tsta3* | Tissue specific transplantation antigen P35B (Tsta3), mRNA |  | 2.48 |
| 1161 | Ttc39a | Tetratricopeptide repeat domain 39A (Ttc39a), transcript variant 2, mRNA | 2 |  |
| 1162 | *Txndc17* | Thioredoxin domain containing 17, mRNA (cDNA clone MGC:40618 IMAGE:3673521) | 2.25 |  |
| 1163 | *Txnrd3* | Thioredoxin reductase 3 (Txnrd3), mRNA | 2.65 | 2.31 |
| 1164 | *Tyrobp* | TYRO protein tyrosine kinase binding protein (Tyrobp), mRNA |  | 9.28 |
| 1165 | *Ubxn1* | UBX domain protein 1 (Ubxn1), mRNA |  | 6.11 |
| 1166 | *Ubxn2a* | UBX domain protein 2A (Ubxn2a), mRNA | 2.11 |  |
| 1167 | *Uchl5* | Ubiquitin carboxyl-terminal esterase L5, mRNA (cDNA clone MGC:6295 IMAGE:2650799) | 2.27 |  |
| 1168 | *Ucp2* | Uncoupling protein 2 (mitochondrial, proton carrier), mRNA (cDNA clone MGC:13955 IMAGE:4205625) |  | 6.99 |
| 1169 | *Upf2* | UPF2 regulator of nonsense transcripts homolog (yeast), mRNA (cDNA clone MGC:169983 IMAGE:8861378) | 2.04 |  |
| 1170 | *Uqcrfs1* | Ubiquinol-cytochrome c reductase, Rieske iron-sulfur polypeptide 1, mRNA (cDNA clone MGC:30985 IMAGE:5249225) |  | 2.43 |
| 1171 | *Uqcrh* | Ubiquinol-cytochrome c reductase hinge protein (Uqcrh), mRNA |  | 4.5 |
| 1172 | *Use1* | Unconventional SNARE in the ER 1 homolog (S. cerevisiae) (Use1), transcript variant 1, mRNA | 2.4 |  |
| 1173 | *Usmg5* | Upregulated during skeletal muscle growth 5 (Usmg5), mRNA | 2.01 |  |
| 1174 | *Usp25* | Ubiquitin-specific processing protease (Usp25) | 2.07 |  |
| 1175 | *Usp5* | Ubiquitin specific peptidase 5 (isopeptidase T) (Usp5), mRNA |  | 3.51 |
| 1176 | *Vamp8* | Vesicle-associated membrane protein 8, mRNA (cDNA clone MGC:13842 IMAGE:4205509) | 2.77 |  |
| 1177 | *Vdr* | Vitamin D receptor, mRNA (cDNA clone MGC:12147 IMAGE:3710866) | 9.35 | 2.1 |
| 1178 | *Vegfa* | Vascular endothelial growth factor A, mRNA (cDNA clone IMAGE:4212199) |  | 8.75 |
| 1179 | *Vim* | Vimentin (Vim), mRNA |  | 3.23 |
| 1180 | *Vnn1* | Vanin 1, mRNA (cDNA clone MGC:29064 IMAGE:5041670) | 2.42 |  |
| 1181 | *Vps26a* | Vacuolar protein sorting 26 homolog A (yeast) (Vps26a), transcript variant 1, mRNA | 2.22 |  |
| 1182 | *Vsnl1* | Visinin-like protein 1 | 2.61 | 2.47 |
| 1183 | *Vti1b* | Vesicle transport through interaction with t-SNAREs 1B homolog, mRNA (cDNA clone MGC:13826 IMAGE:4220215) |  | 3.78 |
| 1184 | *Wdr13* | Memory-related protein | 2.37 |  |
| 1185 | *Whamm* | MKIAA1971 protein | 2.22 |  |
| 1186 | *Wipi1* | WD repeat domain, phosphoinositide interacting 1 (Wipi1), mRNA | 2.43 | 3 |
| 1187 | *Wisp2* | WNT1 inducible signaling pathway protein 2, mRNA (cDNA clone MGC:41105 IMAGE:1067632) | 13.46 |  |
| 1188 | *Wnt10a* | Wingless related MMTV integration site 10a, mRNA (cDNA clone MGC:25311 IMAGE:4921327) | 4.64 |  |
| 1189 | *Wnt11* | Wingless-related MMTV integration site 11 (Wnt11), mRNA | 2.48 | 3.38 |
| 1190 | *X76971* | PREDICTED: Mus musculus similar to TCR V alpha chain (LOC100043308), mRNA |  | 3.33 |
| 1191 | *Xcl1* | Chemokine (C motif) ligand 1 (Xcl1), mRNA | 16.69 |  |
| 1192 | *Xdh* | Similar to hypothetical protein MGC37588, mRNA (cDNA clone MGC:28125 IMAGE:3980327) | 2.95 |  |
| 1193 | *Yipf5* | Yip1 domain family, member 5, mRNA (cDNA clone MGC:7012 IMAGE:3155690) | 2.39 |  |
| 1194 | *Ypel5* | Yippee-like 5 (Drosophila), mRNA (cDNA clone MGC:36888 IMAGE:4921751) | 2.18 |  |
| 1195 | *Ythdf2* | YTH domain family 2 (Ythdf2), mRNA |  | 2.35 |
| 1196 | *Zbtb7c* | Zinc finger and BTB domain containing 7C (Zbtb7c), mRNA |  | 3.43 |
| 1197 | *Zc3h12a* | Zinc finger CCCH type containing 12A (Zc3h12a), mRNA | 4.35 |  |
| 1198 | *Zc3hav1* | Zinc finger CCCH type, antiviral 1, mRNA (cDNA clone MGC:27750 IMAGE:2651558) | 2.56 |  |
| 1199 | *Zcchc10* | Zinc finger, CCHC domain containing 10, mRNA (cDNA clone MGC:35654 IMAGE:4504126) |  | 2.11 |
| 1200 | *Zcchc17* | Zinc finger, CCHC domain containing 17 (Zcchc17), mRNA |  | 2.32 |
| 1201 | *Zcchc9* | Zinc finger, CCHC domain containing 9 (Zcchc9), mRNA | 2.42 |  |
| 1202 | *Zdhhc18* | Zinc finger, DHHC domain containing 18, mRNA (cDNA clone IMAGE:40131019) |  | 4.01 |
| 1203 | *Zdhhc21* | Zinc finger, DHHC domain containing 21 (Zdhhc21), mRNA | 2.31 |  |
| 1204 | *Zfp326* | Zinc finger protein 326, mRNA (cDNA clone MGC:46856 IMAGE:4948371) |  | 2.12 |
| 1205 | *Zfp81* | Zinc finger protein 81, mRNA (cDNA clone IMAGE:4985927) | 2.53 |  |
| 1206 | *Zmynd15* | Zinc finger, MYND-type containing 15, mRNA (cDNA clone MGC:183999 IMAGE:9087999) | 5.56 | 4.17 |
| 1207 | *Zranb2* | Zinc finger, RAN-binding domain containing 2, mRNA (cDNA clone MGC:170200 IMAGE:8861595) |  | 2.88 |
