# Supplementary material for: Neotenic phenomenon in gene expression in the skin of Foxn1- deficient (nude) mice - a projection for regenerative skin wound healing
Source: BMC Genomics. 2017 Jan 9;18:56. doi: 10.1186/s12864-016-3401-z (PMC5223329; doi:10.1186/s12864-016-3401-z)
Supplement: Additional file 4: Table S4. — Genes that are down-regulated in nude (vs B6) skin and epidermis, and not in common with E14 (vs E18). (DOCX 101 kb) [file 12864_2016_3401_MOESM4_ESM.docx]

Table S4.

Genes that are down-regulated in nude (vs B6) skin and epidermis, and uncommon with E14 (vs E18).

| No. | Gene | Gene name | Down in nude skin | Down in nude epidermis |
| --- | --- | --- | --- | --- |
| 1 | *16621* | kallikrein B, plasma 1 |  | 2.85 |
| 2 | *41161* | Septin 9 (Sept9), transcript variant 4, mRNA | 3.81 |  |
| 3 | *71198* | OTU domain containing 1 |  | 2.19 |
| 4 | *93707* | protocadherin gamma subfamily C, 4 | 4.13 |  |
| 5 | *93708* | protocadherin gamma subfamily C, 5 | 3.86 |  |
| 6 | *93711* | protocadherin gamma subfamily A, 3 | 3.97 |  |
| 7 | *93713* | protocadherin gamma subfamily A, 5 | 4.12 |  |
| 8 | *93716* | protocadherin gamma subfamily A, 8 | 3.62 |  |
| 9 | *93723* | protocadherin gamma subfamily A, 11 | 3.96 |  |
| 10 | *93898* | LAG1 homolog, ceramide synthase 1 | 2.16 |  |
| 11 | *94223* | DiGeorge syndrome critical region gene 8 | 2.12 |  |
| 12 | *108112* | eukaryotic translation initiation factor 4E binding protein 3 | 2.37 |  |
| 13 | *320456* | RIKEN cDNA B330016D10 gene | 2.08 |  |
| 14 | *320586* | RIKEN cDNA A630089N07 gene |  | 5.29 |
| 15 | *381148* | predicted gene 1614 |  | 2.63 |
| 16 | *382643* |  |  | 2.41 |
| 17 | *434350* | RIKEN cDNA 9430091E24 gene | 2 |  |
| 18 | *623230* | transmembrane protein 200B | 2.27 |  |
| 19 | *667572* | glyceraldehyde-3-phosphate dehydrogenase pseudogene |  | 6.5 |
| 20 | *668902* | predicted gene 9422 |  | 3.84 |
| 21 | *100038822* |  | 2.37 |  |
| 22 | *100039915* |  | 3.36 |  |
| 23 | *100040724* | miRNA containing gene | 4.18 |  |
| 24 | *100040860* | predicted gene 3006 |  | 2.88 |
| 25 | *100042311* | predicted gene 3785 | 2.48 |  |
| 26 | *100042420* |  | 5.25 |  |
| 27 | *100042497* | predicted gene 3871 |  | 4.91 |
| 28 | *100042932* | predicted gene 14813 | 6.32 |  |
| 29 | *100043258* | predicted gene 4320 | 2.64 |  |
| 30 | *100044052* |  | 2.5 |  |
| 31 | *100044112* | similar to even skipped homeotic gene 2 homolog |  | 2.29 |
| 32 | *1,00E+08* |  | 10.39 |  |
| 33 | *1110002H13Rik* | RIKEN cDNA 1110002H13 gene (1110002H13Rik), mRNA | 8.31 |  |
| 34 | *1110020G09Rik* | RIKEN cDNA 1110020G09 gene (1110020G09Rik), transcript variant 1, mRNA | 2.4 |  |
| 35 | *1110020P15Rik* | RIKEN cDNA 1110020P15 gene, mRNA (cDNA clone MGC:37480 IMAGE:4984172) | 2.71 |  |
| 36 | *1190002N15Rik* | RIKEN cDNA 1190002N15 gene (1190002N15Rik), mRNA | 3.72 |  |
| 37 | *1600002H07Rik* | RIKEN cDNA 1600002H07 gene (1600002H07Rik), mRNA | 2.78 |  |
| 38 | *1700029G01Rik* | Mus musculus, RIKEN cDNA 1700029G01 gene, clone IMAGE:4219304, mRNA |  | 2.14 |
| 39 | *2010005J08Rik* | RIKEN cDNA 2010005J08 gene (2010005J08Rik), transcript variant 2, mRNA | 2.14 |  |
| 40 | *2010305A19Rik* | RIKEN cDNA 2010305A19 gene, mRNA (cDNA clone MGC:8003 IMAGE:3585966) | 2.09 |  |
| 41 | *2010309E21Rik* | RIKEN cDNA 2010309E21 gene, mRNA (cDNA clone MGC:35736 IMAGE:5100000) | 2.58 |  |
| 42 | *2210404O07Rik* | RIKEN cDNA 2210404O07 gene (2210404O07Rik), mRNA |  | 5.39 |
| 43 | *2310002L09Rik* | RIKEN cDNA 2310002L09 gene, mRNA (cDNA clone MGC:144294 IMAGE:40100599) | 3.26 |  |
| 44 | *2310004N24Rik* | RIKEN cDNA 2310004N24 gene, mRNA (cDNA clone MGC:41326 IMAGE:1069092) |  | 2.21 |
| 45 | *2310015B20Rik* | PREDICTED: Mus musculus RIKEN cDNA 2310015B20 gene (2310015B20Rik), mRNA | 6.99 |  |
| 46 | *2310022B05Rik* | RIKEN cDNA 2310022B05 gene, mRNA (cDNA clone MGC:73467 IMAGE:6401577) | 2.01 |  |
| 47 | *2310045A20Rik* | RIKEN cDNA 2310045A20 gene, mRNA (cDNA clone IMAGE:4988442) | 2.63 |  |
| 48 | *2410076I21Rik* | RIKEN cDNA 2410076I21 gene, mRNA (cDNA clone IMAGE:1448701) | 4.81 |  |
| 49 | *2610528E23Rik* | RIKEN cDNA 2610528E23 gene, mRNA (cDNA clone MGC:48272 IMAGE:3371329) | 2.05 |  |
| 50 | *2810432L12Rik* | RIKEN cDNA 2810432L12 gene (2810432L12Rik), mRNA | 2.22 | 4.38 |
| 51 | *3110057O12Rik* | RIKEN cDNA 3110057O12 gene, mRNA (cDNA clone IMAGE:4007705) | 2.34 |  |
| 52 | *3830431G21Rik* | RIKEN cDNA 3830431G21 gene, mRNA (cDNA clone MGC:169226 IMAGE:8860621) |  | 3.83 |
| 53 | *4631416L12Rik* | RIKEN cDNA 4631416L12 gene (4631416L12Rik), mRNA | 2.88 |  |
| 54 | *4732471D19Rik* | RIKEN cDNA 4732471D19 gene, mRNA (cDNA clone IMAGE:5357053) | 2.36 |  |
| 55 | *4921501E09Rik* | RIKEN cDNA 4921501E09 gene, mRNA (cDNA clone MGC:183866 IMAGE:9087866) | 3.65 |  |
| 56 | *4930422G04Rik* | RIKEN cDNA 4930422G04 gene, mRNA (cDNA clone IMAGE:5068638) | 2.74 |  |
| 57 | *5430432N15Rik* | PREDICTED: Mus musculus RIKEN cDNA 5430432N15 gene (5430432N15Rik), mRNA | 3.33 |  |
| 58 | *6230427J02Rik* | RIKEN cDNA 6230427J02 gene, mRNA (cDNA clone MGC:144382 IMAGE:40101503) | 2.79 |  |
| 59 | *6330403K07Rik* | RIKEN cDNA 6330403K07 gene (6330403K07Rik), mRNA | 9.35 |  |
| 60 | *6332401O19Rik* | RIKEN cDNA 6332401O19 gene, mRNA (cDNA clone MGC:182393 IMAGE:9056287) |  | 3.25 |
| 61 | *6430706D22Rik* | RIKEN cDNA 6430706D22 gene, mRNA (cDNA clone IMAGE:3156506) | 2.37 |  |
| 62 | *8430408G22Rik* | RIKEN cDNA 8430408G22 gene (8430408G22Rik), mRNA |  | 9.63 |
| 63 | *8430427H17Rik* | RIKEN cDNA 8430427H17 gene (8430427H17Rik), transcript variant 2, mRNA | 2.61 |  |
| 64 | *9130213B05Rik* | RIKEN cDNA 9130213B05 gene (9130213B05Rik), mRNA | 2.75 |  |
| 65 | *9130404D08Rik* | RIKEN cDNA 9130404D08 gene (9130404D08Rik), mRNA | 2.2 |  |
| 66 | *9530091C08Rik* | RIKEN cDNA 9530091C08 gene (9530091C08Rik), mRNA | 2.51 |  |
| 67 | *9630013A20Rik* | PREDICTED: Mus musculus RIKEN cDNA 9630013A20 gene (9630013A20Rik), mRNA | 5.56 |  |
| 68 | *A2bp1* | Hexaribonucleotide binding protein 1 (Hrnbp1) | 5.08 |  |
| 69 | *A630038E17Rik* | RIKEN cDNA A630038E17 gene, mRNA (cDNA clone IMAGE:4168121) |  | 6.7 |
| 70 | *A930038C07Rik* | RIKEN cDNA A930038C07 gene, mRNA (cDNA clone MGC:54980 IMAGE:6489681) |  | 2.47 |
| 71 | *Abat* | 4-aminobutyrate aminotransferase, mRNA (cDNA clone IMAGE:30532686) | 2.58 |  |
| 72 | *Abca5* | ATP-binding cassette, sub-family A (ABC1), member 5 (Abca5), mRNA |  | 5.08 |
| 73 | *Abcb1b* | ATP-binding cassette, sub-family B (MDR/TAP), member 1B (Abcb1b), mRNA |  | 2.02 |
| 74 | *Abcc5* | ATP-binding cassette protein (Abcc5a) |  | 2.93 |
| 75 | *Ablim3* | Actin binding LIM protein family, member 3, mRNA (cDNA clone MGC:106450 IMAGE:30534236) | 2.13 | 2.68 |
| 76 | *Abr* | Active BCR-related gene (Abr), transcript variant 2, mRNA |  | 2.07 |
| 77 | *Abtb2* | CDNA clone IMAGE:3465372 |  | 2.01 |
| 78 | *Acad10* | Acyl-Coenzyme A dehydrogenase family, member 10, mRNA (cDNA clone IMAGE:4950406) |  | 2.8 |
| 79 | *Acd* | Adrenocortical dysplasia, mRNA (cDNA clone IMAGE:3500864) | 4 |  |
| 80 | *Acer1* | Alkaline ceramidase |  | 2.04 |
| 81 | *Acer3* | Alkaline ceramidase 3, mRNA (cDNA clone MGC:36600 IMAGE:5324078) |  | 2.04 |
| 82 | *Ache* | Acetylcholinesterase (Ache), mRNA |  | 4.18 |
| 83 | *Acsl6* | Acyl-CoA synthetase long-chain family member 6 (Acsl6), transcript variant 1, mRNA | 4.47 |  |
| 84 | *Acss1* | Acyl-CoA synthetase short-chain family member 1, mRNA (cDNA clone MGC:31687 IMAGE:4911679) | 2.2 |  |
| 85 | *Actc1* | Actin, alpha, cardiac muscle 1 (Actc1), mRNA | 25.28 |  |
| 86 | *Actn2* | Actinin alpha 2 (Actn2) | 6.95 |  |
| 87 | *Actr6* | ARP6 actin-related protein 6 homolog (yeast) (Actr6), mRNA | 2.51 |  |
| 88 | *Adam19* | Meltrin beta |  | 2.32 |
| 89 | *Adamts14* | A disintegrin-like and metallopeptidase (reprolysin type) with thrombospondin type 1 motif, 14, mRNA (cDNA clone MGC:189783 I |  | 5.14 |
| 90 | *Adarb1* | Adenosine deaminase ADAR2 mRNA, complete cds; alternatively spliced |  | 2.84 |
| 91 | *Adcy1* | Adenylate cyclase 1 (Adcy1), mRNA | 2.07 | 3.67 |
| 92 | *Adcy6* | Adenylate cyclase 6 (Adcy6), mRNA |  | 3.52 |
| 93 | *Adh1* | Alcohol dehydrogenase 1 (class I), mRNA (cDNA clone MGC:18885 IMAGE:4238555) |  | 4.16 |
| 94 | *Adh6a* | Alcohol dehydrogenase 6A (class V) (Adh6a), mRNA |  | 5.02 |
| 95 | *Adprhl1* | ADP-ribosylhydrolase like 1, mRNA (cDNA clone MGC:70059 IMAGE:30134000) | 7.51 |  |
| 96 | *Adrb2* | Adrenergic receptor, beta 2, mRNA (cDNA clone MGC:41462 IMAGE:1349283) |  | 3.16 |
| 97 | *Adrbk2* | Adrenergic receptor kinase, beta 2 (Adrbk2), transcript variant 2, mRNA |  | 2 |
| 98 | *Afp* | Alpha fetoprotein (Afp), mRNA | 13.12 |  |
| 99 | *Aga* | Aspartylglucosaminidase, mRNA (cDNA clone MGC:178859 IMAGE:9053851) |  | 2.57 |
| 100 | *Ahdc1* | AT hook, DNA binding motif, containing 1 (Ahdc1), mRNA | 2.8 |  |
| 101 | *AI597468* | Expressed sequence AI597468 (AI597468), mRNA | 3.34 |  |
| 102 | *Akap12* | A kinase (PRKA) anchor protein (gravin) 12 (Akap12), mRNA | 2.94 |  |
| 103 | *Akap6* | A-kinase anchoring protein alpha (Akapalpha) | 3.96 |  |
| 104 | *Alas2* | Aminolevulinic acid synthase 2, erythroid, mRNA (cDNA clone IMAGE:5054102) | 9.2 |  |
| 105 | *Alb* | Serum albumin (alb gene) | 2.68 |  |
| 106 | *Alk* | Anaplastic lymphoma kinase (Alk), mRNA |  | 11.09 |
| 107 | *Alox12* | Arachidonate 12-lipoxygenase (Alox12), mRNA |  | 6.1 |
| 108 | *Amot* | Angiomotin (Amot), mRNA | 6.53 |  |
| 109 | *Ank1* | Ankyrin 1, erythroid (Ank1), transcript variant 2, mRNA | 4.04 |  |
| 110 | *Ank2* | Ankyrin 2, brain (Ank2), transcript variant 3, mRNA | 2.75 |  |
| 111 | *Ankrd10* | Ankyrin repeat domain 10 (Ankrd10), mRNA | 3.99 |  |
| 112 | *Ankrd28* | Ankyrin repeat domain 28 (Ankrd28), mRNA | 2.44 |  |
| 113 | *Ankrd52* | Ankyrin repeat domain 52, mRNA (cDNA clone MGC:144061 IMAGE:40097194) | 2.71 |  |
| 114 | *Ankrd6* | Ankyrin repeat domain 6 (Ankrd6), transcript variant 2, mRNA | 2.31 | 4.18 |
| 115 | *Anks1* | Ankyrin repeat and SAM domain containing 1, mRNA (cDNA clone IMAGE:3155544) | 2.03 |  |
| 116 | *Antxr1* | Tumor endothelial marker 8 precursor (Tem8) |  | 2.13 |
| 117 | *Anxa6* | Annexin A6, mRNA (cDNA clone MGC:6574 IMAGE:3482035) | 2.13 |  |
| 118 | *Anxa8* | Annexin A8, mRNA (cDNA clone MGC:40628 IMAGE:5322310) |  | 2.06 |
| 119 | *Ap2a1* | Adaptor protein complex AP-2, alpha 1 subunit, mRNA (cDNA clone MGC:25419 IMAGE:4481643) | 3.35 |  |
| 120 | *Apln* | Preproapelin (preproapelin gene) | 2.04 |  |
| 121 | *Aplnr* | Apelin receptor, mRNA (cDNA clone MGC:31413 IMAGE:4457726) | 3.26 |  |
| 122 | *Apoa1* | Apolipoprotein A-I, mRNA (cDNA clone MGC:29972 IMAGE:5123746) | 7.91 |  |
| 123 | *Appl2* | Adaptor protein, phosphotyrosine interaction, PH domain and leucine zipper containing 2 (Appl2), mRNA |  | 2.02 |
| 124 | *Arap3* | ArfGAP with RhoGAP domain, ankyrin repeat and PH domain 3, mRNA (cDNA clone IMAGE:4024601) | 2.48 | 2.45 |
| 125 | *Arg1* | Arginase, liver, mRNA (cDNA clone MGC:13983 IMAGE:4163626) |  | 11.56 |
| 126 | *Arhgap28* | MKIAA1314 protein | 7.81 |  |
| 127 | *Arhgef4* | Rho guanine nucleotide exchange factor (GEF) 4, mRNA (cDNA clone MGC:170998 IMAGE:8862393) |  | 2.43 |
| 128 | *Armcx2* | Armadillo repeat containing, X-linked 2, mRNA (cDNA clone IMAGE:2655807) | 4.49 |  |
| 129 | *Arrdc3* | Arrestin domain containing 3, mRNA (cDNA clone IMAGE:5344962) |  | 2.04 |
| 130 | *Arsa* | TISP73 |  | 4.51 |
| 131 | *Arsj* | Arylsulfatase J, mRNA (cDNA clone MGC:143729 IMAGE:40092671) | 2.87 |  |
| 132 | *Art5* | Mono(ADP ribosyl)transferase (Art5 gene), splice variant 1 | 2.34 |  |
| 133 | *Ascc1* | Activating signal cointegrator 1 complex subunit 1, mRNA (cDNA clone MGC:31553 IMAGE:4503445) | 2 |  |
| 134 | *Asb2* | Ankyrin repeat and SOCS box-containing 2 (Asb2), mRNA | 3.26 |  |
| 135 | *Aspm* | Abnormal spindle (Asp) | 2.66 |  |
| 136 | *Asrgl1* | Asparaginase like 1 (Asrgl1), mRNA | 2.57 |  |
| 137 | *Asxl3* | PREDICTED: Mus musculus additional sex combs like 3 (Drosophila) (Asxl3), mRNA | 3.75 |  |
| 138 | *Atad3a* | TOB3 | 2.51 |  |
| 139 | *Ate1* | Arginyltransferase 1 (Ate1), transcript variant 1, mRNA | 2.1 |  |
| 140 | *Atg4b* | Autophagy-related 4B (yeast), mRNA (cDNA clone MGC:28676 IMAGE:4237950) | 2.03 |  |
| 141 | *Atic* | 5-aminoimidazole-4-carboxamide ribonucleotide formyltransferase/IMP cyclohydrolase (Atic), mRNA | 2.26 |  |
| 142 | *Atp1b2* | ATPase, Na+/K+ transporting, beta 2 polypeptide (Atp1b2), mRNA | 6.27 |  |
| 143 | *Atp5c1* | ATP synthase, H+ transporting, mitochondrial F1 complex, gamma polypeptide 1, mRNA (cDNA clone MGC:6552 IMAGE:2655884) | 2.78 |  |
| 144 | *Atp5o* | ATP synthase, H+ transporting, mitochondrial F1 complex, O subunit (Atp5o), nuclear gene encoding mitochondrial protein, mRNA | 5.98 |  |
| 145 | *Atp6v0a1* | ATPase, H+ transporting, lysosomal V0 subunit A1 (Atp6v0a1), mRNA |  | 2.39 |
| 146 | *AU040320* | Expressed sequence AU040320 (AU040320), transcript variant 3, mRNA | 2.11 |  |
| 147 | *AW551984* | Expressed sequence AW551984, mRNA (cDNA clone MGC:74355 IMAGE:30250574) | 4 |  |
| 148 | *B230206H07Rik* | RIKEN cDNA B230206H07 gene (B230206H07Rik), mRNA |  | 2.58 |
| 149 | *B4galnt2* | Beta-1,4-N-acetyl-galactosaminyl transferase 2 (B4galnt2), mRNA |  | 10.64 |
| 150 | *Bat2d* | BAT2 domain containing 1 (Bat2d), mRNA | 3.32 |  |
| 151 | *Bbs10* | PREDICTED: Mus musculus Bardet-Biedl syndrome 10 (human), transcript variant 1 (Bbs10), mRNA | 2.09 |  |
| 152 | *Bbs2* | Bardet-Biedl syndrome 2 (human) (Bbs2), mRNA |  | 2.09 |
| 153 | *BC020535* | CDNA sequence BC020535 (BC020535), mRNA | 2.87 |  |
| 154 | *BC021381* | CDNA sequence BC021381, mRNA (cDNA clone MGC:38018 IMAGE:5150988) |  | 2.28 |
| 155 | *BC030046* | CDNA sequence BC030046, mRNA (cDNA clone MGC:183956 IMAGE:9087956) | 5.14 |  |
| 156 | *BC039966* | PREDICTED: Mus musculus cDNA sequence BC039966 (BC039966), mRNA |  | 4.15 |
| 157 | *Bcl2l14* | BCL2-like 14 (apoptosis facilitator), mRNA (cDNA clone MGC:38086 IMAGE:5253808) |  | 3.03 |
| 158 | *Bcl6b* | B-cell CLL/lymphoma 6, member B (Bcl6b), mRNA | 3.89 |  |
| 159 | *Best2* | Bestrophin 2, mRNA (cDNA clone MGC:37437 IMAGE:4982413) |  | 3.53 |
| 160 | *Bex1* | Brain expressed X-linked protein 1 (Bex1) | 17.37 |  |
| 161 | *Bin1* | Bridging integrator 1 (Bin1), transcript variant 1, mRNA | 2.65 |  |
| 162 | *Bmp2* | Bone morphogenetic protein 2 (Bmp2), mRNA | 3.55 | 2.4 |
| 163 | *Bmp4* | Bone morphogenetic protein 4, mRNA (cDNA clone MGC:31017 IMAGE:4192158) |  | 3.23 |
| 164 | *Bmpr1b* | Bone morphogenetic protein receptor, type 1B (Bmpr1b), mRNA | 2.75 |  |
| 165 | *Bpgm* | 2,3-bisphosphoglycerate mutase, mRNA (cDNA clone MGC:6263 IMAGE:3587820) | 3.1 |  |
| 166 | *Brca1* | Breast cancer 1 (Brca1), mRNA | 2.63 |  |
| 167 | *Brca2* | Breast cancer 2 (Brca2), transcript variant 2, mRNA | 2.44 |  |
| 168 | *Brd8* | Bromodomain containing 8 (Brd8), mRNA | 2.05 |  |
| 169 | *Bri3bp* | Bri3 binding protein (Bri3bp), mRNA | 2.47 |  |
| 170 | *Brms1l* | Breast cancer metastasis-suppressor 1-like (Brms1l), mRNA | 3.07 |  |
| 171 | *Btbd12* | BTB (POZ) domain containing 12, mRNA (cDNA clone IMAGE:30536895) | 2.28 |  |
| 172 | *Btc* | Betacellulin, epidermal growth factor family member (Btc), mRNA |  | 4.04 |
| 173 | *Bves* | Blood vessel epicardial substance (Bves), mRNA | 7.73 |  |
| 174 | *Bzw1* | Basic leucine zipper and W2 domains 1, mRNA (cDNA clone MGC:25505 IMAGE:4910833) | 2.86 |  |
| 175 | *Bzw2* | Basic leucine zipper and W2 domains 2, mRNA (cDNA clone MGC:7203 IMAGE:3482251) | 2.92 |  |
| 176 | *C030030A07Rik* | RIKEN cDNA C030030A07 gene (C030030A07Rik), mRNA |  | 6 |
| 177 | *C530008M17Rik* | RIKEN cDNA C530008M17 gene, mRNA (cDNA clone IMAGE:30632196) | 7.06 |  |
| 178 | *Cabc1* | Chaperone, ABC1 activity of bc1 complex like (S. pombe), mRNA (cDNA clone MGC:31744 IMAGE:4922892) |  | 2.25 |
| 179 | *Cacnb1* | L-type calcium channel beta 1C subunit | 3.47 | 3.4 |
| 180 | *Cad* | Carbamoyl-phosphate synthetase 2, aspartate transcarbamylase, and dihydroorotase, mRNA (cDNA clone MGC:178528 IMAGE:9053520) | 2.05 |  |
| 181 | *Calca* | Calcitonin/calcitonin-related polypeptide, alpha, mRNA (cDNA clone MGC:41274 IMAGE:1399214) | 3.02 |  |
| 182 | *Calml4* | Calmodulin-like 4 (Calml4), transcript variant 1, mRNA | 3.89 |  |
| 183 | *Calr3* | Calreticulin 3 (Calr3), transcript variant 1, mRNA |  | 3.4 |
| 184 | *Camk2a* | Calcium/calmodulin-dependent protein kinase II alpha, mRNA (cDNA clone MGC:25415 IMAGE:4506005) | 3.16 |  |
| 185 | *Camk2b* | Calcium/calmodulin-dependent protein kinase II, beta (Camk2b), mRNA | 3.15 |  |
| 186 | *Cand2* | Cullin-associated and neddylation-dissociated 2 (putative) (Cand2), mRNA | 6.69 |  |
| 187 | *Cap2* | CAP, adenylate cyclase-associated protein, 2 (yeast) (Cap2), mRNA | 7.04 |  |
| 188 | *Capn6* | Calpain 6 (Capn6), mRNA | 12.18 |  |
| 189 | *Caprin2* | Caprin family member 2, mRNA (cDNA clone MGC:159076 IMAGE:40129888) |  | 2.07 |
| 190 | *Car6* | Carbonic anhydrase 6 (Car6), mRNA |  | 5.4 |
| 191 | *Carm1* | Coactivator-associated arginine methyltransferase 1, mRNA (cDNA clone MGC:46828 IMAGE:4935077) | 2.34 |  |
| 192 | *Casd1* | CAS1 domain containing 1, mRNA (cDNA clone IMAGE:2649724) | 2.44 |  |
| 193 | *Casp8ap2* | FLASH (Flash) | 2.5 |  |
| 194 | *Casq2* | Calsequestrin 2 (Casq2), mRNA | 6.36 |  |
| 195 | *Cav3* | Caveolin 3, mRNA (cDNA clone MGC:36120 IMAGE:4988598) | 6.9 |  |
| 196 | *Cbln2* | Cerebellin 2 precursor protein, mRNA (cDNA clone MGC:66500 IMAGE:6412317) | 8.19 |  |
| 197 | *Cbx5* | Chromobox homolog 5 (Drosophila HP1a), mRNA (cDNA clone MGC:5952 IMAGE:3499489) | 3.97 |  |
| 198 | *Ccbp2* | Chemokine binding protein 2 (Ccbp2), mRNA |  | 2.49 |
| 199 | *Ccdc126* | Coiled-coil domain containing 126, mRNA (cDNA clone MGC:143769 IMAGE:40093139) |  | 2.4 |
| 200 | *Ccdc18* | Coiled-coil domain containing 18 (Ccdc18), mRNA | 2.44 |  |
| 201 | *Ccdc21* | Coiled-coil domain containing 21, mRNA (cDNA clone MGC:25753 IMAGE:3992639) | 2.65 |  |
| 202 | *Ccdc43* | Coiled-coil domain containing 43 (Ccdc43), mRNA | 2.17 |  |
| 203 | *Ccdc46* | Coiled-coil domain containing 46 (Ccdc46), transcript variant 1, mRNA | 2 |  |
| 204 | *Cchcr1* | Coiled-coil alpha-helical rod protein 1 (Cchcr1), mRNA |  | 2.3 |
| 205 | *Ccnd2* | Cyclin D2 (Ccnd2), mRNA | 2.01 |  |
| 206 | *Ccnjl* | Cyclin J-like, mRNA (cDNA clone MGC:170971 IMAGE:8862366) | 3.16 |  |
| 207 | *Cd3e* | CD3 antigen, epsilon polypeptide (Cd3e), mRNA |  | 26.73 |
| 208 | *Cd55* | CD55 antigen, mRNA (cDNA clone MGC:18419 IMAGE:3663561) |  | 4.57 |
| 209 | *Cd59a* | CD59a antigen (Cd59a), transcript variant 2, mRNA |  | 2.42 |
| 210 | *Cdc42ep1* | CDC42 effector protein (Rho GTPase binding) 1 (Cdc42ep1), mRNA | 2.32 |  |
| 211 | *Cdca2* | Cell division cycle associated 2, mRNA (cDNA clone IMAGE:4954162) | 7.21 |  |
| 212 | *Cdh15* | Cadherin 15 (Cdh15), mRNA | 13.36 |  |
| 213 | *Cdkn1c* | Cyclin-dependent kinase inhibitor 1C (P57), mRNA (cDNA clone MGC:5658 IMAGE:3490380) | 6 |  |
| 214 | *Cdsn* | Corneodesmosin (Cdsn), mRNA |  | 2.31 |
| 215 | *Cenpc1* | Centromere protein C1 (Cenpc1), mRNA | 2.57 |  |
| 216 | *Cep170* | Centrosomal protein 170, mRNA (cDNA clone IMAGE:5357404) | 2.33 |  |
| 217 | *Cep68* | Centrosomal protein 68, mRNA (cDNA clone MGC:6990 IMAGE:3154964) | 2.17 |  |
| 218 | *Cep70* | Centrosomal protein 70 (Cep70), mRNA | 2.28 |  |
| 219 | *Cep76* | Centrosomal protein 76 (Cep76), mRNA | 2.15 |  |
| 220 | *Cfl2* | Cofilin 2, muscle, mRNA (cDNA clone MGC:6084 IMAGE:3590849) | 2.65 |  |
| 221 | *Cgn* | PREDICTED: Mus musculus cingulin (Cgn), mRNA |  | 3.44 |
| 222 | *Chd3* | Chromodomain helicase DNA binding protein 3 (Chd3), mRNA | 3.7 |  |
| 223 | *Chd6* | Chromodomain helicase DNA binding protein 6, mRNA (cDNA clone IMAGE:3597702) | 2.13 |  |
| 224 | *Chek1* | Checkpoint kinase 1 homolog (S. pombe), mRNA (cDNA clone IMAGE:5352918) | 4.25 |  |
| 225 | *Chn1* | Chimerin (chimaerin) 1, mRNA (cDNA clone MGC:39019 IMAGE:5364554) | 5.47 |  |
| 226 | *Chrna1* | Cholinergic receptor, nicotinic, alpha polypeptide 1 (muscle) (Chrna1), mRNA | 2.47 |  |
| 227 | *Chrng* | Cholinergic receptor, nicotinic, gamma polypeptide (Chrng), mRNA | 8.87 |  |
| 228 | *Chuk* | Conserved helix-loop-helix ubiquitous kinase, mRNA (cDNA clone MGC:25325 IMAGE:4511275) | 2.06 |  |
| 229 | *Cited1* | Cbp/p300-interacting transactivator with Glu/Asp-rich carboxy-terminal domain 1 (Cited1), mRNA | 10.85 |  |
| 230 | *Cited2* | Cbp/p300-interacting transactivator, with Glu/Asp-rich carboxy-terminal domain, 2 (Cited2), mRNA |  | 2.29 |
| 231 | *Cited4* | Cbp/p300-interacting transactivator, with Glu/Asp-rich carboxy-terminal domain, 4, mRNA (cDNA clone IMAGE:3670674) |  | 3.43 |
| 232 | *Clcn7* | Chloride channel 7, mRNA (cDNA clone IMAGE:3600566) |  | 2.41 |
| 233 | *Clec14a* | C-type lectin domain family 14, member a, mRNA (cDNA clone MGC:28590 IMAGE:4215467) | 4.76 |  |
| 234 | *Clpb* | ClpB caseinolytic peptidase B homolog (E. coli) (Clpb), mRNA | 2.45 |  |
| 235 | *Cmas* | Cytidine monophospho-N-acetylneuraminic acid synthetase (Cmas), mRNA | 2.05 |  |
| 236 | *Cnn3* | Calponin 3, acidic (Cnn3), mRNA |  | 4.03 |
| 237 | *Col16a1* | Collagen, type XVI, alpha 1, mRNA (cDNA clone IMAGE:5342460) |  | 2.12 |
| 238 | *Col23a1* | Collagen, type XXIII, alpha 1 (Col23a1), mRNA |  | 2.58 |
| 239 | *Col27a1* | Procollagen, type XXVII, alpha 1, mRNA (cDNA clone IMAGE:4194659) | 2.29 |  |
| 240 | *Col4a1* | Collagen, type IV, alpha 1, mRNA (cDNA clone IMAGE:3495898) | 2.56 |  |
| 241 | *Copa* | Coatomer protein complex subunit alpha, mRNA (cDNA clone MGC:37774 IMAGE:5097228) | 2.83 |  |
| 242 | *Cpm* | Carboxypeptidase M (Cpm), mRNA |  | 2.27 |
| 243 | *Cpne5* | Copine V (Cpne5), mRNA |  | 2.71 |
| 244 | *Cpxm2* | Carboxypeptidase X 2 (M14 family), mRNA (cDNA clone MGC:29082 IMAGE:5038528) |  | 2.55 |
| 245 | *Crbn* | Cereblon (Crbn), transcript variant 1, mRNA | 2.56 |  |
| 246 | *Creld1* | Cysteine-rich with EGF-like domains 1, mRNA (cDNA clone MGC:36401 IMAGE:5310111) |  | 2.19 |
| 247 | *Crispld1* | Cysteine-rich secretory protein LCCL domain containing 1, mRNA (cDNA clone IMAGE:5030052) | 3.03 |  |
| 248 | *Crlf1* | Cytokine receptor-like factor 1 (Crlf1), mRNA |  | 3.11 |
| 249 | *Crtc1* | CREB regulated transcription coactivator 1 (Crtc1), mRNA | 2.54 |  |
| 250 | *Cry1* | Cryptochrome 1 (photolyase-like), mRNA (cDNA clone MGC:13933 IMAGE:4036758) | 2.63 |  |
| 251 | *Cry2* | Cryptochrome 2 (photolyase-like), mRNA (cDNA clone IMAGE:3593948) |  | 2.81 |
| 252 | *Cse1l* | Chromosome segregation 1-like (S. cerevisiae) (Cse1l), mRNA | 2.27 |  |
| 253 | *Cst7* | Cystatin F (leukocystatin) (Cst7), mRNA |  | 2.97 |
| 254 | *Ctgf* | Connective tissue growth factor, mRNA (cDNA clone MGC:8122 IMAGE:3589136) |  | 4.62 |
| 255 | *Ctla2a* | Cytotoxic T lymphocyte-associated protein 2 alpha, mRNA (cDNA clone MGC:41186 IMAGE:1244311) |  | 2.68 |
| 256 | *Ctla4* | Cytotoxic T-lymphocyte-associated protein 4 (Ctla4), mRNA |  | 3.17 |
| 257 | *Ctr9* | Ctr9, Paf1/RNA polymerase II complex component, homolog (S. cerevisiae) (Ctr9), mRNA | 2 |  |
| 258 | *Cul7* | Cullin 7, mRNA (cDNA clone IMAGE:4022086) | 3.23 |  |
| 259 | *Cutc* | CutC copper transporter homolog (E.coli), mRNA (cDNA clone MGC:25524 IMAGE:3582902) | 2.53 |  |
| 260 | *Cyp39a1* | Cytochrome P450, family 39, subfamily a, polypeptide 1, mRNA (cDNA clone MGC:46855 IMAGE:5352740) |  | 2.33 |
| 261 | *Cyp3a13* | Cytochrome P450, family 3, subfamily a, polypeptide 13 (Cyp3a13), mRNA |  | 2.06 |
| 262 | *Cyp4v3* | Kallikrein B, plasma 1, mRNA (cDNA clone MGC:36124 IMAGE:4972788) |  | 2.84 |
| 263 | *Cyr61* | Cysteine rich protein 61 (Cyr61), mRNA | 2.98 |  |
| 264 | *D0H4S114* | DNA segment, human D4S114 (D0H4S114), transcript variant 1, mRNA | 4.82 |  |
| 265 | *D10Ertd322e* | DNA segment, Chr 10, ERATO Doi 322, expressed, mRNA (cDNA clone MGC:35676 IMAGE:4984291) | 2.24 |  |
| 266 | *D10Ertd610e* | DNA segment, Chr 10, ERATO Doi 610, expressed, mRNA (cDNA clone IMAGE:5027413) | 2.45 |  |
| 267 | *D930015E06Rik* | RIKEN cDNA D930015E06 gene, mRNA (cDNA clone IMAGE:4947647) |  | 2.19 |
| 268 | *D930020B18Rik* | RIKEN cDNA D930020B18 gene (D930020B18Rik), mRNA |  | 9.77 |
| 269 | *Daam2* | Dishevelled associated activator of morphogenesis 2 (Daam2), mRNA | 2.73 |  |
| 270 | *Dach1* | DACH protein (Dach) | 3.11 |  |
| 271 | *Daglb* | Diacylglycerol lipase, beta (Daglb), mRNA |  | 4.65 |
| 272 | *Dbc1* | BRINP mRNA for BMP/retinoic acid-inducible neural-specific protein (BRINP) | 3.74 |  |
| 273 | *Dbf4* | DBF4 homolog (S. cerevisiae) (Dbf4), mRNA | 3.16 |  |
| 274 | *Dbp* | D site albumin promoter binding protein, mRNA (cDNA clone MGC:18803 IMAGE:4195116) |  | 3.12 |
| 275 | *Ddah2* | Dimethylarginine dimethylaminohydrolase 2, mRNA (cDNA clone MGC:5866 IMAGE:3158175) | 6.4 |  |
| 276 | *Ddc* | Dopa decarboxylase (Ddc), mRNA | 5.03 |  |
| 277 | *Ddit4l* | DNA-damage-inducible transcript 4-like, mRNA (cDNA clone IMAGE:5254530) | 2.82 |  |
| 278 | *Ddx42* | DEAD (Asp-Glu-Ala-Asp) box polypeptide 42 (Ddx42), mRNA | 2.27 |  |
| 279 | *Dennd2a* | DENN/MADD domain containing 2A, mRNA (cDNA clone MGC:183710 IMAGE:9087710) | 3.48 |  |
| 280 | *Dgat2* | Diacylglycerol O-acyltransferase 2 (Dgat2), mRNA |  | 2.48 |
| 281 | *Dhodh* | Dihydroorotate dehydrogenase, mRNA (cDNA clone MGC:35899 IMAGE:5253723) | 2.05 |  |
| 282 | *Dixdc1* | DIX domain containing 1, mRNA (cDNA clone IMAGE:4239578) | 2.15 |  |
| 283 | *Dkk1* | Dickkopf homolog 1 (Xenopus laevis) (Dkk1), mRNA | 8.22 |  |
| 284 | *Dld* | Dihydrolipoamide dehydrogenase, mRNA (cDNA clone MGC:5874 IMAGE:3589344) | 2.08 |  |
| 285 | *Dlk1* | Delta-like 1 homolog (Drosophila) (Dlk1), mRNA | 9.01 |  |
| 286 | *Dll4* | Dll-4 mRNA for Delta-4 | 3.18 |  |
| 287 | *Dlst* | Dihydrolipoamide S-succinyltransferase (E2 component of 2-oxo-glutarate complex), mRNA (cDNA clone IMAGE:5068310) | 2.03 |  |
| 288 | *Dlx5* | Homeodomain protein DLX5 variant (Dlx5) |  | 3.07 |
| 289 | *Dmkn* | Dermokine (Dmkn), transcript variant 1, mRNA |  | 2.06 |
| 290 | *Dmrt2* | Doublesex and mab-3 related transcription factor 2, mRNA (cDNA clone MGC:41586 IMAGE:1248080) | 5.35 |  |
| 291 | *Dnajc12* | DnaJ (Hsp40) homolog, subfamily C, member 12, mRNA (cDNA clone MGC:19166 IMAGE:4222828) | 2.91 |  |
| 292 | *Dnajc7* | DnaJ (Hsp40) homolog, subfamily C, member 7, mRNA (cDNA clone MGC:35912 IMAGE:5343384) | 2.05 |  |
| 293 | *Dnmt1* | DNA (cytosine-5)-methyltransferase (Dnmt1) | 2.67 |  |
| 294 | *Dnmt3a* | DNA methyltransferase 3A (Dnmt3a), transcript variant 2, mRNA | 3.23 |  |
| 295 | *Dok5* | Docking protein 5 (Dok5), mRNA | 5.97 |  |
| 296 | *Dtna* | Alpha-dystrobrevin 2b | 4.47 |  |
| 297 | *Dusp27* | Dual specificity phosphatase 27 (putative), mRNA (cDNA clone MGC:144106 IMAGE:40097789) | 6.24 |  |
| 298 | *Dzip1* | DAZ interacting protein 1, mRNA (cDNA clone MGC:106777 IMAGE:6825425) | 2.13 |  |
| 299 | *E2f1* | Strain ILS E2F1 | 2.92 |  |
| 300 | *E2f7* | E2F transcription factor 7, mRNA (cDNA clone MGC:178995 IMAGE:9053987) | 3.62 |  |
| 301 | *Ebf3* | Early B-cell factor 3 (Ebf3), transcript variant 3, mRNA | 6.34 |  |
| 302 | *Ebpl* | Emopamil binding protein-like, mRNA (cDNA clone MGC:35635 IMAGE:4035224) | 2.02 |  |
| 303 | *Echdc3* | Enoyl Coenzyme A hydratase domain containing 3 (Echdc3), mRNA |  | 3.92 |
| 304 | *Echs1* | Enoyl Coenzyme A hydratase, short chain, 1, mitochondrial, mRNA (cDNA clone IMAGE:3487040) | 2.08 |  |
| 305 | *Edil3* | Del1 minor splice variant (Del1) | 3.93 |  |
| 306 | *Efcab7* | EF-hand calcium binding domain 7 (Efcab7), mRNA | 2 |  |
| 307 | *Efemp1* | Epidermal growth factor-containing fibulin-like extracellular matrix protein 1 (Efemp1), mRNA |  | 4.02 |
| 308 | *Efna2* | Ephrin A2 (Efna2), mRNA | 2.41 |  |
| 309 | *EG545802* | PREDICTED: Mus musculus predicted gene, EG545802 (EG545802), mRNA |  | 3.44 |
| 310 | *EG638833* | PREDICTED: Mus musculus predicted gene, EG638833 (EG638833), mRNA | 4.78 |  |
| 311 | *Egfl6* | EGF-like-domain, multiple 6 (Egfl6), mRNA | 7.52 |  |
| 312 | *Eif3g* | Eukaryotic translation initiation factor 3, subunit G, mRNA (cDNA clone MGC:5726 IMAGE:3592537) | 3.7 |  |
| 313 | *Eif4a3* | Eukaryotic translation initiation factor 4A, isoform 3 (Eif4a3), mRNA | 2.28 |  |
| 314 | *Eif4g1* | Eukaryotic translation initiation factor 4, gamma 1 (Eif4g1), transcript variant 1, mRNA | 2.52 |  |
| 315 | *Ell3* | Elongation factor RNA polymerase II-like 3 (Ell3), mRNA |  | 2.2 |
| 316 | *Elovl2* | Elongation of very long chain fatty acids (FEN1/Elo2, SUR4/Elo3, yeast)-like 2 (Elovl2), mRNA | 4.23 |  |
| 317 | *Elp2* | Elongation protein 2 homolog (S. cerevisiae), mRNA (cDNA clone MGC:18889 IMAGE:4239056) | 2.34 |  |
| 318 | *Emr4* | EGF-like module containing, mucin-like, hormone receptor-like sequence 4 (Emr4), mRNA |  | 6.41 |
| 319 | *Enho* | Energy homeostasis associated, mRNA (cDNA clone IMAGE:5097248) | 5.25 |  |
| 320 | *Eno3* | Enolase 3, beta muscle, mRNA (cDNA clone MGC:18648 IMAGE:4217678) | 8.83 |  |
| 321 | *Enpp5* | Ectonucleotide pyrophosphatase/phosphodiesterase 5 (Enpp5), mRNA |  | 4.16 |
| 322 | *Entpd6* | Ectonucleoside triphosphate diphosphohydrolase 6, mRNA (cDNA clone MGC:47939 IMAGE:1348160) | 2.31 |  |
| 323 | *Epb4.1l3* | Protein 4.1B (Epb4.1l3) | 2.88 |  |
| 324 | *Epb4.1l4b* | Erythrocyte protein band 4.1-like 4b (Epb4.1l4b), mRNA |  | 2.11 |
| 325 | *Epha7* | Eph receptor A7, mRNA (cDNA clone MGC:14056 IMAGE:3991628) | 3.62 |  |
| 326 | *Ephb2* | Sek-3 mRNA | 3.15 |  |
| 327 | *Ephb3* | Eph receptor B3, mRNA (cDNA clone MGC:18409 IMAGE:3673003) | 2.21 |  |
| 328 | *Epm2aip1* | EPM2A (laforin) interacting protein 1, mRNA (cDNA clone MGC:27780 IMAGE:3156440) | 2.29 |  |
| 329 | *Ergic1* | Endoplasmic reticulum-golgi intermediate compartment (ERGIC) 1, mRNA (cDNA clone MGC:7601 IMAGE:3494155) | 2.41 |  |
| 330 | *Esrrb* | Estrogen related receptor, beta (Esrrb), mRNA | 2.51 |  |
| 331 | *Etnk1* | Ethanolamine kinase 1, mRNA (cDNA clone IMAGE:5320201) |  | 2.17 |
| 332 | *Evi5l* | Ecotropic viral integration site 5 like (Evi5l), mRNA |  | 2.05 |
| 333 | *Exd2* | Exonuclease 3-5 domain containing 2 (Exd2), mRNA | 3.04 |  |
| 334 | *Ext2* | Exostoses (multiple) 2, mRNA (cDNA clone MGC:11478 IMAGE:3965079) |  | 2.09 |
| 335 | *Eya1* | Eyes absent 1 homolog (Drosophila) (Eya1), mRNA | 3.02 |  |
| 336 | *F3* | Coagulation factor III, mRNA (cDNA clone MGC:18513 IMAGE:3591823) |  | 4.83 |
| 337 | *Fam101b* | PREDICTED: Mus musculus RIKEN cDNA 1500005K14 gene (1500005K14Rik), mRNA | 2.58 |  |
| 338 | *Fam110b* | Family with sequence similarity 110, member B, mRNA (cDNA clone MGC:66511 IMAGE:6414160) | 4.21 |  |
| 339 | *Fam122b* | Family with sequence similarity 122, member B (Fam122b), mRNA | 3.11 |  |
| 340 | *Fam13c* | Family with sequence similarity 13, member C, mRNA (cDNA clone IMAGE:3483802) | 2.02 |  |
| 341 | *Fam171a1* | Family with sequence similarity 171, member A1, mRNA (cDNA clone IMAGE:5375863) | 2.51 |  |
| 342 | *Fam171a2* | Family with sequence similarity 171, member A2 (Fam171a2), mRNA | 3.9 |  |
| 343 | *Fam62b* | Family with sequence similarity 62, member B (Fam62b), mRNA |  | 2.05 |
| 344 | *Fam70a* | Family with sequence similarity 70, member A, mRNA (cDNA clone MGC:86163 IMAGE:30550174) | 4.17 |  |
| 345 | *Fam73a* | Family with sequence similarity 73, member A, mRNA (cDNA clone MGC:169953 IMAGE:8861348) | 2.75 |  |
| 346 | *Fat3* | FAT tumor suppressor homolog 3 (Drosophila) (Fat3), mRNA | 2.27 |  |
| 347 | *Fat4* | Fat4 | 3.73 |  |
| 348 | *Fbn2* | Fibrillin 2, mRNA (cDNA clone IMAGE:1498131) | 2.27 |  |
| 349 | *Fbxl12* | F-box and leucine-rich repeat protein 12 (Fbxl12), transcript variant 2, mRNA | 2.43 |  |
| 350 | *Fbxl19* | F-box and leucine-rich repeat protein 19, mRNA (cDNA clone MGC:69570 IMAGE:6401846) | 3.57 |  |
| 351 | *Fbxl7* | F-box and leucine-rich repeat protein 7, mRNA (cDNA clone MGC:102204 IMAGE:30355382) | 2.46 |  |
| 352 | *Fbxo18* | F-box protein 18 (Fbxo18), mRNA | 2.41 |  |
| 353 | *Fbxo32* | F-box protein 32, mRNA (cDNA clone MGC:25730 IMAGE:3981597) |  | 2.45 |
| 354 | *Fem1a* | Feminization 1 homolog a (C. elegans), mRNA (cDNA clone MGC:6309 IMAGE:2811079) | 2.64 |  |
| 355 | *Ffar2* | Free fatty acid receptor 2 (Ffar2), mRNA |  | 2.01 |
| 356 | *Fgf13* | Fibroblast growth factor 13, mRNA (cDNA clone MGC:25467 IMAGE:4481123) | 3.83 | 2.88 |
| 357 | *Fgfrl1* | Fibroblast growth factor receptor-like 1 (Fgfrl1), mRNA | 2.48 |  |
| 358 | *Fh1* | Fumarate hydratase 1, mRNA (cDNA clone IMAGE:3594292) | 3.07 |  |
| 359 | *Fhl1* | Four and a half LIM domains 1, mRNA (cDNA clone MGC:32284 IMAGE:5012394) | 4.16 |  |
| 360 | *Fibin* | Fin bud initiation factor homolog (zebrafish), mRNA (cDNA clone MGC:29048 IMAGE:3603588) | 2.36 |  |
| 361 | *Fkbp5* | FK506 binding protein 5, mRNA (cDNA clone MGC:18417 IMAGE:4237766) | 2.09 |  |
| 362 | *Flnc* | Strain C3H filamin | 3.52 |  |
| 363 | *Flot2* | Flotillin 2 (Flot2), transcript variant 2, mRNA | 2.44 |  |
| 364 | *Fmod* | Fibromodulin (Fmod), mRNA | 3.7 |  |
| 365 | *Fndc3c1* | Fibronectin domain-containing protein 3c (Fndc3c) | 12.91 |  |
| 366 | *Fndc5* | Peroxisomal protein (PeP gene) | 5.11 |  |
| 367 | *Foxo6* | Forkhead box O6, mRNA (cDNA clone MGC:73469 IMAGE:6810803) |  | 2.67 |
| 368 | *Freq* | Frequenin homolog (Drosophila) (Freq), mRNA | 2.34 |  |
| 369 | *Frzb* | Frizzled-related protein, mRNA (cDNA clone MGC:18388 IMAGE:4237554) | 5.08 |  |
| 370 | *Fut10* | Fucosyltransferase 10, mRNA (cDNA clone MGC:40819 IMAGE:5368333) | 9.35 |  |
| 371 | *Fut8* | Fucosyltransferase 8, mRNA (cDNA clone MGC:11418 IMAGE:3594582) |  | 2.17 |
| 372 | *Fxn* | Frataxin, mRNA (cDNA clone IMAGE:2651152) | 2.83 |  |
| 373 | *Gaa* | Glucosidase, alpha, acid, mRNA (cDNA clone MGC:11484 IMAGE:3969127) | 2.71 |  |
| 374 | *Gabrb3* | Gamma-aminobutyric acid (GABA-A) receptor, subunit beta 3 (Gabrb3), transcript variant 1, mRNA | 2.39 |  |
| 375 | *Gadl1* | Glutamate decarboxylase-like 1 (Gadl1), mRNA |  | 3.26 |
| 376 | *Gap43* | Growth associated protein 43, mRNA (cDNA clone MGC:25402 IMAGE:4503501) | 13.81 |  |
| 377 | *Gas6* | Growth arrest specific 6, mRNA (cDNA clone MGC:6124 IMAGE:3592398) |  | 2.78 |
| 378 | *Gata2* | GATA binding protein 2 (Gata2), mRNA | 3.19 |  |
| 379 | *Gatm* | Glycine amidinotransferase (L-arginine:glycine amidinotransferase), mRNA (cDNA clone MGC:6646 IMAGE:3495871) | 2.08 |  |
| 380 | *Gcn1l1* | GCN1 general control of amino-acid synthesis 1-like 1 (yeast), mRNA (cDNA clone IMAGE:4241519) | 3.22 |  |
| 381 | *Gdf10* | Growth differentiation factor 10 (Gdf10), mRNA | 2.85 |  |
| 382 | *Gdpd2* | Glycerophosphodiester phosphodiesterase domain containing 2, mRNA (cDNA clone MGC:46808 IMAGE:4457335) |  | 4.05 |
| 383 | *Gdpd5* | Glycerophosphodiester phosphodiesterase domain containing 5, mRNA (cDNA clone MGC:37148 IMAGE:4953174) | 3.08 |  |
| 384 | *Gen1* | Gen homolog 1, endonuclease (Drosophila), mRNA (cDNA clone MGC:169782 IMAGE:8861177) | 2.92 |  |
| 385 | *Gja5* | Gap junction membrane channel protein alpha 5 (Gja5), mRNA | 2.86 |  |
| 386 | *Gjb5* | Gap junction protein, beta 5, mRNA (cDNA clone MGC:18579 IMAGE:4207506) |  | 2.99 |
| 387 | *Gjc1* | Gap junction protein, gamma 1 (Gjc1), mRNA | 2.43 |  |
| 388 | *Glg1* | Golgi apparatus protein 1, mRNA (cDNA clone MGC:29292 IMAGE:4239405) | 2.08 |  |
| 389 | *Gm347* | Gene model 347, (NCBI) (Gm347), mRNA | 3.14 |  |
| 390 | *Gm94* | Gene model 94, (NCBI), mRNA (cDNA clone MGC:198700 IMAGE:9054281) |  | 2.31 |
| 391 | *Gna14* | Guanine nucleotide binding protein, alpha 14, mRNA (cDNA clone MGC:36137 IMAGE:5375817) |  | 3.61 |
| 392 | *Gnas* | GNAS (guanine nucleotide binding protein, alpha stimulating) complex locus, mRNA (cDNA clone IMAGE:4013293) | 4.48 |  |
| 393 | *Gnaz* | Guanine nucleotide binding protein, alpha z subunit, mRNA (cDNA clone MGC:25486 IMAGE:4501587) | 5.1 |  |
| 394 | *Gnb4* | CDNA fis, clone TRACH2010883,highly similar to GUANINE NUCLEOTIDE-BINDING PROTEIN BETA SUBUNIT 4 | 3.92 |  |
| 395 | *Gng13* | G protein gamma subunit 13 |  | 12.9 |
| 396 | *Gpc6* | Glypican 6, mRNA (cDNA clone MGC:32221 IMAGE:5008374) |  | 3.74 |
| 397 | *Gpr126* | G protein-coupled receptor 126, mRNA (cDNA clone IMAGE:30437066) |  | 2.52 |
| 398 | *Gpr146* | G protein-coupled receptor 146, mRNA (cDNA clone MGC:7035 IMAGE:3156060) |  | 2.58 |
| 399 | *Gpr64* | Me6 receptor long splice variant mRNA, complete cds; alternatively spliced |  | 2.02 |
| 400 | *Gprasp1* | G protein-coupled receptor associated sorting protein 1 (Gprasp1), transcript variant 1, mRNA | 6.26 |  |
| 401 | *Gprasp2* | PREDICTED: Mus musculus G protein-coupled receptor associated sorting protein 2 (Gprasp2), mRNA | 6.47 |  |
| 402 | *Grb10* | Growth factor receptor bound protein 10, mRNA (cDNA clone IMAGE:1313582) | 6.89 |  |
| 403 | *Gsr* | Glutathione reductase, mRNA (cDNA clone IMAGE:3589654) |  | 2.1 |
| 404 | *Gstt2* | Glutathione S-transferase, theta 2, mRNA (cDNA clone MGC:13991 IMAGE:3994154) |  | 2.17 |
| 405 | *Gtdc1* | Glycosyltransferase-like domain containing 1, mRNA (cDNA clone MGC:62476 IMAGE:5715781) | 2.19 |  |
| 406 | *Gtpbp3* | GTP binding protein 3 (Gtpbp3), nuclear gene encoding mitochondrial protein, mRNA | 2.2 |  |
| 407 | *Gucy1a3* | Guanylate cyclase 1, soluble, alpha 3 (Gucy1a3), mRNA | 2.23 |  |
| 408 | *Gyg* | Glycogenin, mRNA (cDNA clone MGC:35999 IMAGE:4487769) | 2.42 |  |
| 409 | *Gypa* | Glycophorin A (Gypa), mRNA | 12.94 |  |
| 410 | *H19* | H19 fetal liver mRNA, mRNA (cDNA clone IMAGE:3592592) | 18.16 |  |
| 411 | *H2-M3* | Histocompatibility 2, M region locus 3, mRNA (cDNA clone MGC:18978 IMAGE:4007538) |  | 2.55 |
| 412 | *Hapln1* | Cartilage link protein (Crtl1) | 8.49 |  |
| 413 | *Has2* | Hyaluronan synthase 2 (Has2), mRNA | 2.89 |  |
| 414 | *Hba-a1* | Hemoglobin alpha, adult chain 1 (Hba-a1), mRNA | 5.87 |  |
| 415 | *Hcfc1* | B2 repetitive sequence, 5 end | 2.32 |  |
| 416 | *Hcfc2* | Host cell factor C2 (Hcfc2), mRNA | 2.01 |  |
| 417 | *Hdac5* | Histone deacetylase 5, mRNA (cDNA clone IMAGE:4458013) |  | 2.45 |
| 418 | *Heatr2* | HEAT repeat containing 2, mRNA (cDNA clone MGC:182395 IMAGE:9056289) |  | 8.19 |
| 419 | *Heatr5b* | HEAT repeat containing 5B, mRNA (cDNA clone IMAGE:4237004) | 2.35 |  |
| 420 | *Hectd2* | HECT domain containing 2, mRNA (cDNA clone MGC:178371 IMAGE:9053363) | 2.1 |  |
| 421 | *Hemk1* | HemK methyltransferase family member 1 (Hemk1), mRNA | 2.3 |  |
| 422 | *Herc3* | Hect domain and RLD 3 (Herc3), mRNA | 2.08 |  |
| 423 | *Herpud1* | Adrenergic receptor kinase, beta 2 (Adrbk2), transcript variant 2, mRNA |  | 2 |
| 424 | *Hes5* | Hairy and enhancer of split 5 (Drosophila) (Hes5), mRNA | 10.26 |  |
| 425 | *Hexdc* | Hexosaminidase (glycosyl hydrolase family 20, catalytic domain) containing (Hexdc), transcript variant 1, mRNA |  | 2.06 |
| 426 | *Hey2* | HES-related repressor protein 1 HERP1 | 2.39 |  |
| 427 | *Hiat1* | Hippocampus abundant gene transcript 1 (Hiat1), mRNA | 2.05 |  |
| 428 | *Hic2* | Hypermethylated in cancer 2 protein (Hic2) | 2.22 |  |
| 429 | *Hip1* | Huntingtin interacting protein 1 (Hip1), mRNA | 2.66 |  |
| 430 | *Hist1h4d* | Histone cluster 1, H4d, mRNA (cDNA clone IMAGE:40061655) |  | 3.62 |
| 431 | *Hmgxb4* | HMG box domain containing 4, mRNA (cDNA clone IMAGE:4948318) | 2.11 |  |
| 432 | *Hn1l* | Hematological and neurological expressed 1-like, mRNA (cDNA clone MGC:91108 IMAGE:30460749) | 2.17 |  |
| 433 | *Hnmt* | Histamine N-methyltransferase, mRNA (cDNA clone MGC:41227 IMAGE:1546890) |  | 2.85 |
| 434 | *Hnrnpd* | Heterogeneous nuclear ribonucleoprotein D, mRNA (cDNA clone IMAGE:4187388) | 2.68 |  |
| 435 | *Homer1* | Homer homolog 1 (Drosophila) (Homer1), transcript variant L, mRNA | 2.06 |  |
| 436 | *Hoxa1* | Homeo box A1 (Hoxa1), mRNA | 2.95 |  |
| 437 | *Hoxa10* | Homeo box A10 (Hoxa10), transcript variant 1, mRNA | 26.66 |  |
| 438 | *Hoxa4* | Hox-1.4 protein | 6.85 |  |
| 439 | *Hoxa5* | Homeo box A5, mRNA (cDNA clone MGC:18622 IMAGE:3985274) | 5.25 |  |
| 440 | *Hoxa7* | Homeo box A7, mRNA (cDNA clone IMAGE:4986801) | 3.86 |  |
| 441 | *Hoxb4* | Homeo box B4 (Hoxb4), mRNA | 3.18 |  |
| 442 | *Hoxb8* | Homeo box B8 (Hoxb8), mRNA | 6.37 |  |
| 443 | *Hoxb9* | Hox-2.5 mRNA | 7.28 |  |
| 444 | *Hoxd9* | Homeo box D9, mRNA (cDNA clone MGC:29423 IMAGE:5064578) | 2.37 |  |
| 445 | *Hs6st2* | Heparan sulfate 6-O-sulfotransferase 2, mRNA (cDNA clone IMAGE:5356211) | 3.71 |  |
| 446 | *Hsd3b2* | Hydroxy-delta-5-steroid dehydrogenase, 3 beta- and steroid delta-isomerase 2 (Hsd3b2), mRNA |  | 2.5 |
| 447 | *Hsd3b7* | Hydroxy-delta-5-steroid dehydrogenase, 3 beta- and steroid delta-isomerase 7 (Hsd3b7), transcript variant 1, mRNA |  | 2.58 |
| 448 | *Hsdl1* | Hydroxysteroid dehydrogenase like 1, mRNA (cDNA clone MGC:86138 IMAGE:6809862) |  | 2.01 |
| 449 | *Hsf2* | Heat shock factor 2, mRNA (cDNA clone MGC:25434 IMAGE:3990765) |  | 2.3 |
| 450 | *Hspa12a* | Heat shock protein 12A, mRNA (cDNA clone MGC:40846 IMAGE:5369051) | 2.05 | 3.2 |
| 451 | *Hspa12b* | Heat shock protein 12B (Hspa12b), mRNA | 2.39 |  |
| 452 | *Hspa2* | Heat shock protein 2, mRNA (cDNA clone MGC:7795 IMAGE:3499661) |  | 3.13 |
| 453 | *Hspb3* | Heat shock protein 3 (Hspb3), mRNA | 10.43 |  |
| 454 | *Hspb7* | Cardiovascular heat shock protein | 4.1 |  |
| 455 | *Ifngr1* | Strain SJL/J interferon gamma receptor (Ifngr) |  | 2.7 |
| 456 | *Ift172* | Intraflagellar transport 172 homolog (Chlamydomonas) (Ift172), mRNA | 3.16 |  |
| 457 | *Igdcc4* | DDM36E | 2.62 |  |
| 458 | *Igf2* | Insulin-like growth factor 2 (Igf2), transcript variant 1, mRNA | 21.43 |  |
| 459 | *Igf2as* | Peg8/Igf2as mRNA, imprinting gene | 5.29 |  |
| 460 | *Igf2bp1* | Insulin-like growth factor 2 mRNA binding protein 1 (Igf2bp1), mRNA | 3.97 |  |
| 461 | *Igf2bp2* | RIKEN cDNA C330012H03 gene, mRNA (cDNA clone IMAGE:5354659) | 7.76 |  |
| 462 | *Igf2bp3* | Insulin-like growth factor 2 mRNA binding protein 3 (Igf2bp3), mRNA | 11.03 |  |
| 463 | *Igf2r* | Insulin-like growth factor 2 receptor (Igf2r), mRNA | 2.43 |  |
| 464 | *Igfbp3* | Insulin-like growth factor binding protein 3 (Igfbp3), mRNA |  | 4.83 |
| 465 | *Igsf1* | CDNA clone IMAGE:9053310 | 5.23 |  |
| 466 | *Igsf11* | Immunoglobulin superfamily, member 11 (Igsf11), mRNA | 3.3 |  |
| 467 | *Il17d* | Interleukin 17D (Il17d), mRNA | 2.02 |  |
| 468 | *Il20ra* | Interleukin 20 receptor, alpha, mRNA (cDNA clone MGC:67678 IMAGE:5033177) |  | 3.66 |
| 469 | *Il22ra2* | CDNA clone IMAGE:40044751 |  | 4.63 |
| 470 | *Il33* | Interleukin 33 (Il33), mRNA |  | 2.31 |
| 471 | *Il34* | Interleukin 34, mRNA (cDNA clone MGC:28891 IMAGE:4912097) |  | 2.59 |
| 472 | *Inppl1* | SH2-containing inositol 5-phosphatase 2 | 3.48 |  |
| 473 | *Iqcb1* | IQ calmodulin-binding motif containing 1, mRNA (cDNA clone MGC:27790 IMAGE:3157011) | 2.54 |  |
| 474 | *Irf4* | Interferon regulatory factor 4 (Irf4), mRNA |  | 2.67 |
| 475 | *Irf5* | Interferon regulatory factor 5 (Irf5), mRNA | 2.15 |  |
| 476 | *Isl1* | ISL1 transcription factor, LIM/homeodomain (Isl1), mRNA | 3.72 |  |
| 477 | *Ism1* | Isthmin 1 homolog (zebrafish) (Ism1), mRNA |  | 6.84 |
| 478 | *Itga5* | Integrin alpha 5 subunit | 2.01 |  |
| 479 | *Itga6* | Integrin alpha 6, mRNA (cDNA clone IMAGE:5355083) | 3.25 |  |
| 480 | *Itm2a* | Integral membrane protein 2A, mRNA (cDNA clone MGC:18323 IMAGE:3668557) | 6.1 |  |
| 481 | *Itpr3* | Inositol 1,4,5-triphosphate receptor 3, mRNA (cDNA clone IMAGE:5345968) |  | 2.51 |
| 482 | *Itsn1* | Intersectin short from (Itsn) | 2.78 |  |
| 483 | *Kank1* | Ankyrin repeat domain 15, mRNA (cDNA clone IMAGE:4220878) |  | 3.41 |
| 484 | *Kbtbd10* | Kelch repeat and BTB (POZ) domain containing 10, mRNA (cDNA clone MGC:183609 IMAGE:9087609) | 12.07 |  |
| 485 | *Kbtbd5* | Kelch repeat and BTB (POZ) domain containing 5, mRNA (cDNA clone MGC:169513 IMAGE:8860908) | 4.52 |  |
| 486 | *Kcnj12* | Potassium inwardly-rectifying channel, subfamily J, member 12 (Kcnj12), mRNA | 4.72 |  |
| 487 | *Kcnj15* | Potassium inwardly rectifying channel (Kir4.2 gene and 3 UTR |  | 2 |
| 488 | *Kcnj16* | Potassium inwardly-rectifying channel, subfamily J, member 16, mRNA (cDNA clone MGC:28639 IMAGE:4223640) |  | 3.02 |
| 489 | *Kctd12* | Potassium channel tetramerisation domain containing 12, mRNA (cDNA clone IMAGE:40086280) |  | 2.86 |
| 490 | *Kdm3b* | Jumonji domain containing 1B, mRNA (cDNA clone IMAGE:5360429) |  | 2.05 |
| 491 | *Kdr* | Kinase insert domain protein receptor, mRNA (cDNA clone MGC:18600 IMAGE:4238984) | 4.76 |  |
| 492 | *Kif22* | Kinesin family member 22 (Kif22), mRNA | 4.53 |  |
| 493 | *Kif2a* | Kinesin family member 2A, mRNA (cDNA clone MGC:11609 IMAGE:3152883) | 2.39 |  |
| 494 | *Kifap3* | Kinesin-associated protein 3 (Kifap3), mRNA | 2.55 |  |
| 495 | *Kin* | Antigenic determinant of rec-A protein, mRNA (cDNA clone MGC:18413 IMAGE:3673830) | 2.59 |  |
| 496 | *Klf12* | Kruppel-like factor 12 (Klf12), mRNA | 2.99 |  |
| 497 | *Klhl13* | Kelch-like 13 (Drosophila) (Klhl13), mRNA | 2.34 |  |
| 498 | *Klhl17* | Kelch-like 17 (Drosophila), mRNA (cDNA clone MGC:170936 IMAGE:8862331) |  | 2.01 |
| 499 | *Klhl23* | Kelch-like 23 (Drosophila), mRNA (cDNA clone MGC:99858 IMAGE:6827841) | 2.93 |  |
| 500 | *Klhl30* | Kelch-like 30 (Drosophila), mRNA (cDNA clone MGC:143857 IMAGE:40094088) | 2.91 |  |
| 501 | *Klhl7* | Kelch-like 7 (Drosophila) (Klhl7), mRNA | 2.24 |  |
| 502 | *Klhl9* | Kelch-like 9 (Drosophila), mRNA (cDNA clone MGC:106634 IMAGE:6811241) | 2.06 |  |
| 503 | *Kpna3* | Karyopherin (importin) alpha 3, mRNA (cDNA clone MGC:30454 IMAGE:3979788) | 2.13 |  |
| 504 | *Krba1* | KRAB-A domain containing 1 (Krba1), mRNA | 2.49 |  |
| 505 | *Krt18* | Keratin 18 (Krt18), mRNA | 8.33 |  |
| 506 | *Krt32* | Keratin 32 (Krt32), mRNA |  | 3.52 |
| 507 | *Krtap3-2* | Keratin associated protein 3-2 (Krtap3-2), mRNA |  | 2.51 |
| 508 | *L2hgdh* | L-2-hydroxyglutarate dehydrogenase (L2hgdh), nuclear gene encoding mitochondrial protein, mRNA | 2.41 |  |
| 509 | *Lamc1* | Laminin B2 chain | 2.3 |  |
| 510 | *Laptm4b* | Lysosomal-associated protein transmembrane 4B, mRNA (cDNA clone MGC:29407 IMAGE:4507607) | 2.01 |  |
| 511 | *Lba1* | PREDICTED: Mus musculus lupus brain antigen 1 (Lba1), mRNA | 3.82 |  |
| 512 | *Lca5* | Leber congenital amaurosis 5 (human) (Lca5), transcript variant 1, mRNA | 2.71 |  |
| 513 | *Lce1m* | Late cornified envelope 1M (Lce1m), mRNA |  | 3.04 |
| 514 | *Ldb2* | LIM domain binding 2 (Ldb2), transcript variant 1, mRNA | 2.16 |  |
| 515 | *Lefty1* | Left right determination factor 1 (Lefty1), mRNA |  | 2.47 |
| 516 | *Lhx6* | LIM homeobox protein 6 (Lhx6), transcript variant 1, mRNA | 2.81 |  |
| 517 | *Lig1* | Ligase I, DNA, ATP-dependent, mRNA (cDNA clone MGC:25340 IMAGE:4911261) | 3.08 |  |
| 518 | *Limch1* | LIM and calponin homology domains 1 (Limch1), mRNA | 3.11 |  |
| 519 | *Lime1* | Lck interacting transmembrane adaptor 1, mRNA (cDNA clone MGC:35801 IMAGE:5357176) | 3.15 |  |
| 520 | *Lin28b* | CDNA clone IMAGE:30695895 | 5.44 |  |
| 521 | *Lin54* | Lin-54 homolog (C. elegans), mRNA (cDNA clone MGC:178581 IMAGE:9053573) | 2.73 |  |
| 522 | *Lin7a* | Lin-7 homolog A (C. elegans), mRNA (cDNA clone IMAGE:5356681) | 2.53 |  |
| 523 | *Lman2l* | Lectin, mannose-binding 2-like (Lman2l), mRNA | 2.12 |  |
| 524 | *Lmod3* | Leiomodin 3 (fetal) (Lmod3), mRNA | 6.26 |  |
| 525 | *LOC100039820* | PREDICTED: Mus musculus similar to ribosomal protein S28 (LOC100039820), mRNA | 10.39 |  |
| 526 | *LOC674419* | PREDICTED: Mus musculus similar to ribosomal protein L7a (LOC674419), mRNA | 2.5 |  |
| 527 | *Lonp2* | Lon peptidase 2, peroxisomal (Lonp2), mRNA | 2.13 |  |
| 528 | *Lonrf2* | LON peptidase N-terminal domain and ring finger 2, mRNA (cDNA clone MGC:170754 IMAGE:8862149) |  | 5.78 |
| 529 | *Lpar4* | Lysophosphatidic acid receptor 4, mRNA (cDNA clone IMAGE:4008657) | 2.01 |  |
| 530 | *Lrp4* | LDLR dan |  | 2.02 |
| 531 | *Lrpprc* | Leucine-rich PPR-motif containing (Lrpprc), mRNA | 2 |  |
| 532 | *Lrrc40* | Leucine rich repeat containing 40 (Lrrc40), mRNA | 2.33 |  |
| 533 | *Lrrc50* | Leucine rich repeat containing 50 (Lrrc50), mRNA |  | 2.03 |
| 534 | *Lrrc58* | Mus musculus, clone IMAGE:2647796, mRNA | 2.36 |  |
| 535 | *Lrrn1* | Leucine rich repeat protein 1, neuronal, mRNA (cDNA clone MGC:36161 IMAGE:4972803) | 7.42 |  |
| 536 | *Ltbp1* | Latent transforming growth factor beta binding protein 1 (Ltbp1), transcript variant 1, mRNA | 3.75 |  |
| 537 | *Lysmd1* | LysM, putative peptidoglycan-binding, domain containing 1 (Lysmd1), transcript variant 2, mRNA |  | 2.53 |
| 538 | *Mad1l1* | Mitotic arrest deficient 1-like 1, mRNA (cDNA clone MGC:11400 IMAGE:3156639) | 2 |  |
| 539 | *Macrod2* | MACRO domain containing 2, mRNA (cDNA clone IMAGE:1429747) | 2.27 |  |
| 540 | *Mad2l2* | MAD2 mitotic arrest deficient-like 2 (yeast) (Mad2l2), mRNA |  | 2.83 |
| 541 | *Man1c1* | Mannosidase, alpha, class 1C, member 1, mRNA (cDNA clone IMAGE:5373083) |  | 2.28 |
| 542 | *Man2b2* | Mannosidase 2, alpha B2 (Man2b2), mRNA |  | 2.04 |
| 543 | *Manea* | Mannosidase, endo-alpha, mRNA (cDNA clone MGC:91328 IMAGE:30628555) | 3.98 |  |
| 544 | *Maob* | Monoamine oxidase B, mRNA (cDNA clone MGC:132955 IMAGE:40061991) |  | 2.23 |
| 545 | *Mapk1ip1* | Mitogen-activated protein kinase 1 interacting protein 1 (Mapk1ip1), transcript variant 2, mRNA |  | 2.11 |
| 546 | *Matk* | Megakaryocyte-associated tyrosine kinase (Matk), mRNA |  | 6.9 |
| 547 | *Mboat2* | Membrane bound O-acyltransferase domain containing 2 (Mboat2), transcript variant 1, mRNA |  | 2.82 |
| 548 | *Mcam* | Melanoma cell adhesion molecule, mRNA (cDNA clone MGC:35823 IMAGE:5372497) | 2.16 |  |
| 549 | *Mcm2* | Minichromosome maintenance deficient 2 mitotin (S. cerevisiae) (Mcm2), mRNA | 2.84 |  |
| 550 | *Mcm3* | Minichromosome maintenance deficient 3 (S. cerevisiae), mRNA (cDNA clone MGC:30531 IMAGE:5007886) | 2.07 |  |
| 551 | *Mcm8* | Minichromosome maintenance deficient 8 (S. cerevisiae) (Mcm8), mRNA | 3.72 |  |
| 552 | *Med23* | Mediator complex subunit 23 (Med23), mRNA | 3.06 |  |
| 553 | *Med4* | Mediator of RNA polymerase II transcription, subunit 4 homolog (yeast), mRNA (cDNA clone MGC:28939 IMAGE:3992287) | 2.14 |  |
| 554 | *Mef2c* | Myocyte enhancer factor 2C, mRNA (cDNA clone MGC:46981 IMAGE:4500786) | 2.88 |  |
| 555 | *Megf10* | Multiple EGF-like-domains 10 (Megf10), mRNA | 3.35 |  |
| 556 | *Meis1* | Meis homeobox 1, mRNA (cDNA clone MGC:36059 IMAGE:5344307) | 2.05 |  |
| 557 | *Mesp2* | Mesoderm posterior 2 (Mesp2), mRNA |  | 3.15 |
| 558 | *Mest* | Mesoderm-specific transcript isoform 2 (Mest) | 23.35 |  |
| 559 | *Metrn* | Meteorin, glial cell differentiation regulator, mRNA (cDNA clone IMAGE:4222131) | 4.1 |  |
| 560 | *Mid1* | Midline 1 (Mid1), transcript variant 1, mRNA | 2.7 |  |
| 561 | *Mlf1* | Myeloid leukemia factor 1, mRNA (cDNA clone MGC:41163 IMAGE:1495721) | 2.76 |  |
| 562 | *Mllt3* | Myeloid/lymphoid or mixed-lineage leukemia (trithorax homolog, Drosophila); translocated to, 3 (Mllt3), transcript variant 1, | 2.14 |  |
| 563 | *Mmd2* | Monocyte to macrophage differentiation-associated 2, mRNA (cDNA clone MGC:36872 IMAGE:4502289) | 5.51 |  |
| 564 | *Mmel1* | Neprilysin-like peptidase gamma |  | 3.57 |
| 565 | *Mmp15* | Matrix metallopeptidase 15 (Mmp15), mRNA | 2.35 |  |
| 566 | *Mmp9* | Matrix metallopeptidase 9 (Mmp9), mRNA |  | 2.22 |
| 567 | *Mogat2* | Monoacylglycerol O-acyltransferase 2, mRNA (cDNA clone MGC:60738 IMAGE:30041797) | 9.04 |  |
| 568 | *Mpdz* | WSR2 multiple PDZ domain protein (Mpdz) | 3.33 |  |
| 569 | *Mpzl1* | Myelin protein zero-like 1 (Mpzl1), transcript variant 1, mRNA | 4.42 |  |
| 570 | *Mrgprb2* | MAS-related GPR, member B2, mRNA (cDNA clone MGC:130391 IMAGE:40057816) |  | 6.19 |
| 571 | *Mrpl21* | Mitochondrial ribosomal protein L21, mRNA (cDNA clone MGC:41352 IMAGE:1282313) | 2.08 |  |
| 572 | *Mrps2* | Mitochondrial ribosomal protein S2 (Mrps2), nuclear gene encoding mitochondrial protein, mRNA | 2.08 |  |
| 573 | *Mrps24* | Mitochondrial ribosomal protein S24, mRNA (cDNA clone MGC:35674 IMAGE:4911699) | 2.12 |  |
| 574 | *Msc* | Musculin (Msc), mRNA | 4.96 |  |
| 575 | *Msi2* | Musashi homolog 2 (Drosophila) (Msi2), mRNA | 3.35 |  |
| 576 | *Mstn* | Myostatin (Mstn), mRNA | 6.03 |  |
| 577 | *Mt4* | Metallothionein 4 (Mt4), mRNA |  | 8.26 |
| 578 | *Mta2* | Metastasis associated protein MTA2 (MTA2) | 2.68 |  |
| 579 | *Mtap1s* | Microtubule-associated protein 1S, mRNA (cDNA clone MGC:183640 IMAGE:9087640) | 2.47 |  |
| 580 | *Mtg1* | Mitochondrial GTPase 1 homolog (S. cerevisiae), mRNA (cDNA clone MGC:28365 IMAGE:4019717) | 2.05 |  |
| 581 | *Mtmr12* | Myotubularin related protein 12, mRNA (cDNA clone MGC:62683 IMAGE:3661020) |  | 2.26 |
| 582 | *Mtr* | 5-methyltetrahydrofolate-homocysteine methyltransferase, mRNA (cDNA clone MGC:175570 IMAGE:40130986) | 2.42 |  |
| 583 | *Mtrr* | 5-methyltetrahydrofolate-homocysteine methyltransferase reductase, mRNA (cDNA clone MGC:37944 IMAGE:5132457) | 2.37 |  |
| 584 | *Mtss1l* | Metastasis suppressor 1-like (Mtss1l), mRNA | 2.59 |  |
| 585 | *Mum1l1* | Melanoma associated antigen (mutated) 1-like 1, mRNA (cDNA clone MGC:169289 IMAGE:8860684) | 4.15 |  |
| 586 | *Mybpc1* | RIKEN cDNA 8030451F13 gene, mRNA (cDNA clone MGC:70062 IMAGE:30134842) | 6.68 |  |
| 587 | *Mybpc3* | Myosin binding protein C, cardiac (Mybpc3), mRNA | 4.62 |  |
| 588 | *Myf5* | Myogenic factor 5 (Myf5), mRNA | 2.31 |  |
| 589 | *Myh10* | Myosin, heavy polypeptide 10, non-muscle, mRNA (cDNA clone IMAGE:30100060) | 3.24 |  |
| 590 | *Myh14* | Nonmuscle myosin II-C2 (Myh14) |  | 2.7 |
| 591 | *Myl3* | Ventricular alkali myosin light chain | 11.88 |  |
| 592 | *Myl4* | Myosin, light polypeptide 4 (Myl4), mRNA | 11.51 | 3.27 |
| 593 | *Mylpf* | Myosin light chain, phosphorylatable, fast skeletal muscle (Mylpf), mRNA | 4.19 |  |
| 594 | *Myo18b* | Myosin XVIIIb, mRNA (cDNA clone IMAGE:9053769) | 3.02 |  |
| 595 | *Myo1b* | Myosin IB (Myo1b), mRNA | 3.43 |  |
| 596 | *Myod1* | Myogenic differentiation 1 (Myod1), mRNA | 17.76 |  |
| 597 | *Myog* | Myogenin (Myog), mRNA | 15.45 |  |
| 598 | *Myom1* | Myomesin 1 (Myom1), transcript variant 1, mRNA | 4.29 |  |
| 599 | *Myoz2* | Myozenin 2, mRNA (cDNA clone MGC:35862 IMAGE:5012436) | 6.66 |  |
| 600 | *Ncapg2* | Non-SMC condensin II complex, subunit G2 (Ncapg2), mRNA | 2.09 |  |
| 601 | *Ncoa6* | Nuclear receptor coactivator 6, mRNA (cDNA clone IMAGE:4976596) | 2.12 |  |
| 602 | *Ndn* | Necdin (Ndn), mRNA | 2.78 |  |
| 603 | *Ndufa9* | NADH dehydrogenase (ubiquinone) 1 alpha subcomplex, 9, mRNA (cDNA clone IMAGE:3601317) | 2.25 |  |
| 604 | *Ndufaf1* | NADH dehydrogenase (ubiquinone) 1 alpha subcomplex, assembly factor 1 (Ndufaf1), mRNA | 2.41 |  |
| 605 | *Ndufv2* | NADH dehydrogenase (ubiquinone) flavoprotein 2, mRNA (cDNA clone MGC:32165 IMAGE:5003524) | 2.2 |  |
| 606 | *Neb* | Nebulin (Neb), mRNA | 2.19 |  |
| 607 | *Nedd4* | Neural precursor cell expressed, developmentally down-regulated 4 (Nedd4), mRNA | 3.68 |  |
| 608 | *Neo1* | Neogenin (Neo1), transcript variant 1, mRNA | 2.11 |  |
| 609 | *Nes* | Nestin, mRNA (cDNA clone IMAGE:4206131) | 8.69 |  |
| 610 | *Neurod6* | Neurogenic differentiation 6 (Neurod6), mRNA | 3.74 |  |
| 611 | *Nexn* | Nexilin (Nexn), mRNA | 2.29 |  |
| 612 | *Ngef* | Neuronal guanine nucleotide exchange factor, mRNA (cDNA clone MGC:37278 IMAGE:4973826) |  | 3.56 |
| 613 | *Ngfr* | Nerve growth factor receptor (TNFR superfamily, member 16), mRNA (cDNA clone MGC:35588 IMAGE:5367638) | 3.19 | 2.02 |
| 614 | *Nhsl1* | NHS-like 1, mRNA (cDNA clone IMAGE:4035973) |  | 2.12 |
| 615 | *Nicn1* | Nicolin 1, mRNA (cDNA clone MGC:35757 IMAGE:5133763) | 2.5 |  |
| 616 | *Nkd1* | Naked cuticle 1 homolog (Drosophila), mRNA (cDNA clone MGC:40995 IMAGE:1429943) | 2.31 |  |
| 617 | *Nkd2* | Naked cuticle 2 homolog (Drosophila), mRNA (cDNA clone MGC:28383 IMAGE:4021772) |  | 3.92 |
| 618 | *Nnt* | Nicotinamide nucleotide transhydrogenase, mRNA (cDNA clone MGC:6038 IMAGE:3601702) | 2.77 |  |
| 619 | *Notch2* | (Notch2) |  | 2.36 |
| 620 | *Notch4* | Notch gene homolog 4 (Drosophila) (Notch4), mRNA | 2.48 |  |
| 621 | *Npepl1* | Aminopeptidase-like 1, mRNA (cDNA clone IMAGE:4207154) | 2.93 |  |
| 622 | *Npr2* | Natriuretic peptide receptor 2, mRNA (cDNA clone IMAGE:4159633) | 2.84 |  |
| 623 | *Npr3* | Natriuretic peptide receptor 3 (Npr3), transcript variant 1, mRNA | 2.89 |  |
| 624 | *Nptx1* | Neuronal pentraxin 1 (Nptx1), mRNA |  | 10.8 |
| 625 | *Nr2f2* | Nuclear receptor subfamily 2, group F, member 2 (Nr2f2), transcript variant 1, mRNA | 2.41 |  |
| 626 | *Nrm* | Nurim (nuclear envelope membrane protein), mRNA (cDNA clone MGC:36566 IMAGE:4984796) | 2.68 |  |
| 627 | *Nsmaf* | Neutral sphingomyelinase (N-SMase) activation associated factor (Nsmaf), mRNA |  | 2.48 |
| 628 | *Nt5dc2* | 5-nucleotidase domain containing 2, mRNA (cDNA clone MGC:19108 IMAGE:4207917) | 6.11 |  |
| 629 | *Nt5e* | 5 nucleotidase, ecto (Nt5e), mRNA |  | 2.7 |
| 630 | *Nudt4* | Nudix (nucleoside diphosphate linked moiety X)-type motif 4, mRNA (cDNA clone MGC:25580 IMAGE:3994391) | 2.25 |  |
| 631 | *Nup85* | Frount protein | 2.66 |  |
| 632 | *Nxn* | Nucleoredoxin, mRNA (cDNA clone MGC:7861 IMAGE:3501314) | 2.03 |  |
| 633 | *Obsl1* | Obscurin-like 1 (Obsl1), mRNA | 4.43 |  |
| 634 | *Ogfrl1* | Opioid growth factor receptor-like 1, mRNA (cDNA clone IMAGE:4485439) |  | 2.11 |
| 635 | *Olfm2* | Olfactomedin 2, mRNA (cDNA clone MGC:144796 IMAGE:40106193) |  | 2.96 |
| 636 | *Olfr1463* | Olfactory receptor 1463 (Olfr1463), mRNA |  | 2.53 |
| 637 | *Osgin2* | Oxidative stress induced growth inhibitor family member 2 (Osgin2), mRNA | 3.17 |  |
| 638 | *OTTMUSG00000015280* | PREDICTED: Mus musculus similar to keratinocyate lipid-binding protein (LOC677242), misc RNA | 2.44 |  |
| 639 | *Otud6b* | OTU domain containing 6B (Otud6b), mRNA | 2.52 |  |
| 640 | *Ovol2* | Ovo-like 2 (Drosophila) (Ovol2), transcript variant B, mRNA |  | 2.04 |
| 641 | *P4ha1* | Procollagen-proline, 2-oxoglutarate 4-dioxygenase (proline 4-hydroxylase), alpha 1 polypeptide, mRNA (cDNA clone MGC:6332 IMA | 2.5 |  |
| 643 | *Paqr5* | Progestin and adipoQ receptor family member V, mRNA (cDNA clone MGC:118370 IMAGE:30919009) |  | 2.7 |
| 644 | *Patl1* | Protein associated with topoisomerase II homolog 1 (yeast), mRNA (cDNA clone MGC:66563 IMAGE:6825758) | 2.28 |  |
| 645 | *Pcdh12* | Protocadherin 12 (Pcdh12), mRNA | 2 |  |
| 646 | *Pcdh18* | Protocadherin 18 (Pcdh18), mRNA | 4.49 |  |
| 647 | *Pcdh7* | Protocadherin 7 (Pcdh7), transcript variant 2, mRNA |  | 2.32 |
| 648 | *Pcdhga12* | Protocadherin gamma subfamily A, 10, mRNA (cDNA clone MGC:40648 IMAGE:5400956) | 3.79 |  |
| 649 | *Pcif1* | PDX1 C-terminal inhibiting factor 1 (Pcif1), mRNA | 2.35 |  |
| 650 | *Pck2* | Phosphoenolpyruvate carboxykinase 2 (mitochondrial), mRNA (cDNA clone IMAGE:3962737) | 2.05 |  |
| 651 | *Pcm1* | Pericentriolar material gene 1 protein (Pcm1) and pericentriolar material gene 1 protein (Pcm1) mRNAs | 2.45 |  |
| 652 | *Pcnt* | Pericentrin (kendrin) (Pcnt), transcript variant 1, mRNA | 2.19 |  |
| 653 | *Pdgfc* | Platelet-derived growth factor, C polypeptide, mRNA (cDNA clone MGC:46836 IMAGE:4008749) | 3.71 |  |
| 654 | *Pdlim3* | PDZ and LIM domain 3 (Pdlim3), mRNA |  | 4.96 |
| 655 | *Peg10* | Paternally expressed 10 (Peg10), transcript variant 1, mRNA | 11.07 |  |
| 656 | *Peg12* | FRAT3 (Frat3) | 4.48 |  |
| 657 | *Peg3* | Peg3 mRNA for zinc finger protein | 4.69 |  |
| 658 | *Peli2* | Pellino 2, mRNA (cDNA clone MGC:38603 IMAGE:5355240) |  | 2.73 |
| 659 | *Per2* | Period homolog 2 (Drosophila) (Per2), mRNA |  | 6.98 |
| 660 | *Pfkfb1* | 6-phosphofructo-2-kinase/fructose-2,6-bisphosphatase, clone 500bC6 | 2.96 |  |
| 661 | *Pfkm* | Phosphofructokinase, muscle, mRNA (cDNA clone MGC:7629 IMAGE:3495173) | 6.7 |  |
| 662 | *Pgm2* | Phosphoglucomutase 2 (Pgm2), mRNA | 2.1 |  |
| 663 | *Phc1* | Polyhomeotic-like 1 (Drosophila) (Phc1), transcript variant 1, mRNA | 3.05 |  |
| 664 | *Phf15* | PHD finger protein 15, mRNA (cDNA clone MGC:143877 IMAGE:40094330) |  | 2.06 |
| 665 | *Phyhipl* | Phytanoyl-CoA hydroxylase interacting protein-like, mRNA (cDNA clone MGC:46972 IMAGE:5400532) | 7.26 |  |
| 666 | *Pik3cd* | Phosphatidylinositol 3-kinase catalytic delta polypeptide, mRNA (cDNA clone MGC:28528 IMAGE:4192906) |  | 2.08 |
| 667 | *Pitx2* | Paired-like homeodomain transcription factor Munc 30 | 10.67 |  |
| 668 | *Pja2* | Praja 2, RING-H2 motif containing (Pja2), transcript variant 2, mRNA | 2.22 |  |
| 669 | *Pkia* | Protein kinase inhibitor, alpha (Pkia), mRNA | 2.35 |  |
| 670 | *Plagl1* | Lost on transformation protein 1 (Lot1) | 23.44 |  |
| 671 | *Plekha4* | Pleckstrin homology domain containing, family A (phosphoinositide binding specific) member 4 (Plekha4), mRNA |  | 2.21 |
| 672 | *Plod3* | Lysyl hydroxylase 3 | 2.23 |  |
| 673 | *Plxna3* | Plexin A3 (Plxna3), mRNA | 2.26 |  |
| 674 | *Pnkd* | Paroxysmal nonkinesiogenic dyskinesia (Pnkd), transcript variant 2, mRNA | 2.02 | 3.23 |
| 675 | *Pogz* | Pogo transposable element with ZNF domain, mRNA (cDNA clone IMAGE:5322259) | 2.02 |  |
| 676 | *Pold3* | Unknown mRNA | 3.45 |  |
| 677 | *Pom121* | Nuclear pore membrane protein 121 (Pom121), mRNA | 2.47 |  |
| 678 | *Popdc2* | Popeye domain containing 2 (Popdc2), transcript variant 2, mRNA | 2.42 |  |
| 679 | *Ppapdc3* | Phosphatidic acid phosphatase type 2 domain containing 3 (Ppapdc3), mRNA | 6.36 |  |
| 680 | *Ppil6* | Peptidylprolyl isomerase (cyclophilin)-like 6, mRNA (cDNA clone MGC:182811 IMAGE:9056705) |  | 2.06 |
| 681 | *Ppm1f* | Protein phosphatase 1F (PP2C domain containing) (Ppm1f), mRNA | 2.74 |  |
| 682 | *Ppp1r3c* | Protein phosphatase 1 binding protein PTG | 2.44 |  |
| 683 | *Ppp1r3f* | Protein phosphatase 1, regulatory (inhibitor) subunit 3F (Ppp1r3f), mRNA | 2.18 |  |
| 684 | *Ppp1r9a* | Protein phosphatase 1, regulatory (inhibitor) subunit 9A, mRNA (cDNA clone IMAGE:3481819) | 4.23 |  |
| 685 | *Prdm2* | PR domain containing 2, with ZNF domain, mRNA (cDNA clone MGC:183975 IMAGE:9087975) | 2.04 |  |
| 686 | *Prickle1* | Prickle like 1 (Drosophila), mRNA (cDNA clone IMAGE:4215628) | 2.44 | 2.47 |
| 687 | *Prkaca* | Protein kinase, cAMP dependent, catalytic, alpha, mRNA (cDNA clone MGC:6169 IMAGE:3497908) | 2.6 |  |
| 688 | *Prkcbp1* | Protein kinase C binding protein 1 (Prkcbp1), mRNA | 2.14 |  |
| 689 | *Prmt7* | Protein arginine N-methyltransferase 7 (Prmt7), mRNA | 3.1 |  |
| 690 | *Prrg3* | Proline rich Gla (G-carboxyglutamic acid) 3 (transmembrane), mRNA (cDNA clone MGC:169243 IMAGE:8860638) | 2.43 |  |
| 691 | *Ptbp1* | Polypyrimidine tract binding protein 1, mRNA (cDNA clone MGC:25792 IMAGE:4022742) | 2.43 |  |
| 692 | *Pter* | Phosphotriesterase related, mRNA (cDNA clone MGC:6034 IMAGE:3601675) | 2.78 |  |
| 693 | *Ptgs1* | Prostaglandin-endoperoxide synthase 1, mRNA (cDNA clone MGC:28109 IMAGE:3968136) |  | 2.04 |
| 694 | *Ptk7* | PTK7 protein tyrosine kinase 7, mRNA (cDNA clone IMAGE:5151185) | 2.58 |  |
| 695 | *Ptp4a3* | Protein tyrosine phosphatase 4a3, mRNA (cDNA clone MGC:36146 IMAGE:4482106) | 2.78 |  |
| 696 | *Ptpla* | Protein tyrosine phosphatase-like (proline instead of catalytic arginine), member a (Ptpla), transcript variant 1, mRNA | 3.02 |  |
| 697 | *Ptpn13* | Protein tyrosine phosphatase, non-receptor type 13 (Ptpn13), mRNA |  | 2.22 |
| 698 | *Ptpn14* | PTP36-D isoform |  | 2.01 |
| 699 | *Ptpn21* | Protein tyrosine phosphatase, non-receptor type 21 (Ptpn21), mRNA |  | 3.45 |
| 700 | *Ptprd* | Protein tyrosine phosphatase, receptor type, D, mRNA (cDNA clone IMAGE:4223353) | 3.85 |  |
| 701 | *Ptprs* | Protein tyrosine phosphatase | 2.09 |  |
| 702 | *Ptprz1* | Protein tyrosine phosphatase, receptor type Z, polypeptide 1, mRNA (cDNA clone IMAGE:3590815) | 3.34 | 2.76 |
| 703 | *Pum1* | Pumilio 1 (Drosophila) (Pum1), mRNA | 2.06 |  |
| 704 | *Pus3* | Pseudouridine synthase 3, mRNA (cDNA clone MGC:35810 IMAGE:4187198) | 2.25 |  |
| 705 | *Pvrl3* | Poliovirus receptor-related 3 (Pvrl3), transcript variant gamma, mRNA | 2.34 | 4.32 |
| 706 | *Qrsl1* | Glutaminyl-tRNA synthase (glutamine-hydrolyzing)-like 1, mRNA (cDNA clone IMAGE:4923133) | 2.71 |  |
| 707 | *Qsox2* | Quiescin Q6 sulfhydryl oxidase 2 (Qsox2), mRNA |  | 2.02 |
| 708 | *R3hdm1* | R3H domain 1 (binds single-stranded nucleic acids), mRNA (cDNA clone MGC:169396 IMAGE:8860791) | 2.62 |  |
| 709 | *Rab44* | RIKEN cDNA 9830134C10 gene, mRNA (cDNA clone MGC:170999 IMAGE:8862394) |  | 2.27 |
| 710 | *Rab4a* | RAB4A, member RAS oncogene family (Rab4a), mRNA | 4.52 |  |
| 711 | *Rab8b* | RAB8B, member RAS oncogene family, mRNA (cDNA clone MGC:189796 IMAGE:9007296) |  | 2.71 |
| 712 | *Rac3* | RAS-related C3 botulinum substrate 3, mRNA (cDNA clone IMAGE:5149761) | 2.18 |  |
| 713 | *Rai1* | Retinoic acid induced 1 (Rai1), transcript variant 1, mRNA | 2.68 |  |
| 714 | *Ranbp6* | RAN binding protein 6, mRNA (cDNA clone MGC:171022 IMAGE:8862417) | 3.93 |  |
| 715 | *Rasgrp3* | RasGRP3 | 3.31 |  |
| 716 | *Rasl12* | RAS-like, family 12, mRNA (cDNA clone MGC:144440 IMAGE:40102054) | 2.2 |  |
| 717 | *Rassf1* | Ras association (RalGDS/AF-6) domain family member 1, mRNA (cDNA clone MGC:7339 IMAGE:3486727) | 2.49 |  |
| 718 | *Raver2* | Ribonucleoprotein, PTB-binding 2 (Raver2), mRNA | 2.5 |  |
| 719 | *Rbm15* | RNA binding motif protein 15, mRNA (cDNA clone MGC:169365 IMAGE:8860760) | 2.45 |  |
| 720 | *Rbm24* | RNA binding motif protein 24 (Rbm24), mRNA | 11.16 |  |
| 721 | *Rbm38* | RNA binding motif protein 38 (Rbm38), mRNA | 3.25 |  |
| 722 | *Rbm5* | RNA binding motif protein 5 (Rbm5), mRNA | 2.41 |  |
| 723 | *Rbm9* | RNA binding motif protein 9, mRNA (cDNA clone MGC:28928 IMAGE:3661748) | 2.45 |  |
| 724 | *Rcn2* | Reticulocalbin 2 (Rcn2), mRNA | 2.47 |  |
| 725 | *Rcor2* | REST corepressor 2 (Rcor2), mRNA | 5.56 |  |
| 726 | *Reck* | Reversion-inducing-cysteine-rich protein with kazal motifs (Reck), mRNA | 2.64 |  |
| 727 | *Reep1* | Receptor expression enhancing protein 1 (Reep1) | 3.68 |  |
| 728 | *Rev3l* | REV3-like, catalytic subunit of DNA polymerase zeta RAD54 like (S. cerevisiae), mRNA (cDNA clone IMAGE:3967923) | 2.93 |  |
| 729 | *Rfx4* | Regulatory factor X, 4 (influences HLA class II expression), mRNA (cDNA clone MGC:170346 IMAGE:8861741) | 7.25 |  |
| 730 | *Rgmb* | RGM domain family, member B, mRNA (cDNA clone IMAGE:5351870) | 2.11 | 2.28 |
| 731 | *Rhobtb1* | Rho-related BTB domain containing 1, mRNA (cDNA clone MGC:178356 IMAGE:9053348) | 2 |  |
| 732 | *Rhobtb3* | MKIAA0878 protein | 4.43 |  |
| 733 | *Ripply3* | Ripply3 homolog (zebrafish) (Ripply3), mRNA | 2.96 |  |
| 734 | *Rlbp1l1* | Retinaldehyde binding protein 1-like 1, mRNA (cDNA clone IMAGE:30626969) | 2.82 |  |
| 735 | *Rnf2* | Ring finger protein 2, mRNA (cDNA clone MGC:28374 IMAGE:4021046) | 2.19 |  |
| 736 | *Rnmtl1* | RNA methyltransferase like 1, mRNA (cDNA clone IMAGE:4238990) | 2.53 |  |
| 737 | *Robo1* | Dutt1 protein |  | 2.41 |
| 738 | *Robo4* | Roundabout homolog 4 (Drosophila) (Robo4), mRNA | 3.29 |  |
| 739 | *Romo1* | Reactive oxygen species modulator 1, mRNA (cDNA clone MGC:41030 IMAGE:1196408) | 3.35 |  |
| 740 | *Rorc* | RAR-related orphan receptor gamma, mRNA (cDNA clone MGC:25220 IMAGE:4489193) |  | 3.11 |
| 741 | *Rpa1* | Replication protein A1 (Rpa1), mRNA | 2.21 |  |
| 742 | *Rpap2* | RNA polymerase II associated protein 2 (Rpap2), mRNA | 2.21 |  |
| 743 | *Rpl15* | Ribosomal protein L15 (Rpl15), mRNA | 2.48 |  |
| 744 | *Rragd* | Ras-related GTP binding D, mRNA (cDNA clone IMAGE:1349177) | 6.35 |  |
| 745 | *Rrm1* | Ribonucleotide reductase M1, mRNA (cDNA clone MGC:25382 IMAGE:4485739) | 2.67 |  |
| 746 | *Rsad1* | Radical S-adenosyl methionine domain containing 1, mRNA (cDNA clone MGC:170901 IMAGE:8862296) | 4.52 |  |
| 747 | *Rspo3* | R-spondin 3 homolog (Xenopus laevis), mRNA (cDNA clone MGC:175818 IMAGE:40131234) | 3.85 | 8.34 |
| 748 | *Rtn2* | Reticulon 2 (Z-band associated protein), mRNA (cDNA clone MGC:35990 IMAGE:5359358) | 4.3 |  |
| 749 | *Rtn4* | RTN4 (Rtn4) mRNA, complete cds, alternatively spliced | 2.91 |  |
| 750 | *Rtn4r* | Reticulon 4 receptor (Rtn4r), mRNA |  | 3.68 |
| 751 | *S1pr1* | Sphingosine-1-phosphate receptor 1 (S1pr1), mRNA | 2.79 |  |
| 752 | *S1pr2* | Sphingosine-1-phosphate receptor 2, mRNA (cDNA clone MGC:107350 IMAGE:30042746) | 2.55 |  |
| 753 | *Samd14* | Sterile alpha motif domain containing 14 (Samd14), transcript variant 1, mRNA | 2.18 |  |
| 754 | *Saps3* | SAPS domain family, member 3 (Saps3), mRNA | 2.34 |  |
| 755 | *Satb1* | Special AT-rich sequence binding protein 1, mRNA (cDNA clone MGC:18461 IMAGE:4164993) | 5.79 | 3.59 |
| 756 | *Sbsn* | Suprabasin, mRNA (cDNA clone MGC:171012 IMAGE:8862407) |  | 2.2 |
| 757 | *Scaf1* | SR-related CTD-associated factor 1 (Scaf1), mRNA | 4.17 |  |
| 758 | *Scd2* | Fatty acid desaturase (Scd3) | 3.68 |  |
| 759 | *Schip1* | Schwannomin interacting protein 1 (Schip1), transcript variant 4, mRNA | 2.52 |  |
| 760 | *Scml2* | Sex comb on midleg-like 2 (Drosophila) (Scml2), mRNA |  | 2.27 |
| 761 | *Scn3b* | Sodium channel, voltage-gated, type III, beta (Scn3b), transcript variant 1, mRNA | 3.68 |  |
| 762 | *Scoc* | Short coiled-coil protein, mRNA (cDNA clone IMAGE:4506964) | 2.73 |  |
| 763 | *Scrib* | Scribbled homolog (Drosophila), mRNA (cDNA clone IMAGE:4459388) | 2.02 |  |
| 764 | *Sema3d* | Sema domain, immunoglobulin domain (Ig), short basic domain, secreted, (semaphorin) 3D, mRNA (cDNA clone MGC:169755 IMAGE:886 |  | 2.19 |
| 766 | *Sepn1* | Selenoprotein N, 1, mRNA (cDNA clone IMAGE:4036667) | 2.11 |  |
| 767 | *Serpina12* | Serine (or cysteine) peptidase inhibitor, clade A (alpha-1 antiproteinase, antitrypsin), member 12 (Serpina12), mRNA |  | 2.24 |
| 768 | *Serpina3h* | Serine (or cysteine) peptidase inhibitor, clade A, member 3H, mRNA (cDNA clone MGC:164278 IMAGE:40130924) |  | 2.35 |
| 769 | *Serpinb10* | Serine (or cysteine) peptidase inhibitor, clade B (ovalbumin), member 10, mRNA (cDNA clone MGC:25706 IMAGE:3676050) |  | 2 |
| 770 | *Serpinb11* | Serine (or cysteine) peptidase inhibitor, clade B (ovalbumin), member 11, mRNA (cDNA clone MGC:6485 IMAGE:2646766) |  | 3.68 |
| 771 | *Serpinb7* | Serine (or cysteine) peptidase inhibitor, clade B, member 7 (Serpinb7), mRNA |  | 2.72 |
| 772 | *Setd6* | SET domain containing 6, mRNA (cDNA clone MGC:170825 IMAGE:8862220) |  | 2.29 |
| 773 | *Sf1* | Splicing factor 1, mRNA (cDNA clone MGC:7095 IMAGE:3157495) | 2.89 |  |
| 774 | *Sf3b3* | Splicing factor 3b, subunit 3, mRNA (cDNA clone IMAGE:5252985) | 3.59 |  |
| 775 | *Sfrp2* | Secreted frizzled-related protein 2, mRNA (cDNA clone MGC:25299 IMAGE:4487469) | 2.38 |  |
| 776 | *Sfxn2* | Sideroflexin 2, mRNA (cDNA clone MGC:30418 IMAGE:5043343) | 2.26 |  |
| 777 | *Sh2b1* | PSM/SH2-B delta (Sh2bpsm1) | 2.14 |  |
| 778 | *Sh2d2a* | SH2 domain protein 2A, mRNA (cDNA clone MGC:41076 IMAGE:3327113) |  | 2.08 |
| 779 | *Sh3bgr* | Putative SH3BGR protein (SH3BGR gene) | 3.95 |  |
| 780 | *Sh3bp4* | SH3-domain binding protein 4, mRNA (cDNA clone IMAGE:3962517) | 4.03 |  |
| 781 | *Shoc2* | Soc-2 (suppressor of clear) homolog (C. elegans), mRNA (cDNA clone MGC:31051 IMAGE:4014650) | 3.62 |  |
| 782 | *Sik1* | Salt inducible kinase 1 (Sik1), mRNA |  | 2.71 |
| 783 | *Sin3a* | Transcriptional regulator, SIN3A (yeast) (Sin3a), transcript variant 1, mRNA | 2.03 |  |
| 784 | *Skint10* | Strain C57BL/6J skint 10 isoform a precursor (Skint10) |  | 2.38 |
| 785 | *Slc16a3* | Solute carrier family 16 (monocarboxylic acid transporters), member 3 (Slc16a3), mRNA | 4.02 |  |
| 786 | *Slc1a1* | Solute carrier family 1 (neuronal/epithelial high affinity glutamate transporter, system Xag), member 1 (Slc1a1), mRNA |  | 3.94 |
| 787 | *Slc1a3* | Solute carrier family 1 (glial high affinity glutamate transporter), member 3 (Slc1a3), mRNA | 2.17 |  |
| 788 | *Slc1a4* | Solute carrier family 1 (glutamate/neutral amino acid transporter), member 4 (Slc1a4), mRNA | 3.39 |  |
| 789 | *Slc22a4* | Solute carrier family 22 (organic cation transporter), member 4, mRNA (cDNA clone MGC:18328 IMAGE:3670138) |  | 2.81 |
| 790 | *Slc25a24* | Solute carrier family 25 (mitochondrial carrier, phosphate carrier), member 24, mRNA (cDNA clone IMAGE:4208509) | 2.04 |  |
| 791 | *Slc25a27* | Solute carrier family 25, member 27 (Slc25a27), nuclear gene encoding mitochondrial protein, mRNA |  | 2.76 |
| 792 | *Slc25a37* | Solute carrier family 25, member 37, mRNA (cDNA clone IMAGE:4456736) |  | 2.22 |
| 793 | *Slc25a38* | Solute carrier family 25, member 38 (Slc25a38), mRNA |  | 2.17 |
| 794 | *Slc25a4* | Solute carrier family 25 (mitochondrial carrier, adenine nucleotide translocator), member 4, mRNA (cDNA clone MGC:25083 IMAGE | 3.29 |  |
| 795 | *Slc26a11* | Solute carrier family 26, member 11, mRNA (cDNA clone IMAGE:4923075) |  | 2.12 |
| 796 | *Slc2a12* | Solute carrier family 2 (facilitated glucose transporter), member 12, mRNA (cDNA clone MGC:169942 IMAGE:8861337) |  | 3.73 |
| 797 | *Slc30a9* | Solute carrier family 30 (zinc transporter), member 9, mRNA (cDNA clone IMAGE:5345284) | 2.03 |  |
| 798 | *Slc35a2* | Solute carrier family 35 (UDP-galactose transporter), member A2, mRNA (cDNA clone MGC:46966 IMAGE:4187325) |  | 2.02 |
| 799 | *Slc37a1* | Solute carrier family 37 (glycerol-3-phosphate transporter), member 1 (Slc37a1), mRNA |  | 2.63 |
| 800 | *Slc38a10* | Solute carrier family 38, member 10, mRNA (cDNA clone IMAGE:5011722) | 2.12 |  |
| 801 | *Slc38a4* | Solute carrier family 38, member 4, mRNA (cDNA clone MGC:37778 IMAGE:5097293) | 7.53 |  |
| 802 | *Slc39a8* | Solute carrier family 39 (metal ion transporter), member 8, mRNA (cDNA clone MGC:7011 IMAGE:3155670) |  | 2.1 |
| 803 | *Slc4a1* | Solute carrier family 4 (anion exchanger), member 1 (Slc4a1), mRNA | 15.87 |  |
| 804 | *Slc5a7* | Solute carrier family 5 (choline transporter), member 7 (Slc5a7), mRNA | 2.38 |  |
| 805 | *Slc6a19* | Solute carrier family 6 (neurotransmitter transporter), member 19, mRNA (cDNA clone MGC:130422 IMAGE:40059042) |  | 2.54 |
| 806 | *Slc7a6* | Solute carrier family 7 (cationic amino acid transporter, y+ system), member 6, mRNA (cDNA clone MGC:37035 IMAGE:4949962) |  | 2.43 |
| 807 | *Slc9a9* | Solute carrier family 9 (sodium/hydrogen exchanger), member 9, mRNA (cDNA clone MGC:164721 IMAGE:40093280) |  | 2.48 |
| 808 | *Slco4a1* | Solute carrier organic anion transporter family, member 4a1 (Slco4a1), mRNA |  | 3.08 |
| 809 | *Sln* | Sarcolipin, mRNA (cDNA clone MGC:41044 IMAGE:1397989) | 11.01 |  |
| 810 | *Smad1* | MAD homolog 1 (Drosophila) (Smad1), mRNA | 2.56 |  |
| 811 | *Smarca1* | SWI/SNF related, matrix associated, actin dependent regulator of chromatin, subfamily a, member 1 (Smarca1), mRNA | 2.91 |  |
| 812 | *Smarcc1* | SWI/SNF related, matrix associated, actin dependent regulator of chromatin, subfamily c, member 1, mRNA (cDNA clone IMAGE:401 | 3.04 |  |
| 813 | *Smarcd3* | SWI/SNF related, matrix associated, actin dependent regulator of chromatin, subfamily d, member 3, mRNA (cDNA clone MGC:8018 | 2.73 |  |
| 814 | *Smoc2* | SPARC related modular calcium binding 2, mRNA (cDNA clone MGC:28521 IMAGE:4191849) |  | 3.67 |
| 815 | *Smpx* | SMPX protein (Smpx) | 8.91 |  |
| 816 | *Sms* | Spermine synthase (Sms), mRNA | 3.23 |  |
| 817 | *Smtnl2* | Smoothelin-like 2, mRNA (cDNA clone MGC:47155 IMAGE:5064159) | 8.4 |  |
| 818 | *Snca* | Alpha-synuclein mRNA, alternatively spliced | 4.49 |  |
| 819 | *Sod3* | Superoxide dismutase 3, extracellular, mRNA (cDNA clone MGC:13799 IMAGE:3983917) |  | 3.5 |
| 820 | *Sorbs1* | C-Cbl associated protein CAP |  | 3.71 |
| 821 | *Sorbs2* | Sorbin and SH3 domain containing 2, mRNA (cDNA clone IMAGE:4017890) | 2.98 |  |
| 822 | *Sos2* | Son of sevenless 2 | 2.21 |  |
| 823 | *Sox11* | SRY-box containing gene 11 (Sox11), mRNA | 11.56 |  |
| 824 | *Sox12* | SRY-box containing gene 12 (Sox12), mRNA | 4.38 |  |
| 825 | *Sox13* | SRY-box containing gene 13, mRNA (cDNA clone MGC:5792 IMAGE:3493465) | 2.42 |  |
| 826 | *Sox17* | SRY-box containing gene 17 (Sox17), mRNA | 2.41 |  |
| 827 | *Sox18* | SRY-box containing gene 18, mRNA (cDNA clone MGC:11476 IMAGE:3967084) | 5.73 |  |
| 828 | *Sox6* | SRY-box containing gene 6 (Sox6), transcript variant 1, mRNA | 2.71 |  |
| 829 | *Spata7* | SPATA7 isoform mRNA, complete cds, alternatively spliced | 3.19 |  |
| 830 | *Speg* | Striated muscle-specific serine/threonine protein kinase (Speg) | 3.27 |  |
| 831 | *Sphkap* | SPHK1 interactor, AKAP domain containing, mRNA (cDNA clone MGC:76468 IMAGE:30431760) | 3.02 |  |
| 832 | *Spnb1* | Spectrin beta 1 (Spnb1), mRNA | 2.61 |  |
| 833 | *Spock3* | Sparc/osteonectin, cwcv and kazal-like domains proteoglycan 3, mRNA (cDNA clone MGC:27597 IMAGE:4502169) | 2.94 |  |
| 834 | *Spry1* | Sprouty homolog 1 (Drosophila), mRNA (cDNA clone MGC:18307 IMAGE:3672353) |  | 4.93 |
| 835 | *Spryd4* | SPRY domain containing 4, mRNA (cDNA clone MGC:25683 IMAGE:4923277) | 2.13 |  |
| 836 | *Sptlc3* | Serine palmitoyltransferase, long chain base subunit 3, mRNA (cDNA clone MGC:106295 IMAGE:3483569) |  | 2.69 |
| 837 | *Sqrdl* | Sulfide quinone reductase-like (yeast), mRNA (cDNA clone MGC:18811 IMAGE:4196280) |  | 2.49 |
| 838 | *Srf* | Serum response factor (Srf), mRNA | 2.37 |  |
| 839 | *Srgap2* | SLIT-ROBO Rho GTPase activating protein 2, mRNA (cDNA clone MGC:183994 IMAGE:9087994) |  | 2.7 |
| 840 | *Srl* | Sarcalumenin, mRNA (cDNA clone MGC:175574 IMAGE:40130990) | 8.65 |  |
| 841 | *Sspn* | Sarcospan, mRNA (cDNA clone MGC:36063 IMAGE:5344514) | 2.22 |  |
| 842 | *Ssrp1* | Structure specific recognition protein 1, mRNA (cDNA clone IMAGE:5355658) | 2.18 |  |
| 843 | *St3gal4* | ST3 beta-galactoside alpha-2,3-sialyltransferase 4, mRNA (cDNA clone MGC:6096 IMAGE:3497996) |  | 3.14 |
| 844 | *St6galnac3* | ST6 (alpha-N-acetyl-neuraminyl-2,3-beta-galactosyl-1,3)-N-acetylgalactosaminide alpha-2,6-sialyltransferase 3 (St6galnac3), m | 4.78 |  |
| 845 | *St6galnac4* | ST6 (alpha-N-acetyl-neuraminyl-2,3-beta-galactosyl-1,3)-N-acetylgalactosaminide alpha-2,6-sialyltransferase 4 (St6galnac4), m | 2.77 |  |
| 846 | *St6galnac6* | ST6GalNAc VI mRNA for GD1 alpha/GT1a alpha/GQ1b alpha synthase | 2.58 |  |
| 847 | *St8sia2* | ST8 alpha-N-acetyl-neuraminide alpha-2,8-sialyltransferase 2 (St8sia2), mRNA | 3.08 |  |
| 848 | *Stk10* | Serine/threonine kinase 10 (Stk10), mRNA |  | 2.52 |
| 849 | *Strn4* | Striatin, calmodulin binding protein 4 (Strn4), transcript variant 1, mRNA | 3.57 |  |
| 850 | *Suhw4* | Suppressor of hairy wing homolog 4 (Drosophila) (Suhw4), mRNA | 2.3 |  |
| 851 | *Sumf2* | Sulfatase modifying factor 2, mRNA (cDNA clone MGC:141062 IMAGE:40050522) | 2.47 | 2.07 |
| 852 | *Susd2* | Sushi domain containing 2 (Susd2), mRNA |  | 2 |
| 853 | *Suv39h1* | Suppressor of variegation 3-9 homolog 1 (Drosophila), mRNA (cDNA clone MGC:36038 IMAGE:5352230) | 2.83 |  |
| 854 | *Syde1* | PREDICTED: Mus musculus synapse defective 1, Rho GTPase, homolog 1 (C. elegans) (Syde1), mRNA | 2.28 |  |
| 855 | *Syngr1* | Synaptogyrin 1 (Syngr1), transcript variant 1b, mRNA |  | 2.11 |
| 856 | *Tanc1* | Tetratricopeptide repeat, ankyrin repeat and coiled-coil containing 1, mRNA (cDNA clone MGC:176329 IMAGE:9055980) |  | 3.01 |
| 857 | *Tbpl1* | TATA box binding protein-like 1, mRNA (cDNA clone MGC:41136 IMAGE:1382180) |  | 2.82 |
| 858 | *Tbrg4* | Transforming growth factor beta regulated gene 4, mRNA (cDNA clone MGC:38943 IMAGE:5362943) | 2.61 |  |
| 859 | *Tbx15* | T-box 15 (Tbx15), mRNA | 2.27 |  |
| 860 | *Tbx3* | T-box 3, mRNA (cDNA clone MGC:106457 IMAGE:30547736) | 3.96 |  |
| 861 | *Tceal5* | Transcription elongation factor A (SII)-like 5, mRNA (cDNA clone MGC:144259 IMAGE:40100237) | 4.93 |  |
| 862 | *Tcf7l2* | HMG box transcription factor TCF7L2 (Tcf7l2) mRNA, complete cds, alternatively spliced | 2.05 |  |
| 863 | *Tcfap4* | Transcription factor AP4 (Tcfap4), mRNA | 2.28 |  |
| 864 | *Tcfcp2* | Transcription factor CP2, mRNA (cDNA clone MGC:37813 IMAGE:5098336) | 2.97 |  |
| 865 | *Tcta* | T-cell leukemia translocation altered gene, mRNA (cDNA clone MGC:25540 IMAGE:3672301) |  | 2.02 |
| 866 | *Tcte1* | T-complex-associated testis expressed 1 (Tcte1), mRNA | 6.28 |  |
| 867 | *Tdrd1* | Tdr1 mRNA for tudor repeat 1 protein | 4.23 |  |
| 868 | *Tdrd7* | Tudor domain containing 7 (Tdrd7), mRNA |  | 2.17 |
| 869 | *Tecpr2* | RIKEN cDNA 4930573I19 gene, mRNA (cDNA clone MGC:169979 IMAGE:8861374) |  | 2.01 |
| 870 | *Tesc* | Tescalcin, mRNA (cDNA clone MGC:28673 IMAGE:4237590) |  | 3.78 |
| 871 | *Tgfb2* | Transforming growth factor, beta 2, mRNA (cDNA clone MGC:7998 IMAGE:3585774) | 3.09 |  |
| 872 | *Th1l* | TH1-like homolog (Drosophila), mRNA (cDNA clone MGC:18723 IMAGE:3673038) | 2.6 |  |
| 873 | *Thsd1* | Thrombospondin, type I, domain 1 (Thsd1), mRNA |  | 9.14 |
| 874 | *Thyn1* | Thymocyte protein mThy28 | 2.33 |  |
| 875 | *Timeless* | Timeless homolog (Drosophila), mRNA (cDNA clone IMAGE:4924460) | 2 |  |
| 876 | *Tmc7* | CDNA clone IMAGE:30617269 | 3.8 |  |
| 877 | *Tmem38a* | Transmembrane protein 38A (Tmem38a), mRNA | 3.43 |  |
| 878 | *Tmem54* | Transmembrane protein 54, mRNA (cDNA clone MGC:28617 IMAGE:4219997) |  | 2.11 |
| 879 | *Tmem88* | Transmembrane protein 88 (Tmem88), mRNA | 3.27 |  |
| 880 | *Tmtc2* | Transmembrane and tetratricopeptide repeat containing 2, mRNA (cDNA clone IMAGE:5012310) | 2.74 |  |
| 881 | *Tmtc4* | Transmembrane and tetratricopeptide repeat containing 4, mRNA (cDNA clone MGC:32342 IMAGE:5029461) |  | 2.21 |
| 882 | *Tnfrsf25* | Tumor necrosis factor receptor superfamily, member 25, mRNA (cDNA clone MGC:27681 IMAGE:4912933) |  | 2.35 |
| 883 | *Tnik* | TRAF2 and NCK interacting kinase, mRNA (cDNA clone MGC:183916 IMAGE:9087916) |  | 2.66 |
| 884 | *Tnnc1* | Troponin C, cardiac/slow skeletal (Tnnc1), mRNA | 10.08 |  |
| 885 | *Tnnt1* | Troponin T1, skeletal, slow (Tnnt1), mRNA | 5.34 |  |
| 886 | *Tom1l1* | Adaptor molecule SRCASM (Srcasm) | 2.06 |  |
| 887 | *Tpm2* | Tropomyosin 2, beta, mRNA (cDNA clone MGC:35840 IMAGE:4990394) | 3.81 |  |
| 888 | *Tram1l1* | Translocation associated membrane protein 1-like 1 (Tram1l1), mRNA | 2.23 |  |
| 889 | *Trat1* | T cell receptor interacting molecule (Trim gene) |  | 2.77 |
| 890 | *Trfr2* | Transferrin receptor 2, mRNA (cDNA clone MGC:18814 IMAGE:4196597) |  | 2.69 |
| 891 | *Trib2* | Tribbles homolog 2 (Drosophila), mRNA (cDNA clone MGC:32449 IMAGE:5043179) | 3.11 |  |
| 892 | *Trim35* | Tripartite motif-containing 35 (Trim35), mRNA | 2.8 |  |
| 893 | *Tsc22d1* | TSC22-related inducible leucine zipper 1b (Tilz1b) | 2.01 |  |
| 894 | *Tsc22d3* | TSC22 domain family, member 3, mRNA (cDNA clone MGC:36071 IMAGE:5136440) |  | 2.48 |
| 895 | *Tsix* | Domesticus antisense RNA from the Xist locus, complete sequence |  | 2.08 |
| 896 | *Tspan18* | PREDICTED: Mus musculus hypothetical protein LOC100042789 (LOC100042789), mRNA | 2.89 |  |
| 897 | *Tspan32* | AML-1 regulated transmembrane protein Art-1 |  | 3.07 |
| 898 | *Tspan8* | Tetraspanin 8 (Tspan8), mRNA |  | 2.58 |
| 899 | *Ttc28* | Tetratricopeptide repeat domain 28, mRNA (cDNA clone MGC:7623 IMAGE:3495045) | 2.57 |  |
| 900 | *Ttc9* | Tetratricopeptide repeat domain 9, mRNA (cDNA clone IMAGE:3966257) |  | 3.06 |
| 901 | *Ttrap* | TRAF and TNF receptor associated protein (Ttrap), mRNA | 3.7 |  |
| 902 | *Tub* | Tubby candidate gene, mRNA (cDNA clone IMAGE:4527484) | 2.95 |  |
| 903 | *Tubgcp5* | Tubulin, gamma complex associated protein 5 (Tubgcp5), mRNA | 2.05 |  |
| 904 | *Uaca* | Uveal autoantigen with coiled-coil domains and ankyrin repeats, mRNA (cDNA clone MGC:37093 IMAGE:4951775) | 2.16 |  |
| 905 | *Ube2o* | Ubiquitinating enzyme E2-230 kDa | 2.29 |  |
| 906 | *Ubqln2* | Ubiquilin 2, mRNA (cDNA clone IMAGE:4009660) | 2.55 |  |
| 907 | *Ubr4* | PREDICTED: Mus musculus zinc finger, UBR1 type 1, transcript variant 1 (Zubr1), mRNA | 2.03 |  |
| 908 | *Ugt8a* | UDP galactosyltransferase 8A, mRNA (cDNA clone MGC:18397 IMAGE:4223057) | 4.04 |  |
| 909 | *Uhrf2* | Ubiquitin-like, containing PHD and RING finger domains 2 (Uhrf2), mRNA | 2.1 |  |
| 910 | *Unc45b* | Unc-45 homolog B (C. elegans), mRNA (cDNA clone MGC:91090 IMAGE:30429260) | 2.03 |  |
| 911 | *Urb1* | MKIAA0539 protein | 2.25 |  |
| 912 | *Usp11* | Ubiquitin specific peptidase 11 (Usp11), mRNA | 2.82 |  |
| 913 | *Usp12* | Deubiquitinating enzyme UBH1 | 2.24 |  |
| 914 | *Usp18* | Ubiquitin specific protease UBP43 |  | 2.18 |
| 915 | *Usp27x* | Ubiquitin specific protease (Uspx) | 2.16 |  |
| 916 | *Usp28* | Ubiquitin specific peptidase 28, mRNA (cDNA clone MGC:91169 IMAGE:30459522) | 2.38 |  |
| 917 | *Usp40* | Ubiquitin specific peptidase 40, mRNA (cDNA clone MGC:144143 IMAGE:40098174) | 2.05 |  |
| 918 | *Vars* | Valyl-tRNA synthetase (G7a) | 2.27 |  |
| 919 | *Vcam1* | Vascular cell adhesion molecule 1, mRNA (cDNA clone MGC:36028 IMAGE:4196681) | 4.38 |  |
| 920 | *Vegfb* | Vascular endothelial growth factor B (Vegfb), mRNA | 2.02 |  |
| 921 | *Vgll2* | Vestigial like 2 homolog (Drosophila) (Vgll2), mRNA | 8.9 |  |
| 922 | *Vwa2* | Von Willebrand factor A domain containing 2, mRNA (cDNA clone MGC:141475 IMAGE:40081354) | 2.03 | 2.06 |
| 923 | *Wdr47* | WD repeat domain 47 (Wdr47), mRNA |  | 2.04 |
| 924 | *Wdr86* | WD repeat domain 86 (Wdr86), mRNA | 6.65 |  |
| 925 | *Wfdc3* | WAP four-disulfide core domain 3 (Wfdc3), mRNA |  | 17.66 |
| 926 | *Wnt7a* | Wingless-related MMTV integration site 7A (Wnt7a), mRNA | 5.63 |  |
| 927 | *Wrnip1* | Werner helicase interacting protein 1 (Wrnip1), mRNA | 2.08 |  |
| 928 | *Wscd1* | MKIAA0523 protein | 7.38 |  |
| 929 | *Wwc2* | WW, C2 and coiled-coil domain containing 2 (Wwc2), mRNA | 2.21 |  |
| 930 | *Xab2* | XPA binding protein 2, mRNA (cDNA clone MGC:29394 IMAGE:5066146) | 2.2 |  |
| 931 | *Xk* | Kell blood group precursor (McLeod phenotype) homolog (Xk), mRNA | 2.38 |  |
| 932 | *Zadh2* | Zinc binding alcohol dehydrogenase, domain containing 2 (Zadh2), mRNA | 2.11 |  |
| 933 | *Zbtb10* | PREDICTED: Mus musculus zinc finger and BTB domain containing 10, transcript variant 1 (Zbtb10), mRNA | 2.51 |  |
| 934 | *Zbtb33* | Zinc finger and BTB domain containing 33 (Zbtb33), transcript variant 1, mRNA | 3.13 |  |
| 935 | *Zc3h4* | Zinc finger CCCH-type containing 4 (Zc3h4), mRNA | 2.02 |  |
| 936 | *Zcchc14* | M-BDG29 | 2.37 |  |
| 937 | *Zcchc3* | Zinc finger, CCHC domain containing 3, mRNA (cDNA clone MGC:67159 IMAGE:6412435) | 7.42 |  |
| 938 | *Zdbf2* | PREDICTED: Mus musculus RIKEN cDNA 4930431J08 gene (4930431J08Rik), mRNA | 10.54 |  |
| 939 | *Zdhhc15* | Zinc finger, DHHC domain containing 15 (Zdhhc15), mRNA | 2.23 |  |
| 940 | *Zfhx4* | Zinc finger homeodomain 4 (Zfhx4), mRNA | 4.19 |  |
| 941 | *Zfp106* | Zinc finger protein 106, mRNA (cDNA clone MGC:29071 IMAGE:4459713) | 2.02 |  |
| 942 | *Zfp2* | Strain ILS multifinger protein mKr2 | 2.15 |  |
| 943 | *Zfp239* | Zinc finger protein 239, mRNA (cDNA clone MGC:7007 IMAGE:3155528) | 2.2 |  |
| 944 | *Zfp260* | Zinc finger protein 260, mRNA (cDNA clone IMAGE:5025492) | 2.04 |  |
| 945 | *Zfp275* | Zinc finger protein 275, mRNA (cDNA clone IMAGE:3991691) | 3.87 |  |
| 946 | *Zfp280b* | Zinc finger protein 280B, mRNA (cDNA clone IMAGE:40095592) | 2.09 |  |
| 947 | *Zfp282* | Zinc finger protein 282 (Zfp282), mRNA | 2 |  |
| 948 | *Zfp318* | Zinc finger protein TZF-L | 2.54 |  |
| 949 | *Zfp322a* | Zinc finger protein 322A, mRNA (cDNA clone IMAGE:5040278) | 2.32 |  |
| 950 | *Zfp334* | Zinc finger protein 334, mRNA (cDNA clone IMAGE:5355142) | 3.54 |  |
| 951 | *Zfp423* | Early B-cell factor-associated zinc finger protein (Ebfaz) | 2.21 |  |
| 952 | *Zfp428* | Zinc finger protein 428 (Zfp428), mRNA | 5.52 |  |
| 953 | *Zfp449* | Zinc finger protein 449, mRNA (cDNA clone MGC:175823 IMAGE:40131239) | 2.05 |  |
| 954 | *Zfp462* | Zinc finger protein 462 (Zfp462) | 2.99 |  |
| 955 | *Zfp503* | Zinc finger protein 503 (Zfp503), mRNA | 2.19 |  |
| 956 | *Zfp518b* | RIKEN cDNA 6820424L24 gene, mRNA (cDNA clone IMAGE:4024509) | 3.83 |  |
| 957 | *Zfp521* | Ecotropic viral integration site 3 (Evi3) | 4.37 |  |
| 958 | *Zfp523* | PREDICTED: Mus musculus similar to collagen, type VIII, alpha 2 (LOC100042764), mRNA | 2.07 |  |
| 959 | *Zfp566* | Zinc finger protein 566 (Zfp566), mRNA | 2.53 |  |
| 960 | *Zfp606* | Zinc finger protein 606 (Zfp606), transcript variant 2, mRNA | 2.3 |  |
| 961 | *Zfp608* | Zinc finger protein 608, mRNA (cDNA clone MGC:183986 IMAGE:9087986) | 2.25 |  |
| 962 | *Zfp618* | Neural precursor cell expressed, developmentally down-regulated gene 10, mRNA (cDNA clone IMAGE:6827030) | 5.01 |  |
| 963 | *Zfp622* | Zinc finger protein 622 (Zfp622), mRNA | 2.62 |  |
| 964 | *Zfp652* | MKIAA0924 protein |  | 2.29 |
| 965 | *Zfp653* | Zinc finger protein 653, mRNA (cDNA clone MGC:47124 IMAGE:4190337) |  | 2.03 |
| 966 | *Zfp689* | Zinc finger protein 689, mRNA (cDNA clone IMAGE:1329526) | 2.06 |  |
| 967 | *Zfp78* | Zinc finger protein 78 (Zfp78), transcript variant 3, mRNA |  | 2.09 |
| 968 | *Zfp9* | Zinc finger protein 9, mRNA (cDNA clone MGC:67724 IMAGE:2647280) | 2.02 |  |
| 969 | *Zkscan5* | Zinc finger protein 95 (Zfp95) | 2.49 |  |
| 970 | *Zmym1* | Zinc finger, MYM domain containing 1, mRNA (cDNA clone IMAGE:5038233) | 2.06 |  |
| 971 | *Zranb3* | Zinc finger, RAN-binding domain containing 3 (Zranb3), mRNA | 2.35 |  |
| 972 | *Zxdb* | Zinc finger, X-linked, duplicated B (Zxdb), mRNA |  | 2.17 |
| 973 | *Zzef1* | Zinc finger, ZZ-type with EF hand domain 1, mRNA (cDNA clone IMAGE:4194479) | 2.09 |  |
